# Experimental and Theoretical Exploration of the Kinetics and Thermodynamics of the Nucleophile-Induced Fragmentation of Ylidenenorbornadiene Carboxylates

**DOI:** 10.1021/acs.joc.3c00980

**Published:** 2023-08-03

**Authors:** Abigail D. Richardson, Scott J. L’Heureux, Ava M. Henry, Elizabeth A. McDonough, Cameron J. Fleischer, Cameron C. McMullen, Trevor R. Reynafarje, Gisele P. Guerrero, Quinn E. Williams, Qingyang Zhou, David M. Malouf, Spencer E. Thurman, Julia E. Soeller, Jerry Y. Sheng, Erica A. Medhurst, Angel E. Canales, Ty B. Cecil, K. N. Houk, Philip J. Costanzo, Daniel A. Bercovici

**Affiliations:** † Department of Chemistry and Biochemistry, California Polytechnic State University, San Luis Obispo, California 93407, United States; ‡ Department of Chemistry and Biochemistry, University of California, Los Angeles, Los Angeles, California 90095, United States

## Abstract

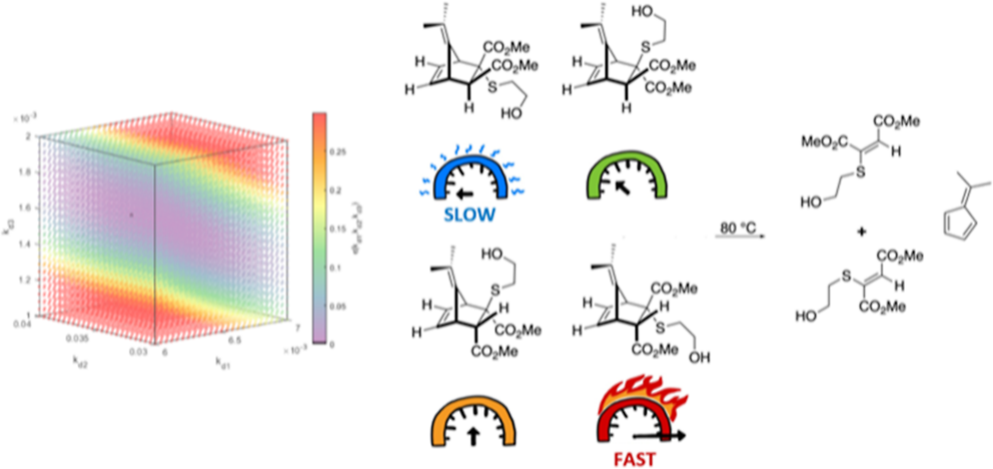

Ylidenenorbornadienes (YNDs), prepared by [4 + 2] cycloadditions between fulvenes and acetylene carboxylates, react with thiol nucleophiles to yield mixtures of four to eight diastereomers depending on the symmetry of the YND substrate. The mixtures of diastereomers fragment via a retro-[4 + 2] cycloaddition with a large variation in rate, with half-lives ranging from 16 to 11,000 min at 80 °C. The diastereomer-enriched samples of propane thiol adducts [YND-propanethiol (PTs)] were isolated and identified by nuclear Overhauser effect spectroscopy (NOESY) correlations. Simulated kinetics were used to extrapolate the rate constants of individual diastereomers from the observed rate data, and it correlated well with rate constants measured directly and from isolated diastereomer-enriched samples. The individual diastereomers of a model system fragment at differing rates with half-lives ranging from 5 to 44 min in CDCl_3_. Density functional theory calculations were performed to investigate the mechanism of fragmentation and support an asynchronous retro-[4 + 2] cycloaddition transition state. The computations generally correlated well with the observed free energies of activation for four diastereomers of the model system as a whole, within 2.6 kcal/mol. However, the observed order of the fragmentation rates across the set of diastereomers deviated from the computational results. YNDs display wide variability in the rate of fragmentation, dependent on the stereoelectronics of the ylidene substituents. A Hammett study showed that the electron-rich aromatic rings attached to the ylidene bridge increase the fragmentation rate, while electron-deficient systems slow fragmentation rates.

## Introduction

**Scheme 1:**
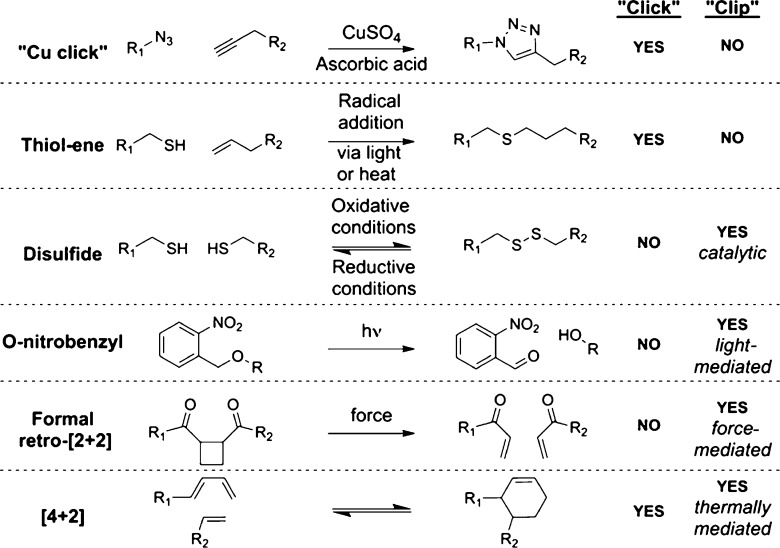
Various “Click” and “Clip” Reactions

**Scheme 2:**
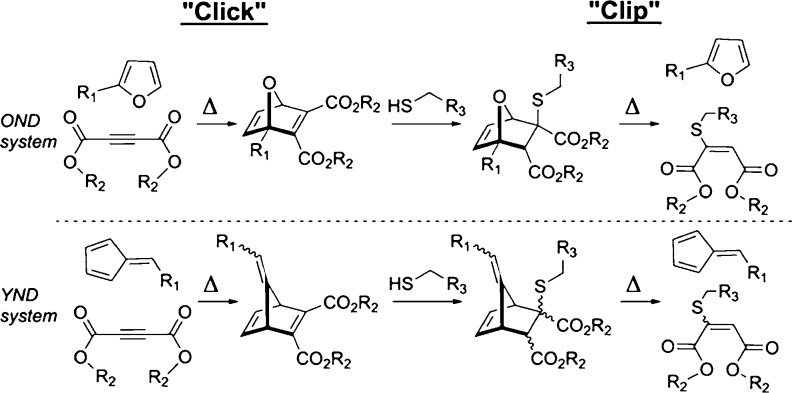
OND and YND Systems as “Click” and Nucleophile-Induced “Clip” Reactions

Transformations known to the chemistry community as “click” reactions have become immensely popular methods in many fields of chemistry for their ability to covalently bond two components in a modular and facile manner.^1^ The 2022 Nobel prize in chemistry, which was awarded to Sharpless, Meldal, and Bertozzi for their pioneering work in “click” chemistry and its applications, highlights the breadth and utility of this class of reactions.^2^ “Clip” reactions, as their name suggests, conversely offer the capability to break covalent bonds in a similarly facile and modular manner. A recent review by Johnson and co-workers divides “clip” reactions into six main classifications: stoichiometric, catalytic, electron-transfer-mediated, light-mediated, thermally mediated, and force-mediated.^3^ A list of commonly utilized “click” and “clip” reactions is given in Scheme 1. It is of interest to note that [4 + 2] cycloadditions and their retrograde reactions (retro-[4 + 2]) are able to react as both “click” and “clip” reactions, respectively. We previously utilized these reactions as dynamic covalent linkages to understand and manipulate polymer topology and solubility.^4−9^ Seminal work by the Finn group has shown that furans can react via [4 + 2] cycloadditions with alkyl acetylenedicarbonyls to yield oxanorbornadienes (ONDs), which can be fragmented via a retro-[4 + 2] cycloaddition after conjugate addition of an appropriate nucleophile.^10−14^ The furan component is thus “clicked” on to an alkyl acetylenedicarboxylate and subsequently “clipped” off again after reaction with the nucleophile, as depicted in Scheme 2. These OND “click-and-clip” systems have already shown utility as fluorogenic probes,^10,15^ pharmaceutical delivery systems,^13,16−18^ linkages in degradable hydrogels,^19,20^ and scaffolds toward the synthesis of difficult-to-prepare substituted heterocycles.^21,22^ We have further built on this “click-and-clip” strategy by reacting fulvene substrates in the place of furans with alkyl acetylenedicarboxylates to yield ylidenenorbornadienes (YNDs).^23^

Unlike their OND counterparts, YND substrates reacted with a thiol nucleophile, beta-mercaptoethanol (BME), to provide a complex mixture of diastereomers. These diastereomers subsequently showed marked differences in the fragmentation “clip” reaction rates. Herein, we build upon the scope of YND substrates as “click-and-clip” systems and describe our experimental and theoretical exploration into the kinetics and mechanism of fragmentation of YND-thiol diastereomer adducts.

## Results and Discussion

### Synthesis of YNDs

**Scheme 3:**
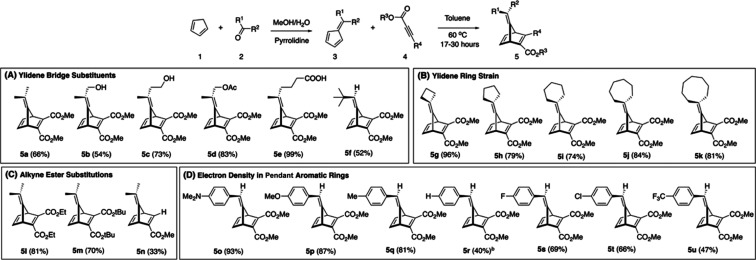
YND Carboxylate Substrate Scope for Experimental Kinetic Analysis Asymmetric YNDs were recovered as a racemic mixture. Yield taken over two steps from benzaldehyde.

Previously prepared YNDs (Scheme 3A,C—excluding **5e**), synthesized via [4 + 2] cycloadditions between fulvenes and acetylene carboxylates and dicarboxylates, provided yields from 33 to 83%. A new series of YNDs has been prepared (Scheme 3B,D) to further explore the scope and mechanism of the fragmentation. The yields of these YNDs were moderate to excellent (47–93%) with mild heating in toluene under an ambient atmosphere. 6-Aryl-substituted fulvenes generate YNDs in yields that trend up with increased electron density (Scheme 3D). Excellent yields were obtained from electron-rich phenyl rings (**5o** 93%), and yields decreased significantly with electron-poor phenyl rings (**5u** 47%). This result can likely be attributed to an increase in the favorability of competing fulvene dimerization via [4 + 2] cycloaddition, especially for electron-deficient fulvenes.^24^ During chromatography of **5u**, a fulvene dimer was observed as a minor side product.

### Model System Kinetics

**Figure 1:**
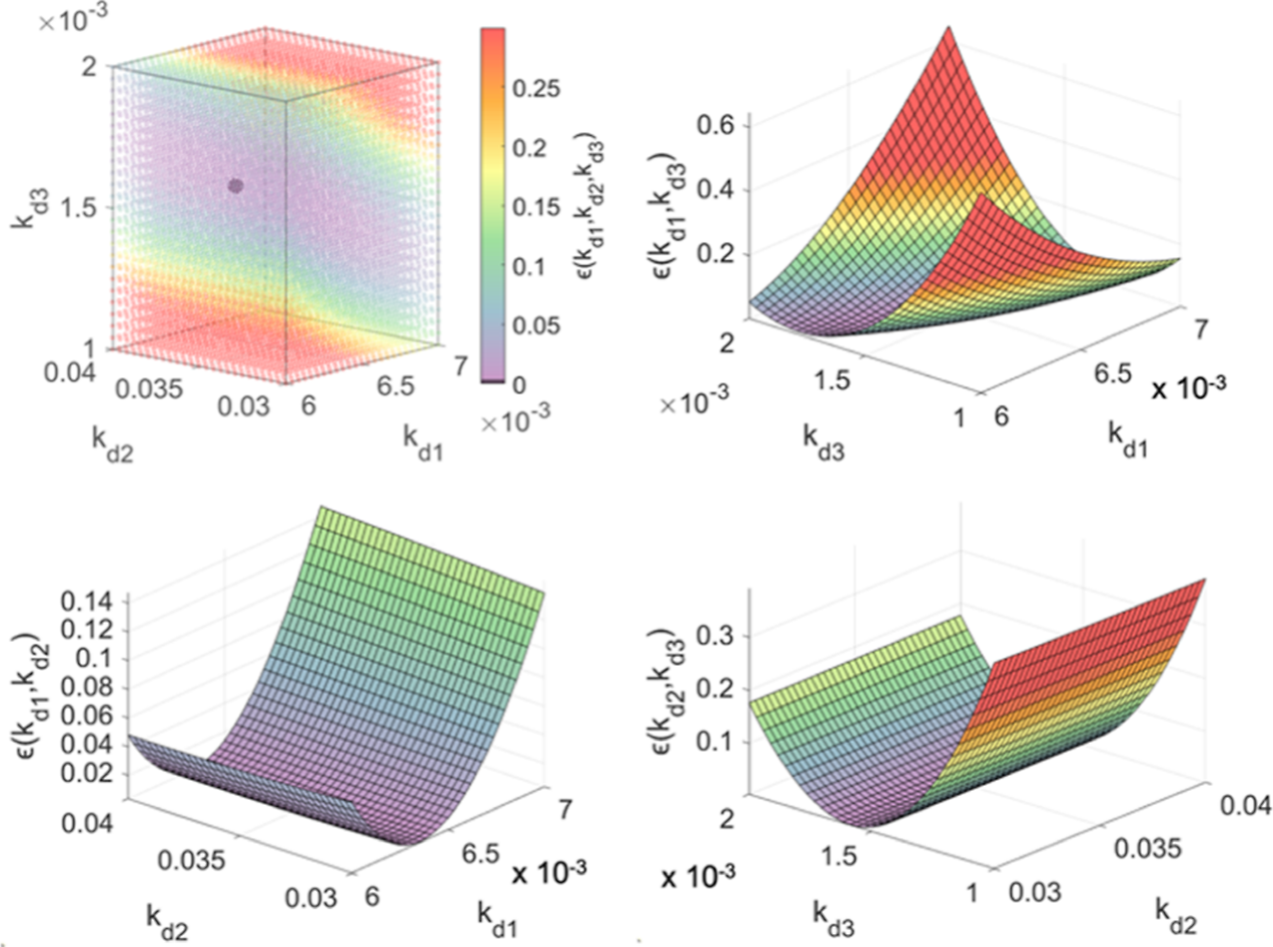
Minimum of the representative error function to extrapolate rate constants from convoluted kinetic data and three 3-dimensional slices of the 4-dimensional plot, where *k*_**d3**_, *k*_**d2**_, and *k*_**d1**_ were held constant, respectively.

**Scheme 4:**
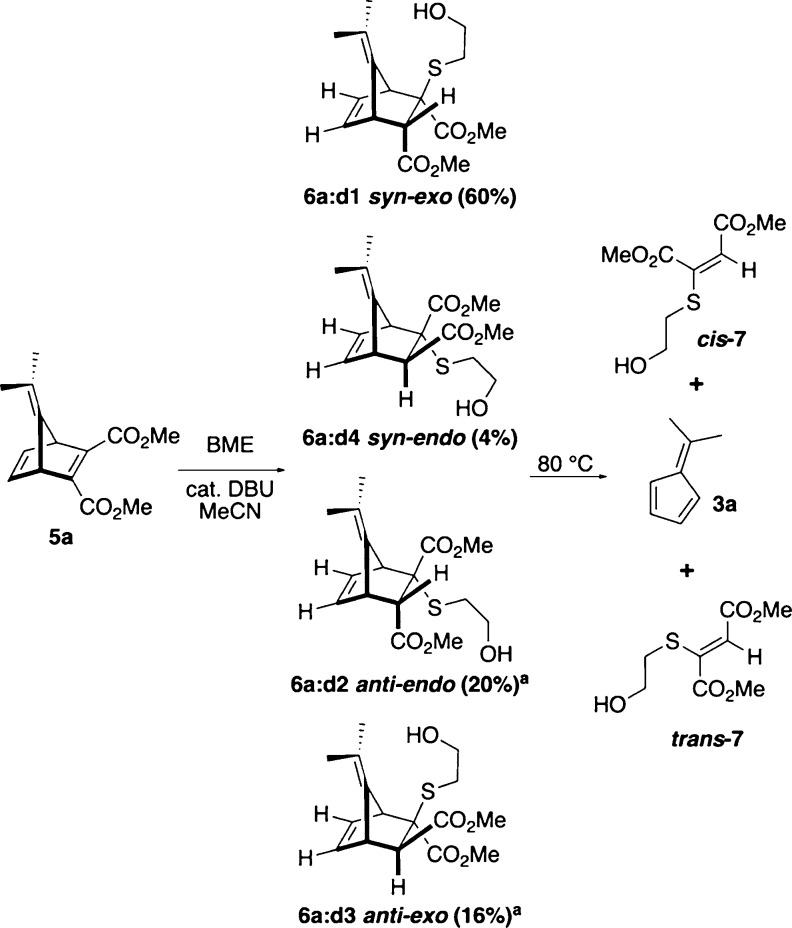
Preparation and Fragmentation of YND–BME Diastereomers **6a:d1-4** As described later in this report, the assignment of diastereomers **d2** and **d3** has been reversed from our previous report.

Nucleophile-induced fragmentation on the model YND system **5a** was analyzed after conjugate addition with BME catalyzed by 1,8-diazabicyclo[5.4.0]undec-7-ene (DBU). The YND–BME adduct **6a** displayed a complex mixture of four diastereomers **6a:d1-4**, as seen in Scheme 4. As previously reported,^23^ three diastereomers **6a:d1-d3** of the model system YND–BME provided overlapping signals in the ^1^H NMR spectrum, and individual diastereomer rate constants could not be directly measured using DMSO-*d*_6_ as the solvent. Diastereomer **6a:d4** did provide an isolated resonance in the ^1^H NMR spectrum but was produced as only 4% of the total mixture, and relative to diastereomers **6a:d1-d3**, **6a:d4** fragmented too slowly at 80 °C for accurate kinetic data to be obtained in the same experiment. Therefore, diastereomer **6a:d4** was not experimentally investigated further for fragmentation kinetic analysis. The integrated first-order rate plot resulting from the combination of degradation kinetics of diastereomers **6a:d1-d3** appeared curved (Figure S19), suggesting that diastereomers were fragmenting at different rates. With the aid of kinetic simulations utilizing a genetic algorithm and the simplex method,^25^ we were able to extrapolate the individual first-order rate constants from the observed convoluted data (Figure 1).

**Figure 2:**
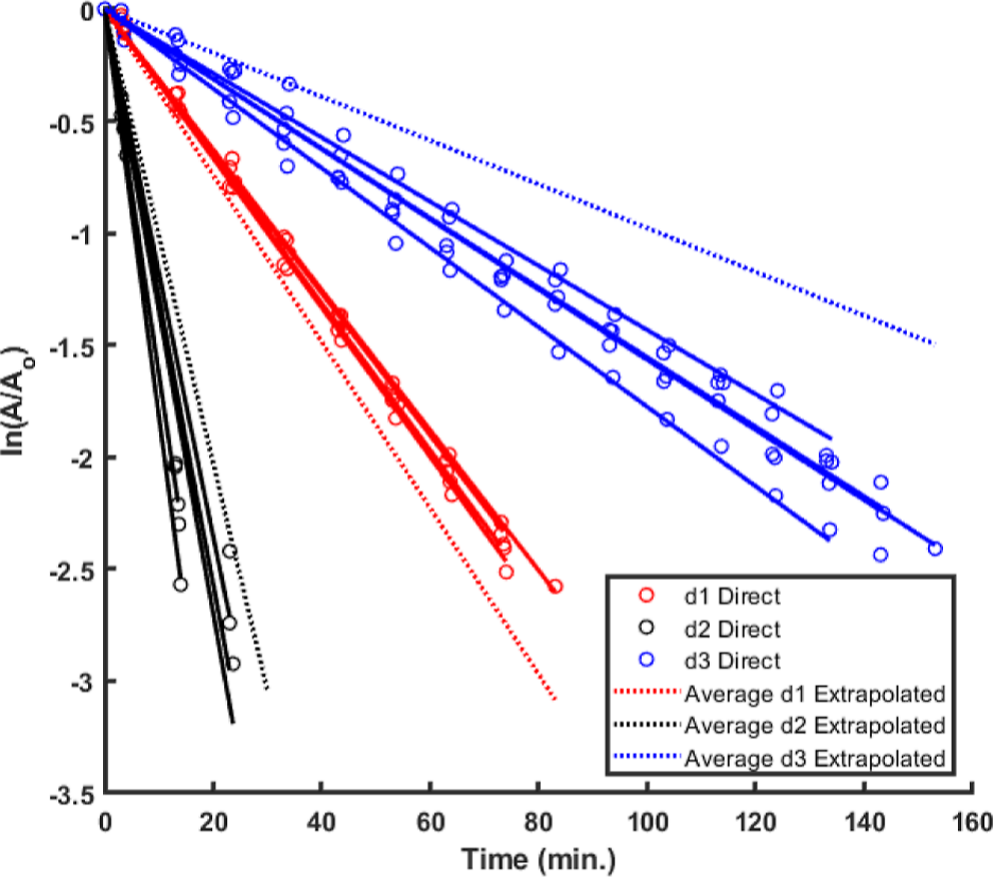
Comparison of integrated first-order kinetics of directly measured and extrapolated retro-[4 + 2] cycloadditions of diastereomers **6a**:**d1-d3** in CDCl_3_.

In an effort to validate the extrapolation method, we directly evaluated the kinetics of the fragmentation of individual diastereomers **6a:d1-d3** utilizing CDCl_3_ as the solvent. In CDCl_3_, the integration of individual diastereomer resonances resolved enough for the fragmentation kinetics to be measured directly by observing the disappearance of the individual signals assigned to diastereomers **6a:d1-3** (Figure S20). Kinetic simulations were then applied to extrapolate individual diastereomer fragmentation rate constants from the convoluted kinetics of the combined ^1^H NMR integration values of diastereomers **6a:d1-d3**. Comparison of these two methods (Table 1 and Figure 2) substantiated the use of the genetic algorithm and simplex method to adequately extrapolate the individual diastereomer kinetic rates from the convoluted kinetics of the complex mixtures. Extrapolated rate constants matched the directly measured rate constants fairly well for **6a:d1** and **d2** as both the extrapolated values were within 20% of the directly measured values. The value for the **6a:d3** diastereomer was within 40%. We were unable to chromatographically separate the mixture of diastereomers for YND–BME **6a** and thus could not directly measure individual rate constants in DMSO-*d*_6_.

**Table 1:** Comparison of Directly Determined and Extrapolated Mean Kinetic Rate Constants of Model System YND–BME **6a:d1-d3** in CDCl_3_

method	**6a:d1**1 avg *k* (s^–1^) *t*_1/2_ (min)	**6a:d2** avg *k* (s^–1^) *t*_1/2_ (min)	**6a:d3** avg *k* (s^–1^) *t*_1/2_ (min)
direct	5.38 ± 0.18 × 10^–4^	2.08 ± 0.20 × 10^–3^	2.64 ± 0.32 × 10^–4^
	21.5 ± 0.7	5.6 ± 0.5	44.0 ± 5.3
extrapolated	6.19 ± 0.53 × 10^–4^	1.69 ± 0.67 × 10^–3^	1.63 ± 0.37 × 10^–4^
	18.7 ± 1.5	7.6 ± 3.8	73.9 ± 17.8

### Substrate Scope

#### Ylidene Bridge Substituent Kinetics

Substituent effects were first explored through the modification of the functional groups atop the ylidene bridge (Table 2). As explained in our previous findings, a decrease in electron density at the ylidene bridge and changes in diastereomer populations affected the observed rates of the mixture of diastereomers.^23^ Compared to the model system **6a** (*t*_1/2_ = 31.1 min), pendent alcohol substrates **6b** (*t*_1/2_ = 89.6 min) and **6c** (*t*_1/2_ = 53.2 min), and the acyl-substituted **6d** fragmented at slower rates, with the most electron-deficient acyl-substituted **6d** providing the longest half-life (*t*_1/2_ = 226 min). Substrates **6b**, **6d**, and **6e**^1^H NMR spectra allowed for the determination of diastereomeric ratios **d1–d4** based on assignments from the model systems **6a** and **8a** (vide infra), suggesting that the *E*/*Z* stereocenter atop the ylidene bridge had little effect on the proton resonances used for diastereomer assignment. As such, when the extrapolation algorithm was applied to the observed kinetics of the mixture of diastereomers for substrates **6b**, **6d**, and **6e**, the results (Table 2) confirmed that individually, all the extrapolated rate constants for diastereomers **d1–d3** of these substrates decrease with electron-withdrawing ylidene bridge substituents. However, YND–BMEs **6c** and **6f** were recovered as a complex mixture of eight diastereomers that could not be resolved in the ^1^H NMR spectra. Thus, our extrapolation technique could not be applied to compare individual diastereomer rate constants.

**Table 2:**
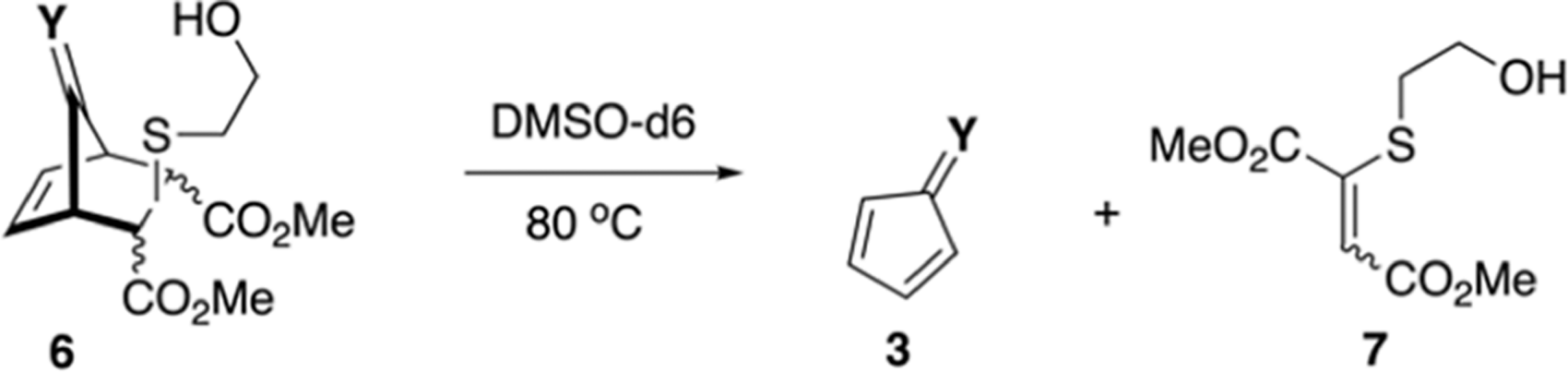

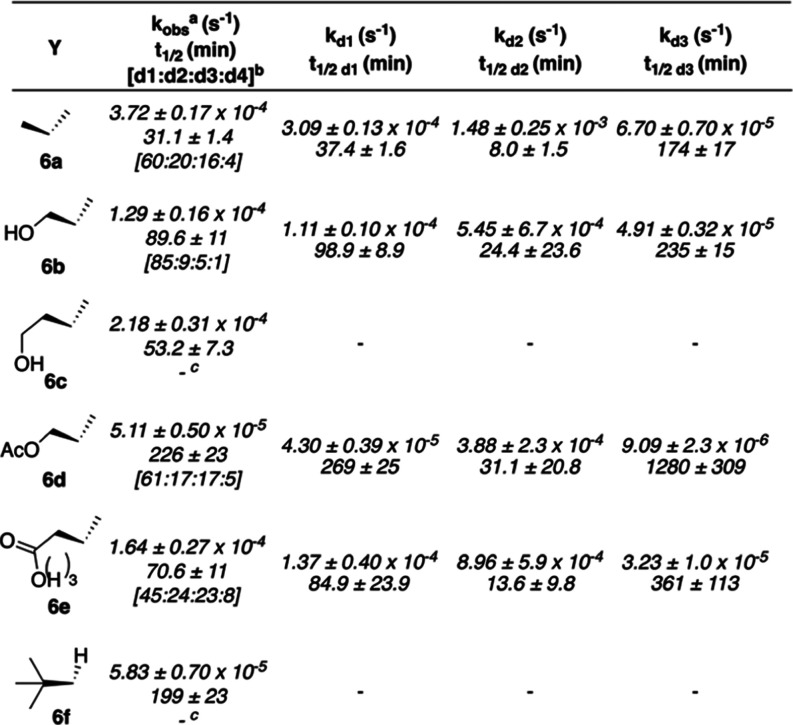
Ylidene-Bridge Substituent Effects on YND–BME Fragmentation Kinetics

a Our previous report^23^ provided *k*_obs_ values from linear fits of the curved integrated rate data for the fragmentation of the mixture of diastereomers taken to 90% completion. Herein, we report more representative *k*_obs_ values obtained from the reaction taken to 50% completion.

b Diastereomeric ratios determined by ^1^H NMR.

c A complex mixture of eight diastereomers with overlapping ^1^H NMR resonances was observed.

#### Ring Strain Kinetics

A near 50% drop in the observed half-life in YND–BME **6h** (*t*_1/2_ = 17.0 min) versus the model system **6a** (*t*_1/2_ = 31.1 min) was observed when the ylidene bridge was connected to a cyclopentane ring. It is well known that the increase in the carbonyl IR stretching frequency of cyclic ketones coincides with an increase in ring strain. This is largely a mechanical effect attributed to the change in C–C(=O)–C bond angle provided by the strain of the ring and not a large change in the C=O force constant.^26,27^ This effect ultimately leads to the shortening of the C=O bond and thus a higher stretching frequency. The same effect is observed in analogous exocyclic alkenes. Since YND–BME fragmentation was affected by delocalization/polarization of the C=C by the electron-withdrawing functional groups adjacent to the ylidene bridge (**6b** and **6d**; Table 2), we imagined that the ring strain on the bridging ylidene would shorten the C=C bond and effectively increase the electron density in the alkene and increase fragmentation rates. Thus, we explored a series of cyclic YND–BME substrates **6g–k** (Table 3). As expected, the highly strained four- and five-membered ring substrates **6g** and **6h** had significantly shorter half-lives, 15.6 and 17.0 min, respectively, than the acyclic model system **6a** with a half-life of 31.1 min. The extrapolated rate constants for the individual diastereomers also fit this trend. Strain was reduced in the six-membered system **6i**, which provided a larger half-life than that in the model system of (44.0 min). As the bond angle of the C=C(Y) ylidene increased further with the seven- and eight-membered rings of **6j** and **6k**, the half-lives increased as well compared to that of the model system to 52.5 and 50.3 min, respectively. Extrapolated diastereomer rate constants showed that slower observed rates of fragmentations of the diastereomeric mixtures in **6i–k** were largely due to increased populations of the slower **d3** diastereomer, and the individual diastereomer rate constants were consistently slower as the ring size increased.

**Table 3:**
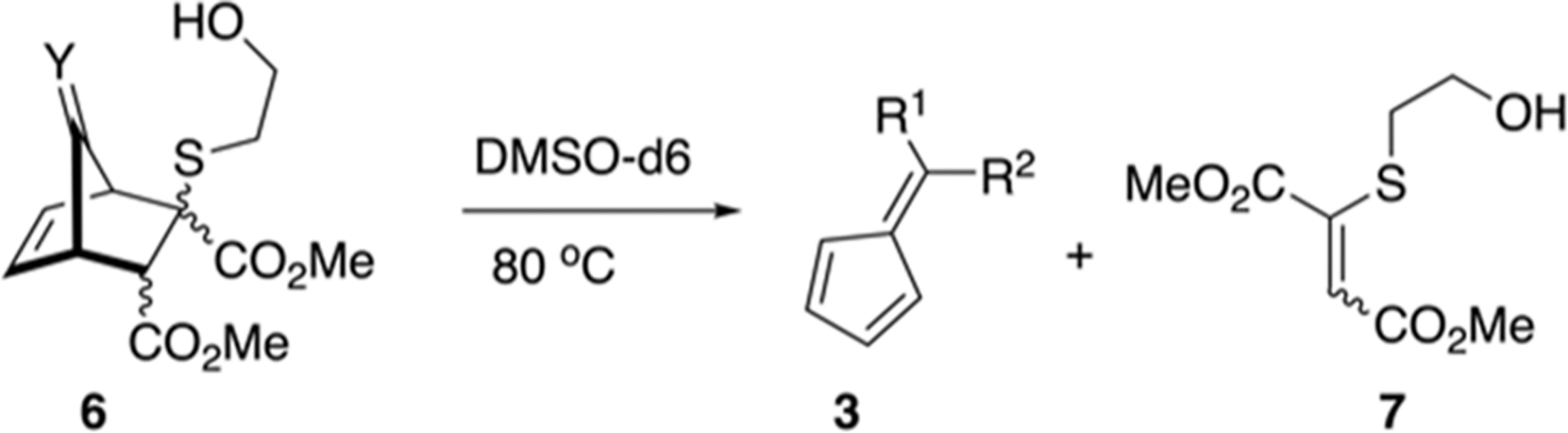

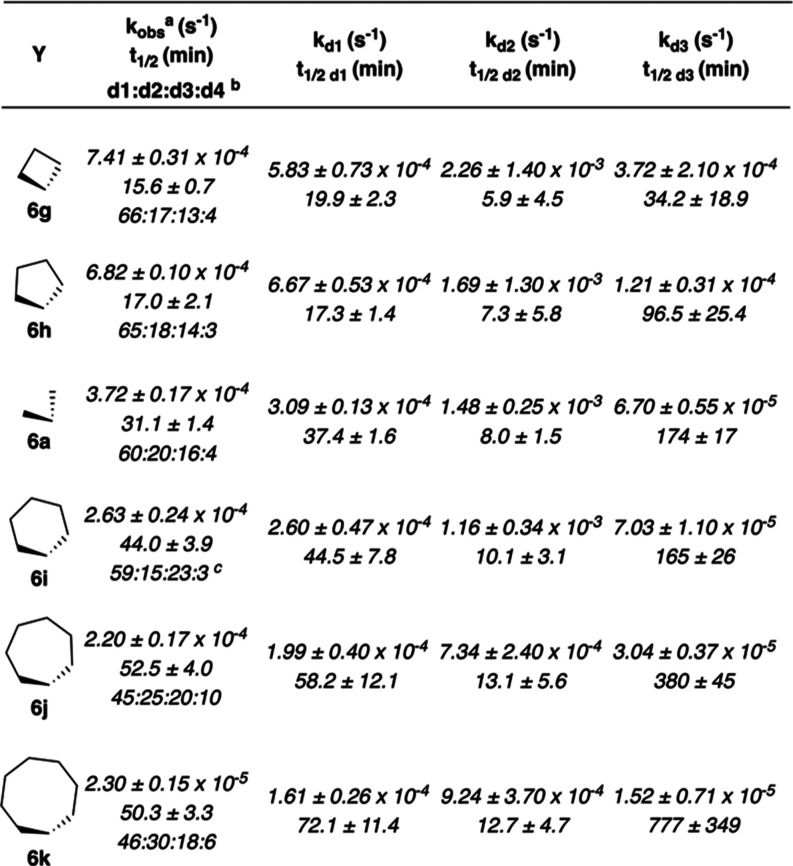
Ylidene-Bridge Ring-Strain Effects on YND–BME Fragmentation Kinetics

a Our previous report^23^ provided k_obs_ values from linear fits of the curved integrated rate data for the fragmentation of the mixture of diastereomers taken to 90% completion. Herein, we report more representative k_obs_ values obtained from the reaction taken to 50% completion.

b Diastereomer ratios were determined from ^1^H NMR resonances of alkene protons.

c ^1^H NMR resonances for the signals corresponding to **d1** and **d2** were slightly overlapping.

#### Ester Kinetics

Our previous report indicated that an increase in the size of the ester substituent led to a decrease in the observed rate constant for the diastereomeric mixture.^23^ We postulated that this was due to larger proportions of the slower-to-fragment **d3** diastereomer. We can now confirm that the extrapolated rate data for each individual diastereomer agreed with this reasoning. The individually extrapolated diastereomer rate constants remained consistent across the series **6a**, **6l**, and **6m**, as seen in Table 4. The diastereomeric ratio of monoester substrate **6n** could not be elucidated and therefore does not have comparable diastereomer data. However, the observed rate constant was significantly (3 orders of magnitude) slower than any of the diester rate constants, and the observed half-life of the diastereomeric mixture was measured to be 11,000 min.

**Table 4:**
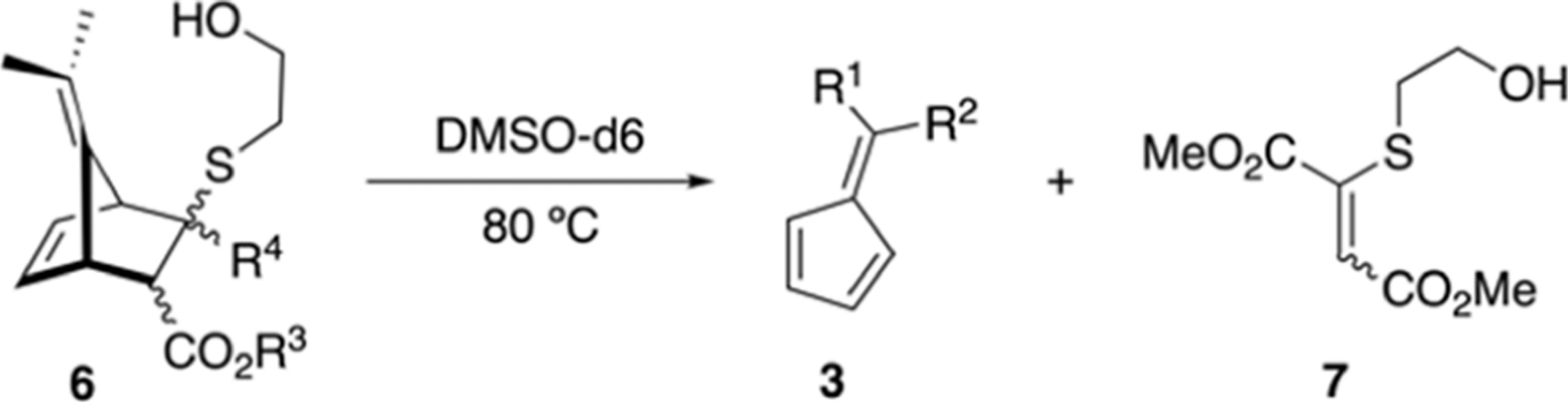

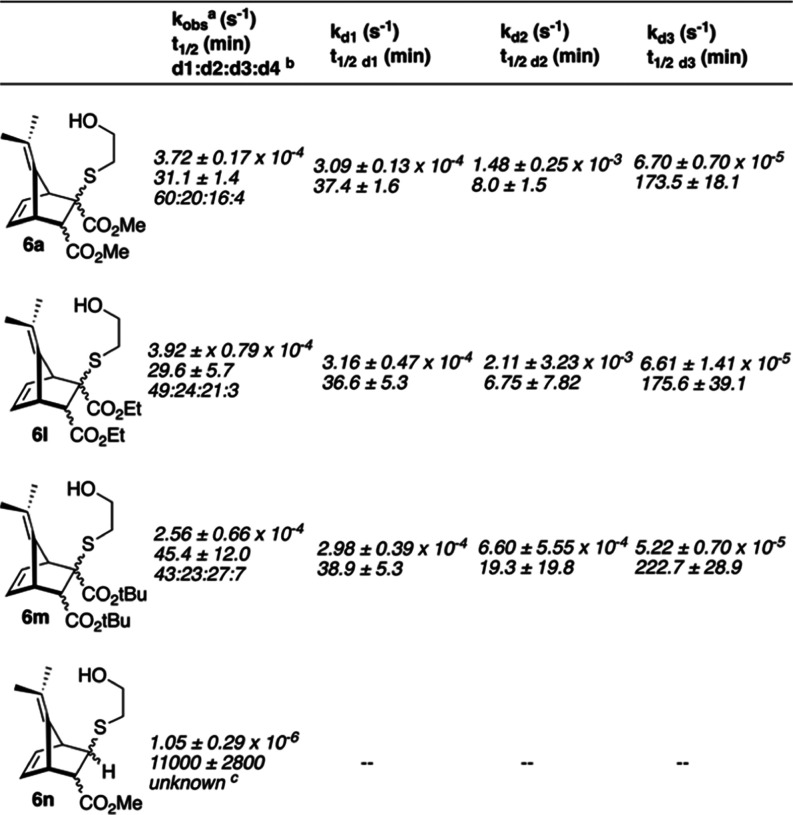
Effect of Ester Substitution on Fragmentation Kinetics of YND–BMEs

a Our previous report^23^ provided *k*_obs_ values from linear fits of the curved integrated rate data for the fragmentation of the mixture of diastereomers taken to 90% completion. Herein, we report more representative *k*_obs_ values obtained from the reaction taken to 50% completion.

b Diastereomer ratios were determined from ^1^H NMR resonances of alkene protons.

c Diastereomer ratios of **6n** were unable to be determined from the ^1^H NMR spectrum.

### Diastereomer Assignment

Our preliminary report explained our diastereomer assignment of **6a:d1-4** based on two factors: (1) the precedence in the OND systems^11^ and (2) the relative rate of appearance and abundance of *cis*- versus *trans*-**7** (the maleate and fumarate isomers, respectively) in the fragmentation products. No published spectra existed confirming the stereochemical configuration of the maleate/fumarate products of fragmentation. Our original assignment of the *syn* versus *anti* diastereomers was further confirmed by identifying *cis***-7** (maleate) as the major isomer of fragmentation through NOESY correlation (Figure S3), solidifying the major diastereomer **6a:d1** as an *anti* diastereomer (see the Supporting Information for more details).

**Figure 3:**
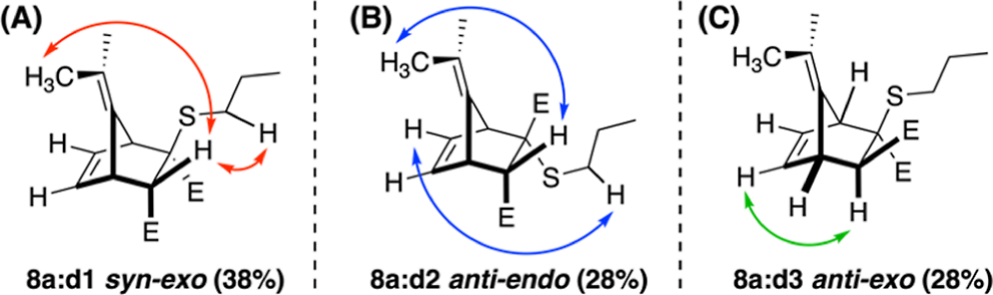
Observed NOESY correlations elucidating the structures of diastereomers **8a:d1-d3** [E = CO_2_Me].

**Scheme 5:**
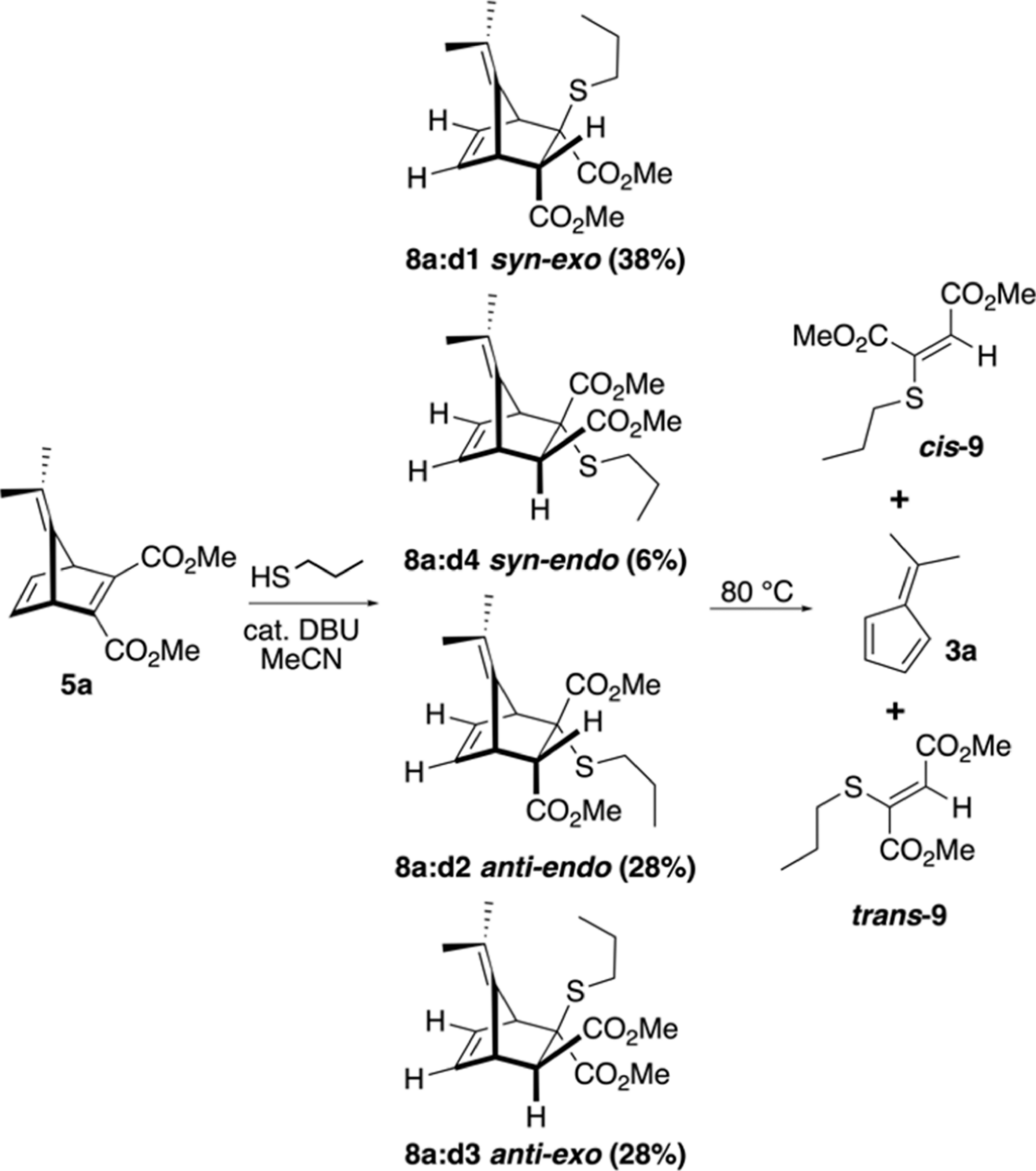
Preparation and Fragmentation of YND-PT Diastereomers **8a:d1-4**

While exploring propanethiol (PT) as a steric analogue for BME as a nucleophile (vide infra), a diastereomeric mixture of YND-PT adducts (**8a**, Scheme 5) was prepared. The YND-PT system proved chromatographically separable into three fractions, eachenriched with diastereomer **8a:d1**, **d2**, and **d3**. NOESY correlations between the exo-proton alpha to the methyl ester and the methyl protons on the top of the ylidene bridge and the alpha protons of the thioether chain confirmed the structure of diastereomer **8a:d1** as the *syn*-exo diastereomer (Figure 3A). This was the predominantly formed diastereomer (38% of the diastereomeric mixture) and the second fastest to fragment in DMSO-*d*_6_ (*t*_1/2_ = 34.7 min; Table 5). A NOESY correlation between the exo-proton alpha to the methyl ester and the methyl protons on the top of the ylidene bridge and a correlation between the alpha protons of the thioether chain and the olefin proton identified the structure of diastereomer **8a:d2** as the *anti*-endo diastereomer and was the fastest to fragment in DMSO-*d*_6_ (*t*_1/2_ = 5 min; Table 5) (Figure 3B). Finally, a NOESY correlation between the endo-proton alpha to the methyl ester and the olefin proton confirmed the structure of diastereomer **8a:d3** as *anti*-exo (Figure 3C) and was the slowest to fragment (*t*_1/2_ = 139 min; Table 5) (see the Supporting Information for full analysis of NOESY spectra).

**Table 5:** Extrapolated and Isolated Kinetic Rate Constants of YND-PT Diastereomers **8a:d1–d3** in DMSO-*d*_6_

system (solvent) method	**d1**	**d2**	**d3**
	*k*_**d1**_ (s^–1^)	*k*_**d2**_ (s^–1^)	*k*_**d3**_ (s^–1^)
	*t*_1/2_ (min)	*t*_1/2_ (min)	*t*_1/2_ (min)
**8a** (DMSO-*d*_6_) extrapolated	3.28 ± 0.25 × 10^–4^	1.66 ± 0.23 × 10^–3^	6.83 ± 0.68 × 10^–5^
	35.3 ± 2.5	7.0 ± 1.0	170 ± 17
**8a** (DMSO-*d*_6_) isolated	3.33 ± 0.25 × 10^–4^	2.33 ± 0.15 × 10^–3^	8.33 ± 1.02 × 10^–5^
	34.7 ± 2.6	5.0 ± 0.3	139 ± 17

### Computational Investigation

To further investigate the fragmentation kinetics of the model YND system **6a**, the free energy of activation of a retro-[4 + 2] cycloaddition reaction was calculated for each diastereomer **6a:d1-4**. Our initial report provided assignments for the **6a:d2** and **6a:d3** diastereomers that were incorrect. The assignment of the YND-PT **8a** diastereomers was used to correct the assignment of the YND–BME **6a** system, exchanging the initial structures of **6a:d2** and **6a:d3**. However, before this correction, we explored four alternate mechanisms, trying to find a lower energy transition state to explain the large discrepancy in the experimental and computational free energies of activation for the *anti*-exo diastereomer. This exploration of the potential energy surface (PES) can be found in the Supporting Information. DFT calculations were performed using GAMESS 2020 R1.^28^ Geometries were first optimized using the density functional ωB97X-D^29^ and the 6-31+G(d) basis set, and the solvation effects were corrected for using DMSO implicit solvation^30^ and the 6-311+G(d,p) basis set. Conformational searches were performed using CREST,^31^ and the generated conformers were re-ranked using single-point calculations with ωB97X-D/6-311+G(d,p) and DMSO implicit solvent. Later, for comparison, geometries were also optimized using the density functionals, M06-2X^32^ and B3LYP,^33,34^ and the 6-31+G(d) basis set. Solvation effects were again corrected for using DMSO implicit solvation and the 6-311+G(d,p) basis set. Free energies were also further corrected by single-point calculations using domain-based local pair natural orbital coupled cluster method DLPNO-CCSD(T)^35,36^ and the def2-TZVPP basis set performed by ORCA v5.0^37,38^ based on geometries optimized by all three functionals (see the Supporting Information for more details).

**Figure 4:**
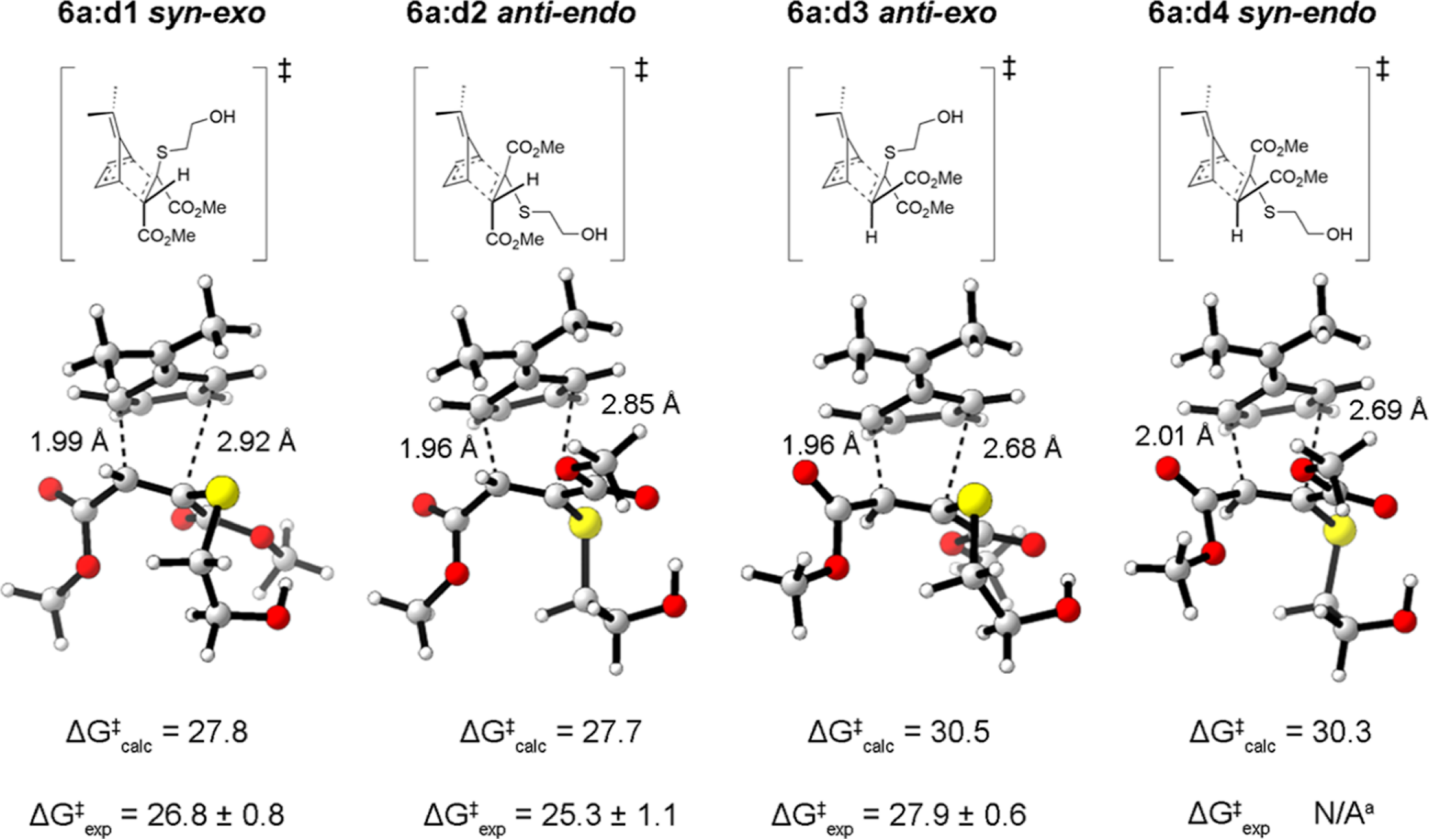
Corrected assignment of diastereomers with calculated and experimental free energies of activation (ωB97X-D). Energies in kcal/mol. Energies were calculated at 25 °C and 1 atm with DMSO implicit solvent, and the experimental energies were calculated at 25 °C using the parameters from Arrhenius plot.^23^^a^experimental free energy of activation for **6a:d4** was not measured because this diastereomer was rerecovered as only 4% of the total diastereomeric mixture, and it fragmented much slower relative to the other three diastereomers.

For the model YND system **6a**, using the DFT functional ωB97X-D, the free energy of activation for the retro-[4 + 2] cycloaddition in kcal/mol for **6a:d1** is 27.8, **6a:d2** is 27.7, **6a:d3** is 30.5, and **6a:d4** is 30.3 (Figure 4). Examining these computational free energies of activation, the values for **6a:d1** and **6a:d2** are nearly identical, as are **6a:d3** and **6a:d4**. This suggests that the density functional ωB97X-D is correctly differentiating the two diastereomers that are faster to fragment from the two diastereomers that are slower to fragment but cannot adequately distinguish them further. Furthermore, using the density functional ωB97X-D provides a relatively accurate estimate for the experimental free energy of activation of diastereomers **6a:d1**, **6a:d2**, and **6a:d3** individually as the energies are within 1.0, 2.4, and 2.6 kcal/mol, respectively. We next utilized the density functionals M06-2X and B3LYP for comparison (Table 6). The density functional B3LYP only identified the two faster fragmenting diastereomers and the two slower fragmenting diastereomers, but the calculated free energies of activation for each diastereomer were more than 10 kcal/mol lower than the experimental values. The density functional M06-2X was overall more accurate when comparing **6a:d1**, **6a:d2**, and **6a:d3** individually with the experimental free energies of activation as the energies were within 1.9, 1.2, and 0.1 kcal/mol, respectively. However, the DFT functional M06-2X identified **6a:d1** and **6a:d2** as faster to fragment than the other two diastereomers (**6a:d3** and **6a:d4**) but calculated the free energy of activation for the fragmentation of **6a:d1** to be to be 1.6 kcal/mol lower than that of **6a:d2**, which is the opposite of what is observed experimentally. Likewise, with the M06-2X functional, **6a:d4** is calculated to be faster to fragment than **6a:d3**, contrary to the experimental data. Finally, we looked at correcting the structures optimized by each DFT functional with single-point calculations using a DLPNO-CCSD(T)) and the basis set def2-TZVPP. Corrections on the B3LYP-optimized values greatly helped improve the similarity between the experimental and calculated free energies of activation for all four diastereomers, with all the computational values being within a difference of 1.2–5 kcal/mol of the experimental values. However, none of the corrected values matched the relative ranking of the experimental free energies of activation for fragmentation across the four diastereomers. Regardless of which DFT functional optimized the structures of diastereomers, the DLPNO-CCSD(T)/def2-TZVPP-corrected energies mis-ranked the free energies of activation for the fragmentation of the two faster and two slower diastereomers (Table 6). Therefore, out of the three density functionals examined and DLPNO-CCSD(T) corrections, the uncorrected ωB97X-D best describes the activation barriers of fragmentation for **6a:d1–d4** relative to one another, but the functional is still unable to identify the stereoelectronic effects leading to a difference between the two fast diastereomers (**6a:d2** and **6a:d1**) and between the two slower diastereomers (**6a:d3** and **6a:d4**). We are continuing to study this system both experimentally and computationally in an effort to discern the factors leading to the difference in diastereomer rates of fragmentation.

**Table 6:** Comparison of DFT Functionals for Computed **6a:d1-4** TS Energies^a^

	experimental	B3LYP			M06-2X			ωB97X-D		
diast.	Δ*G*^‡^	Δ*G*^‡^	nascent bond lengths (Å)	DLPNO-CCSD(T) corrected Δ*G*^‡^	Δ*G*^‡^	nascent bond lengths (Å)	DLPNO-CCSD(T) corrected Δ*G*^‡^	Δ*G*^‡^	nascent bond lengths (Å)	DLPNO-CCSD(T) corrected Δ*G*^‡^
**6a:d1**	26.8	15.2	1.95/3.09	29.4	24.9	1.94/2.78	28.1	27.8	1.99/2.92	28.6
**6a:d2**	25.3	15.7	1.90/3.05	31.6	26.5	1.92/2.73	29.7	27.7	1.96/2.85	30.1
**6a:d3**	27.9	18.5	1.88/2.84	34.3	28.0	2.03/2.28	30.0	30.5	1.96/2.68	32.6
**6a:d4**	—b	17.5	1.92/2.86	31.3	27.2	2.02/2.53	29.9	30.3	2.01/2.69	30.9

a Structures and vibrational frequencies were optimized with the 6-31+G(d) basis set, and electronic energies were calculated with the 6-311+G(d,p) basis set and DMSO implicit solvent. Δ*G*^‡^ values are given in kcal/mol.

b The experimental free energy of activation for **6a:d4** was not measured because this diastereomer was rerecovered as only 4% of the total diastereomeric mixture, and it fragmented much slower relative to the other three diastereomers.

### Solvent Effects and Hydrogen-Bonding

**Figure 5:**
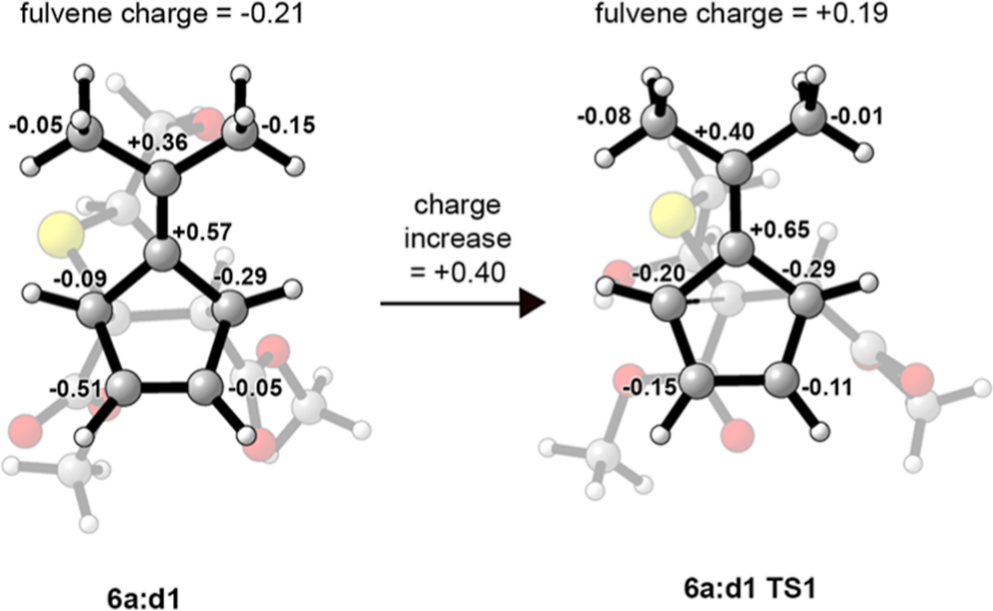
Charge distribution in **6a:d1** and **TS1-6a:d1**. Charges in electrons and hydrogens summed into carbons.

In the course of exploring the model system **6a**, the rate constants were experimentally measured to be higher with chloroform-*d* as the solvent versus DMSO-*d*_6_ for all three diastereomers (Table 7). This contradicts the hypothesis that a more polar solvent would stabilize the buildup of charge in the computed asynchronous transition state structures of a retro-[4 + 2] cycloaddition reaction.^39^ While the degree of asynchronicity was observed to vary depending on the DFT functional chosen as seen in the ratio of nascent bond lengths (Table 5), with the B3LYP functional computing the most asynchronous transition state structures for all diastereomers, all functionals did provide asynchronous transition state structures. The major diastereomer for the model system was examined with respect to Mulliken charges,^40^ using the ωB97X-D functional, in the reactant (**6a:d1**) and the transition state structure **TS1**. As seen in Figure 5, the fulvene portion has an increase in positive charge of 0.40 e on going from YND–BME **6a:d1** to the transition state structure **TS1**. These computational results suggest that in the transition state structure, there is an increase in positive charge on the fulvene portion of the structure and thus, an equal increase of negative charge in the forming alkene portion of the structure.

**Table 7:** Comparison of Kinetic Rate Constants of YND–BME **6a:d1-d3** and YND-PT **8a:d1-d3** in CDCl_3_ and DMSO-*d*_6_

system (solvent) method	diastereomer		
	*k*_**d1**_ (s^–1^)	*k*_**d2**_ (s^–1^)	*k*_**d3**_ (s^–1^)
**6a** (DMSO-*d*_6_) extrapolated^a^	3.09 ± 0.13 × 10^–4^	1.48 ± 0.25 × 10^–3^	6.70 ± 0.65 × 10^–5^
**8a** (DMSO-*d*_6_) isolated^b^	3.33 ± 0.25 × 10^–4^	2.33 ± 0.15 × 10^–3^	8.33 ± 1.02 × 10^–5^
**6a** (CDCl_3_) direct^c^	5.38 ± 0.18 × 10^–4^	2.08 ± 0.20 × 10^–3^	2.64 ± 0.32 × 10^–4^
**8a** (CDCl_3_) isolated^b^	2.75 ± 0.14 × 10^–4^	2.30 ± 0.07 × 10^–3^	8.47 ± 0.46 × 10^–5^

a Rate constants are extrapolated from combined convoluted rate data of a mixture of diastereomers **d1–d3** using kinetic simulations.

b Rate constants are derived from kinetics measured of isolated diastereomer-enriched samples.

c Rate constants are derived from kinetics measured from the direct measurement of single resolvable diastereomer ^1^H NMR resonances in a mixture of diastereomers **d1–d3**.

**Figure 6:**
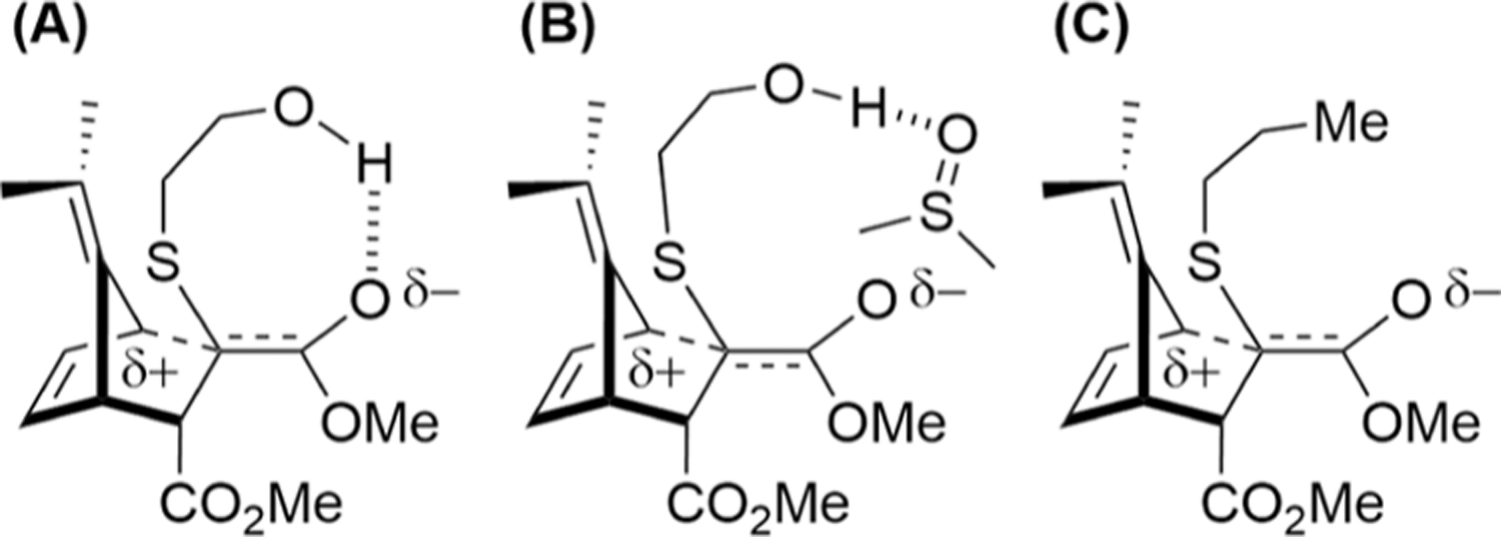
Transition state charge buildup of YND–BME **6a** in (A) CDCl_3_, (B) DMSO-*d*_6_, and (C) YND-PT **8a** (only the **d1** diastereomer is shown for clarity).

We postulated that an intramolecular hydrogen bond between the pendant alcohol of the added BME and the proximal ester was able to stabilize the buildup of negative charge on the carbonyl oxygen, thus lowering the activation energy. Evidently, this hydrogen-bond stabilization was only occurring in CDCl_3_ (Figure 6A). In DMSO, this pendant alcohol would be solvated (Figure 6B). To test this hypothesis, we experimentally studied the YND-PT system **8a** (Figure 6C) as a steric analogue of the YND–BME system, which could not exhibit this hydrogen-bond stabilization.

Model YND **5a** was reacted with PT as a nucleophile with the DBU catalyst in acetonitrile and resulted in a diastereomeric mixture of YND-PT **8a:d1-4**. As mentioned previously, it was possible to chromatographically isolate the individual fractions enriched with diastereomers **8a:d1**, **d2**, and **d3**. The mixture of diastereomers and the isolated samples of diastereomers were each subjected to kinetic fragmentation and extrapolation experiments in DMSO-*d*_6_ (Scheme 5).

The results of extrapolated rate constants derived from our kinetic simulation method aligned very well with the isolated diastereomer rate constants. As shown in Table 5, the extrapolated values for the rate constant *k*_**d1**_ are within 3% of the isolated individual diastereomer rate constant, *k*_**d3**_ is within 12%, and the rate constant *k*_**d2**_ is within 30% of the isolated individual diastereomer rate constant. These results further substantiate our kinetic simulations as a viable tool for extrapolating rate constants from convoluted observed kinetic data.

The YND-PT system **8a** was also fragmented and analyzed in CDCl_3_. YND-PT **8a** fulfilled the expectation that the more polar DMSO solvent would result in an increase in the rate of fragmentation only in **8a:d1** with the rate constant modestly increasing from 2.75 × 10^–4^ to 3.33 × 10^–4^ s^–1^ (Table 7). Furthermore, the respective rate constants, of each diastereomer of **6a** and **8a** in DMSO were observed to be roughly equivalent, for example, **6a:d1**; *k* = 3.09 × 10^–4^ and **8a:d1**; *k* = 3.33 × 10^–4^ (Table 7), suggesting that in DMSO, the pendant alcohol is not undergoing hydrogen bonding with the carbonyl oxygen, rendering the two systems effectively equivalent with regard to the stabilization of charge by the pendant alcohol.

### Hammett Study

**Figure 7:**
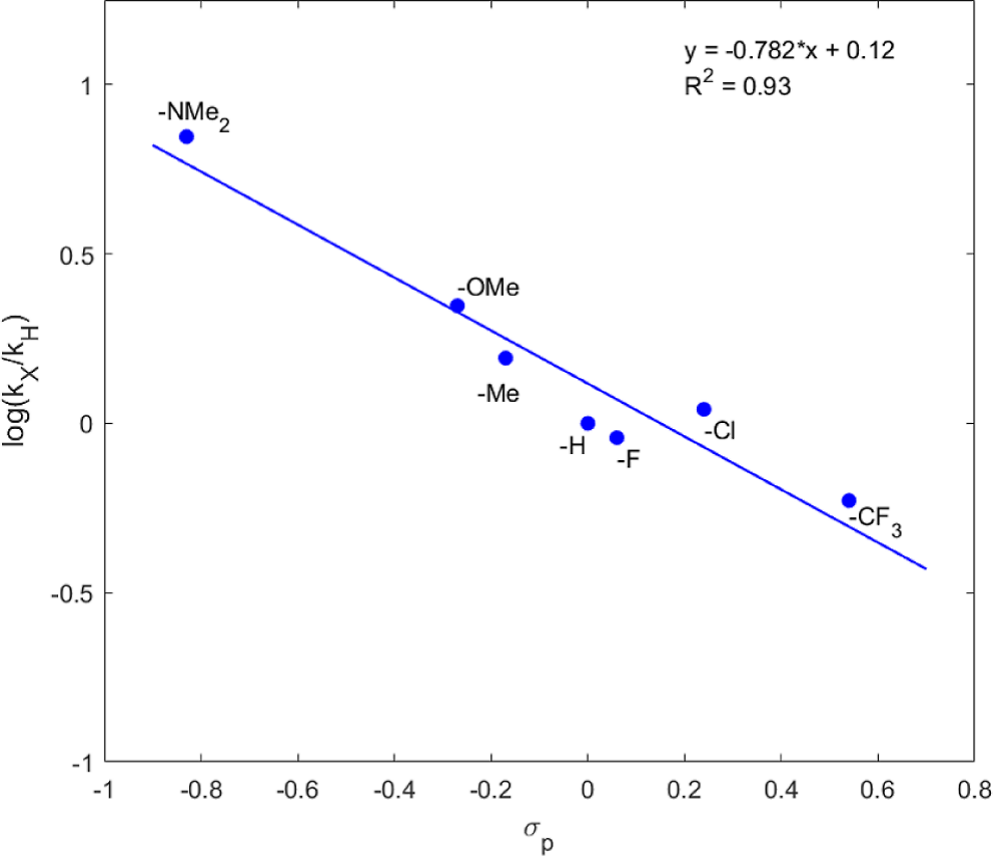
Hammett plot versus sigma parameter.

Finally, a Hammett study was performed to gain further insight into the substituent effects on the rate of YND–BME adduct fragmentation. The added asymmetry created by the four substituted phenyl rings on the ylidene created complex mixtures of eight diastereomers. However, in substrates **6o–6u**, proton integration ratios showed consistent diastereomer ratios across the substrate scope (Figure S16). Since the rate of fragmentation has proven to vary greatly for the different diastereomers, the consistency in diastereomer ratios allowed for a reasonable comparison of the observed rate of the mixture. As seen in Table 8, the para-substitution of electron-donating groups accelerated the fragmentation with the fastest rate observed for dimethylamino-substituted **6o** (*t*_1/2_ = 5.9 min), while the slowest rate of fragmentation was observed for the CF_3_-substituted substrate **6u** (*t*_1/2_ = 70 min). These results further support the theoretical result showing an increase of positive charge (0.40*e*) in the fulvene portion of the structures on going from YND–BME to the transition state (Figure 5). The larger ρ value of −0.78 derived from the Hammett plot^41^ (Figure 7) in comparison to the OND system (ρ = −0.49)^14^ further supports an asynchronous transition state structure. Furthermore, the larger ρ value is in alignment with the fact that the change in charge on the analogous furan portion of the OND system also showed an increase of 0.20*e* versus 0.40*e* for the fulvene portion of the YND.^14^ Finally, in the OND system, theoretical calculations for the retro-[4 + 2] transition state structure gave nascent bond lengths of 1.91 and 2.53 Å in the model system,^12^ while all the diastereomers of the model YND system (**6a**) have more developed transition state structures (Table 5 and Figure 5), with the most asynchronous (**6a:d2**) having nascent bond lengths of 1.96 and 2.85 Å (ωB97X-D functional).

**Table 8:**
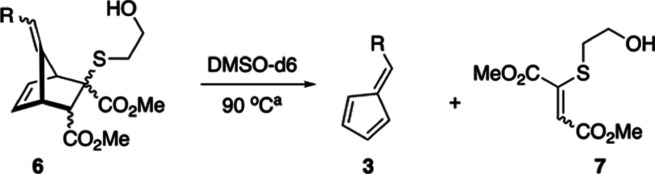

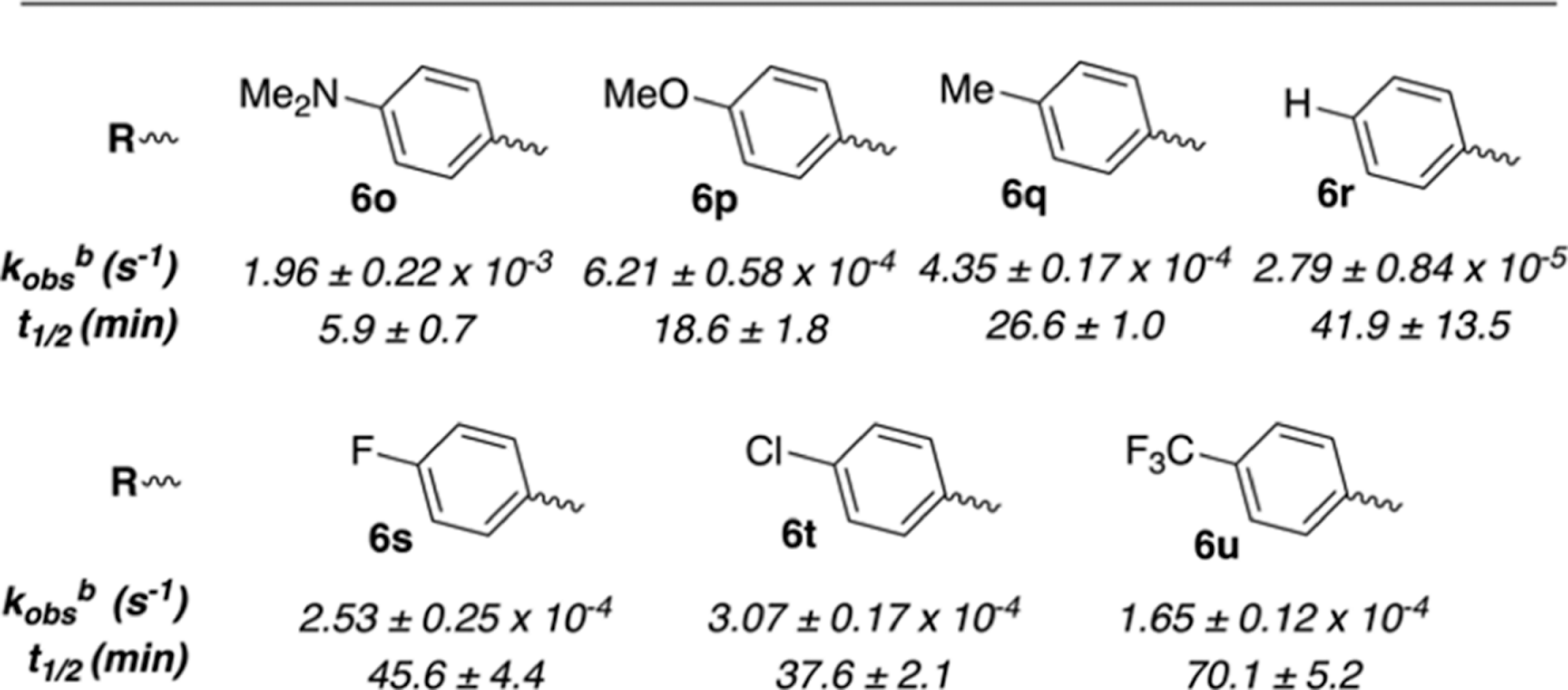
Hammett Substrate Kinetics Data

a The temperature was increased to 90 °C for the Hammett study substrates such that the study of slower-to-fragment substrates could be completed in overnight NMR experiments.

b Our previous report^23^ provided *k*_obs_ values from linear fits of the curved integrated rate data for the fragmentation of the mixture of diastereomers taken to 90% completion. Herein, we report more representative *k*_obs_ values obtained from the reaction taken to 50% completion.

## Conclusions

Thiol nucleophiles BME and propane thiol added to symmetric YND substrates yield a mixture of four diastereomers and added to asymmetric YNDs yield up to eight diastereomers of YND-thiol adducts. These YND thiol adduct diastereomers were observed to fragment via retro-[4 + 2] reactions at rates which can differ by nearly 2 orders of magnitude between the fastest and slowest diastereomers. Kinetic simulations were conducted to extrapolate rate constants and were determined to match individually measured values, thereby validating our methodology. Computational DFT explorations and a Hammett study support the view that the fragmentation reaction proceeds through an asynchronous retro-[4 + 2] transition state, with a buildup of positive charge in the fulvene portion of the molecule. The structures and functional groups which increase the charge density in the ylidene atop the YND bridge stabilize this positive charge buildup and accelerate the fragmentation reaction. Electron-withdrawing functionalities decelerate the fragmentation. This work has helped gain insight into how YNDs could be used as tunable click and clip linkages for a variety of applications.

## Data Availability

The data underlying this study are available in the published article and its Supporting Information.

## Supporting Information Available

Full experimental details, including synthetic and kinetic procedures and characterization details (PDF)
Full computational methods, energies, and Cartesian coordinates for all optimized structures and transition state structures and ^1^H and ^13^C NMR spectra for new compounds (PDF)
