# Supplementary material for: Experimental and Theoretical Exploration of the Kinetics and Thermodynamics of the Nucleophile-Induced Fragmentation of Ylidenenorbornadiene Carboxylates
Source: J Org Chem. 2023 Aug 3;88(16):11683–93. doi: 10.1021/acs.joc.3c00980 (PMC10442913; doi:10.1021/acs.joc.3c00980)
Supplement: Supplementary file 1 — jo3c00980_si_001.pdf [file jo3c00980_si_001.pdf]

Supporting Information

Experimental and Theoretical Exploration of the Kinetics and Thermodynamics of the Nucleophile-Induced Fragmentation of Ylidenenorbornadiene Carboxylates

Abigail D. Richardson,[1] Scott J. L'Heureux,[1] Ava M. Henry,[1] Elizabeth A. McDonough,[1] Cameron J. Fleischer,[1] Cameron C. McMullen,[1] Trevor R. Reynafarje,[1] Gisele P. Guerrero,[1] Quinn E. Williams,[1] Qingyang Zhou,[2] David M. Malouf,[1] Spencer E. Thurman,[1] Julia E. Soeller,[1] Jerry Y. Sheng,[1] Erica A. Medhurst,[1] Angel E. Canales,[1] Ty B. Cecil,[1] K.N. Houk,[2] Philip J. Costanzo,[1] Daniel A. Bercovici[1]*

[1]Department of Chemistry and Biochemistry, California Polytechnic State University, 1 Grand Avenue, San Luis Obispo, CA 93047, United States

[2]Department of Chemistry and Biochemistry, University of California, Los Angeles, California 90095, United States;

*email: dbercovi@calpoly.edu

PART 1 of 2

Table of Contents – Part 1 (*this document*)

1. General ***S2***

2. Synthetic Procedures ***S2***

2.1 Fulvene Synthesis **S2**

2.2 Ylidenenorbornadiene (Di)carboxylate (YND) Synthesis **S2**

2.3 Synthesis of Ylidenenorbornadiene Dicarboxylates β-mercaptoethanol (BME) or Propanethiol (PT) Adducts (YND-BME or YND-PT) via Conjugate Addition **S6**

3. Diastereomer Structural Assignment ***S11***

3.1 Confirmation of cis-7 stereochemistry **S11**

3.2 Identification of YND-PT 8a diastereomers d1-d3 .... **S17**

4. Comparison of Hammett Substrate Diastereomers ***S28***

5. Computational search of PES for lower TS energy of anti-exo diastereomer ***S29***

6. ^{1}H NMR Kinetic Studies ***S32***

7. Kinetic Simulations ***S63***

8. References – Part 1 .... ***S64***

Table of Contents – Part 2 (*other document*)

9. Computational Methods *S63*

9.1 ωB97X -D Cartesian coordinates and energies *S63*

9.2 M06-2X Cartesian coordinates and energies *S88*

9.3 B3LYP Cartesian coordinates and energies *S98*

10. References – Part 2 .... *S110*

11. ^{1}H and ^{13}C NMR Spectra for New Compounds *S111*

1. General

Reagents and solvents were purchased from commercial sources and used without further purification. Unless otherwise stated, reactions were performed under ambient conditions without attempts to exclude air or moisture (other than capping the reaction vessels). Nuclear magnetic resonance (NMR) spectra were obtained using Bruker Ultrashield 300 and Ascend 400 instrumentation. Chloroform ($CDCl_3$) and dimethyl sulfoxide (DMSO-d_6) were our primary NMR solvents used and were purchased from commercial source Cambridge-Isotope or Oakwood Chemical. All ^{1}H NMR spectra contain chemical shifts on the x-axis, reading from -0.5 to 10.5 ppm. ^{1}H NMR spectra obtained in DMSO-d_6 were referenced to the signals of the residual protium in DMSO-d_6. ^{1}H NMR spectra obtained in $CDCl_3$ were referenced to the TMS (tetramethylsilane) peak (0.00 ppm). All ^{13}C NMR unless otherwise noted are proton decoupled "^{13}C{^{1}H}" and read from -20 to 220 ppm. ^{13}C NMR spectra obtained in $CDCl_3$ were referenced to the $CDCl_3$ peak (77.2 ppm). Structural assignments were made with additional information from gCOSY, gHSQC, and gHMBC experiments. Thin-layer chromatography (TLC) was performed using manganese-doped zinc silicate aluminum-backed shortwave ultraviolet-responsive plates and were analyzed via shortwave ultraviolet light. TLC analysis often was aided by the use of potassium permanganate ($KMnO_4$) stain for visualization of reaction progress. Unless otherwise noted, all flash column chromatography was done on silica solid support. The silica used was a 5:1 (v/v) mixture of SiliaFlash P60 (40-63 µm; 230-400 mesh) to SiliaFlash GE60 (60-200 µm; 70-230 mesh) respectively. High-Resolution Mass Spectrometry (HRMS) data was acquired by Joshua Kimball at Promega Biosciences using a Sciex 5600+ TripleTOF with a Sciex ExionAD UHPLC.

2. Synthetic Procedures

2.1 Fulvene Synthesis

Unless otherwise noted, all fulvenes were synthesized using procedures from Coskun and Erden.[1] Products were confirmed via ^{1}H NMR and ^{13}C{^{1}H} NMR spectroscopy and matched previously published spectra (**3b**, **e**, **g**, **h**, **o**, **p**, **q**, **r**, **t**, **u**),[1] (**3c**),[2] (**3d**),[3] (**3f**),[4] (**3g**, **h**),[5] (**3i**),[6] (**3j**, **k**),[7] (**3s**).[8] Fulvene **3a** was purchased from Sigma Aldrich and used without further purification.

2.2 Ylidenenorbornadiene (Di)carboxylate (YND) Synthesis

Scheme S1. Preparation of YND Substrates

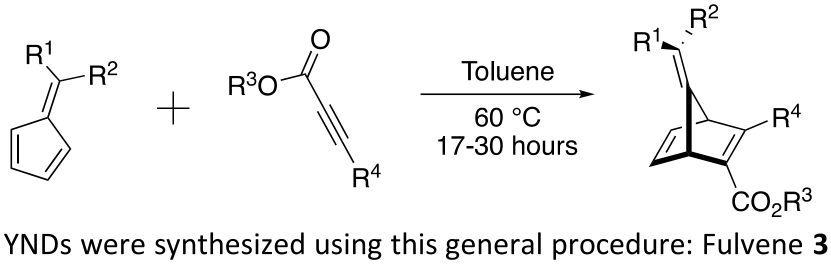

YNDs were synthesized using this general procedure: Fulvene **3** (1.2 – 7.8 mmol, 1.0 equiv.) and dimethyl acetylene dicarboxylate (DMAD) (1.3 – 9.4 mmol, 1.0 – 1.2 equiv.) were dissolved in toluene and heated at 60 °C in an oil bath, capped, and stirred overnight. Reaction progress was monitored by TLC, and upon completion the reaction mixture was removed from heat and

allowed to cool to room temperature. Solvent was removed by rotary evaporation and products were isolated by column chromatography as racemic mixtures and confirmed via ^{1}H NMR and $^{13}C\{^{1}H\}$ NMR spectroscopy. YNDs (**5a, b, c, d, f, l, m, n**)[3] were previously reported by us and (**5g, h, i**)[5] match previously reported spectra.

**5-(-2,3-bis(methoxycarbonyl)bicyclo[2.2.1]hepta-2,5-dien-7-ylidene)hexanoic acid (YND 5e).** Fulvene **3e** (5.0 mmol, 0.89 g, 1.0 eq); DMAD (5.0 mmol, 0.71 g, 1.0 equiv.). Yield: 4.9 mmol, 1.6 g, 99%, orange oil. R_f = 0.45 (1:1 Heptane:EtOAc – silica solid support). ^{1}H NMR (400 MHz, Chloroform-*d*) δ 6.99 (m, 2H), 4.41 (m, 2H), 3.79 (s, 3H), 3.79 (s, 3H), 2.25 (t, *J* = 7.4 Hz, 2H), 1.93 (t, *J* = 7.2 Hz, 2H), 1.72 – 1.63 (m, 2H), 1.48 (s, 3H). $^{13}C\{^{1}H\}$ NMR (75 MHz, $CDCl_3$) δ 179.4, 164.9, 164.7, 163.4, 151.8, 151.1, 142.1, 142.0, 102.3, 55.1, 53.1, 52.0, 52.0, 32.8, 31.3, 22.3, 15.7. HRMS (ESI-TOF) m/z:[M+H]+ Calcd for $C_{17}H_{21}O_6^+$ 321.1333; found 321.1328.	**YND 5e** 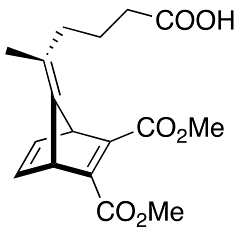
**Dimethyl-7-cycloheptylidenebicyclo[2.2.1]hepta-2,5-diene-2,3-dicarboxylate (YND 5j).** Fulvene **3j** (3.7 mmol, 0.59 g, 1.0 equiv.); DMAD (4.4 mmol, 0.63 g, 1.2 equiv.). Yield: 2.8 mmol, 0.85 g, 84%, dark orange oil. R_f = 0.3 (9:1 Heptane:EtOAc – silica solid support). ^{1}H NMR (300 MHz, Chloroform-*d*) δ 7.00 (*apparent* t, *J* = 2.1 Hz, 2H), 4.41 (*apparent* t, *J* = 2.1 Hz, 2H), 3.79 (s, 6H), 2.12 – 2.01 (m, 4H), 1.53 – 1.45 (m, 4H), 1.44 – 1.38 (m, 4H). $^{13}C\{^{1}H\}$ NMR (75 MHz, $CDCl_3$) δ 164.8, 162.4, 151.5, 141.9, 108.4, 53.0, 51.9, 29.9, 28.8, 27.3. HRMS (ESI-TOF) m/z:[M+H]+ Calcd for $C_{18}H_{23}O_4^+$ 303.1591; found 303.1589.	**YND 5j** 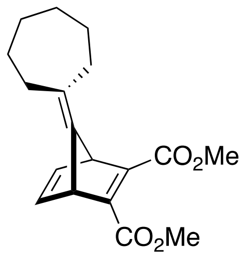
**Dimethyl-7-cyclooctylidenebicyclo[2.2.1]hepta-2,5-diene-2,3-dicarboxylate (YND 5k).** Fulvene **3k** (1.2 mmol, 0.20 g, 1.0 equiv.); DMAD (1.3 mmol, 0.18 g, 1.1 equiv.). Yield: 0.97 mmol, 0.31 g, 81%, orange oil. R_f = 0.27 (95:5 30-60 Ligroin:EtOAc – silica solid support). ^{1}H NMR (400 MHz, Chloroform-*d*) δ 7.02 (*apparent* dd, *J* = 2.4, 1.8 Hz, 2H), 4.43 (*apparent* dd, *J* = 2.4, 1.8 Hz, 2H), 3.82 (s, 6H), 2.09 – 1.91 (m, 4H), 1.56 – 1.49 (m, 4H), 1.45 – 1.37 (m, 6H). $^{13}C\{^{1}H\}$ NMR (101 MHz, $CDCl_3$) δ 165.1, 161.8, 151.5, 141.8, 108.6, 53.3, 52.1, 29.7, 26.6, 25.9, 25.5. HRMS (ESI-TOF) m/z:[M+H]+ Calcd for $C_{19}H_{25}O_4^+$ 317.1748; found 317.1748.	**YND 5k** 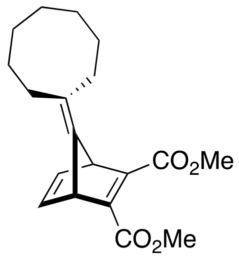

**Dimethyl-7-(4-(dimethylamino)benzylidene)bicyclo[2.2.1] hepta-2,5-diene-2,3-dicarboxylate (YND 5o).** Fulvene **3o** (4.1 mmol, 0.81 g, 1.0 equiv.); DMAD (4.9 mmol, 0.70 g, 1.2 equiv.). Yield: 3.8 mmol, 1.3 g, 93%, light yellow solid. R_f = 0.33 (5:1 30-60 Ligroin:EtOAc – silica solid support). ^{1}H NMR (400 MHz, Chloroform-*d*) δ 7.14 – 7.05 (m, 4H), 6.73 – 6.68 (m, 2H), 5.33 (s, 1H), 4.87 – 4.73 (m, 1H), 4.32 (ddd, *J* = 3.2, 2.5, 0.9 Hz, 1H), 3.84 (s, 3H), 3.83 (s, 3H), 2.96 (s, 6H). ^{13}C{^{1}H} NMR (101 MHz, $CDCl_3$) δ 165.5, 164.8, 164.7, 151.8, 151.0, 149.2, 142.4, 141.9, 128.9, 123.8, 112.5, 100.0, 57.4, 52.9, 52.2, 40.6. HRMS (ESI-TOF) m/z:[M+H]$^+$ Calcd for $C_{20}H_{22}NO_4^+$ 340.1544; found 340.1542.	**YND 5o** 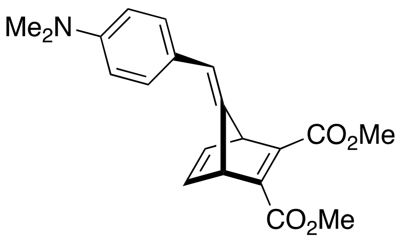
**Dimethyl-7-(4-methoxybenzylidene)bicyclo[2.2.1]hepta-2,5-diene-2,3-dicarboxylate (YND 5p).** Fulvene **3p** (2.7 mmol, 0.50 g, 1.0 equiv.); DMAD (3.2 mmol, 0.46 g, 1.2 equiv.). Yield: 2.4 mmol, 0.77 g, 87%, light yellow solid. R_f = 0.24 (15:1 Heptane:EtOAc – silica solid support). ^{1}H NMR (400 MHz, Chloroform-*d*) δ 7.12 – 7.08 (m, 3H), 7.05 (ddd, *J* = 5.2, 3.2, 0.9 Hz, 1H), 6.87 – 6.83 (m, 2H), 5.33 (s, 1H), 4.77 – 4.72 (m, 1H), 4.31 (ddd, *J* = 3.2, 2.4, 0.9 Hz, 1H), 3.82 (s, 3H), 3.81 (s, 3H), 3.80 (s, 3H). ^{13}C{^{1}H} NMR (101 MHz, $CDCl_3$) δ 165.9, 164.3, 164.2, 158.0, 151.3, 150.4, 142.0, 141.3, 128.8, 127.8, 113.6, 99.1, 56.9, 54.8, 52.4, 51.8, 51.8. HRMS (ESI-TOF) m/z:[M+H]$^+$ Calcd for $C_{19}H_{19}O_5^+$ 327.1227; found 327.1227.	**YND 5p** 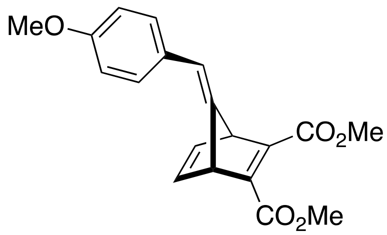
**Dimethyl-7-(4-methylbenzylidene)bicyclo[2.2.1]hepta-2,5-diene-2,3-dicarboxylate (YND 5q).** Fulvene **3q** (2.9 mmol, 0.49 g, 1.0 equiv.); DMAD (3.5 mmol, 0.50 g, 1.2 equiv.). Yield: 2.4 mmol, 0.73 g, 81%, light yellow solid. R_f = 0.33 (15:1 Heptane:EtOAc – silica solid support). ^{1}H NMR (400 MHz, Chloroform-*d*) δ 7.15 -7.01 (m, 6H), 5.36 (s, 1H), 4.76 (*apparent* t, *J* = 2.7 Hz, 1H), 4.31 (*apparent* t, *J* = 2.4 Hz, 1H), 3.82 (s, 3H), 3.80 (s, 3H), 2.32 (s, 3H). ^{13}C{^{1}H} NMR (101 MHz, $CDCl_3$) δ 166.6, 164.5, 164.4, 151.3, 150.5, 142.1, 141.5, 136.0, 132.6, 129.0, 127.8, 99.7, 57.1, 52.6, 52.1, 21.0. HRMS (ESI-TOF) m/z:[M+H]$^+$ Calcd for $C_{19}H_{19}O_4^+$ 311.1278; found 311.1275.	**YND 5q** 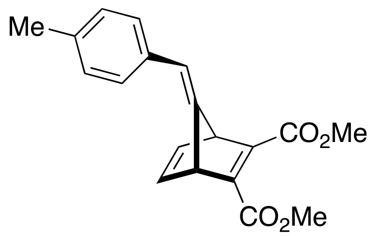

**Dimethyl-7-(benzylidene)bicyclo[2.2.1]hepta-2,5-diene-2,3-dicarboxylate (YND 5r).** Fulvene **3r** (7.8 mmol, 1.2 g, 1.0 equiv.); DMAD (9.4 mmol, 1.3 g, 1.2 equiv.). Yield: 3.1 mmol, 0.93 g, 40%, yellow oil. R_f = 0.26 (4:1 Heptane:EtOAc – silica solid support). ^{1}H NMR (400 MHz, Chloroform-*d*) δ 7.31 (m, 2H), 7.22 – 7.15 (m, 3H), 7.10 (ddd, *J* = 5.2, 3.2, 0.8 Hz, 1H), 7.05 (ddd, *J* = 5.2, 3.2, 0.9 Hz, 1H), 5.39 (s, 1H), 4.80 – 4.74 (m, 1H), 4.32 (ddd, *J* = 3.3, 2.5, 0.9 Hz, 1H), 3.82 (s, 3H), 3.81 (s, 3H). $^{13}C\{^1H\}$ NMR (101 MHz, $CDCl_3$) δ 166.9, 164.5, 164.4, 151.3, 150.5, 142.1, 141.5, 135.5, 128.3, 127.9, 126.4, 99.9, 57.0, 52.6, 52.1. HRMS (ESI-TOF) m/z:$[M+H]^+$ Calcd for $C_{18}H_{17}O_4^+$ 297.1122; found 297.1123	**YND 5r** 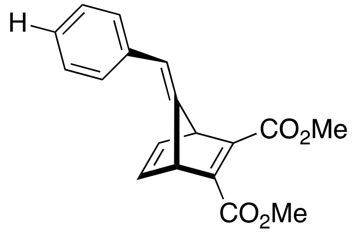
**Dimethyl-7-(4-fluorobenzylidene)bicyclo[2.2.1]hepta-2,5-diene-2,3-dicarboxylate (YND 5s).** Fulvene **3s** (5.8 mmol, 1.0 g, 1.0 equiv.); DMAD (7.0 mmol, 0.99 g, 1.2 equiv.). Yield: 4.0 mmol, 1.3 g, 69%, orange oil. R_f = 0.33 (9:1 30-60 Ligroin:EtOAc – silica solid support). ^{1}H NMR (300 MHz, Chloroform-*d*) δ 7.16-7.08 (m, 3H), 7.07- 6.95 (m, 3H), 5.35 (s, 1H), 4.71 (*apparent* dt, *J* = 2.4, 0.8 Hz, 1H), 4.31 (ddd, *J* = 3.3, 2.4, 1.0 Hz, 1H), 3.83 (s, 3H), 3.81 (s, 3H). $^{13}C\{^1H\}$ NMR (75 MHz, $CDCl_3$) δ 166.8 (d, *J* = 1,7 Hz), 164.5, 161.3 (d, *J* = 245.7 Hz), 159.8, 151.4, 150.4, 142.2, 141.5, 131.5 (d, *J* = 3.3 Hz), 129.4 (d, *J* = 8.0 Hz), 115.2 (d, *J* = 21.5 Hz), 98.9, 57.0, 52.5, 52.2. HRMS (ESI-TOF) m/z:$[M+H]^+$ Calcd for $C_{18}H_{16}FO_4^+$ 315.1027; found 315.1030.	**YND 5s** 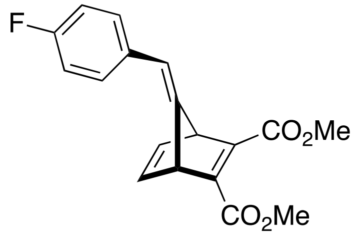
**Dimethyl-7-(4-chlorobenzylidene)bicyclo[2.2.1]hepta-2,5-diene-2,3-dicarboxylate (YND 5t).** Fulvene **3t** (5.0 mmol, 0.94 g, 1.0 equiv.); DMAD (6.0 mmol, 0.85 g, 1.2 equiv.). Yield: 3.3 mmol, 1.1 g, 66%, light yellow solid. R_f = 0.32 (4:1 Heptane:EtOAc – silica solid support). ^{1}H NMR (400 MHz, Chloroform-*d*) δ 7.29 – 7.25 (m, 2H), 7.12 – 7.07 (m, 3H), 7.04 (ddd, *J* = 5.2, 3.3, 0.9 Hz, 1H), 5.34 (s, 1H), 4.72 – 4.69 (m, 1H), 4.34 – 4.28 (m, 1H), 3.82 (s, 3H), 3.81 (s, 3H). $^{13}C\{^1H\}$ NMR (101 MHz, $CDCl_3$) δ 167.4, 164.6, 164.5, 151.4, 150.4, 142.3, 141.6, 134.2, 132.3, 129.4, 128.7, 99.1, 57.2, 52.6, 52.4, 52.4. HRMS (ESI-TOF) m/z:$[M+H]^+$ Calcd for $C_{18}H_{16}ClO_4^+$ 331.0732; found 331.0735.	**YND 5t** 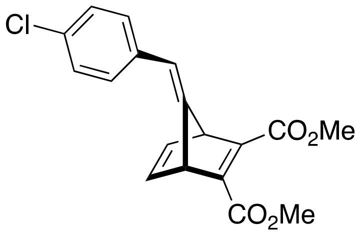

**Dimethyl-7-(4-(trifluoromethyl)benzylidene)bicyclo[2.2.1] hepta-2,5-diene-2,3-dicarboxylate (YND 5u).** Fulvene **3u** (2.7 mmol, 0.60 g, 1.0 equiv.); DMAD (3.3 mmol, 0.46 g, 1.2 equiv.). Yield: 1.3 mmol, 0.46 g, 47%, light yellow solid. R_f = 0.15 (6:1 Heptane:EtOAc – silica solid support). ^{1}H NMR (300 MHz, Chloroform-*d*) δ 7.56 (*apparent* d, *J* = 8.1 Hz, 2H), 7.32 – 7.20 (m, 2H), 7.12 (ddd, *J* = 5.3, 3.2, 0.9 Hz, 1H), 7.06 (ddd, *J* = 5.3, 3.2, 1.0 Hz, 1H), 5.42 (s, 1H), 4.73 (*apparent* td, *J* = 2.4, 0.8 Hz, 1H), 4.34 (ddd, *J* = 3.2, 2.4, 0.9 Hz, 1H), 3.83 (s, 3H), 3.82 (s, 3H). ^{13}C{^{1}H} NMR (75 MHz, $CDCl_3$) δ 168.3, 164.4, 164.3, 151.1, 150.1, 142.1, 141.3, 139.5, 128.3 (q, *J* = 32.3 Hz), 128.2, 125.3 (q, *J* = 3.8 Hz), 124.22 (q, *J* = 271.1 Hz), 98.9, 57.0, 52.5, 52.2, 52.2. HRMS (ESI-TOF) m/z:$[M+H]^+$ Calcd for $C_{19}H_{16}F_3O_4^+$ 365.0995; found 365.0995.	**YND 5u** 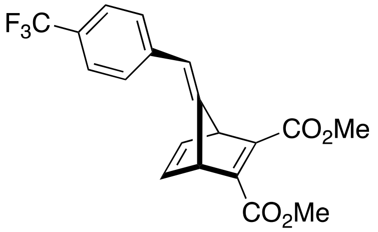

2.3 Synthesis of Ylidenenorbornadiene Dicarboxylates β-mercaptoethanol (BME) or Propanethiol (PT) Adducts (YND-BME or YND-PT) via Conjugate Addition

Scheme S2. Synthesis of YND-BME and YND-PT

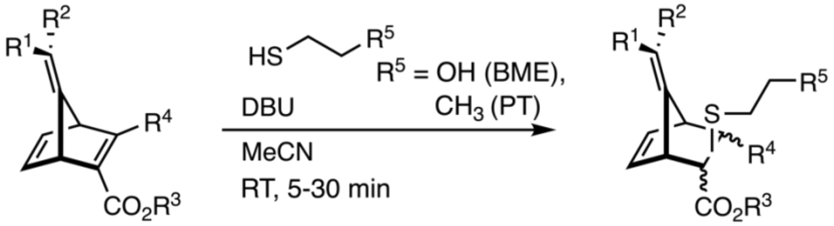

YND-BME and YND-PT substrates were synthesized using this general procedure: YND **5** (0.42 – 3.1 mmol, 1.0 equiv.) and excess thiol nucleophile, β-mercaptoethanol (BME) or propanethiol (PT) (0.53 – 3.1 mmol, 1.0 – 1.5 equiv.), were dissolved in acetonitrile. 1,8-Diazabicyclo[5.4.0]-undec-7-ene (DBU) (0.04 – 3.4 mmol, 0.1 equiv. – **6e** required 1.1 equiv.) was added and the reaction was capped, stirred, and monitored via TLC. Reactions were typically complete within 5-15 minutes. The reaction was quenched with glacial acetic acid (5 μL). Solvent was removed by rotary evaporation and products were isolated by column chromatography and confirmed via ^{1}H NMR spectroscopy. YND-BMEs (**6a, b, c, d, f, l, m, n**)[3] have been previously reported by us.

In the cases of YND-BME or YND-PT substrates with symmetric ylidene bridge substituents (**6a** and **6g-n**) an inseparable mixture of 4 diastereomers was obtained. In the cases of YND-BMEs with asymmetric ylidene bridge substituents (**6b-f** and **6o-u**) an inseparable mixture of up to 8 diastereomers was obtained. Spectra for these diastereomeric mixtures are complex with many overlapping signals. We have done our best to list peaks arising from the most prevalent

diastereomer based on signal height. In many cases however, many signals overlap requiring us to label the peak as a large multiplet. The ^{1}H NMR spectra for these mixtures are attached at the end of part 2 of the Supporting Information.

**5-(5-((2-hydroxyethyl)thio)-5,6-bis(methoxycarbonyl) bicyclo[2.2.1]hept-2-en-7-ylidene)hexanoic acid (YND-BME 6e).** YND **5e** (3.1 mmol, 1.0 g, 1.0 equiv.); BME (3.7 mmol, 0.29 g, 1.2 equiv.); DBU (3.4 mmol, 0.52 g, 1.1 equiv.). Yield: 1.6 mmol, 0.65 g, 53%, light yellow oil. R_f = 0.44 (1:1 Heptane:EtOAc – silica solid support). ^{1}H NMR (400 MHz, Chloroform-*d)* δ 6.59 (*apparent* dd, *J* = 5.6, 2.8 Hz, 1H), 6.27 (ddd, *J* = 5.9, 3.2, 1.0 Hz, 1H), 3.69 (s, 3H), 3.67 (s, 3H), 4.02 – 3.51 (m, 4H), 3.26 (d, *J* = 3.1 Hz, 1H), 3.06 – 2.67 (m, 2H), 2.57 – 2.12 (m, 2H), 2.12 – 1.75 (m, 4H), 1.62 (s, 3H). HRMS (ESI-TOF) m/z:[M+H]$^+$ Calcd for $C_{19}H_{27}O_7S^+$ 399.1472; found 399.1471.	**YND-BME 6e** 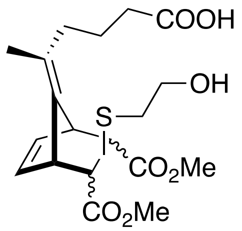
**Dimethyl-7-cyclobutylidene-2-((2-hydroxyethyl)thio)bicyclo [2.2.1]hept-5-ene-2,3-dicarboxylate (YND-BME 6g).** YND **5g** (1.0 mmol, 300 mg, 1.0 eq); BME (1.5 mmol, 120 mg, 1.5 equiv.); DBU (0.1 mmol, 15 mg, 0.1 equiv.). Yield: 0.73 mmol, 250 mg, 73%, light yellow oil. R_f = 0.31 (2:1 Et_2O:30-60 Ligroin – silica solid support). ^{1}H NMR (400 MHz, Chloroform-*d)* δ 6.58 (ddd, *J* = 5.7, 2.9, 0.9 Hz, 1H), 6.26 (ddd, *J* = 5.7, 3.1, 0.9 Hz, 1H), 3.91 – 3.70 (m, 3H), 3.69 (s, 3H), 3.66 (s, 3H), 3.49 – 3.31 (m, 2H), 3.15 (d, *J* = 3.3 Hz, 1H), 3.11 – 2.78 (m, 2H), 2.76 – 2.37 (m, 4H), 2.06 – 1.80 (m, 2H). HRMS (ESI-TOF) m/z:[M+H]$^+$ Calcd for $C_{17}H_{23}O_5S^+$ 339.1261; found 339.1256.	**YND-BME 6g** 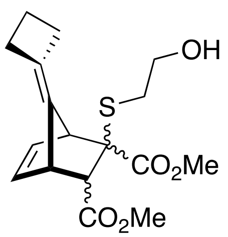
**Dimethyl-7-cyclopentylidene-2-((2-hydroxyethyl)thio)bicyclo [2.2.1]hept-5-ene-2,3-dicarboxylate (YND-BME 6h).** YND **5h** (1.1 mmol, 300 mg, 1.0 equiv.); BME (1.1 mmol, 85 mg, 1.0 equiv.); DBU (0.11 mmol, 17 mg, 0.1 equiv.). Yield: 0.23 mmol, 81 mg, 73%, light yellow oil. R_f = 0.37 (2:1 Et_2O:30-60 Ligroin – silica solid support). ^{1}H NMR (400 MHz, Chloroform-*d)* δ 6.59 (ddd, *J* = 5.9, 3.1, 0.9 Hz, 1H), 6.28 (ddd, *J* = 5.9, 2.9, 1.0 Hz, 1H), 3.90 – 3.60 (m, 2H), 3.69 (s, 3H), 3.66 (s, 3H), 3.56 – 3.40 (m, 2H), 3.16 (d, *J* = 3.1 Hz, 1H), 3.07 – 2.78 (m, 2H), 2.48 – 1.95 (m, 4H), 1.74 – 1.51 (m, 4H). HRMS (ESI-TOF) m/z:[M+H]$^+$ Calcd for $C_{18}H_{25}O_5S^+$ 353.1417; found 353.1416.	**YND-BME 6h** 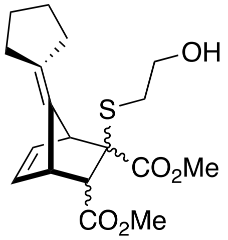

**Dimethyl-7-cyclohexylidene-2-((2-hydroxyethyl)thio)bicyclo [2.2.1]hept-5-ene-2,3-dicarboxylate (YND-BME 6i).** YND **5i** (1.6 mmol, 450 mg, 1.0 equiv.); BME (1.9 mmol, 150 mg, 1.2 equiv.); DBU (0.16 mmol, 24 mg, 0.1 equiv.). Yield: 0.98 mmol, 360 mg, 61%, light yellow oil. R_f = 0.29 (3:1 Et_2O:30-60 Ligroin – silica solid support). ^1H NMR (300 MHz, Chloroform-*d)* δ 6.58 (ddd, *J* = 5.9, 3.2, 0.9 Hz, 1H), 6.29 (ddd, *J* = 5.9, 3.0, 1.0 Hz, 1H), 4.04 – 3.71 (m, 2H), 3.69 (s, 3H), 3.67 (s, 3H), 3.65 – 3.57 (m, 2H), 3.12 (d, *J* = 3.1 Hz, 1H), 3.09 – 2.75 (m, 2H), 2.25 – 2.09 (m, 2H), 2.04 – 1.82 (m, 2H), 1.66 (s, 1H), 1.62 – 1.36 (m, 6H). HRMS (ESI-TOF) m/z:$[M+H]^+$ Calcd for $C_{19}H_{27}O_5S^+$ 367.1574; found 367.1573.	**YND-BME 6i** 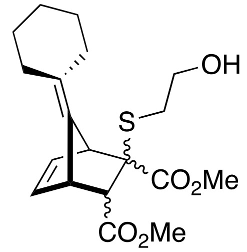
**Dimethyl-7-cycloheptylidene-2-((2-hydroxyethyl)thio)bicyclo [2.2.1]hept-5-ene-2,3-dicarboxylate (YND-BME 6j).** YND **5j** (0.91 mmol, 350 mg, 1.0 equiv.); BME (1.1 mmol, 89 mg, 1.25 equiv.); DBU (0.09 mmol, 14 mg, 0.1 equiv.). Yield: 0.73 mmol, 280 mg, 80%, light yellow oil. R_f = 0.3 (2:1 Et_2O:30-60 Ligroin – silica solid support). ^1H NMR (400 MHz, Chloroform-*d)* δ 6.58 (ddd, *J* = 5.9, 3.2, 0.9 Hz, 1H), 6.29 (ddd, *J* = 5.9, 3.1, 1.0 Hz, 1H), 4.00 – 3.71 (m, 3H), 3.69 (s, 3H), 3.67 (s, 3H), 3.61 – 3.54 (m, 2H), 3.15 (d, *J* = 2.9 Hz, 1H), 3.09 – 2.78 (m, 2H), 2.40 – 1.86 (m, 5H), 1.72 – 1.61 (m, 1H) 1.53 – 1.41 (m, 6H). HRMS (ESI-TOF) m/z:$[M+H]^+$ Calcd for $C_{20}H_{29}O_5S^+$ 381.1730; found 381.1728.	**YND-BME 6j** 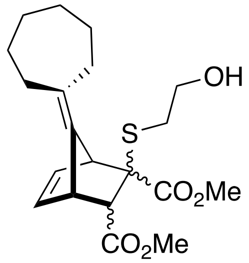
**Dimethyl-7-cyclooctylidene-2-((2-hydroxyethyl)thio)bicyclo [2.2.1]hept-5-ene-2,3-dicarboxylate (YND-BME 6k).** YND **5k** (0.77 mmol, 240 mg, 1.0 equiv.); BME (0.84 mmol, 66 mg, 1.1 equiv.); DBU (0.08 mmol, 12 mg, 0.1 equiv.). Yield: 0.36 mmol, 140 mg, 47%, dark amber oil. R_f = 0.33 (7:2 Et_2O:30-60 Ligroin – silica solid support). ^1H NMR (400 MHz, Chloroform-*d)* δ 6.60 (ddd, *J* = 5.7, 3.0, 0.9 Hz, 1H), 6.28 (ddd, *J* = 5.9, 3.2, 0.,9 Hz, 1H), 4.03 – 3.71 (m, 3H), 3.69 (s, 3H), 3.67 (s, 3H), 3.62 – 3.53 (m, 2H), 3.18 (d, *J* = 3.2 Hz, 1H), 3.10 – 2.78 (m, 2H), 2.25 – 1.62 (m, 6H), 1.54 – 1.21 (m, 8H). HRMS (ESI-TOF) m/z:$[M+H]^+$ Calcd for $C_{21}H_{31}O_5S^+$ 395.1887; found 395.1884.	**YND-BME 6k** 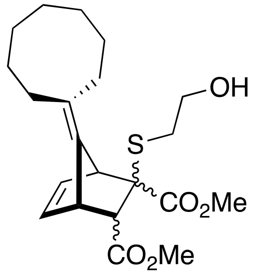

**Dimethyl-7-(4-(dimethylamino)benzylidene)-2-((2-hydroxyethyl)thio)bicyclo[2.2.1]hept-5-ene-2,3-dicarboxylate (YND-BME 6o)** YND **5o** (0.59 mmol, 200 mg, 1.0 equiv.); BME (0.74 mmol, 57 mg, 1.25 equiv.); DBU (0.06 mmol, 9.0 mg, 0.1 equiv.). Yield: 0.46 mmol, 190 mg, 78%, dark amber oil. R_f = 0.43 (9:1 Et_2O:30-60 Ligroin – silica solid support). ^1H NMR (400 MHz, Chloroform-*d)* *δ* 7.45 (d, *J* = 8.9 Hz, 2H), 6.81 – 6.24 (m, 4H), 5.83 (s, 1H), 3.70 (s, 3H), 3.69 (s, 3H), 4.43 – 3.21 (m, 5H), 2.94 (s, 6H), 3.13 – 2.73 (m, 2H). HRMS (ESI-TOF) m/z:$[M+H]^+$ Calcd for $C_{22}H_{28}NO_5S^+$ 418.1683; found 418.1677.	**YND-BME 6o** 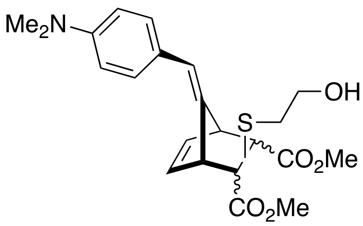
**Dimethyl-2-((2-hydroxyethyl)thio)-7-(4-methoxybenzylidene) bicyclo[2.2.1]hept-5-ene-2,3-dicarboxylate (YND-BME 6p)** YND **5p** (0.5 mmol, 160 mg, 1.0 equiv.); BME (0.55 mmol, 43 mg, 1.1 equiv.); DBU (0.05 mmol, 7.6 mg, 0.1 equiv.). Yield: 0.45 mmol, 180 mg, 91%, yellow-orange oil. R_f = 0.33 (6:1 Et_2O:30-60 Ligroin – silica solid support). ^1H NMR (300 MHz, Chloroform-*d)* *δ* 7.59 – 7.27 (m, 1H), 7.22 – 6.81 (m, 3H), 6.67 (ddd, *J* = 8.3, 5.7, 3.0 Hz, 1H), 6.34 (ddd, 1H), 5.87 (s, 1H), 3.99 – 3.93 (m, 1H), 3.80 (s, 3H), 3.78 – 3.72 (m, 1H), 3.71 (s, 3H), 3.70 (s, 3H), 3.51 – 3.42 (m, 2H), 3.25 (d, *J* = 3.2 Hz, 1H), 3.14 – 2.56 (m, 2H). HRMS (ESI-TOF) m/z:$[M+H]^+$ Calcd for $C_{21}H_{25}O_6S^+$ 405.1367; found 405.1364.	**YND-BME 6p** 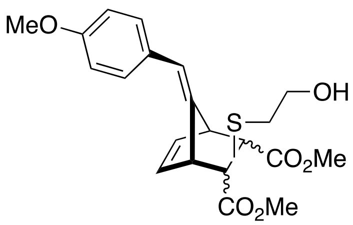
**Dimethyl-2-((2-hydroxyethyl)thio)-7-(4-methylbenzylidene) bicyclo[2.2.1]hept-5-ene-2,3-dicarboxylate (YND-BME 6q)** YND **5q** (2.8 mmol, 880 mg, 1.0 equiv.); BME (2.8 mmol, 220 mg, 1.0 equiv.); DBU (0.28 mmol, 43 mg, 0.1 equiv.). Yield: 2.5 mmol, 960 mg, 88%, light yellow oil. R_f = 0.35 (5:1 Et_2O:30-60 Ligroin – silica solid support). ^1H NMR (400 MHz, Chloroform-*d)* *δ* 7.26 – 7.00 (m, 4H), 6.66 (ddd, 1H), 6.34 (ddd, *J* = 5.9, 3.1, 0.8 Hz, 1H), 5.90 (s, 1H), 4.02 – 3.95 (m, 1H), 3.77 – 7.72 (m, 1H), 3.71 (s, 3H), 3.70 (s, 3H), 3.48 – 3.41 (m, 2H), 3.25 (d, *J* = 3.2 Hz, 1H), 3.13 – 2.70 (m, 2H), 2.33 (s, 3H). HRMS (ESI-TOF) m/z:$[M+H]^+$ Calcd for $C_{21}H_{25}O_5S^+$ 389.1417; found 389.1412.	**YND-BME 6q** 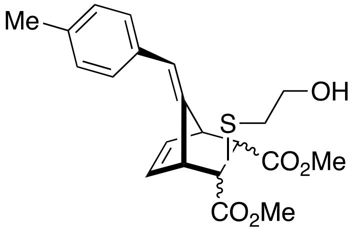

**Dimethyl-7-(benzylidene)-2-((2-hydroxyethyl)thio)bicyclo [2.2.1]hept-5-ene-2,3-dicarboxylate (YND-BME 6r)** YND **5r** (1.0 mmol, 300 mg, 1.0 equiv.); BME (1.25 mmol, 97 mg, 1.25 equiv.); DBU (0.1 mmol, 15 mg, 0.1 equiv.). Yield: 0.4 mmol, 150 mg, 40%, light yellow oil. R_f = 0.3 (4:1 Et_2O:30-60 Ligroin. – silica solid support). ^{1}H NMR (400 MHz, Chloroform-*d) δ* 7.39 – 7.27 (m, 3H), 7.25 – 7.14 (m, 2H), 6.67 (ddd, 1H), 6.35 (ddd, *J* = 5.9, 3.2, 0.9 Hz, 1H), 5.94 (s, 1H), 4.01 – 3.96 (m, 1H), 3.78 – 3.72 (m, 1H), 3.71 (s, 3H), 3.70 (s, 3H), 3.49 – 3.45 (m, 2H), 3.27 (d, *J* = 3.4 Hz, 1H), 3.13 – 2.71 (m, 2H). HRMS (ESI-TOF) m/z:[M+H]$^+$ Calcd for $C_{20}H_{23}O_5S^+$ 375.1261; found 375.1263.	**YND-BME 6r** 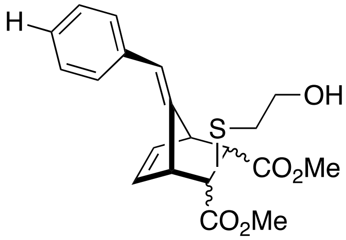
**Dimethyl-7-(4-fluorobenzylidene)-2-((2-hydroxyethyl)thio) bicyclo[2.2.1]hept-5-ene-2,3-dicarboxylate (YND-BME 6s)** YND **5s** (1.0 mmol, 310 mg, 1.0 equiv.); BME (1.4 mmol, 110 mg, 1.4 equiv.); DBU (0.1 mmol, 15 mg, 0.1 equiv.). Yield: 0.6 mmol, 260 mg, 66%, light yellow oil. R_f = 0.31 (6:1 Et_2O:30-60 Ligroin – silica solid support). ^{1}H NMR (400 MHz, Chloroform-*d) δ* 7.52 – 7.27 (m, 1H), 7.22 – 6.93 (m, 3H), 6.65 (ddd, *J* = 5.9, 3.4, 1.1 Hz, 1H), 6.36 (ddd, *J* = 5.9, 3.1, 1.0 Hz, 1H), 5.92 (s, 1H), 3.93 – 3.87 (m, 1H), 3.78 – 3.71 (m, 1H), 3.70 (s, 3H), 3.69 (s, 3H), 3.47 – 3.42 (m, 2H), 3.30 (d, *J* = 3.2 Hz, 1H), 3.10 – 2.73 (m, 2H). HRMS (ESI-TOF) m/z:[M+H]$^+$ Calcd for $C_{20}H_{22}FO_5S^+$ 393.1167; found 393.1169.	**YND-BME 6s** 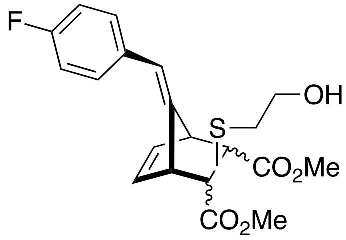
**Dimethyl-7-(4-chlorobenzylidene)-2-((2-hydroxyethyl)thio) bicyclo[2.2.1]hept-5-ene-2,3-dicarboxylate (YND-BME 6t)** YND **5t** (0.42 mmol, 140 mg, 1.0 equiv.); BME (0.53 mmol, 41 mg, 1.25 equiv.); DBU (0.04 mmol, 6.4 mg, 0.1 equiv.). Yield: 0.42 mmol, 170 mg, 99%, light yellow oil. R_f = 0.44 (4:1 Et_2O:30-60 Ligroin – silica solid support). ^{1}H NMR (400 MHz, Chloroform-*d) δ* 7.52 – 7.27 (m, 2H), 7.25 – 6.97 (m, 2H), 6.65 (ddd, *J* = 5.9, 2.9, 0.6 Hz, 1H), 6.35 (ddd, *J* = 5.9, 3.2, 0.9 Hz, 1H), 5.93 (s, 1H), 3.93 – 3.89 (m, 1H), 3.76 – 3.72 (m, 1H), 3.72 (s, 3H), 3.70 (s, 3H), 3.48 – 3.44 (m, 2H), 3.24 (d, *J* = 3.2 Hz, 1H), 2.94 – 2.92 (m, 2H). HRMS (ESI-TOF) m/z:[M+H]$^+$ Calcd for $C_{20}H_{22}ClO_5S^+$ 409.0871; found 409.0866.	**YND-BME 6t** 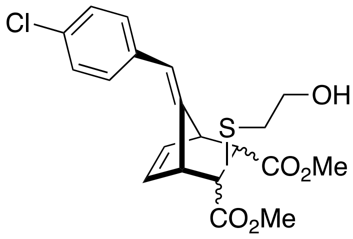

**Dimethyl-2-((2-hydroxyethyl)thio)-7-(4-(trifluoromethyl) benzylidene)bicyclo[2.2.1]hept-5-ene-2,3-dicarboxylate (YND-BME 6u)** YND **5u** (0.64 mmol, 230 mg, 1.0 equiv.); BME (0.77 mmol, 60 mg, 1.2 equiv.); DBU (0.06 mmol, 9.7 mg, 0.1 equiv.). Yield: 0.53 mmol, 240 mg, 83%, light yellow oil. R_f = 0.38 (4:1 Et_2O:30-60 Ligroin – silica solid support). ^1H NMR (400 MHz, Chloroform-*d)* δ 7.73 – 7.29 (m, 4H), 6.68 (ddd, 1H), 6.38 (ddd, 1H), 6.01 (s, 1H), 4.04 – 3.58 (m, 2H), 3.72 (s, 3H), 3.69 (s, 3H), 3.56 – 3.39 (m, 2H), 3.25 (d, *J* = 3.2 Hz, 1H), 3.14 – 2.60 (m, 2H). HRMS (ESI-TOF) m/z:$[M+H]^+$ Calcd for $C_{21}H_{22}F_3O_5S^+$ 443.1135; found 443.1133.	**YND-BME 6u** 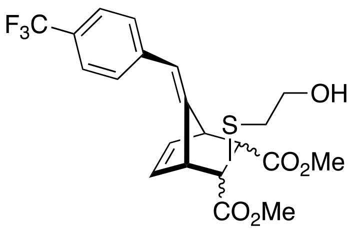
**Dimethyl-7-(propan-2-ylidene)-2-(propylthio)bicyclo[2.2.1] hept-5-ene-2,3-dicarboxylate (YND-PT 8a)** YND **5a** (2.0 mmol, 500 mg, 1.0 equiv.); propanethiol (2.4 mmol, 180 mg, 1.2 equiv.); DBU (0.2 mmol, 30 mg, 0.1 equiv.). Yield: 1.5 mmol, 470 mg, 73%, light yellow oil. R_f = 0.31 (9:1 30-60 Ligroin: Et_2O – silica solid support). ^1H NMR (400 MHz, Chloroform-*d)* δ 6.57 (ddd, *J* = 5.7, 3.0, 0.8 Hz, 1H), 6.22 (ddd, *J* = 5.8, 3.0, 1.0 Hz, 1H), 3.98 (ddd, *J* = 3.2, 1.7, 0.9 Hz, 1H), 3.74 (s, 3H), 3.68 (s, 3H), 3.57 (s, 1H), 3.24 – 3.05 (m, 1H), 2.78 – 2.55 (m, 2H), 1.67 (s, 3H), 1.63 (s, 3H), 1.53 – 1.52 (m, 2H), 1.06 – 0.84 (m, 3H). HRMS (ESI-TOF) m/z:$[M+H]^+$ Calcd for $C_{17}H_{24}O_4S^+$ 325.1468; found 325.1467.	**YND-PT 8a** 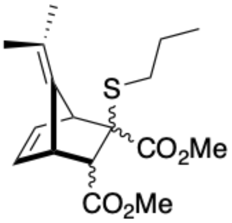

3. Diastereomer Structural Assignment

3.1 Confirmation of cis-7 stereochemistry

YND-BME **6a** (73 mg, 0.2 mmol) was dissolved in 1.5 mL of acetonitrile and reacted at 80 °C until more than 90% of **6a** fragmented to form **3a**, ***cis*-7**, and ***trans*-7** (**Scheme S3**). The reaction was heated further until a ratio of approximately 1:9 of the ***trans*:*cis*** alkene **7** stereoisomers was achieved, determined by ^1H NMR (**Figure S1**). Silica gel column chromatography with 2:1 ethyl acetate:30-60 ligroin mixture was performed to yield the diastereomeric mixture of ***cis*-7** and ***trans*-7** in the same ratio of approximately 1:9 as the isolated products. 2D NMR spectra (NOESY - **Figure S3**, HSQC - **Figure S4** and HMBC - **Figure S5**) were obtained to determine stereochemistry. NOESY correlations between the alkene proton and both sets of methylene protons on the BME chain indicate ***cis-7*** as the major product as shown in **Figure S3**.

Scheme S3. Fragmentation of YND-BME 6a and Subsequent Isomerization of Alkene Products

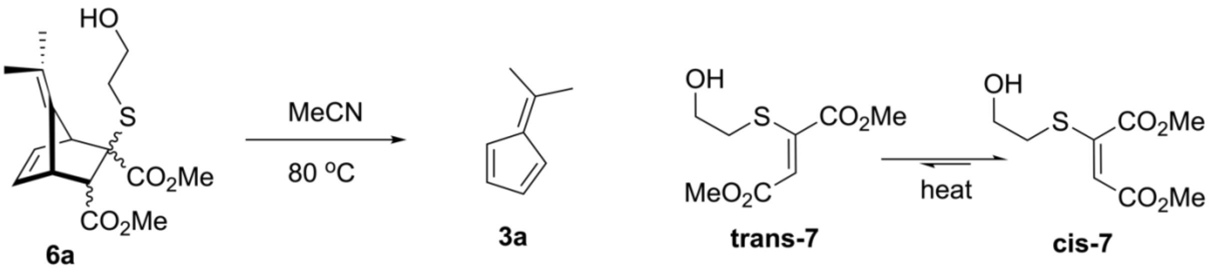

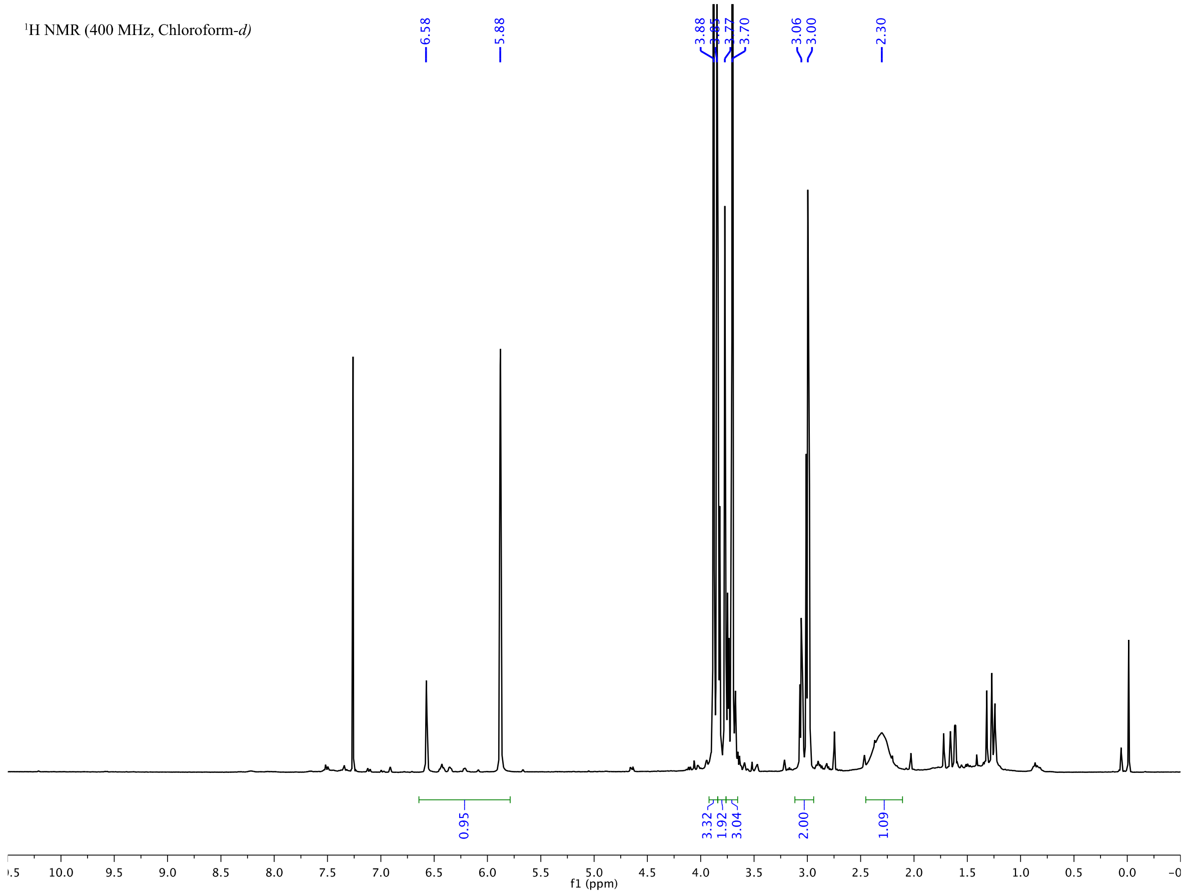

Figure S1. ^{1}H NMR of ~9:1 **cis-7:trans-7** fragmentation product.

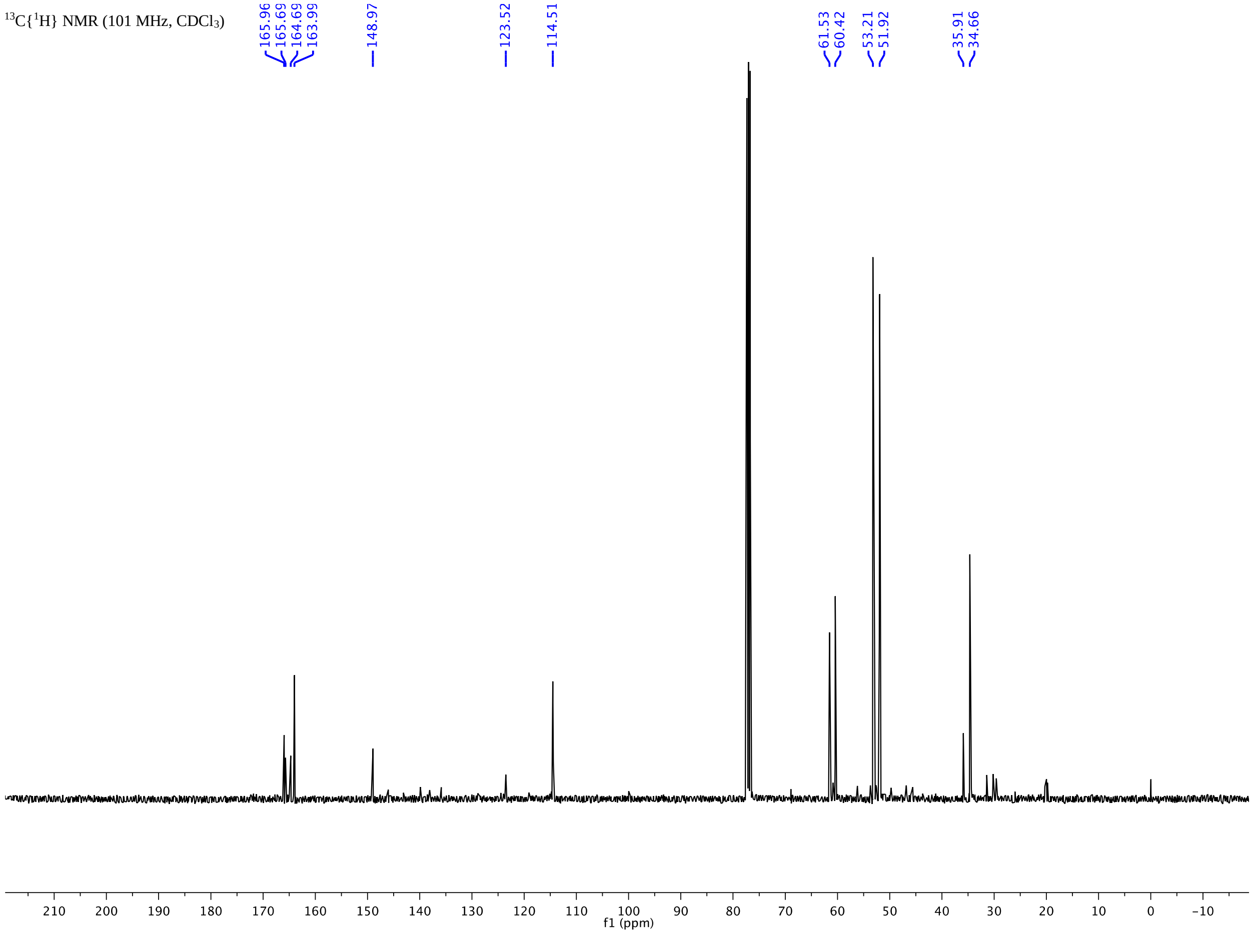

Figure S2. ^{13}C NMR of ~9:1 **cis-7:trans-7** fragmentation product.

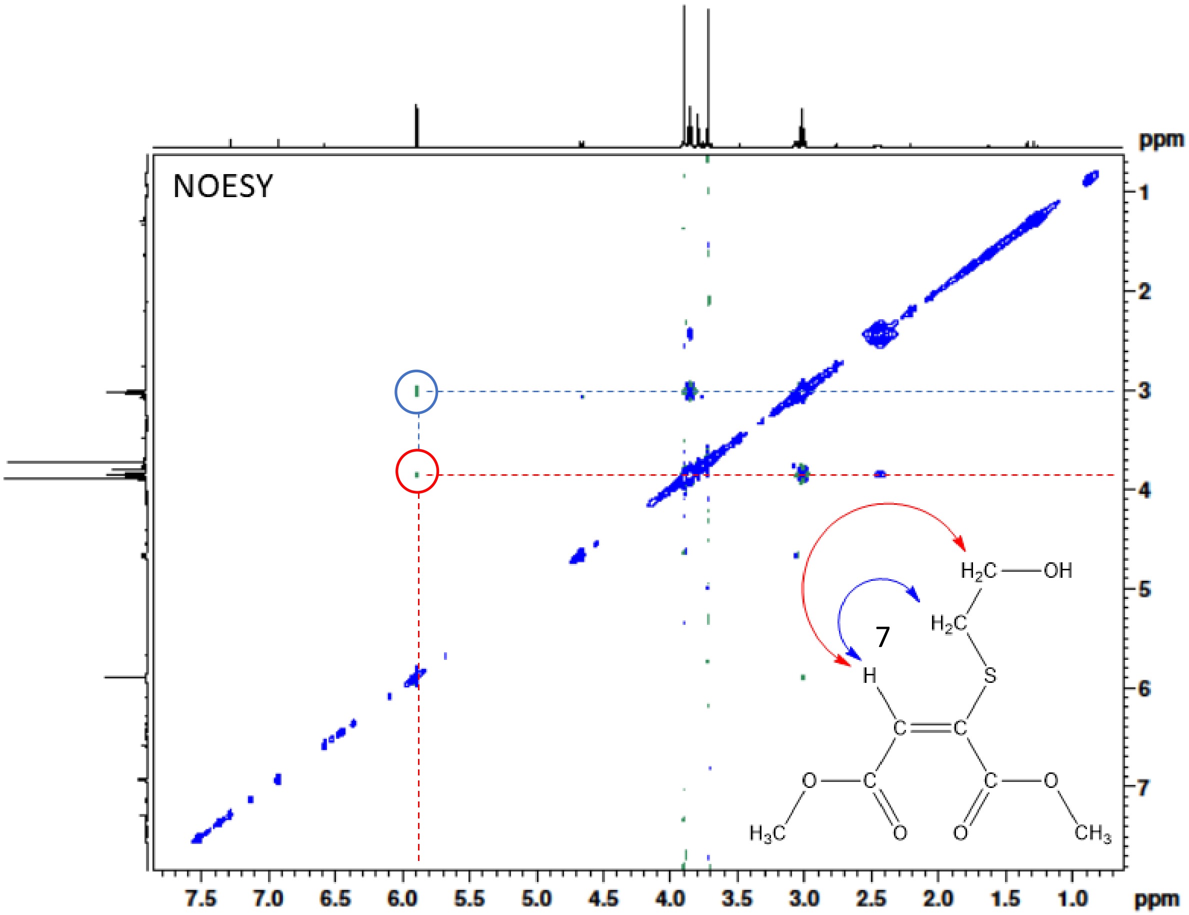

Figure S3. NOESY spectrum of the **6a** fragmentation alkene product, **7**. Correlations between the alkene proton and -CH_2 protons alpha to sulfur and oxygen on the BME chain indicate the **cis-7** isomer as the major product.

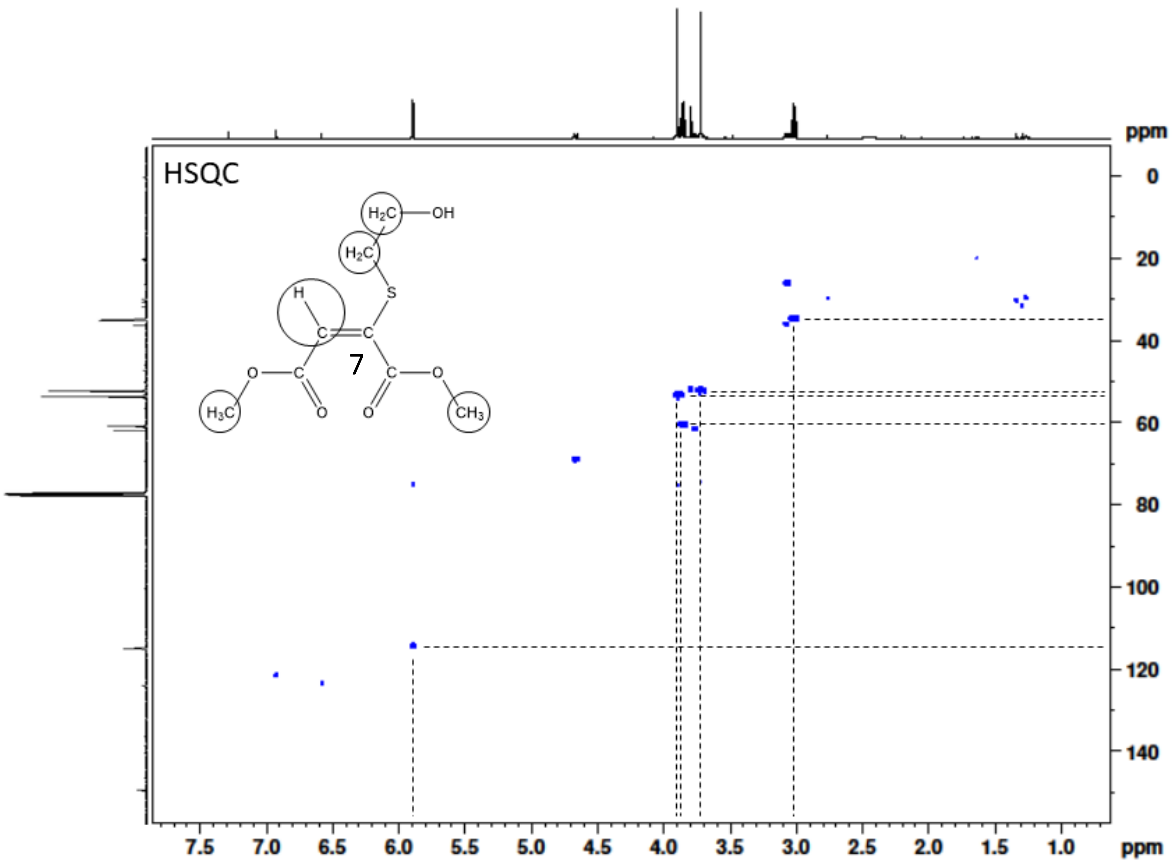

Figure S4. HSQC spectrum of the **6a** fragmentation alkene product **cis-7**.

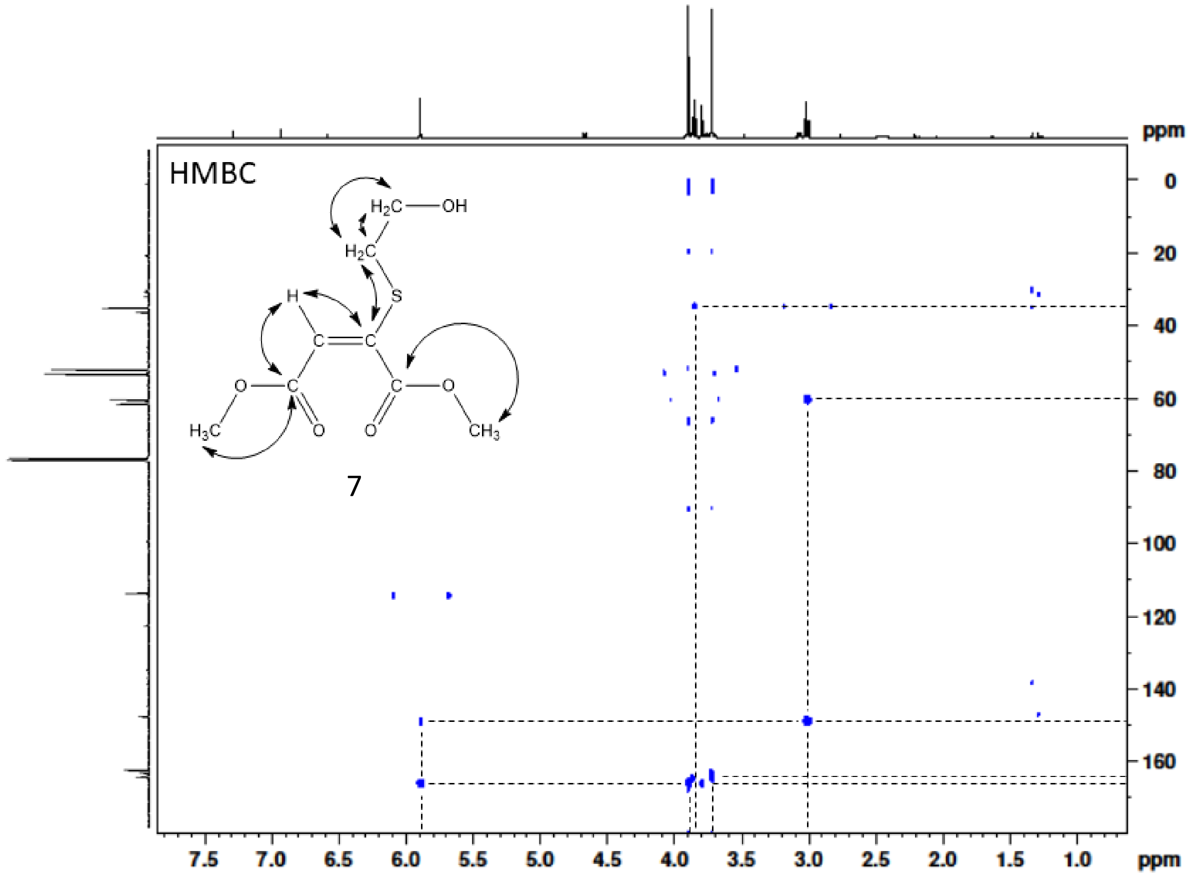

Figure S5. HMBC spectrum of the **6a** fragmentation alkene product **cis-7**.

3.2 Identification of YND-PT 8a diastereomers d1-d3

Diastereomer-enriched samples were collected via flash column chromatography on silica gel (1:6 Et_2O:30-60 Ligroin) of the YND-PT **8a** system. The following analysis describes how each enriched fraction was identified as YND-PT diastereomers **8a:d1-3**. Diastereomer **8a:d4**, which was produced as only 6% of the mixture, was not able to be isolated. **Figure S6** displays the ^{1}H NMR alkene region (6.2-6.8 ppm) clearly identifying the enriched populations of diastereomers in each isolated fraction. NOESY analyses of the diastereomer-enriched fractions were taken to identify through-space coupling of the proton (H_A) added during the conjugate addition with methyl protons (H_B & H_C) atop the ylidene bridge, or the vinyl protons (H_D & H_E) of the endocyclic alkene of the YND-PT. Correlations to bridgehead protons (H_F) and (H_G) helped further confirm the assignments.

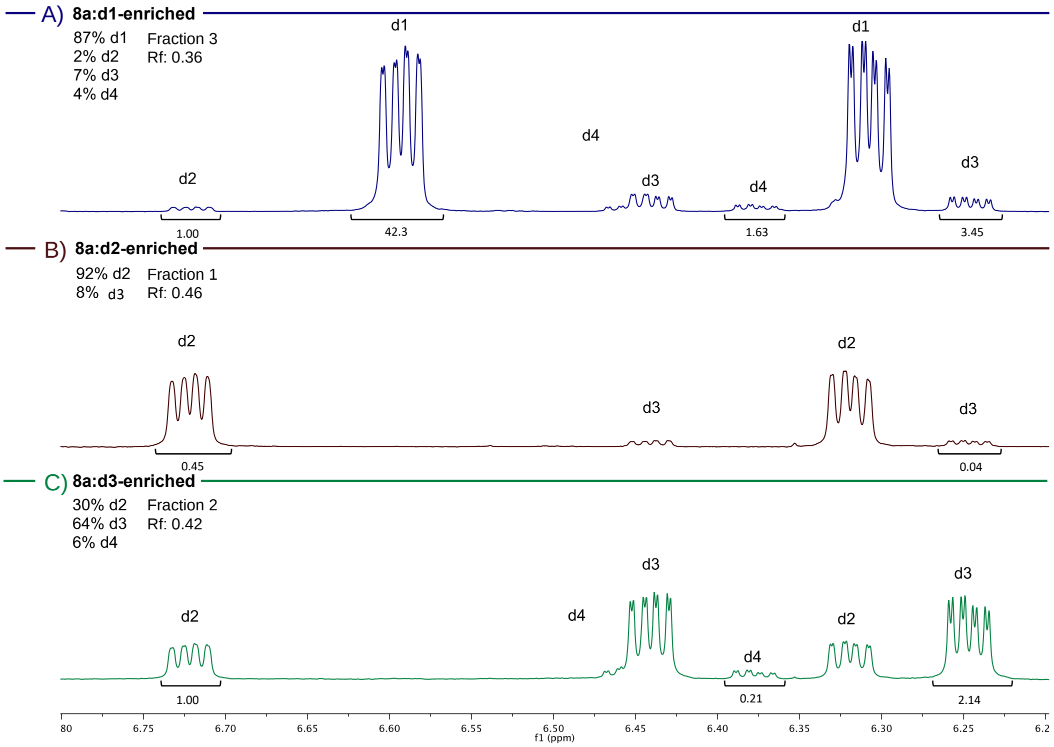

Figure S6. ^{1}H NMR (400 MHz, $CDCl_3$) stack of YND-PT **8a** diastereomer enriched fractions; **A) 8a:d1**-enriched, **B) 8a:d2**-enriched, **C) 8a:d2**-enriched. R_f values measured with 1:6 Et_2O:30-60 Ligroin.

Elucidation of Diastereomer YND-PT ***8a:d1***

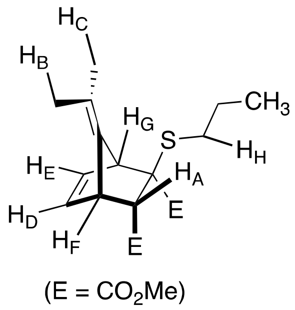

8a:d1 – *syn-exo*
$k_{(DMSO\text{-}d6)}$ = 3.33 ± 0.25 x10^{-4} s^{-1}
$t_{1/2}$ = 34.7 ± 3.6 min

NOESY analysis of the YND-PT **8a:d1**-enriched sample (R_f = 0.36), the most prevalent diastereomer at 38% and the second-fastest to fragment, confirmed a through-space correlation between H_A and H_B atop the ylidene bridge (**Figure S8**), suggesting that proton H_A is in the *exo* position of the norbornene structure. Furthermore, correlations were observed between the protons H_H adjacent to the sulfur atom and H_A and with the bridgehead H_G proton (**Figure S9**), supporting **8a:d1** as the *syn-exo* diastereomer.

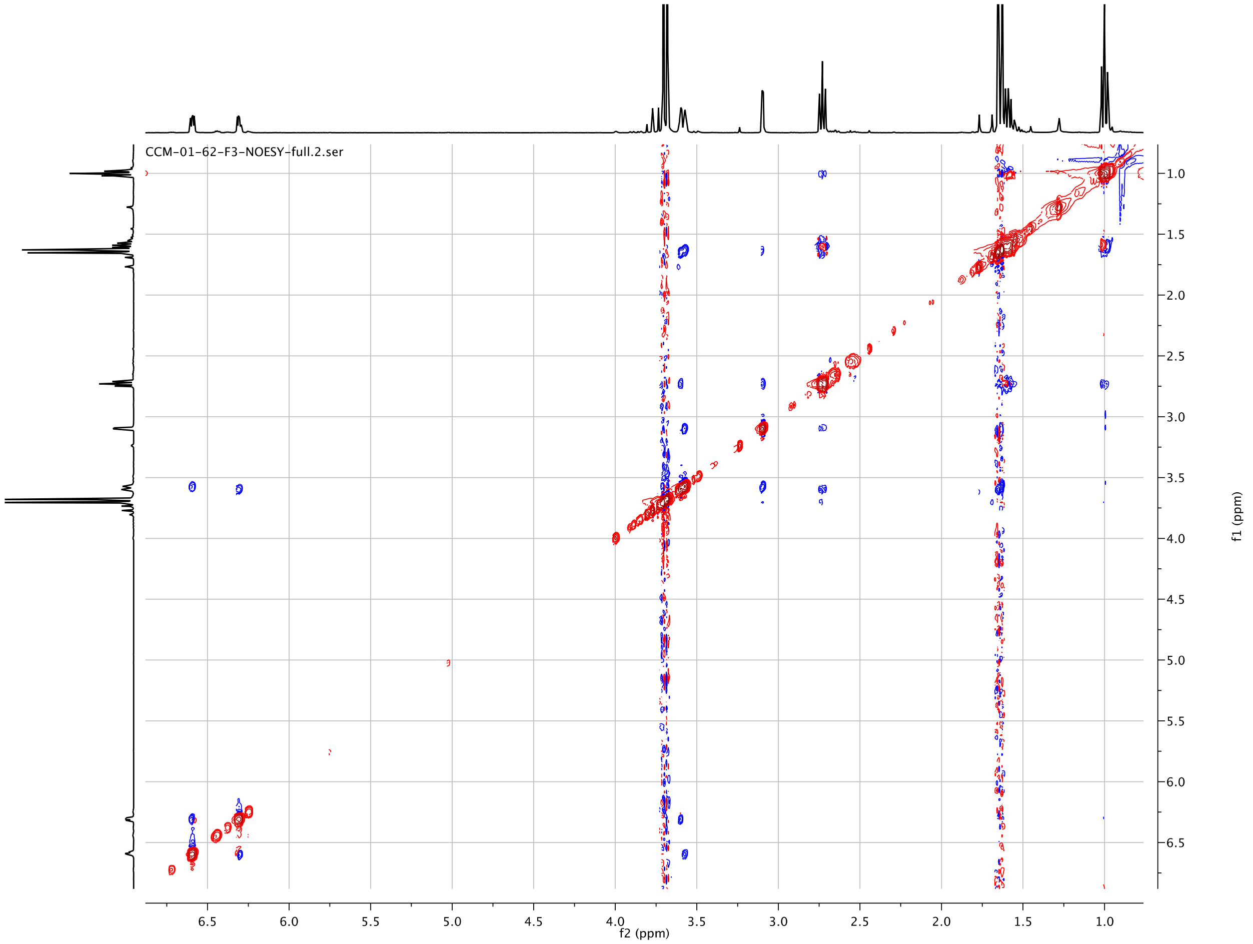

Figure S7. NOESY Full spectrum of YND-PT **8a:d1.**

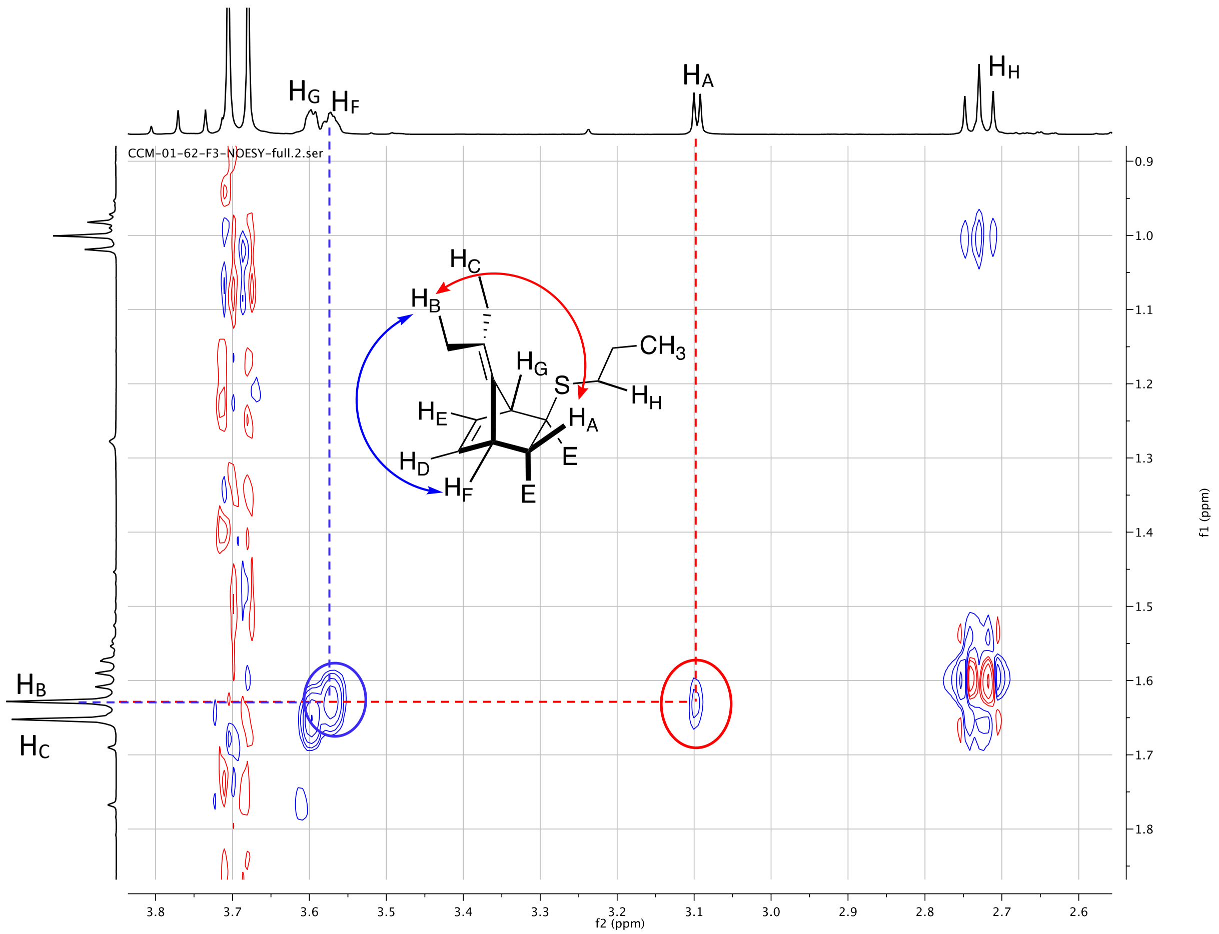

Figure S8. NOESY zoomed-in spectrum of YND-PT **8a:d1,** highlighting H_A-H_B and H_B-H_F correlations (E = CO_2Me).

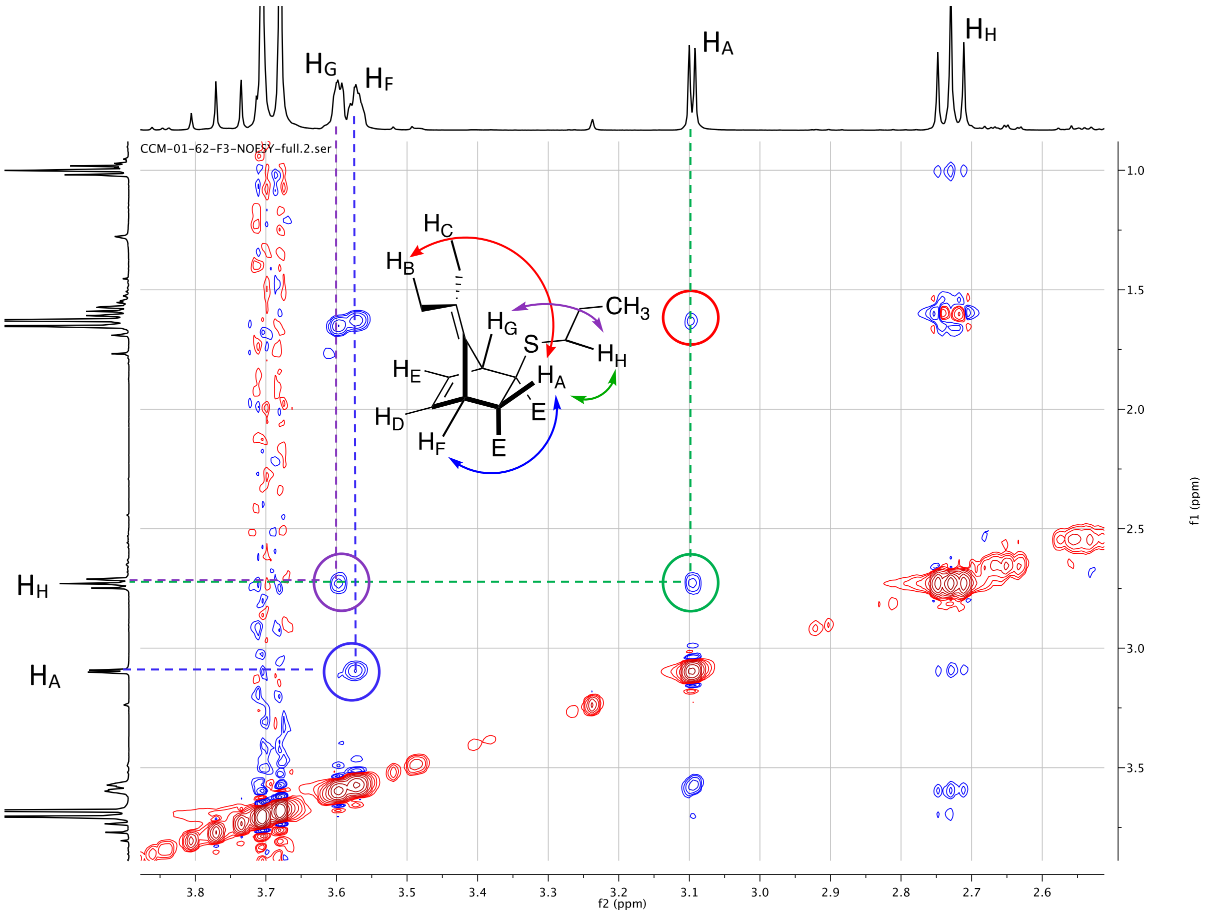

Figure S9. NOESY zoomed-in spectrum of YND-PT **8a:d1**, highlighting H_A-H_H, H_A-H_F, H_A-H_B, and H_G-H_H correlations (E = CO_2Me).

*Elucidation of Diastereomer YND-PT **8a:d2***

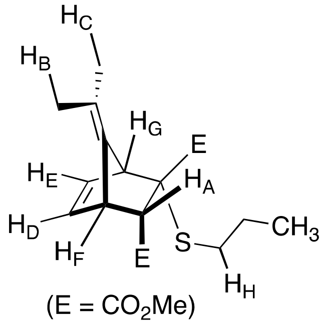

(E = CO_2Me)

8a:d2 – *anti-endo*
$k_{(DMSO-d6)} = 2.33 \pm 0.15 \times 10^{-3}\ s^{-1}$
$t_{1/2} = 4.96 \pm 0.31$ min

NOESY analysis for a **8a:d2**-enriched sample ($R_f = 0.46$), the fastest to fragment and isolated as 28% of the diastereomeric mixture, proved the most difficult of the three diastereomers to confirm. The expected correlation between proton H_A and the methyl protons H_B on top of the ylidene bridge was not easily identified as the correlation signal was adjacent to the stippling, or T1 noise resulting from one of the methyl ester singlets (**Figure S11 - red circle**). When the MestReNova "Reduce T1 noise" process is applied to the spectrum, the correlation is much more notable (**Figure S12 - red circle**). Furthermore, as observed in the spectrum of **8a:d1**, the signal for H_A of **8a:d2** appeared as a doublet, whereas the signal for H_A in the spectrum of the **8a:d3**-enriched sample (vide infra) appears as a singlet. These two observations, coupled with the lack of a correlation from H_A to $H_{D,}$ confirmed H_A to be in the *exo* position of the norbornene. The absence of H_A-$H_{H/H'}$ correlations as observed in the NOESY spectrum for **8a:d1** proved the *anti* relationship between the thioether and proton H_A. Interestingly, the protons alpha to the thioether (H_H and $H_{H'}$) were both observed as distinct signals with complex splitting patterns (1H, dt). This was noted for both *anti*-diastereomers **8a:d2** and **8a:d3** (vide infra). Conversely, the signal for the *syn*-diastereomer **8a:d1** was observed as a single 2H triplet for both H_H protons, providing further evidence that **8a:d2** is the *anti-endo* diastereomer (*endo*- with respect to thioether).

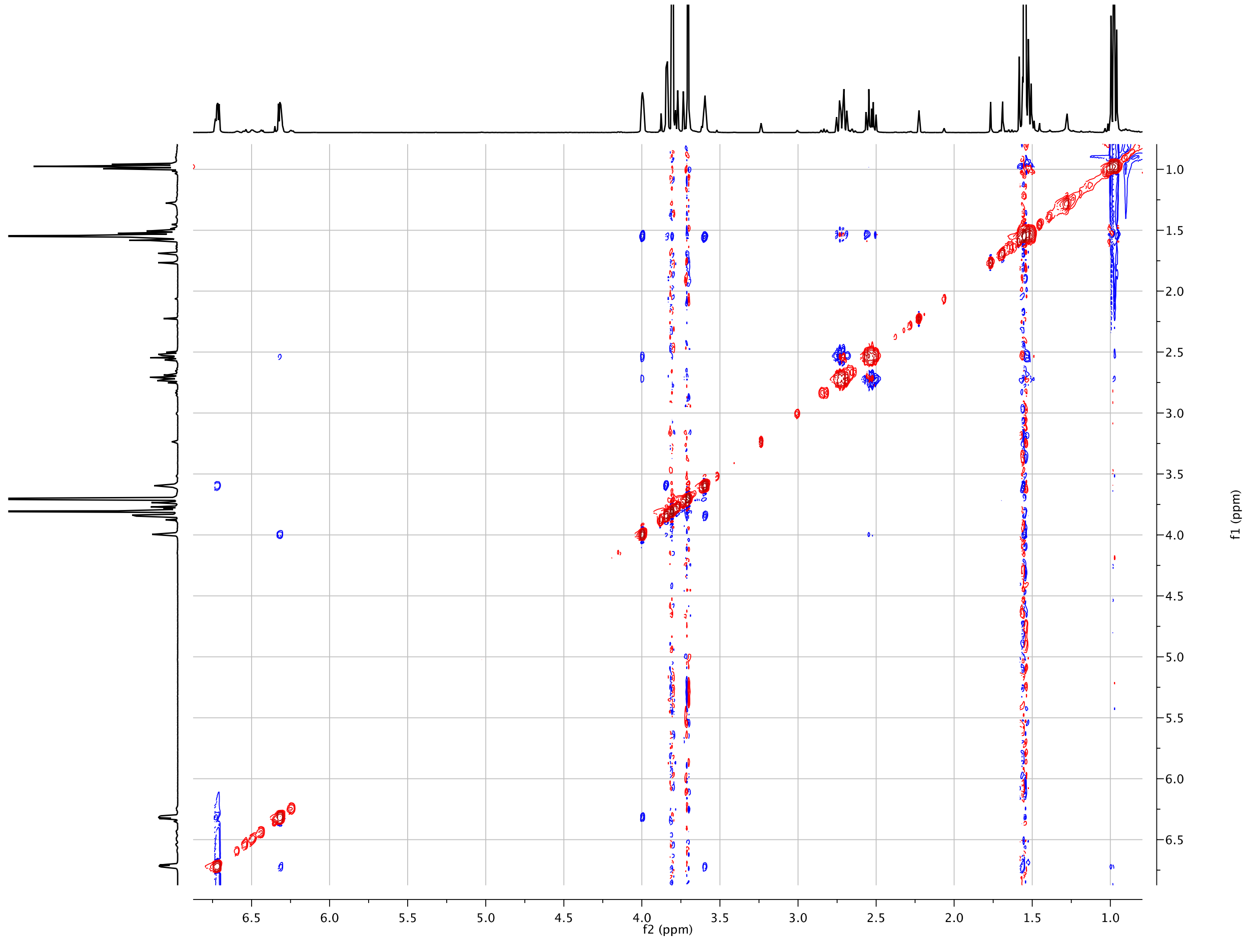

Figure S10. NOESY Full spectrum of YND-PT **8a:d2**

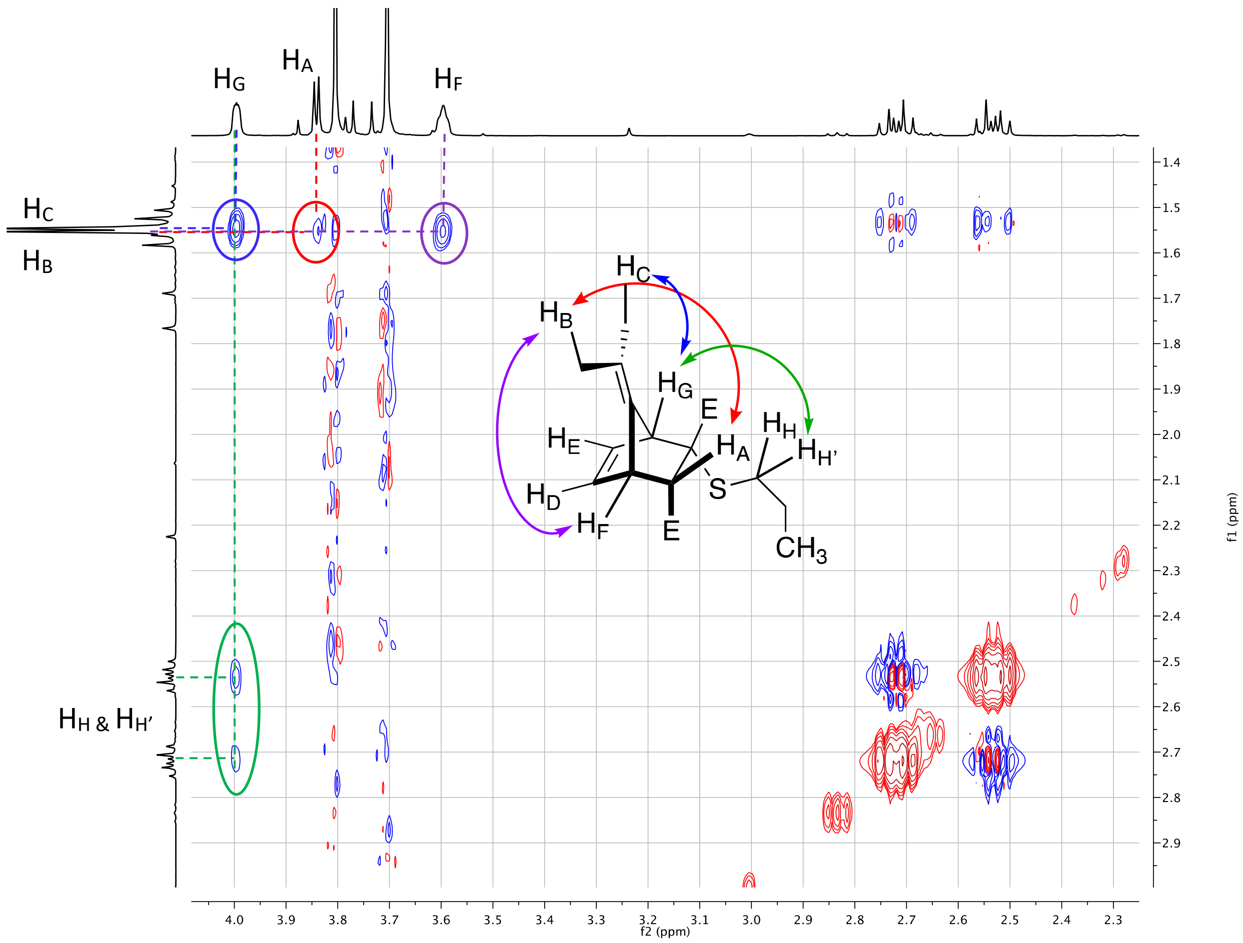

Figure S11. NOESY zoomed-in spectrum of YND-PT **8a:d2,** highlighting H_A-H_B, H_B-H_F, H_C-H_G, and H_G-$H_{H/H'}$ correlations (E = CO_2Me).

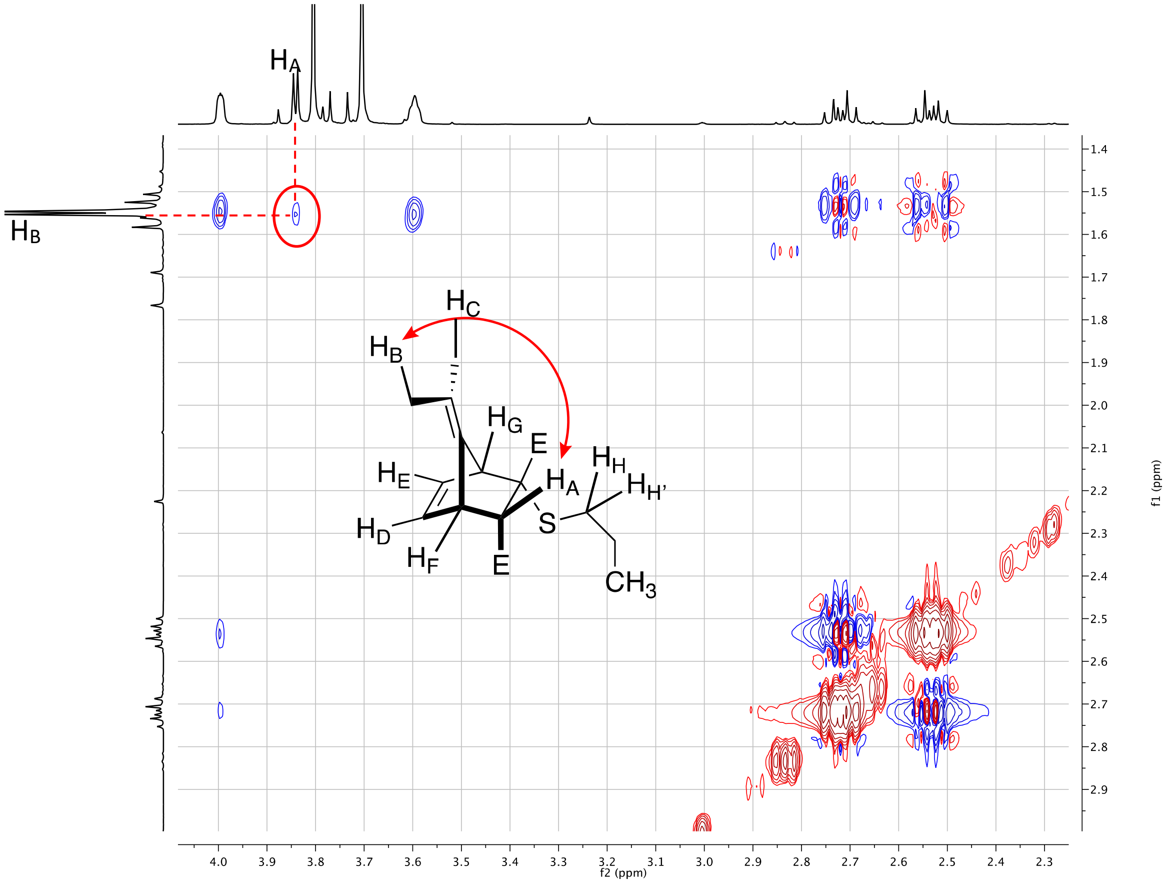

Figure S12. NOESY zoomed-in spectrum *with T1 noise reduced* of YND-PT **8a:d2,** highlighting H_A-H_B, correlation. (E = CO_2Me).

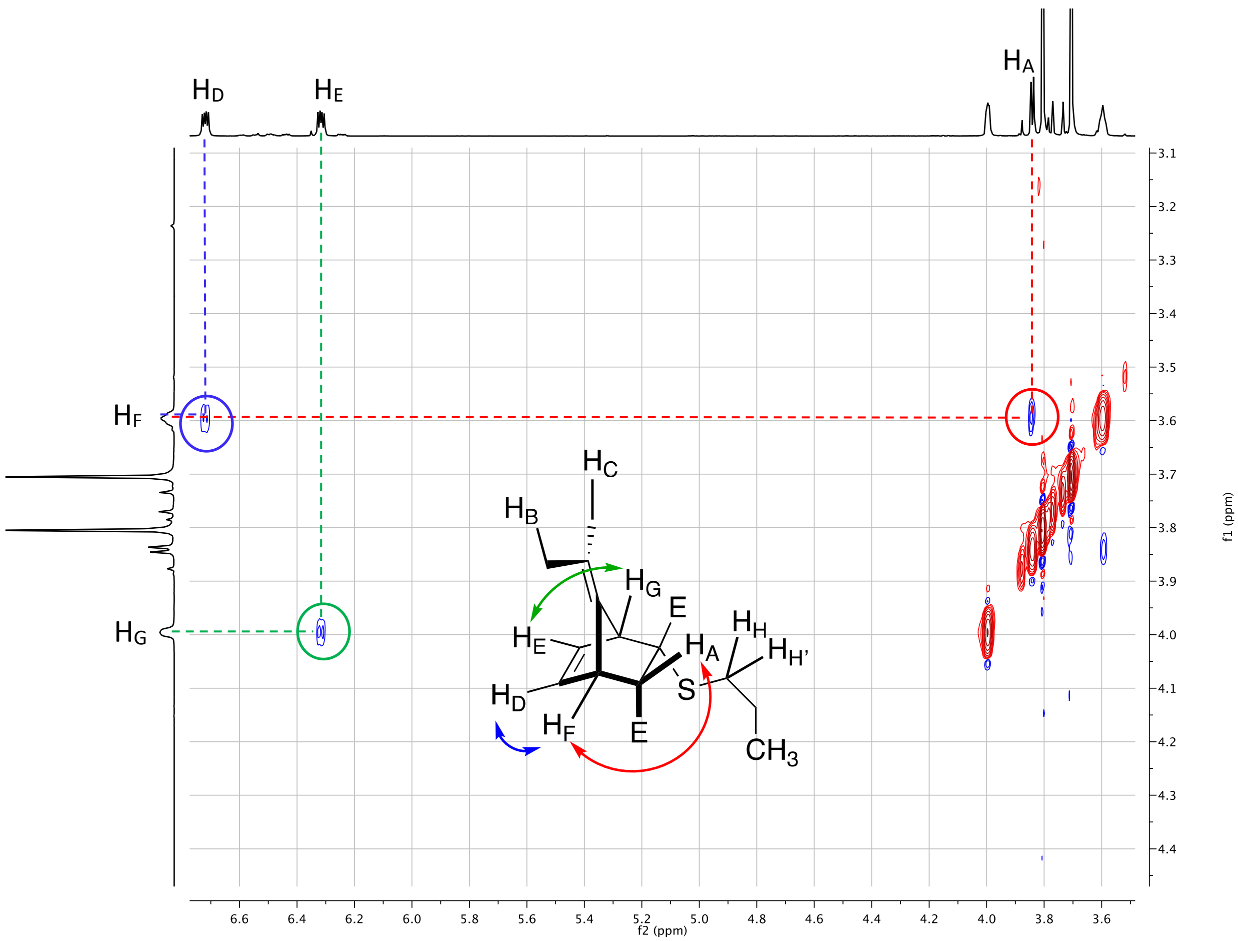

Figure S13. NOESY zoomed-in spectrum of YND-PT **8a:d2,** highlighting H_A-H_F, H_D-H_F, and H_G-H_e correlations (E = CO_2Me).

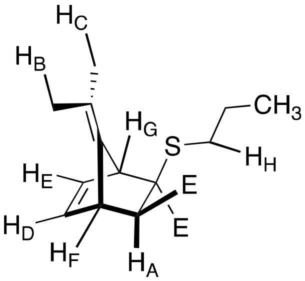

(E = CO_2Me)

8a:d3 – *anti-exo*
$k_{(DMSO\text{-}d6)}$ = 8.33 ± 1.02 x10^{-5} s^{-1}
$t_{1/2}$ = 139 ± 16 min

The NOESY analysis of a **8a:d3**-enriched sample (R_f = 0.42), the slowest to fragment and isolated as 28% of the diastereomeric mixture, had no correlation present between H_A and either of the methyl protons atop the ylidene bridge (H_B and H_C). This suggested that H_A is oriented in the *endo* position of the norbornene structure. Additionally, a correlation between H_A and the vinyl proton H_D confirmed H_A as being in an *endo* position. (**Figure S15**).

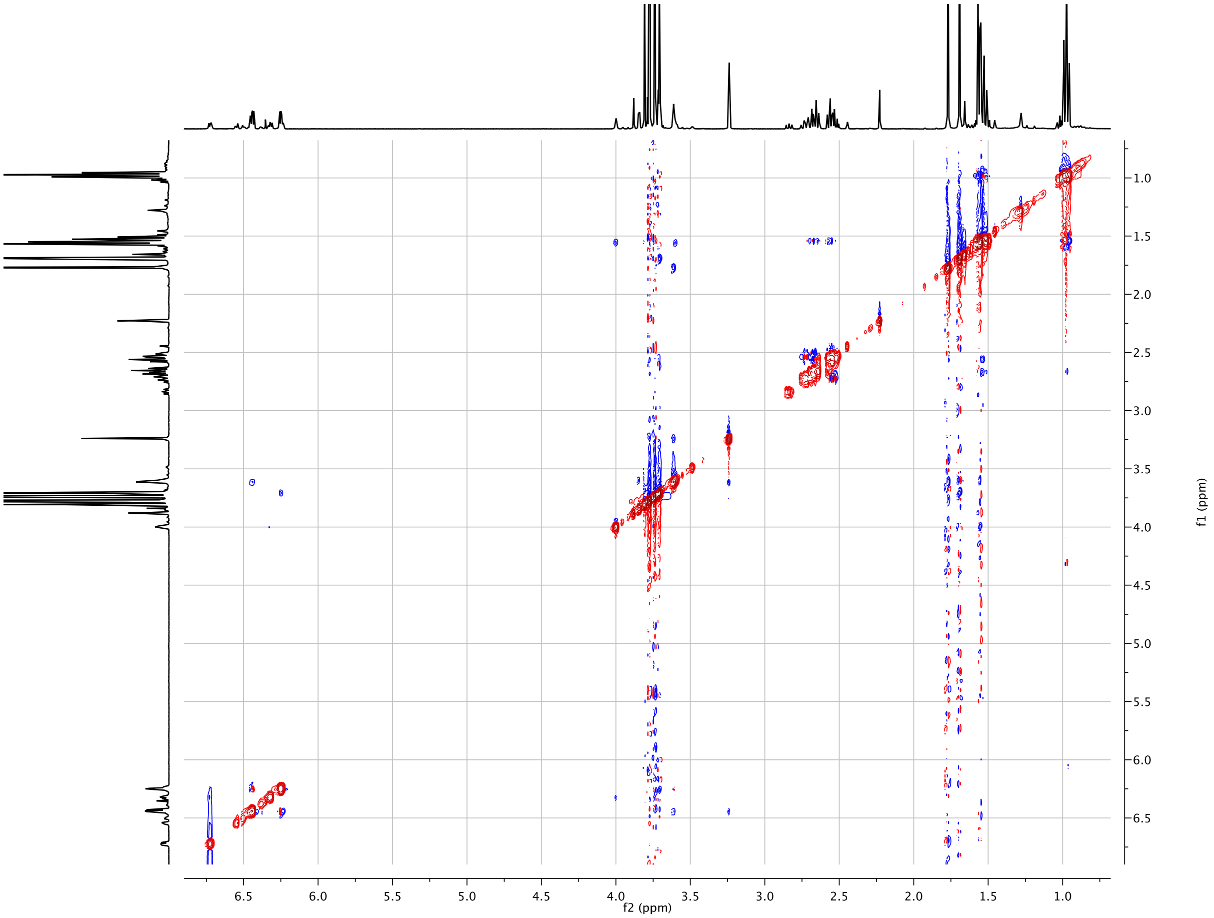

Figure S14. NOESY Full spectrum of YND-PT **8a:d3**

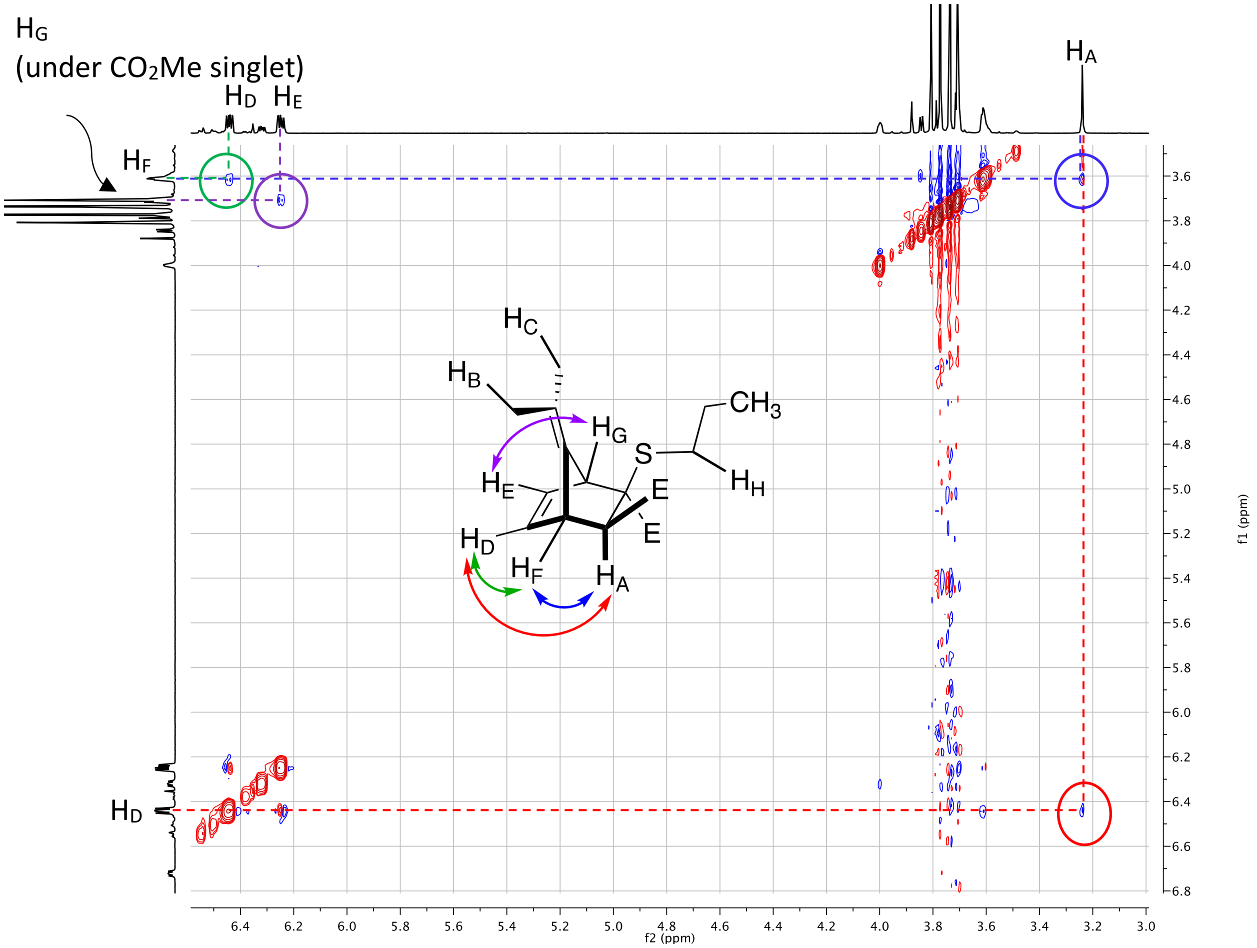

Figure S15. NOESY zoomed-in spectrum of YND-PT **8a:d3,** highlighting H_A-H_D, H_A-H_F, H_D-H_F, and H_E-H_G correlations (E = CO_2Me).

4. Comparison of Hammett Substrate Diastereomers

Because aromatic functionalized YNDs **5o-u** were asymmetric on the ylidene bridge, reacting with the BME nucleophile to form YND-BMEs **6o-u** afforded a complex inseparable mixture of eight diastereomers. In order to compare the observed rate constants of this complex diastereomeric mixture for the Hammett study substrate scope, it was imperative to ensure similar ratios of diastereomers across all seven samples (**6o-u**). As seen in **Figure S16** below, the relative signal heights of the 8 singlets arising from the 8 diastereomers for the ylidene bridge vinylic proton are very similar.

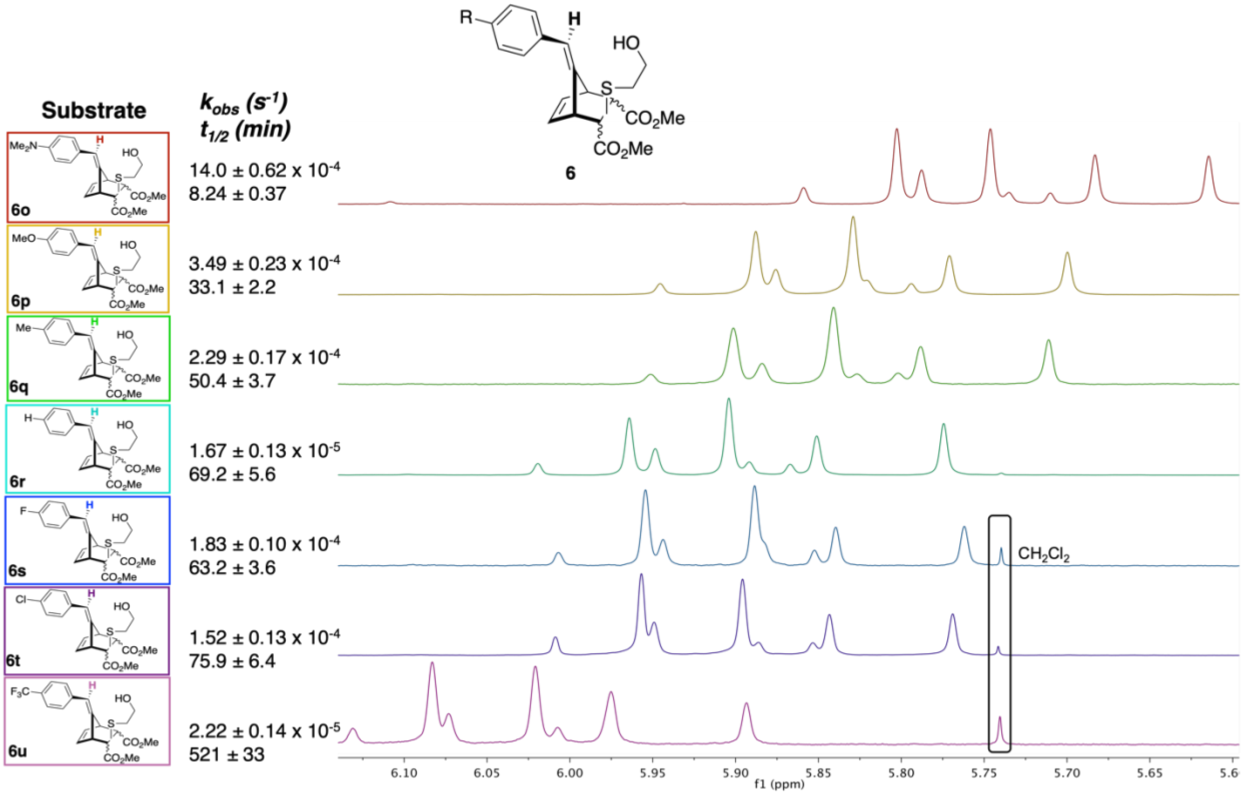

Figure S16. ^{1}H NMR spectra stack qualitatively comparing the ratios of diastereomers based on the resonance of the vinylic proton on the ylidene bridge.

5. Computational search of PES for lower TS energy of *anti-exo* diastereomer

We initially began our DFT calculations utilizing incorrectly assigned structures for the *anti*-diastereomers (**6a:d2** and **6a:d3**). The *syn*-diastereomers (**6a:d1** and **6a:d4)** were initially assigned correctly. The calculated free energies of activation of the retro-[4+2] cycloaddition for **6a:d1** (26.2 kcal/mol) and the diastereomer we *incorrectly* assigned as **6a:d3** (27.7 kcal/mol) were consistent with the experimental results, both being within 0.6 kcal/mol. As mentioned previously, accurate experimental rate measurements could not be made for **6a:d4**. However, the calculated free energy of activation for the fragmentation of the minor **6a:d4** diastereomer was calculated to be larger, 30.3 kcal/mol, and was consistent with the observation that this diastereomer was the slowest to fragment. Interestingly, the calculated free energy of activation for the diastereomer that was fastest to fragment, *incorrectly* assigned as **6a:d2**, was significantly higher (5.2 kcal/mol) than the experimental value (**Figure S17**).

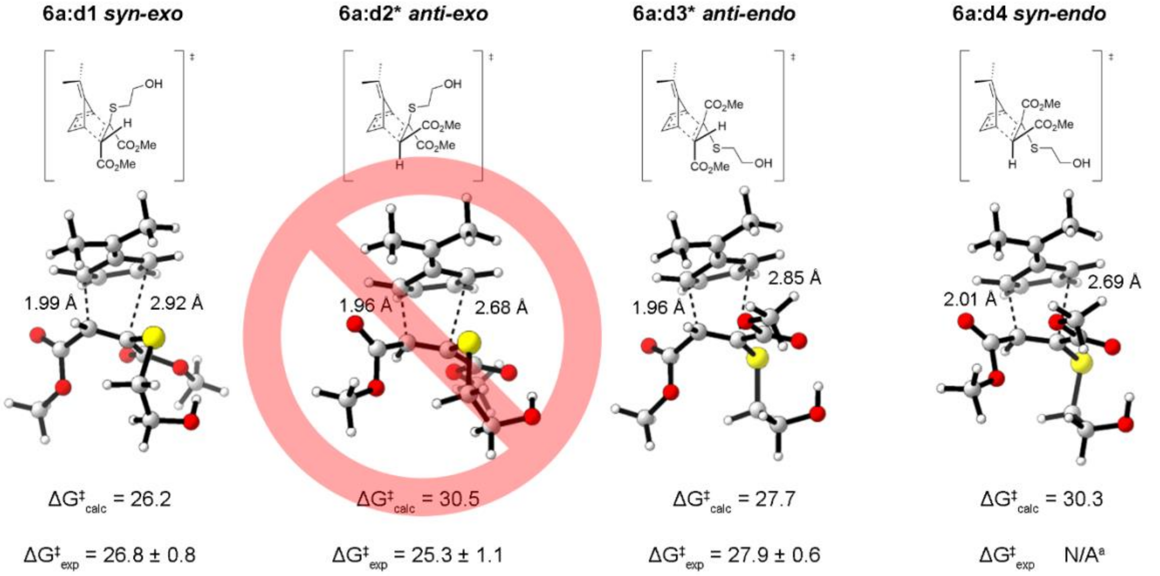

Figure S17. The calculated and experimental free energies of activation for **TS1-6a:d1** and **TS1-6a:d4**, and the initially *INCORRECTLY* assigned ***TS1-6a:d2*** and ***TS1-6a:d3***. Energies were calculated at 25 °C and 1 atm with DMSO implicit solvent, and the experimental energies were determined at 25 °C using the Arrhenius parameters.[3]

[a] Experimental free energy of activation for **6a:d4** was not measured because this diastereomer was rerecovered as only 4% of the total diastereomeric mixture and fragmented much slower than the other three diastereomers.

With three of the four calculated energies seemingly well-aligned with the experimental values, we first looked at the discrepancy in the energies of the *anti-exo* diastereomer (originally incorrectly assigned as **6a:d2***), and turned our attention to finding a lower calculated transition state structure energy. We first made an exhaustive conformer search by re-ranking conformers generated by CREST,[9] MacroModel,[10,11] and chemical intuition using single point calculations with

ωB97X-D/6-311+G(d,p) and DMSO implicit solvent. Through this search, a significantly lower energy calculated transition state structure was not found.

Scheme S4. Computationally Investigated Mechanisms for YND-BME *anti-exo* diastereomer (Energies in kcal/mol).

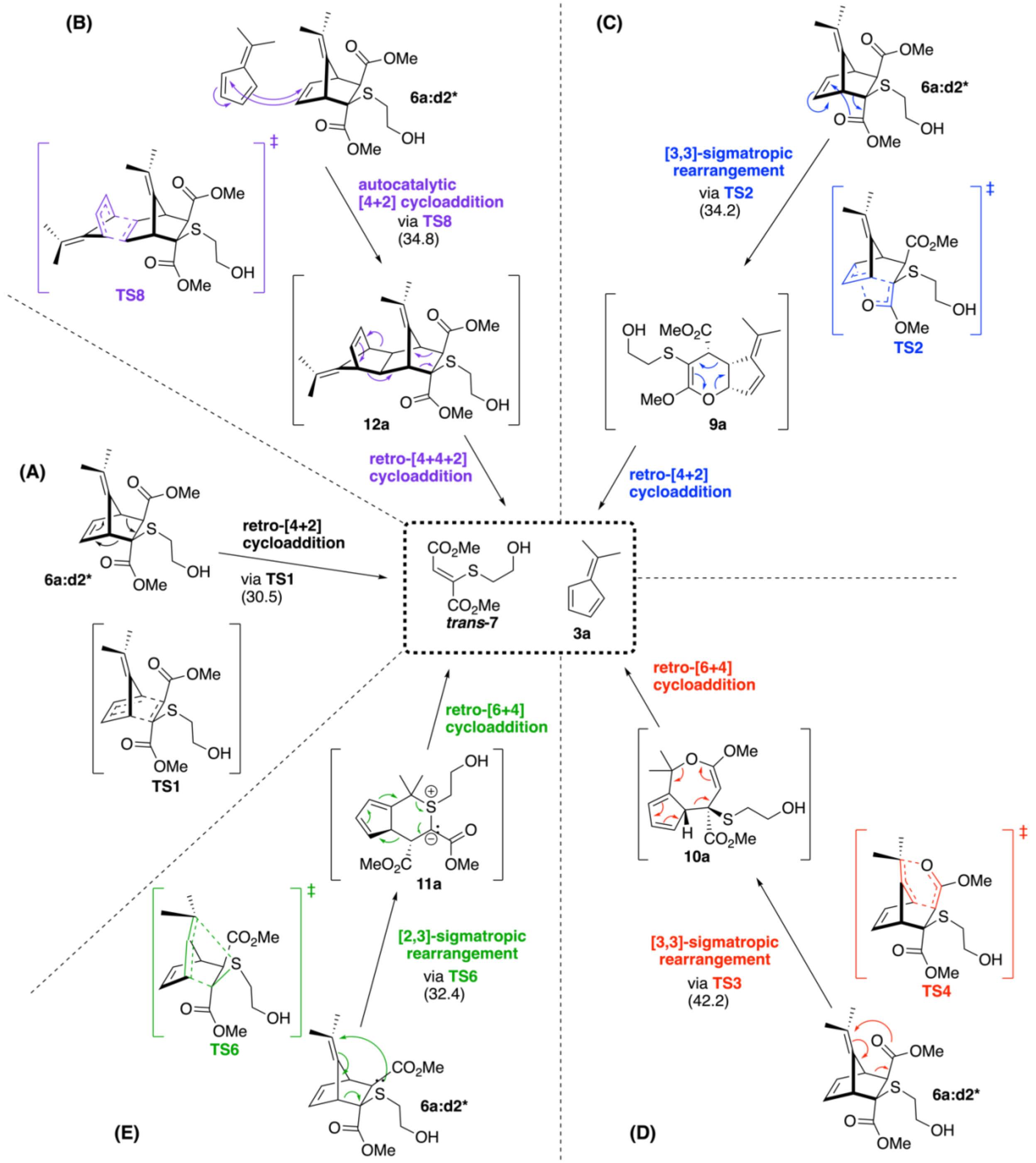

Next, based on the fact that fulvenes have been proven to undergo a variety of cycloaddition reactions,[12–19] four alternate mechanisms of fragmentation were also explored (**Scheme S4**): **(B)** an autocatalytic fragmentation mechanism,[20,21] wherein the YND-BME reacts initially with a fulvene molecule in a [4+2] cycloaddition (**TS8** = 34.8 kcal/mol), followed by a retro[4+4+2] cycloaddition, **(C)** a [3,3]-sigmatropic rearrangement with the *endo*-carbonyl and the endocyclic alkene in the ylidenenorbornene core (**TS2** = 34.2 kcal/mol), followed by a retro-[4+2] cycloaddition, **(D)** a [3,3]-sigmatropic rearrangement in which the *exo*-ester and the exocyclic alkene of the ylidenenorbornene structure participate (**TS4** = 42.2 kcal/mol), followed by a retro-[6+4] cycloaddition, and **(E)** a [2,3]-sigmatropic rearrangement involving the sulfur atom and the exocyclic alkene of the ylidenenorbornene (**TS6** = 32.4 kcal/mol), followed by a retro-[6+4] cycloaddition. All four of these potential pathways provide identical products to a direct retro-[4+2] fragmentation of YND-BME **6a**. However, none of these mechanisms provided a lower activation barrier for fragmentation of the *anti-exo* diastereomer (**Figure S18**).

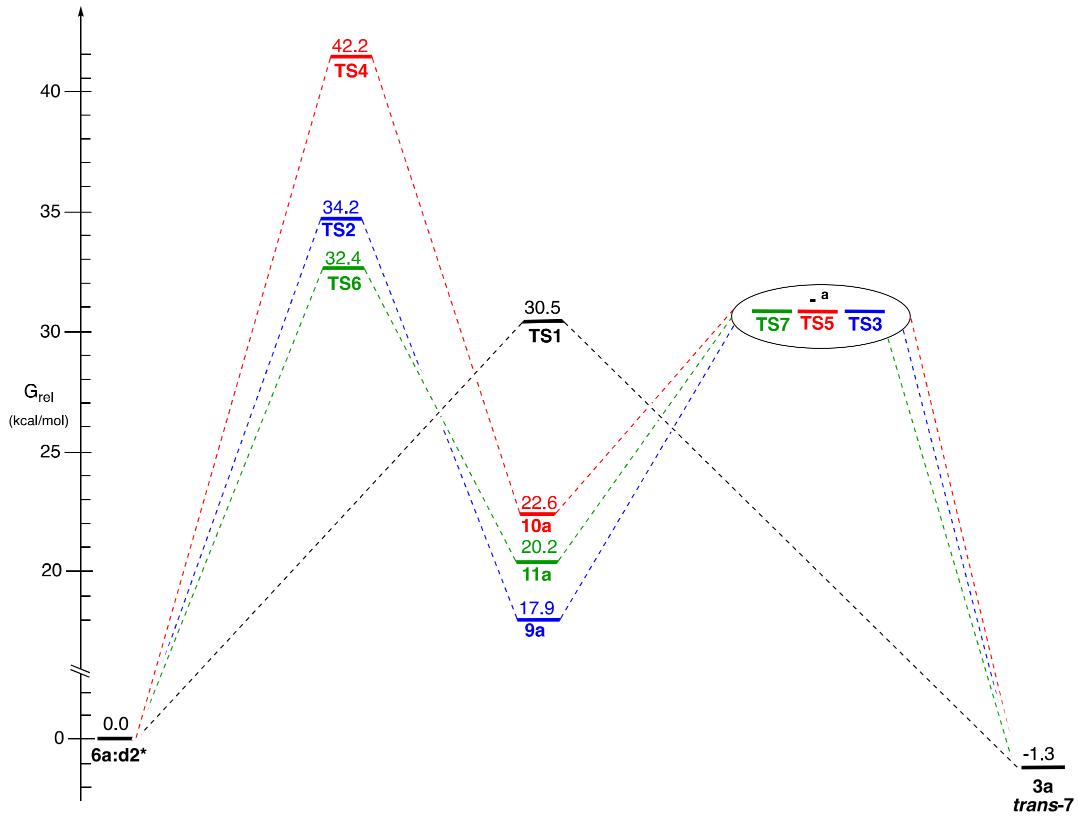

Figure S18. Energy coordinate for computationally explored mechanisms of **6a:d2*** (ωB97X-D/6-31+G(d,p)). Energies in kcal/mol.

[a] **TS3**, **TS5**, and **TS7** were not investigated as **TS2**, **TS4**, and **TS6** were all higher energy than **TS1**.

6. ^{1}H NMR Kinetic Studies

^{1}H NMR kinetic studies for the purified YND-BME and YND-PT adducts were performed according to the previously published procedure outlined in the supporting information section of Malouf et. al.[3] Purified YND-BME adduct (20-25 mg) was dissolved in DMSO-d_6 (0.5 mL) and placed in a standard 5-mm NMR tube with 1,4-Dinitrobenzene (DNB) (approximately 0.003 g) as the internal standard.[22] ^{1}H NMR kinetic studies were performed on a Bruker Ascend 400 using the multiple run variable/fixed delay [multi_zgvd] experiment with automated lock, tune and match, and shimming and the following acquisition parameters: AQ = 4.0894465 sec, DW = 62.4 msec, DE = 6.50 msec, D1 = 5 sec, DS = 0, NS = 4, P1 = 9.750. An initial reference ^{1}H NMR was collected to serve as a zero-time point prior to exposure to heat for the fragmentation study. The sample was then removed and replaced with an empty tube. When the probe temperature reached the desired temperature the sample was reinserted, and the sample solvent was relocked and tuned and shimmed. The time for this process was recorded and accounted for in the timing of the kinetic data. A ^{1}H NMR was acquired at fixed time delays until the sample reached at least 90% degradation. Spectra were auto-phased and baseline corrected using MestreNova software. Integrals for isolated peaks from the DNB internal standard, BME-adducts, and fulvene degradation products were measured until at least 90% fragmentation was observed. This data was used to plot the fragmentation progress and the integrated first-order rate decays. Each substrate and temperature sample were repeated in triplicate and error values are given with a 95% confidence. Data for substrates **6f** and **6l-n** can be found in our previous report.[3] Data for substrates **6b-d** were reanalyzed from our previous report for reduced error in the extrapolated rate constants.

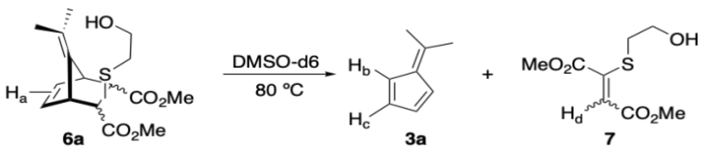

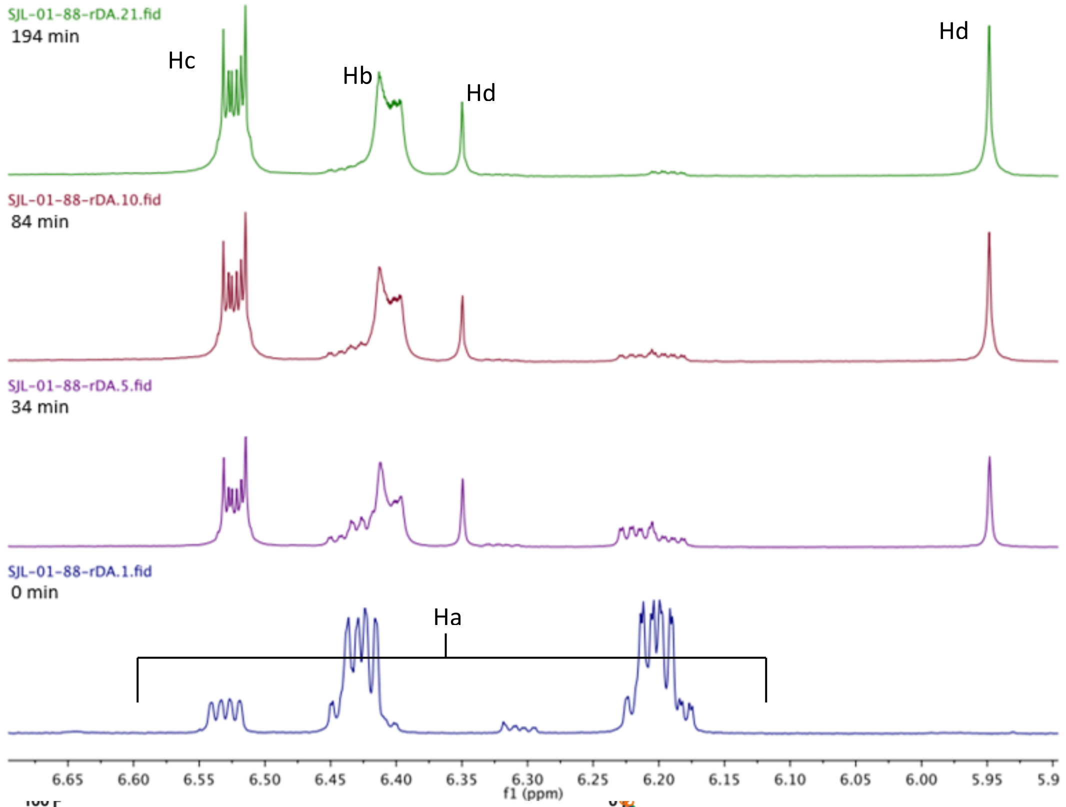

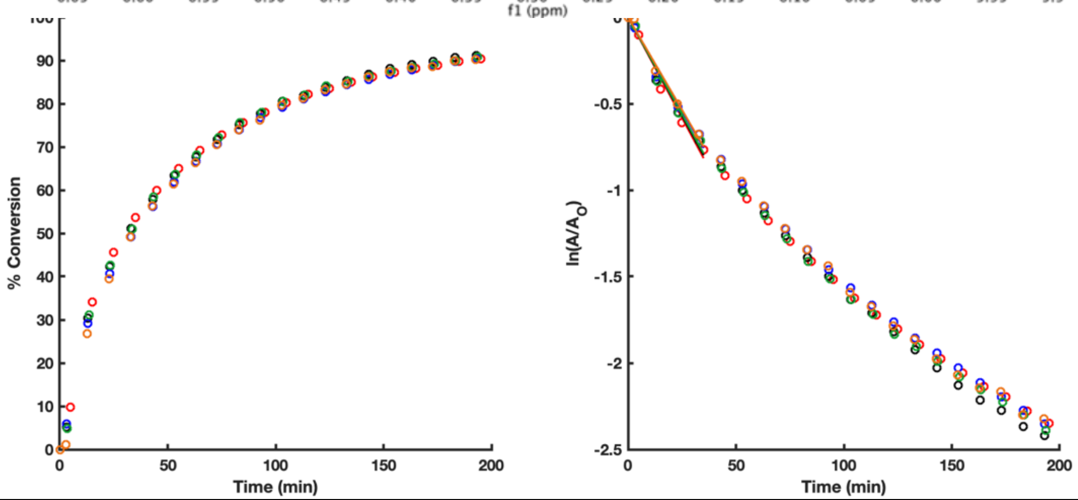

Trial	*k (s^{-1})*	*avg k (s^{-1})*	*$t_{1/2}$ (min)*	*avg $t_{1/2}$ (min)*
1	3.88E-04	3.72 ± 0.17-04	29.7	31.1 ± 1.4
2	3.80E-04		30.4	
3	3.60E-04		32.1	
4	3.75E-04		30.8	
5	3.57E-04		32.4	

Figure S19. Fragmentation kinetics of YND-BME **6a:d1-3** at 80 °C in DMSO-d6.

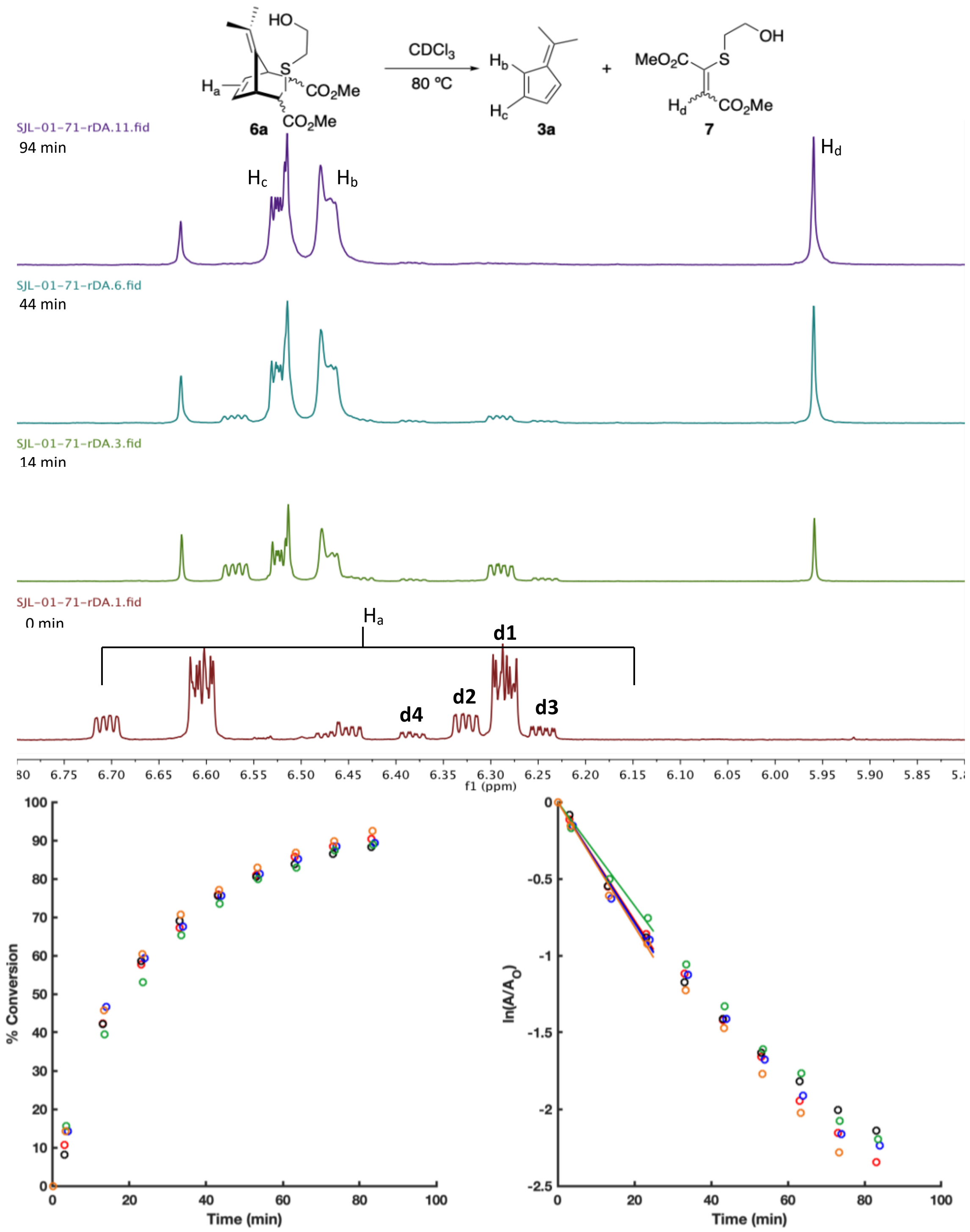

Trial	*k (s⁻¹)*	*avg k (s⁻¹)*	*t1/2 (min)*	*avg t1/2 (min)*
1	6.40E-04	6.36 ± 0.54 E-04	18.1	18.2 ± 1.7
2	6.52E-04		17.7	
3	6.55E-04		17.6	
4	5.62E-04		20.6	
5	6.72E-04		17.2	

Figure S20. Fragmentation kinetics of YND-BME **6a:d1-3** at 80 °C in $CDCl_3$.

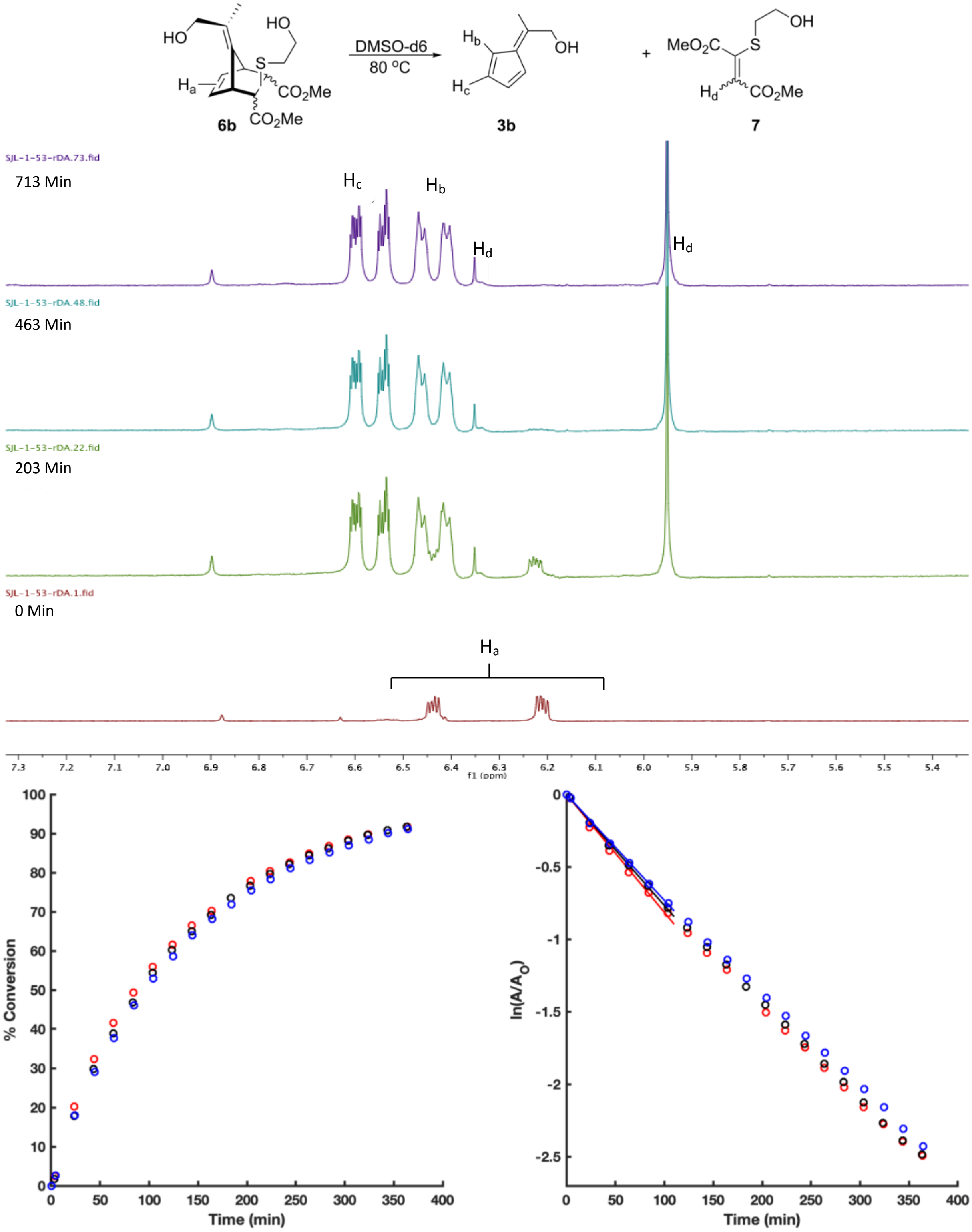

Trial	*k (s^{-1})*	*avg k (s^{-1})*	*t$_{1/2}$ (min)*	*avg t$_{1/2}$ (min)*
1	1.36E-04	1.29 ± 0.16 E-04	85.0	89.6 ± 11.3
2	1.29E-04		89.6	
3	1.23E-04		94.2	

Figure S21. Fragmentation kinetics of YND-BME **6b** at 80 °C.

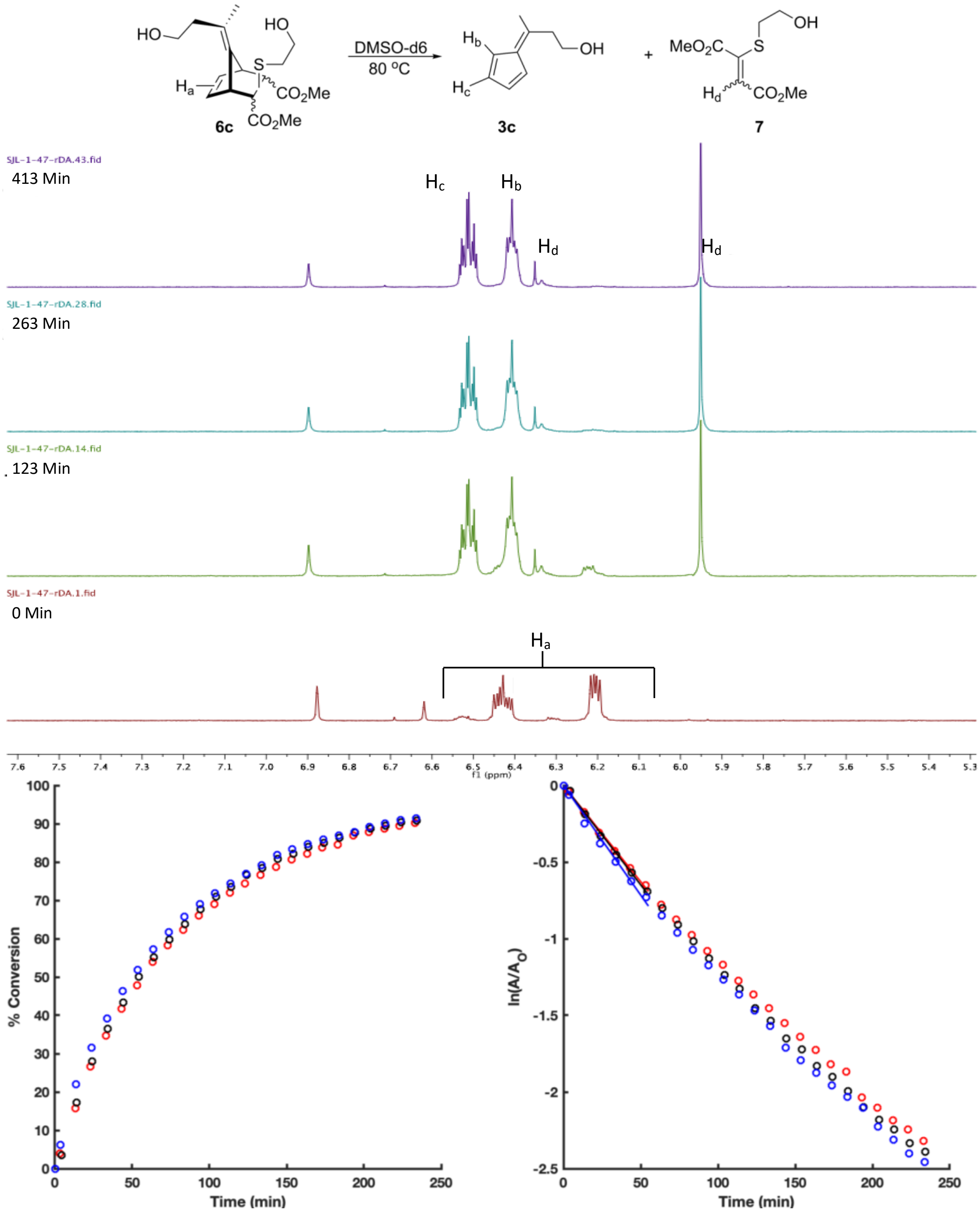

Trial	*k (s*$^{-1}$*)*	*avg k (s*$^{-1}$*)*	*t*$_{1/2}$ *(min)*	*avg t*$_{1/2}$ *(min)*
1	2.08E-04	2.18 ± 0.31 E-04	55.5	53.2 ± 7.3
2	2.13E-04		54.2	
3	2.32E-04		49.9	

Figure S22. Fragmentation kinetics of YND-BME **6c** at 80 °C.

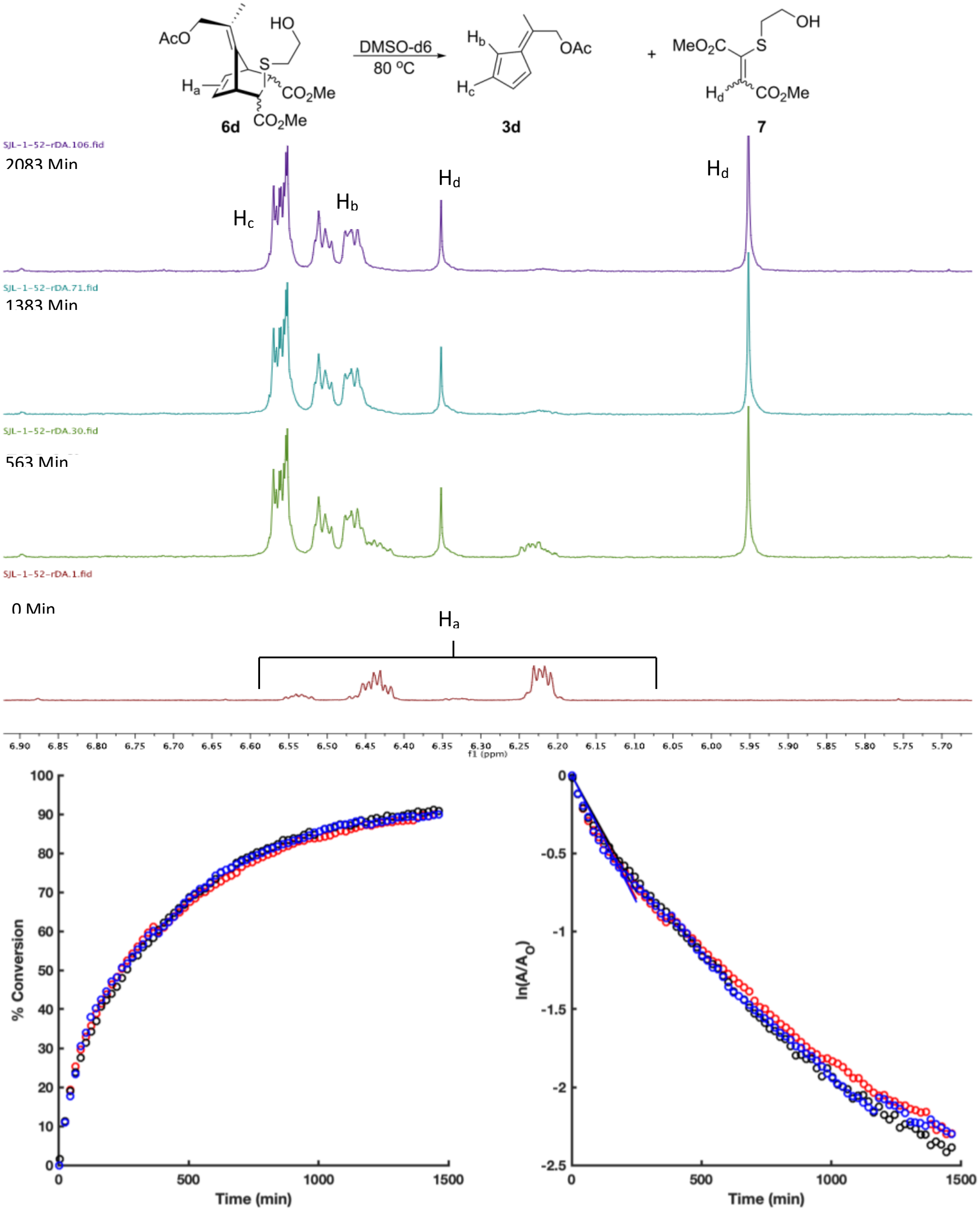

Trial	*k (s^{-1})*	*avg k (s^{-1})*	*$t_{1/2}$ (min)*	*avg $t_{1/2}$ (min)*
1	5.18E-05	5.11 ± 0.50 E-05	223	226 ± 23
2	4.88E-05		237	
3	5.27E-05		219	

Figure S23. Fragmentation kinetics of YND-BME **6d** at 80 °C.

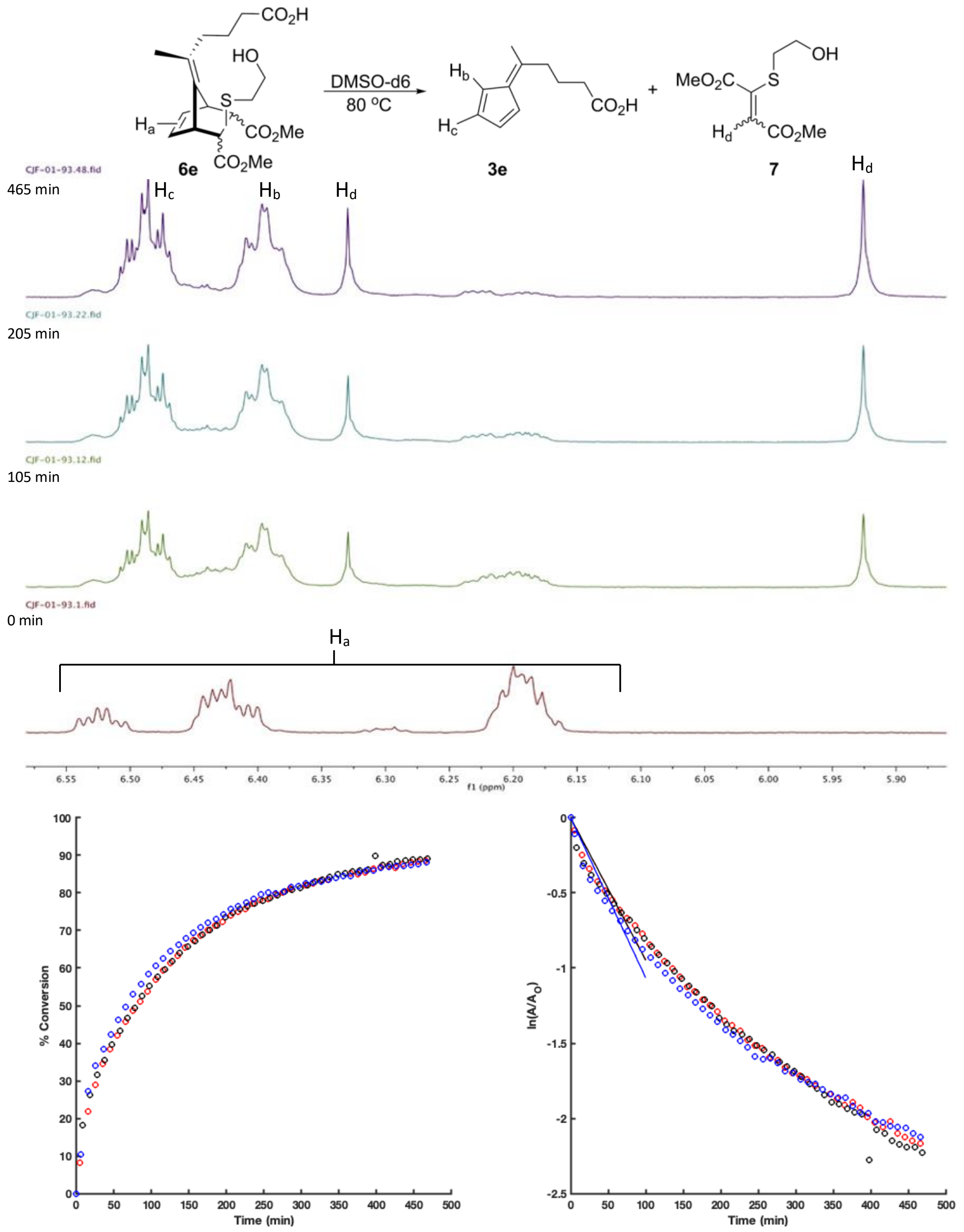

Trial	*k* (s^{-1})	*avg k* (s^{-1})	$t_{1/2}$ *(min)*	*avg* $t_{1/2}$ *(min)*
1	1.58E-04	1.64 ± 0.27 E-04	73.3	70.6 ± 11.3
2	1.58E-04		73.1	
3	1.77E-04		65.4	

Figure S24. Fragmentation kinetics of YND-BME **6e** at 80 °C.

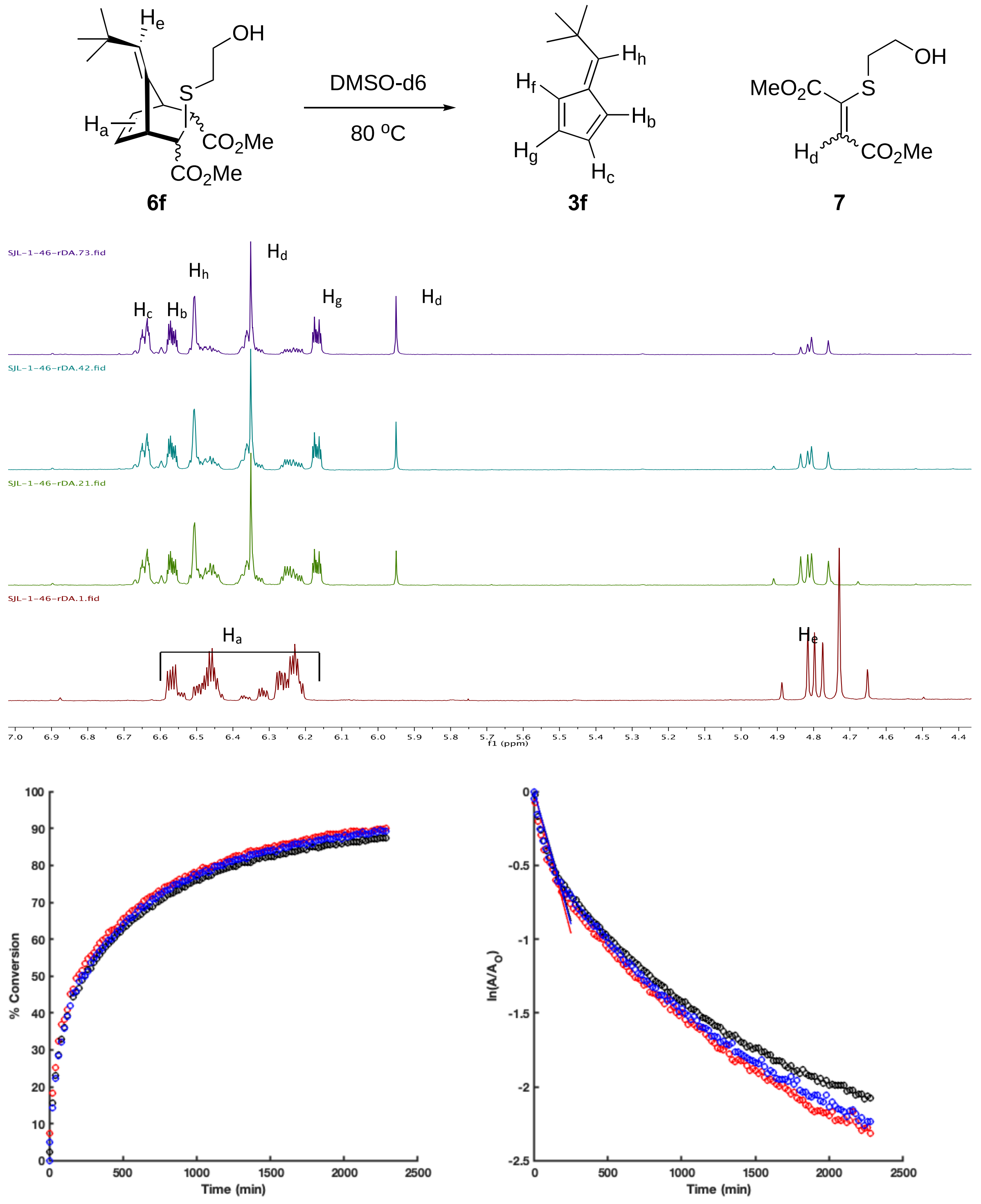

Trial	*k (s⁻¹)*	*avg k (s⁻¹)*	*t1/2 (min)*	*avg t1/2 (min)*
1	6.15E-05	5.83 ± 0.70 E-05	188	199 ± 23
2	5.62E-05		206	
3	5.72E-05		202	

Figure S25. Fragmentation kinetics of YND-BME **6f** at 80 °C.

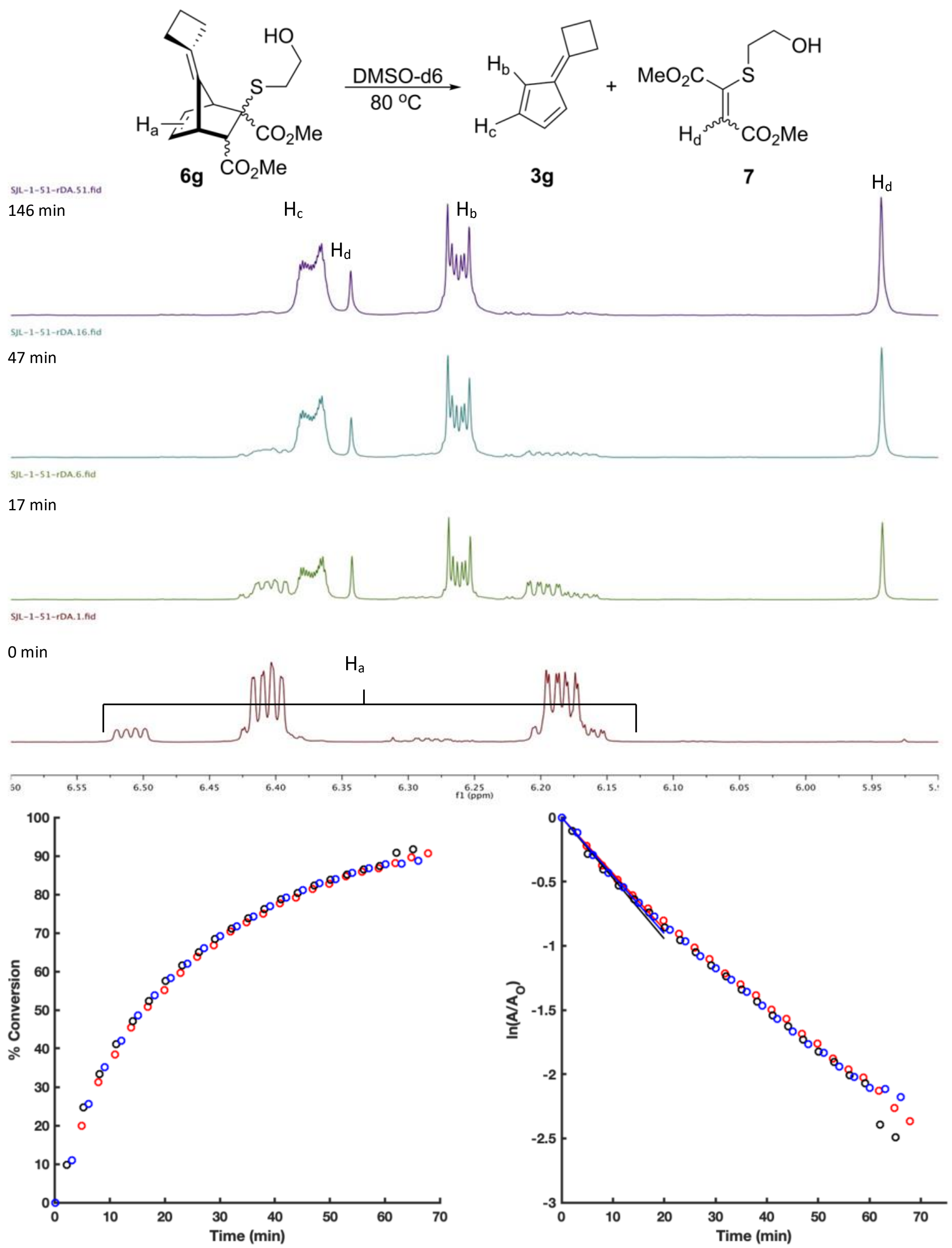

Trial	*k (s^{-1})*	*avg k (s^{-1})*	*$t_{1/2}$ (min)*	*avg $t_{1/2}$ (min)*
1	7.27E-04	7.41 ± 0.31 E-04	15.9	15.6 ± 0.7
2	7.60E-04		15.2	
3	7.37E-04		15.7	

Figure S26. Fragmentation kinetics of YND-BME **6g** at 80 °C.

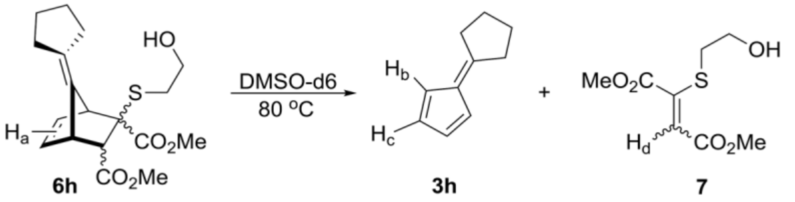

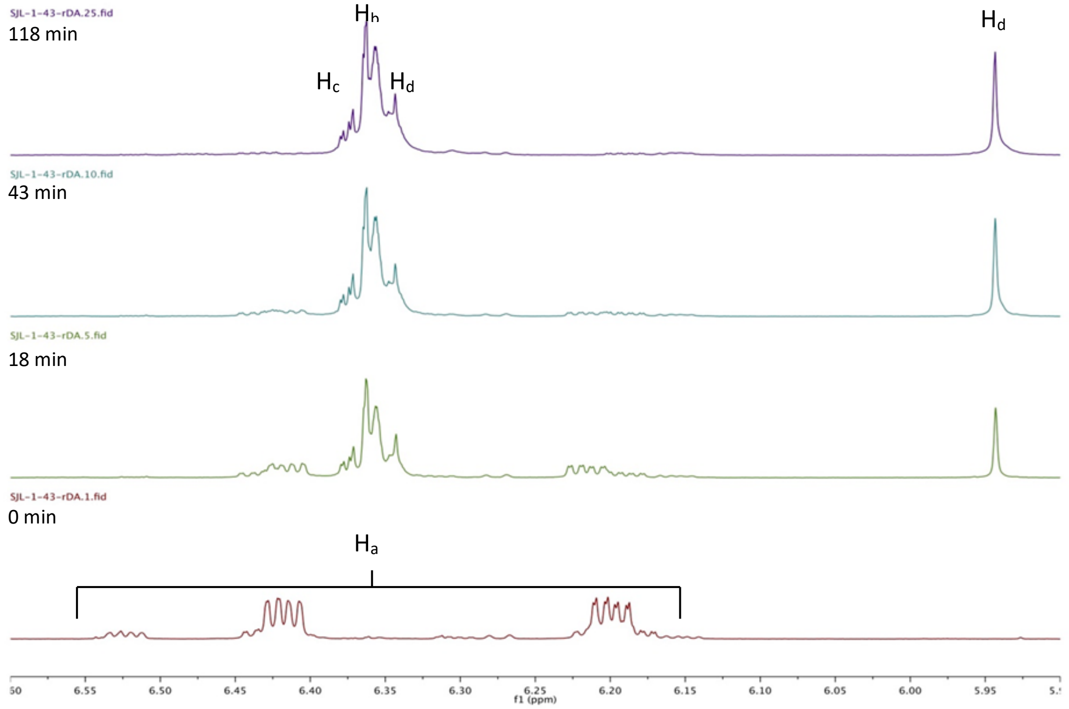

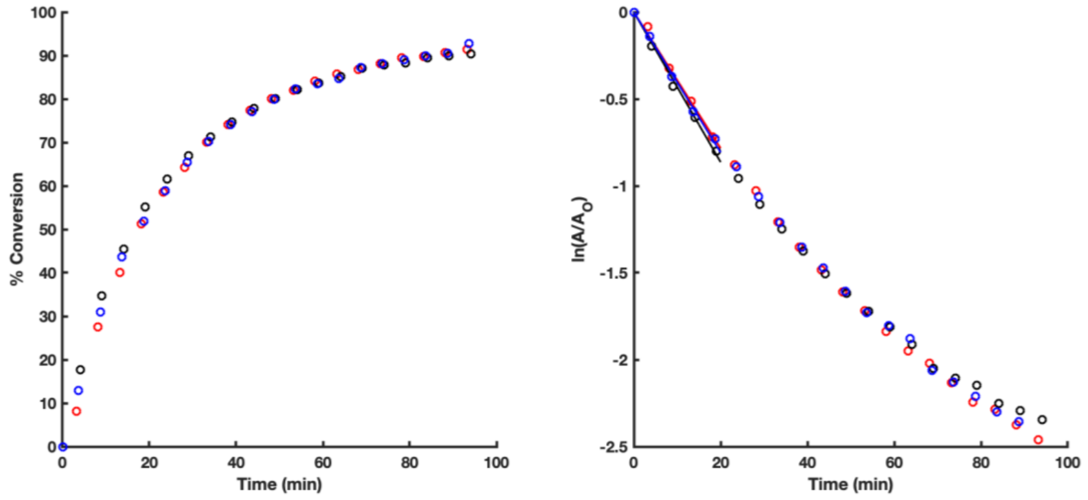

Trial	*k (s^{-1})*	*avg k (s^{-1})*	*t$_{1/2}$ (min)*	*avg t$_{1/2}$ (min)*
1	6.53E-04	6.82 ± 0.85 E-04	17.7	17.0 ± 2.1
2	7.20E-04		16.0	
3	6.73E-04		17.2	

Figure S27. Fragmentation kinetics of YND-BME **6h** at 80 °C.

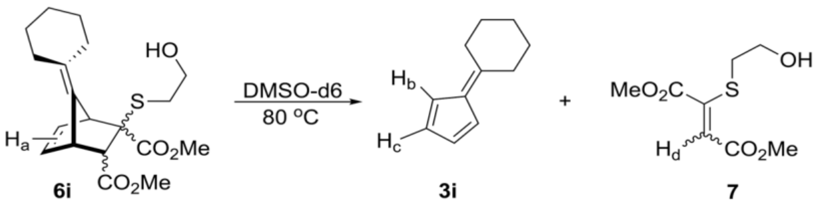

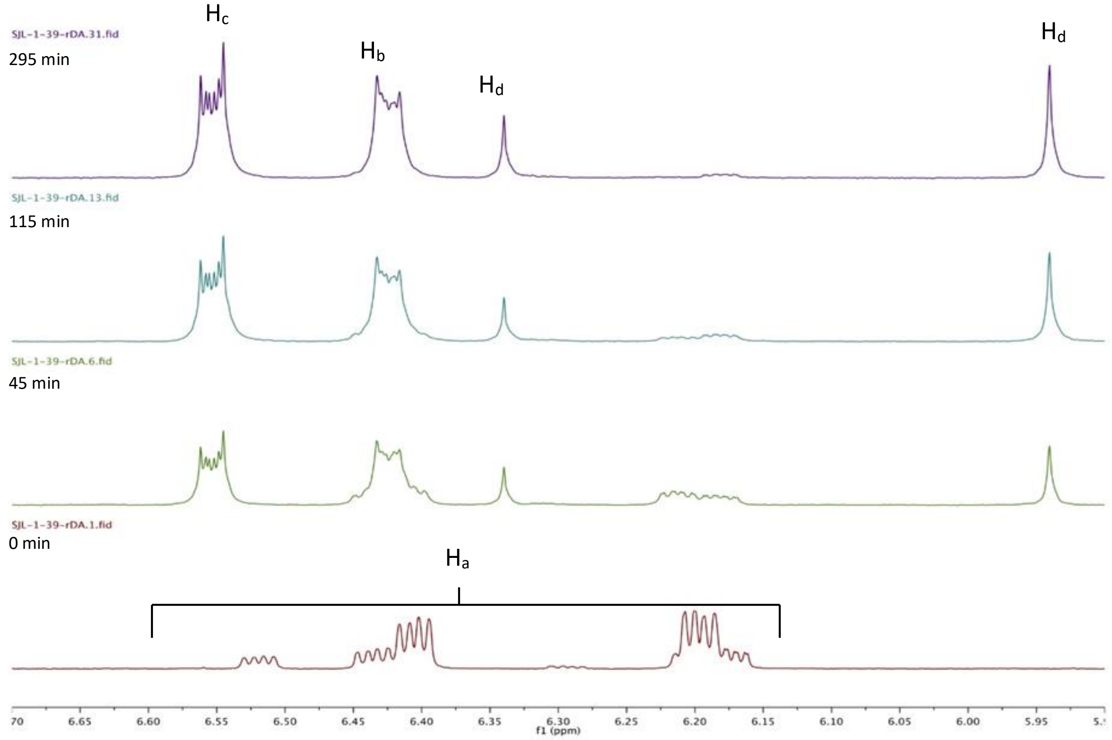

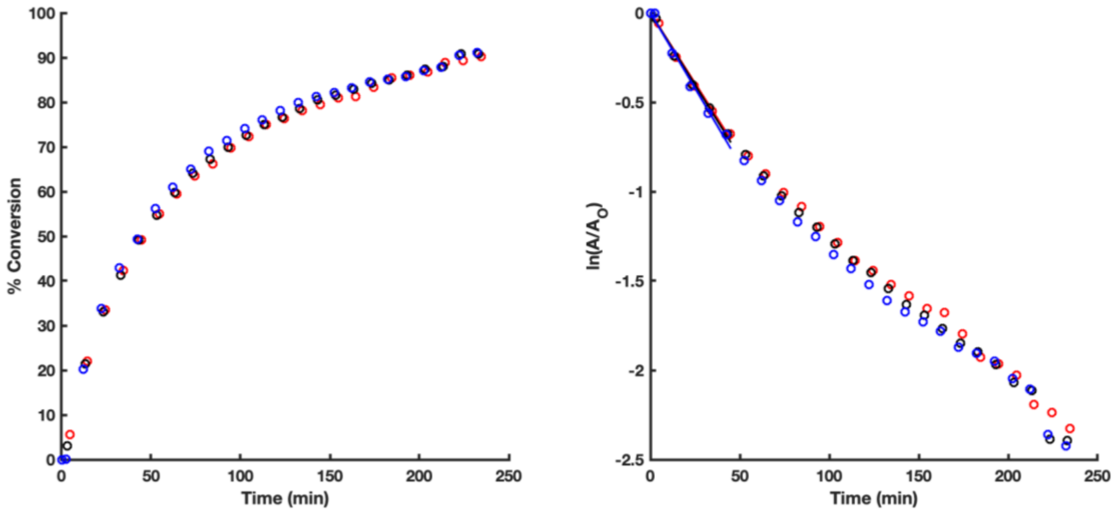

Trial	*k (s^{-1})*	*avg k (s^{-1})*	*t$_{1/2}$ (min)*	*avg t$_{1/2}$ (min)*
1	2.55E-04	2.63 ± 0.24 E-04	45.3	44.0 ± 3.9
2	2.60E-04		44.4	
3	2.73E-04		42.3	

Figure S28. Fragmentation kinetics of YND-BME **6i** at 80 °C.

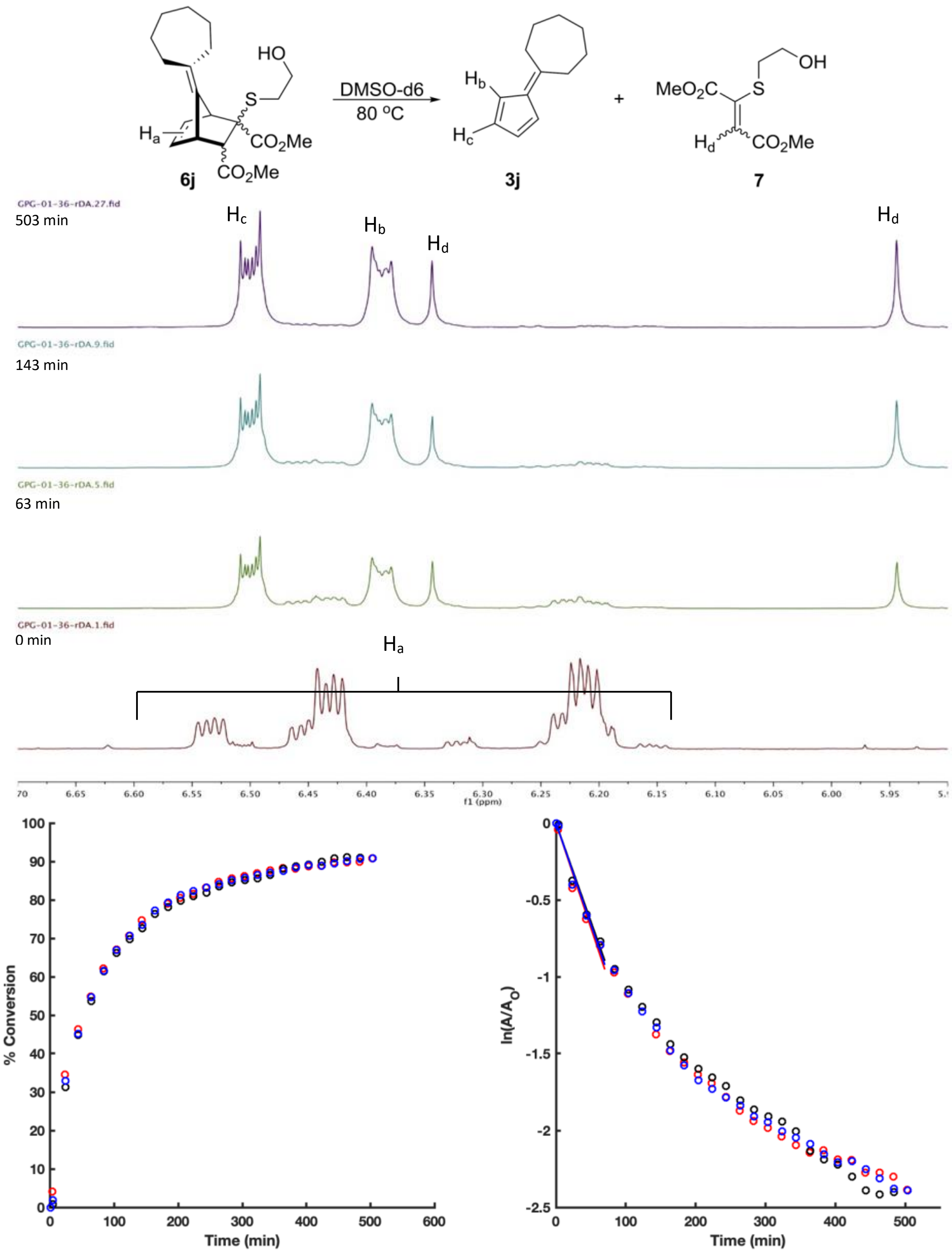

Trial	*k (s^{-1})*	*avg k (s^{-1})*	*$t_{1/2}$ (min)*	*avg $t_{1/2}$ (min)*
1	2.27E-04	2.20 ± 0.17 E-04	51.0	52.5 ± 4.0
2	2.13E-04		54.2	
3	2.20E-04		52.5	

Figure S29. Fragmentation kinetics of YND-BME **6j** at 80 °C.

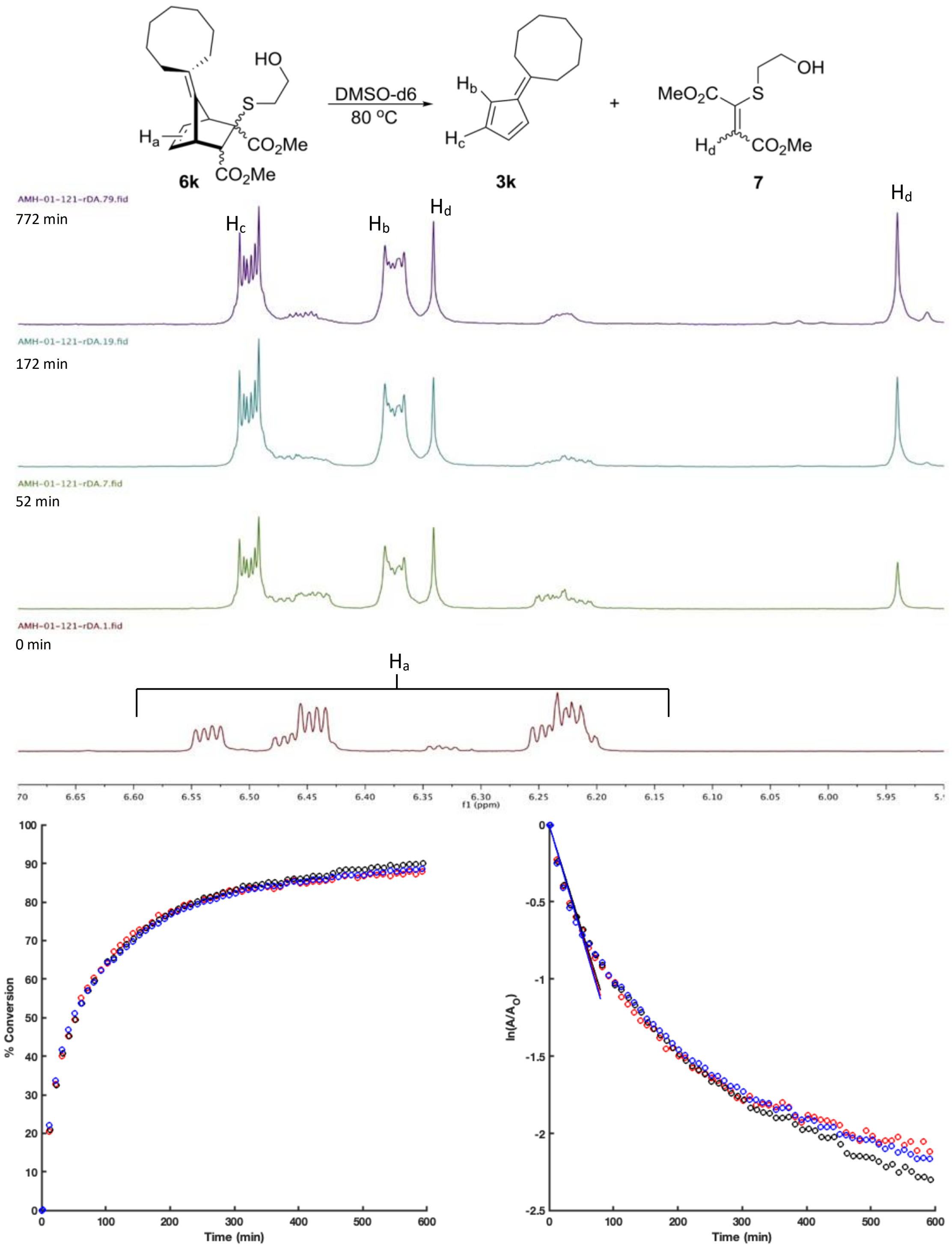

Trial	*k (s^{-1})*	*avg k (s^{-1})*	*$t_{1/2}$ (min)*	*avg $t_{1/2}$ (min)*
1	2.32E-04	2.30 ± 0.15 E-04	49.9	50.3 ± 3.3
2	2.23E-04		51.7	
3	2.35E-04		49.2	

Figure S30. Fragmentation kinetics of YND-BME **6k** at 80 °C.

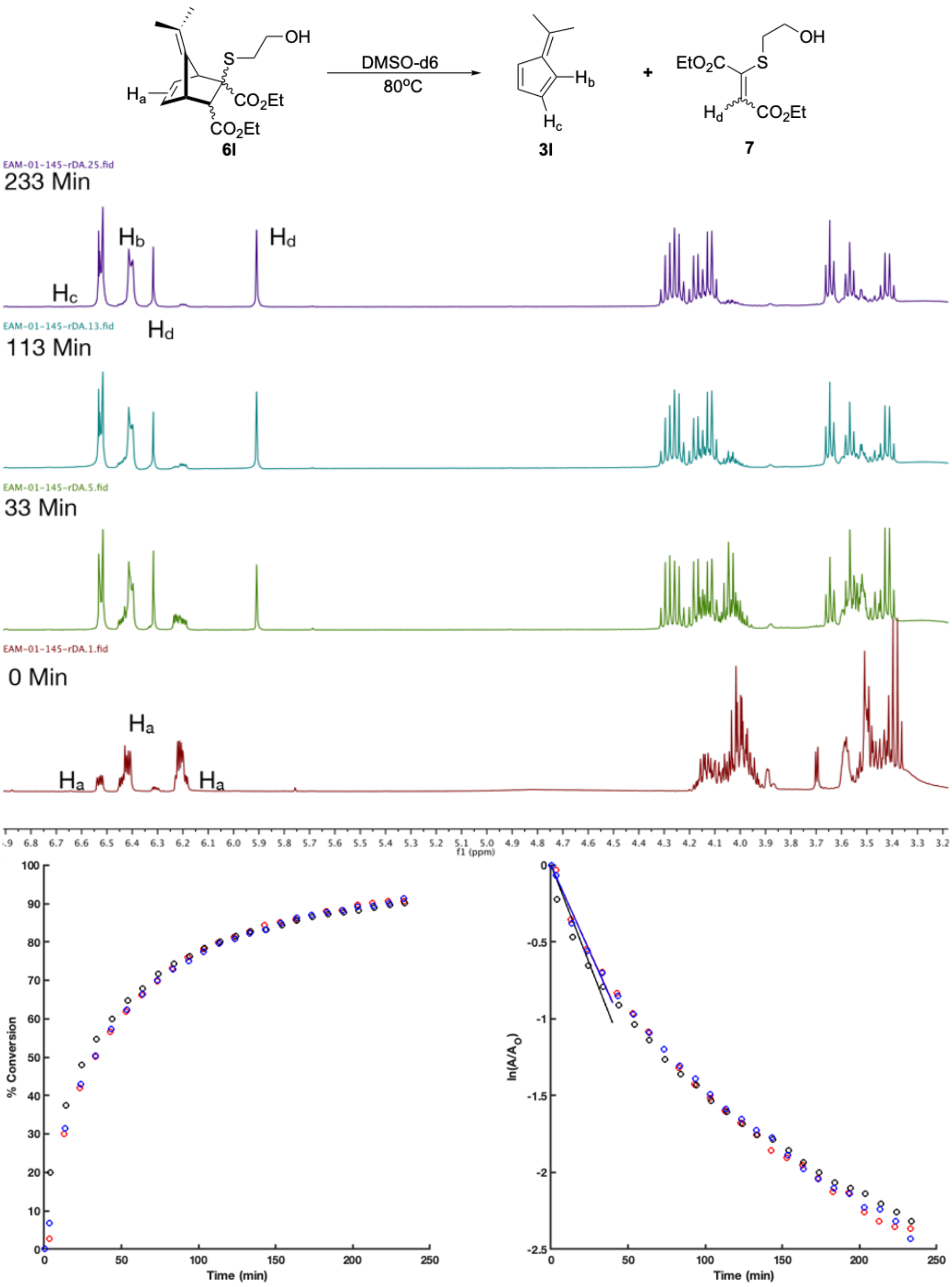

Trial	*k* (s^{-1})	*avg k* (s^{-1})	$t_{1/2}$ *(min)*	*avg* $t_{1/2}$ *(min)*
1	2.55E-04	2.63E ± 0.24E-04	45.3	44.0 ± 3.89
2	2.60E-04		44.4	
3	2.73E-04		42.3	

Figure S31. Fragmentation kinetics of YND-BME **6l** at 80 °C.

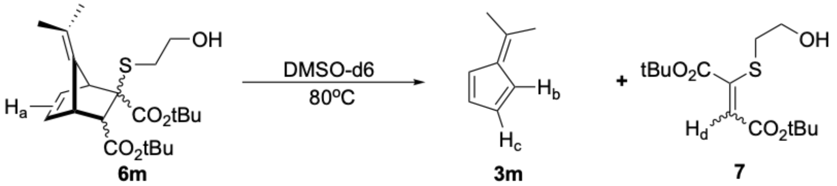

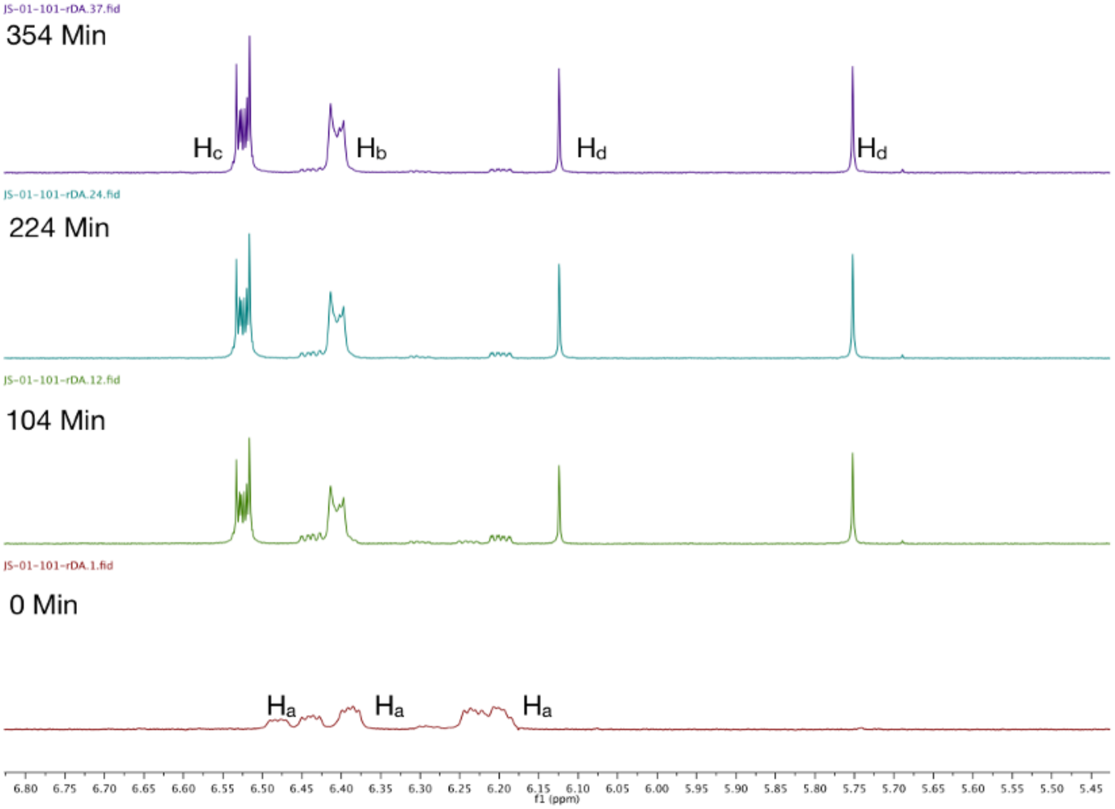

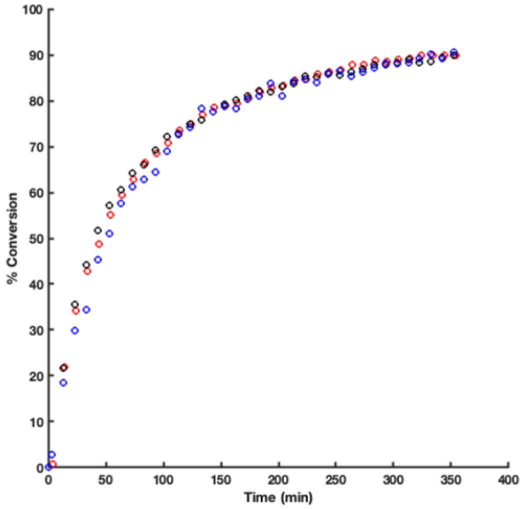

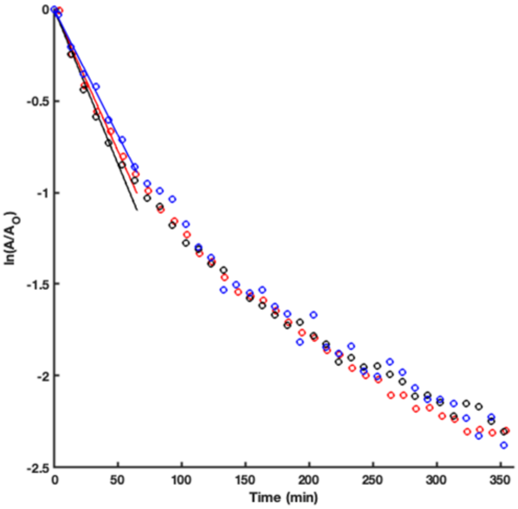

Trial	*k (s^{-1})*	*avg k (s^{-1})*	*$t_{1/2}$ (min)*	*avg $t_{1/2}$ (min)*
1	2.28E-04	2.56 ± 0.66 E-04	50.6	45.4 ± 12.0
2	2.58E-04		44.7	
3	2.82E-04		41.0	

Figure S32. Fragmentation kinetics of YND-BME **6m** at 80 °C.

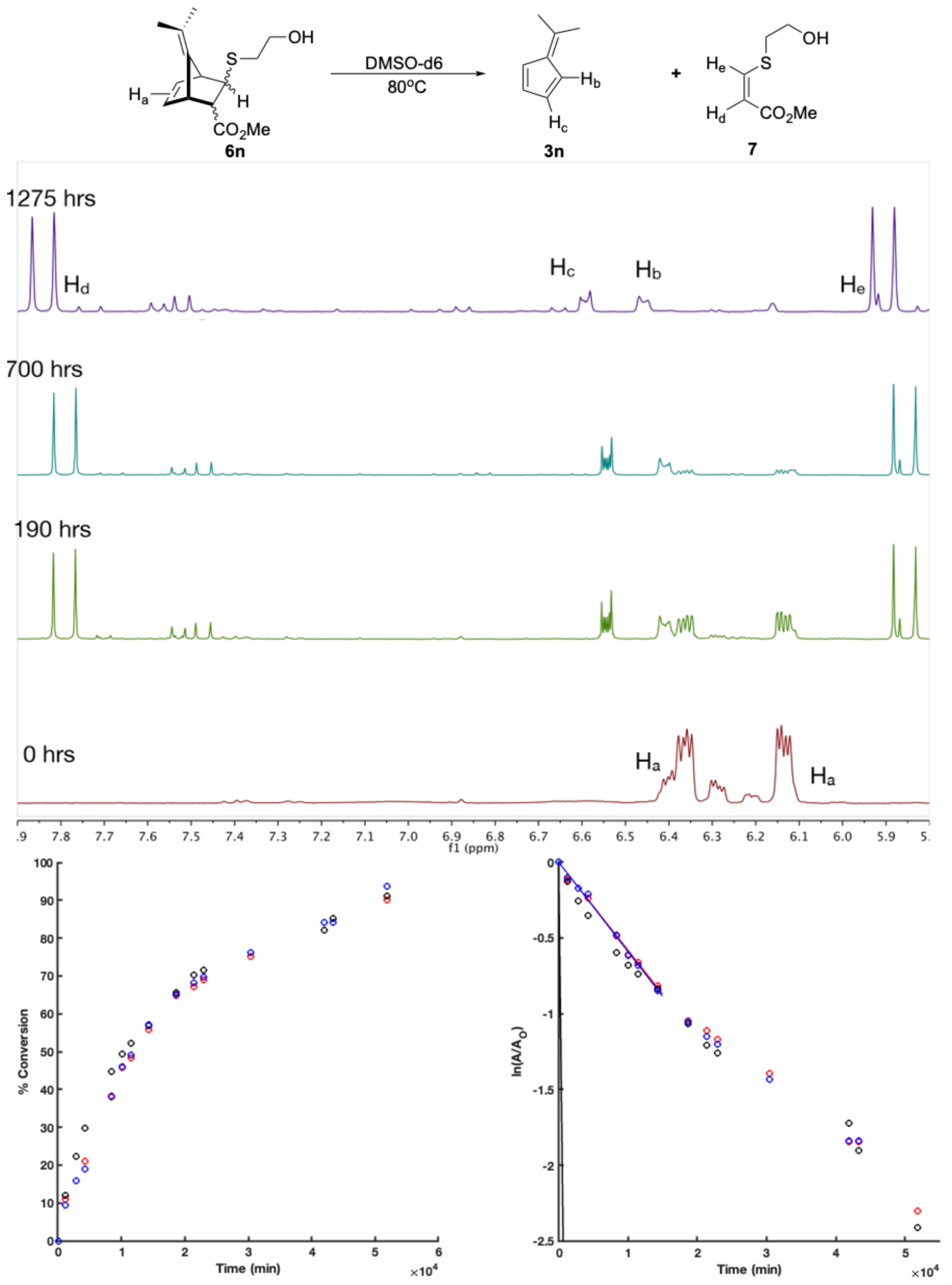

Trial	*k (s⁻¹)*	*avg k (s⁻¹)*	*t1/2 (min)*	*avg t1/2 (min)*
1	9.92E-07	1.05E ± 0.29 E-06	11649.5	11045.8543 ± 2812.70841
2	1.19E-06		9739.8	
3	9.83E-07		11748.3	

Figure S33. Fragmentation kinetics of YND-BME **6n** at 80 °C.

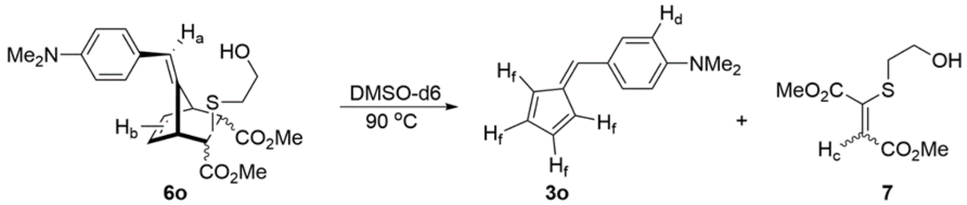

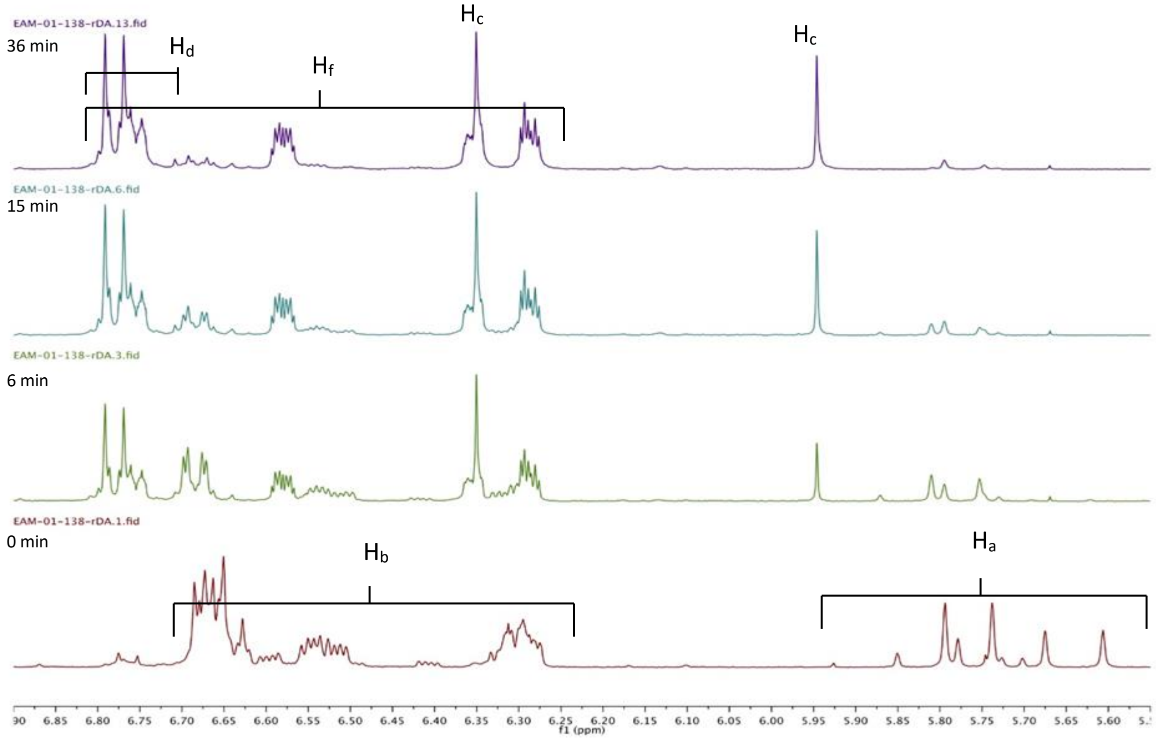

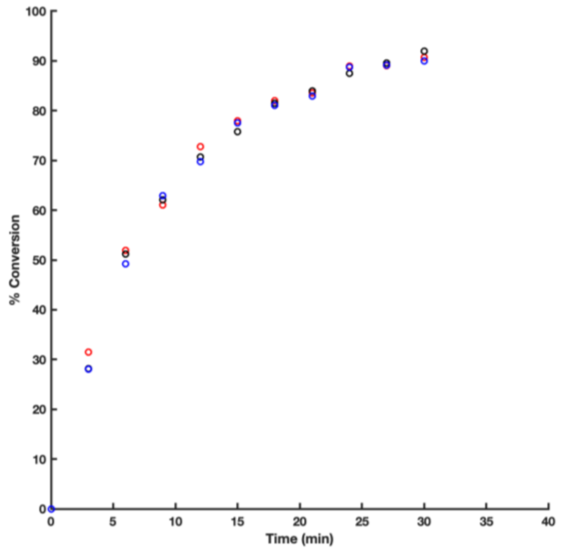

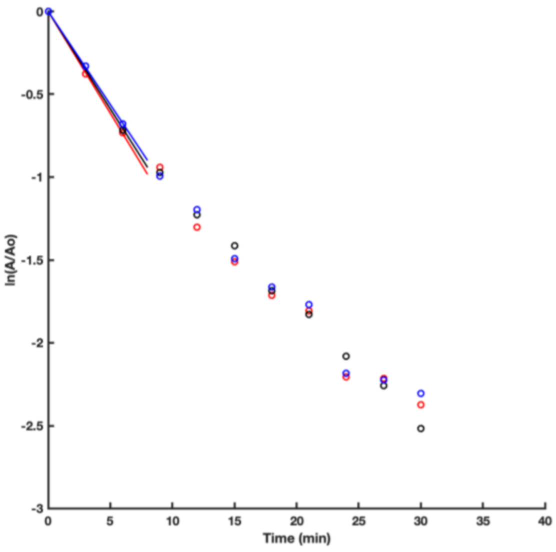

Trial	*k (s^{-1})*	*avg k (s^{-1})*	*$t_{1/2}$ (min)*	*avg $t_{1/2}$ (min)*
1	2.05E-03	1.96 ± 0.22 E-03	5.64	5.90 ± 0.66
2	1.96E-03		5.89	
3	1.87E-03		6.17	

Figure S34. Fragmentation kinetics of YND-BME **6o** at 90 °C.

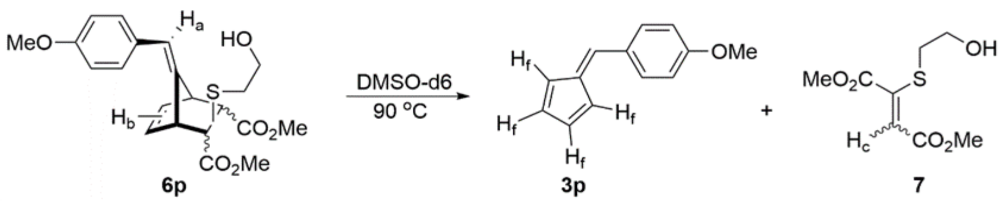

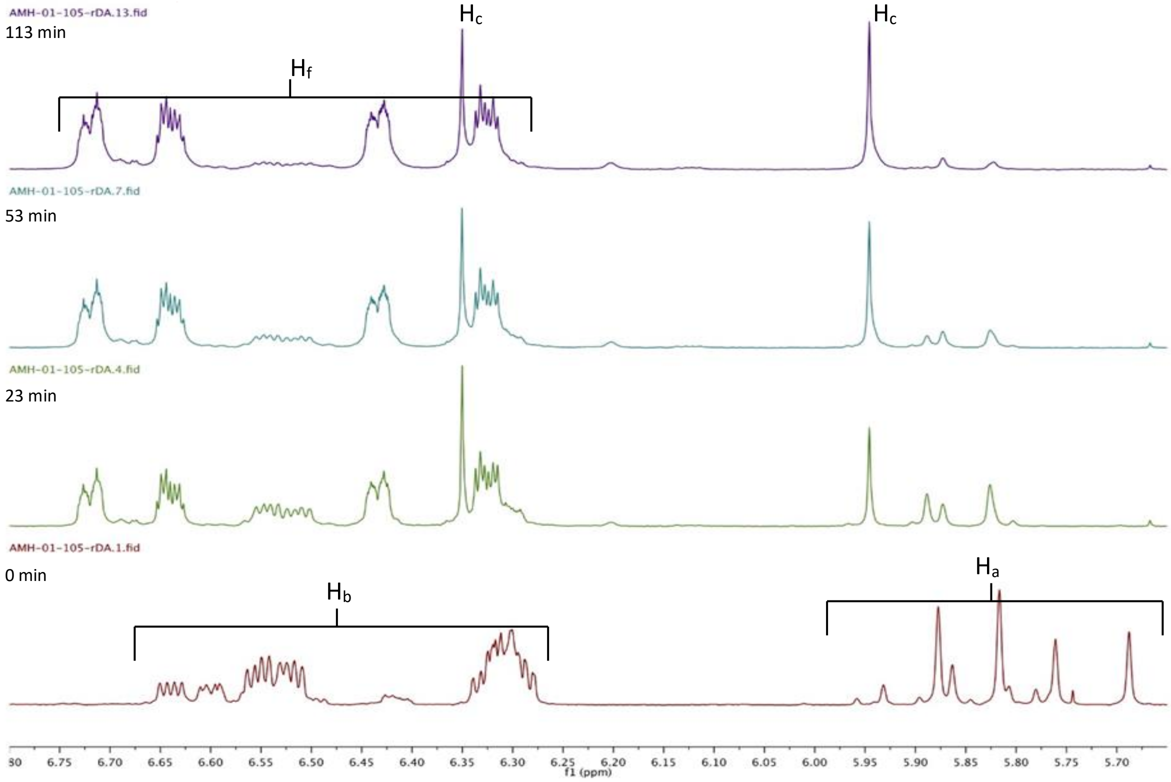

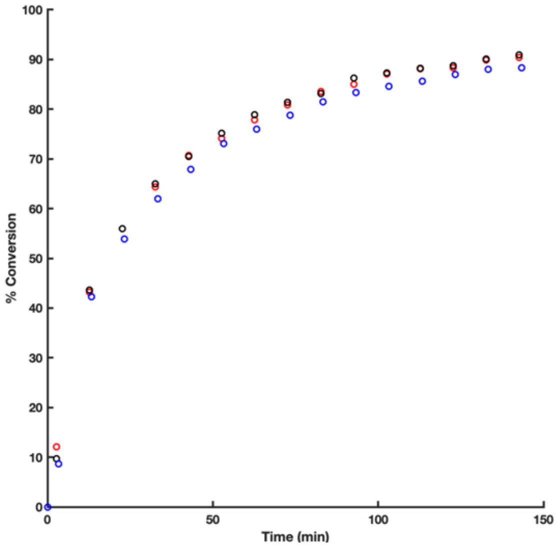

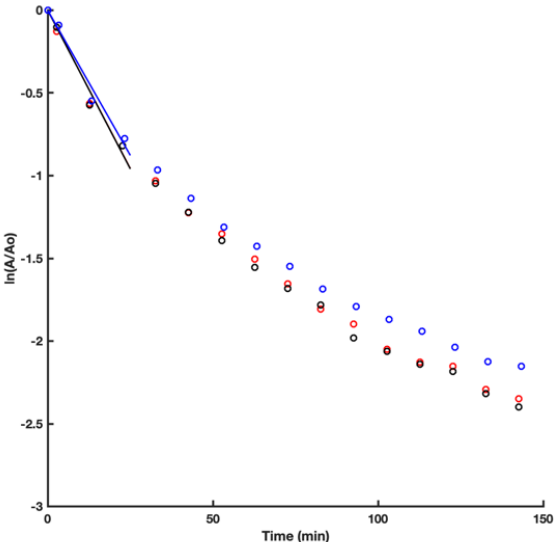

Trial	*k (s^{-1})*	*avg k (s^{-1})*	*$t_{1/2}$ (min)*	*avg $t_{1/2}$ (min)*
1	5.85E-04	6.21 ± 0.58 E-04	19.7	18.6 ± 1.8
2	6.40E-04		18.1	
3	6.38E-04		18.1	

Figure S35. Fragmentation kinetics of YND-BME **6p** at 90 °C.

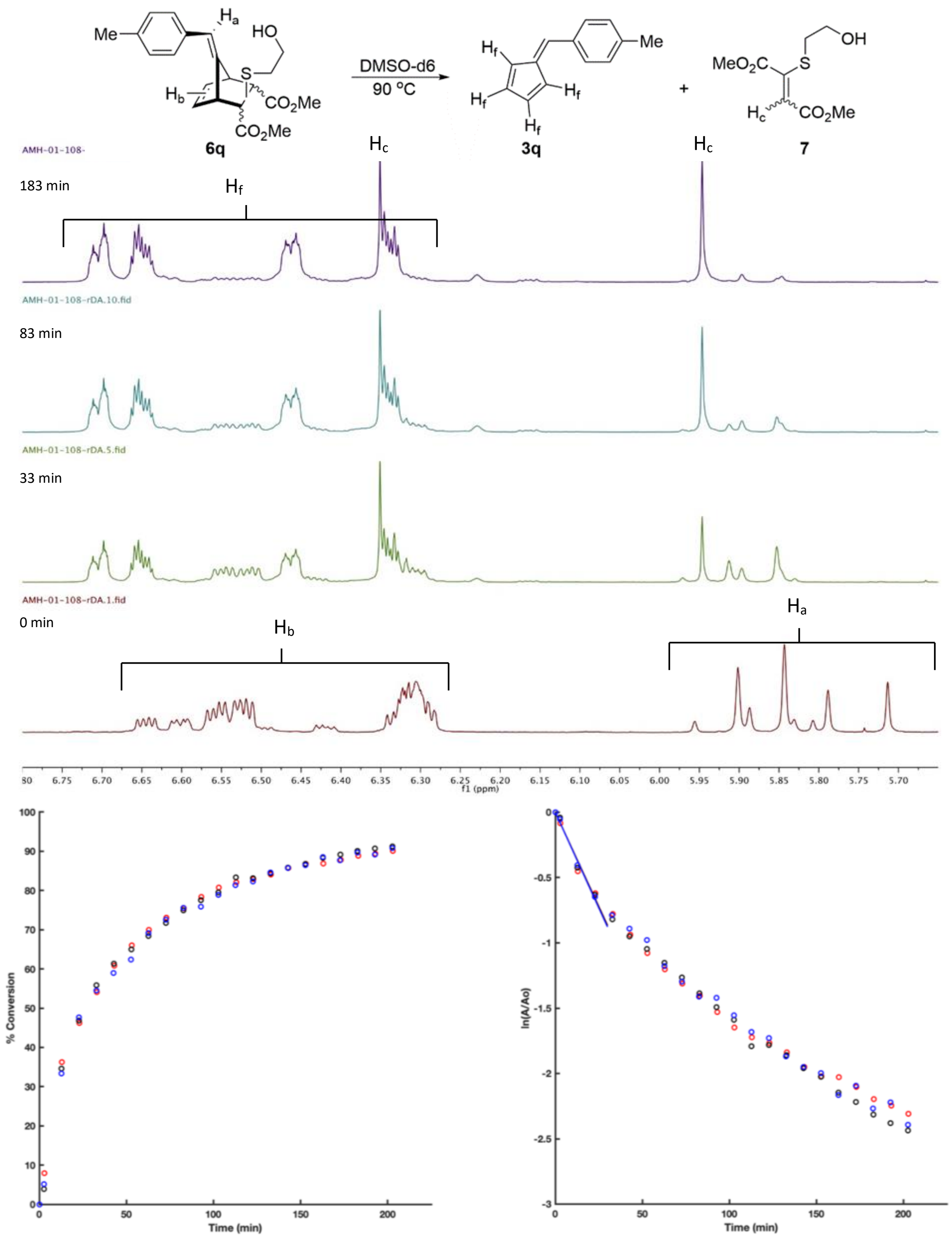

Trial	*k (s⁻¹)*	*avg k (s⁻¹)*	*t1/2 (min)*	*avg t1/2 (min)*
1	4.28E-04	4.35 ± 0.17 E-04	27.0	26.6 ± 1.0
2	4.42E-04		26.2	
3	4.35E-04		26.6	

Figure S36. Fragmentation kinetics of YND-BME **6q** at 90 °C.

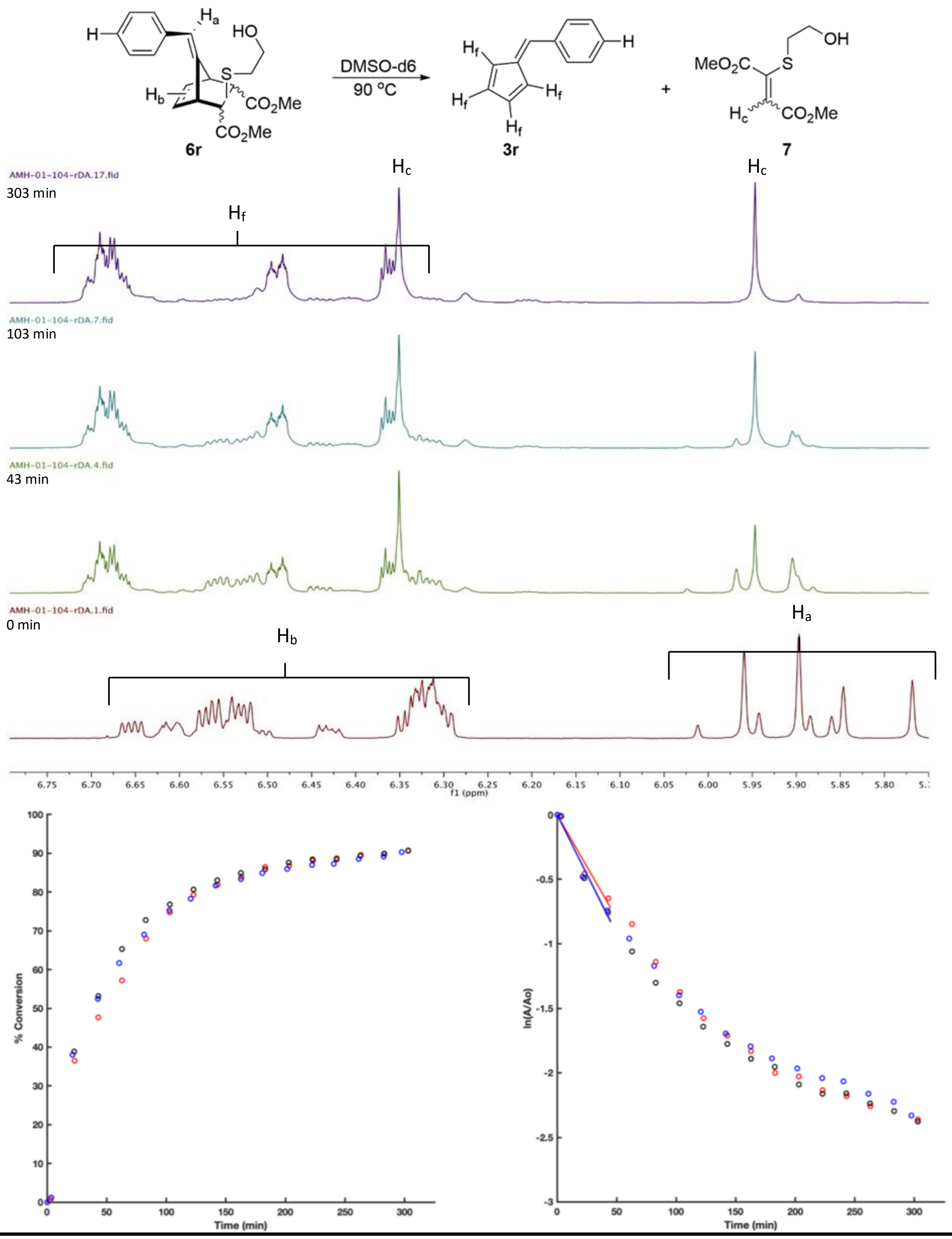

Trial	*k (s^{-1})*	*avg k (s^{-1})*	*$t_{1/2}$ (min)*	*avg $t_{1/2}$ (min)*
1	2.40E-04	2.79 ± 0.84 E-04	48.1	41.9 ± 13.5
2	3.00E-04		38.5	
3	2.97E-04		38.9	

Figure S37. Fragmentation kinetics of YND-BME **6r** at 90 °C.

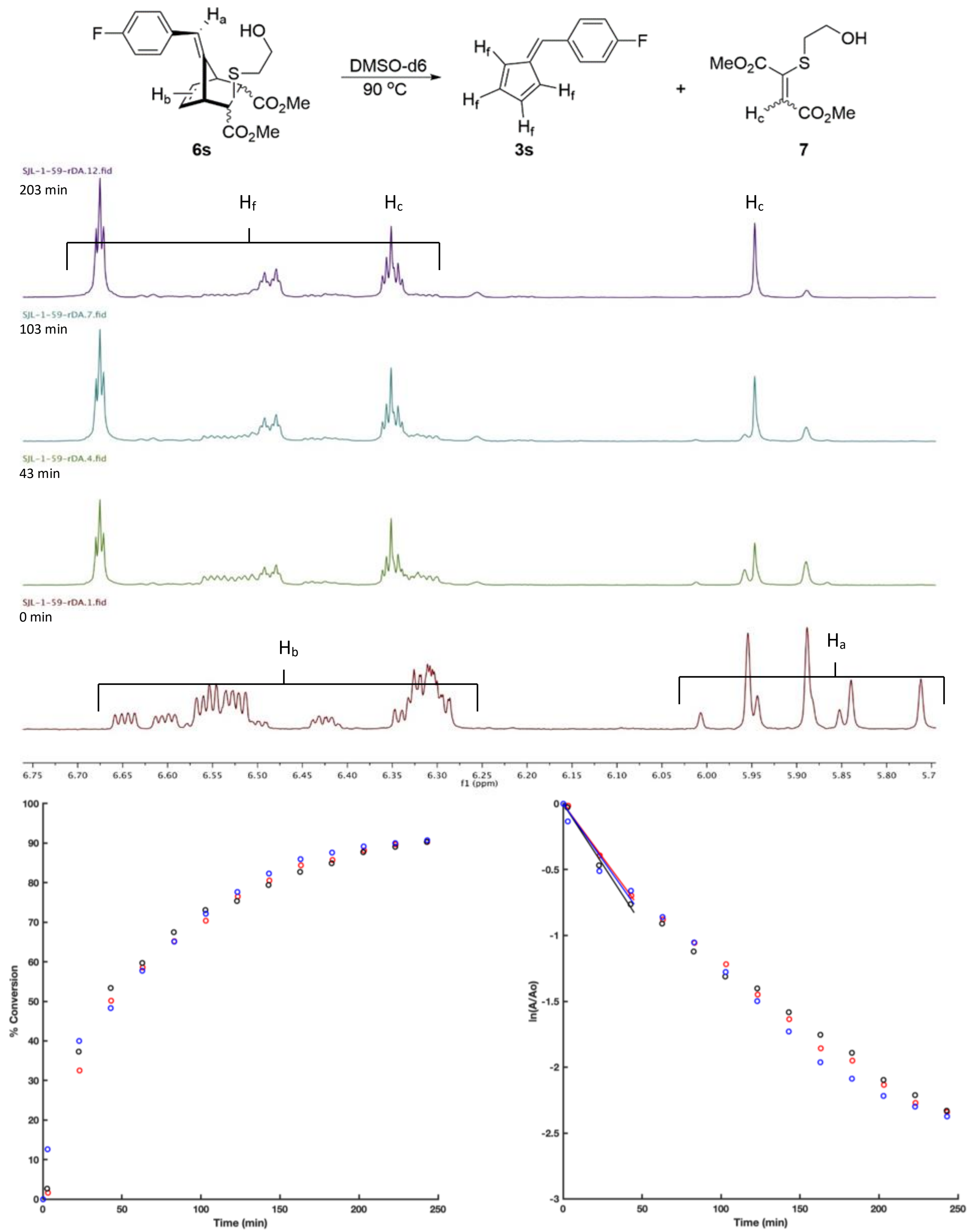

Trial	*k (s⁻¹)*	*avg k (s⁻¹)*	*t1/2 (min)*	*avg t1/2 (min)*
1	2.47E-04	2.53 ± 0.25 E-04	46.8	45.6 ± 4.4
2	2.65E-04		43.6	
3	2.48E-04		46.5	

Figure S38. Fragmentation kinetics of YND-BME **6s** at 90 °C.

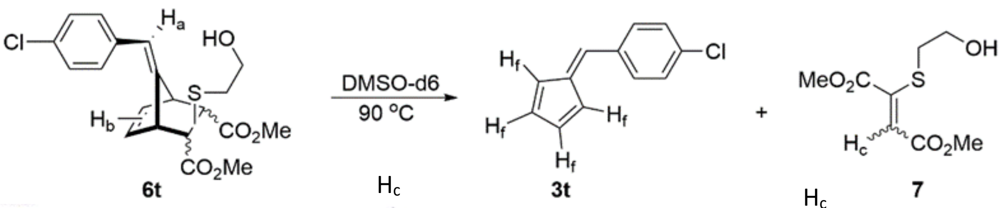

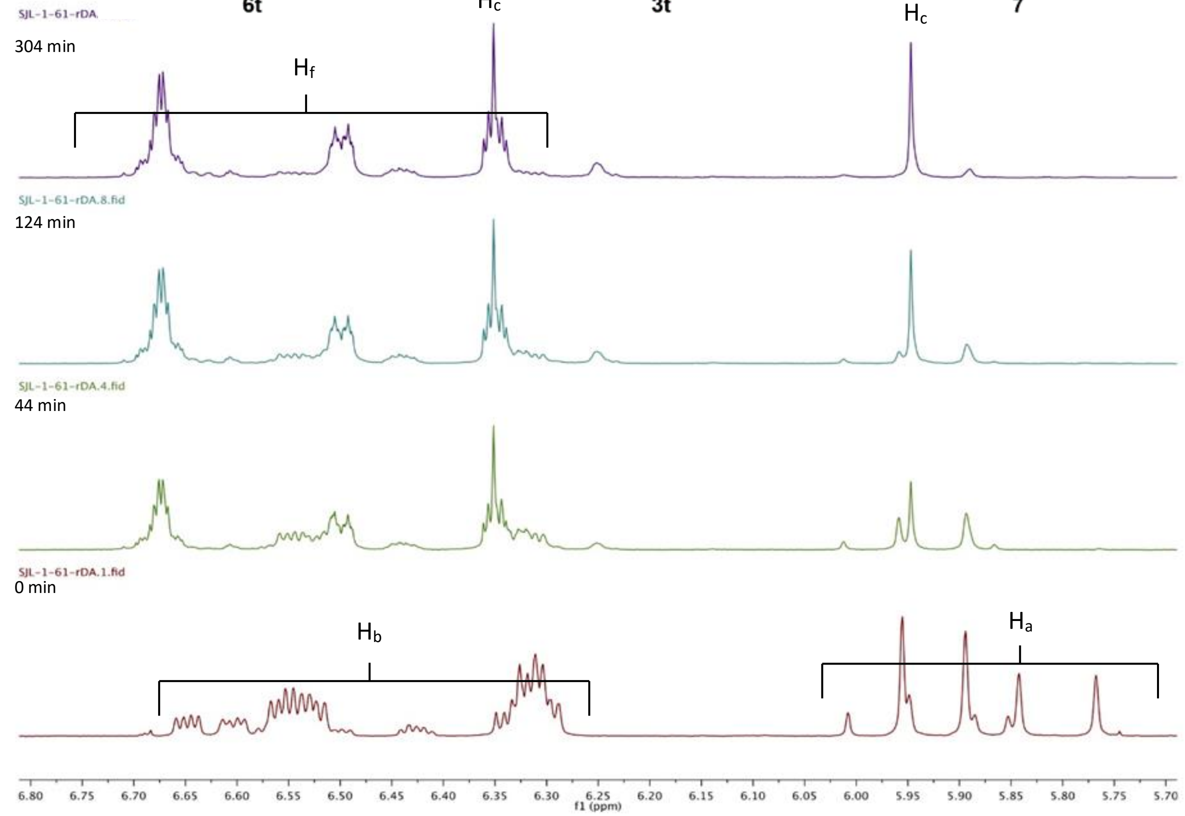

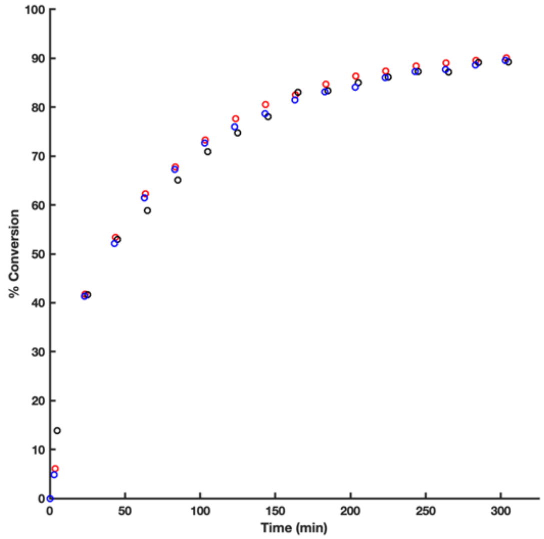

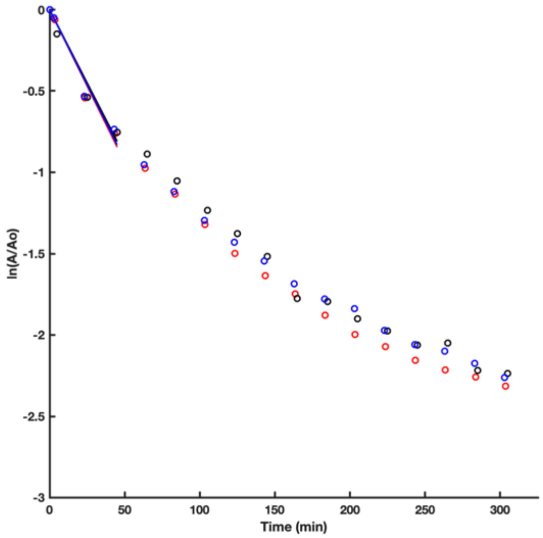

Trial	*k* (s^{-1})	*avg k* (s^{-1})	$t_{1/2}$ *(min)*	*avg* $t_{1/2}$ *(min)*
1	3.13E-04	3.07 ± 0.17 E-04	36.9	37.6 ± 2.1
2	3.00E-04		38.5	
3	3.08E-04		37.5	

Figure S39. Fragmentation kinetics of YND-BME **6t** at 90 °C.

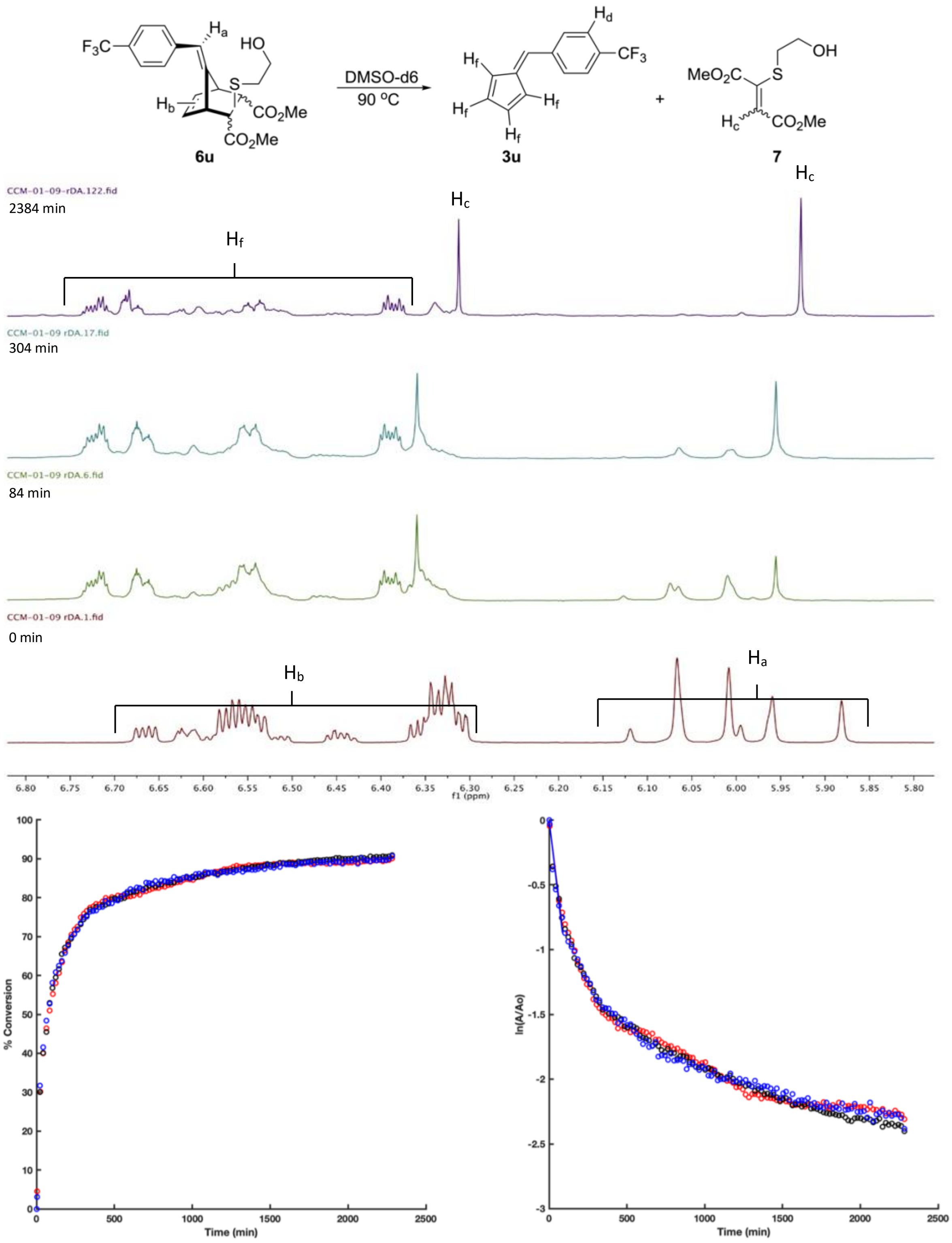

Trial	*k (s^{-1})*	*avg k (s^{-1})*	*$t_{1/2}$ (min)*	*avg $t_{1/2}$ (min)*
1	1.60E-04	1.65 ± 0.12 E-04	72.1	70.1 ± 5.2
2	1.70E-04		68.0	
3	1.65E-04		70.1	

Figure S40. Fragmentation kinetics of YND-BME **6u** at 90 °C.

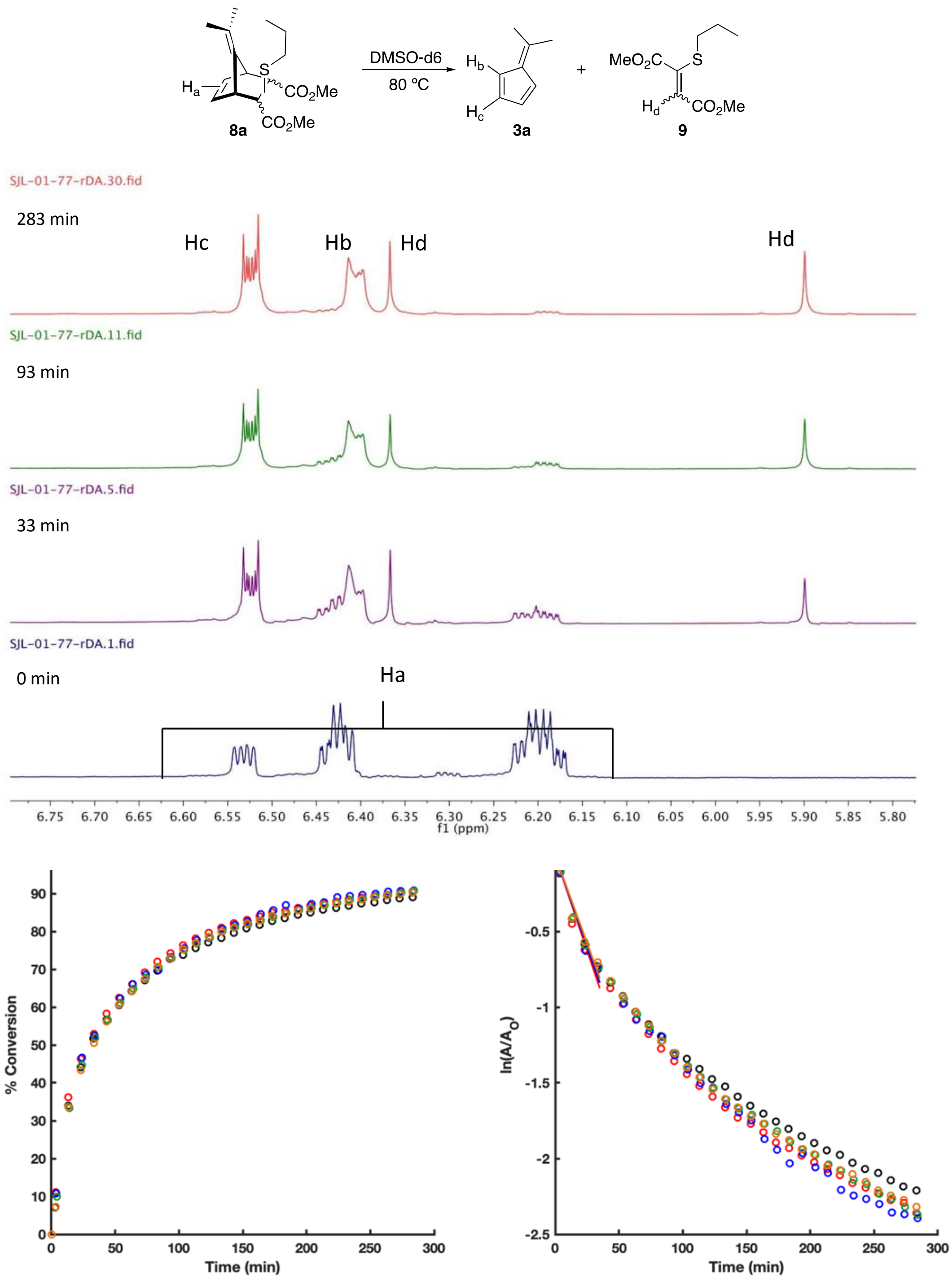

Trial	*k (s⁻¹)*	*avg k (s⁻¹)*	*t$_{1/2}$ (min)*	*avg t$_{1/2}$ (min)*
1	4.17E-04	3.97 ± 0.16 E-04	27.7	29.1 ± 1.1
2	4.00E-04		28.9	
3	3.85E-04		30.0	
4	3.98E-04		29.0	
5	3.85E-04		30.0	

Figure S41. Fragmentation kinetics of YND-PT **8a:d1-3** at 80 °C in DMSO-d6.

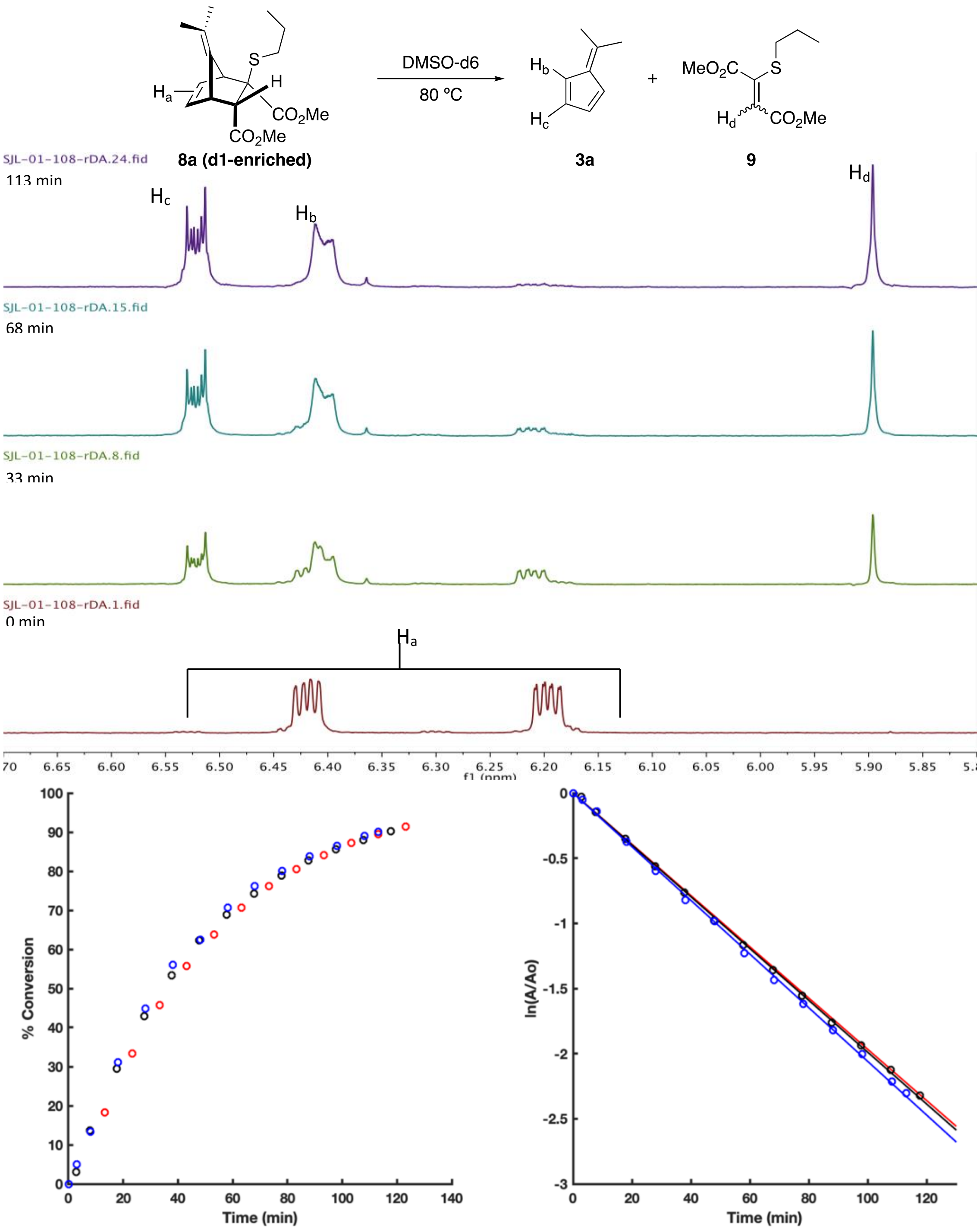

Trial	*k (s^{-1})*	*avg k (s^{-1})*	*$t_{1/2}$ (min)*	*avg $t_{1/2}$ (min)*
1	3.33E-04	3.33 ± 0.25 E-04	34.7	34.7 ± 2.6
2	3.23E-04		35.7	
3	3.43E-04		33.6	

Figure S42. Fragmentation kinetics of YND-PT **8a:d1** at 80 °C in DMSO-d6.

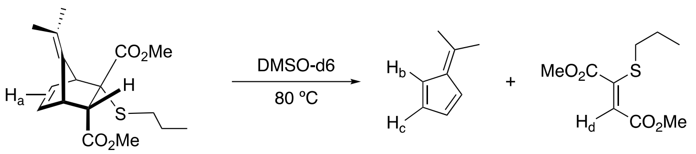

8a (d2-enriched) **3a** **9**

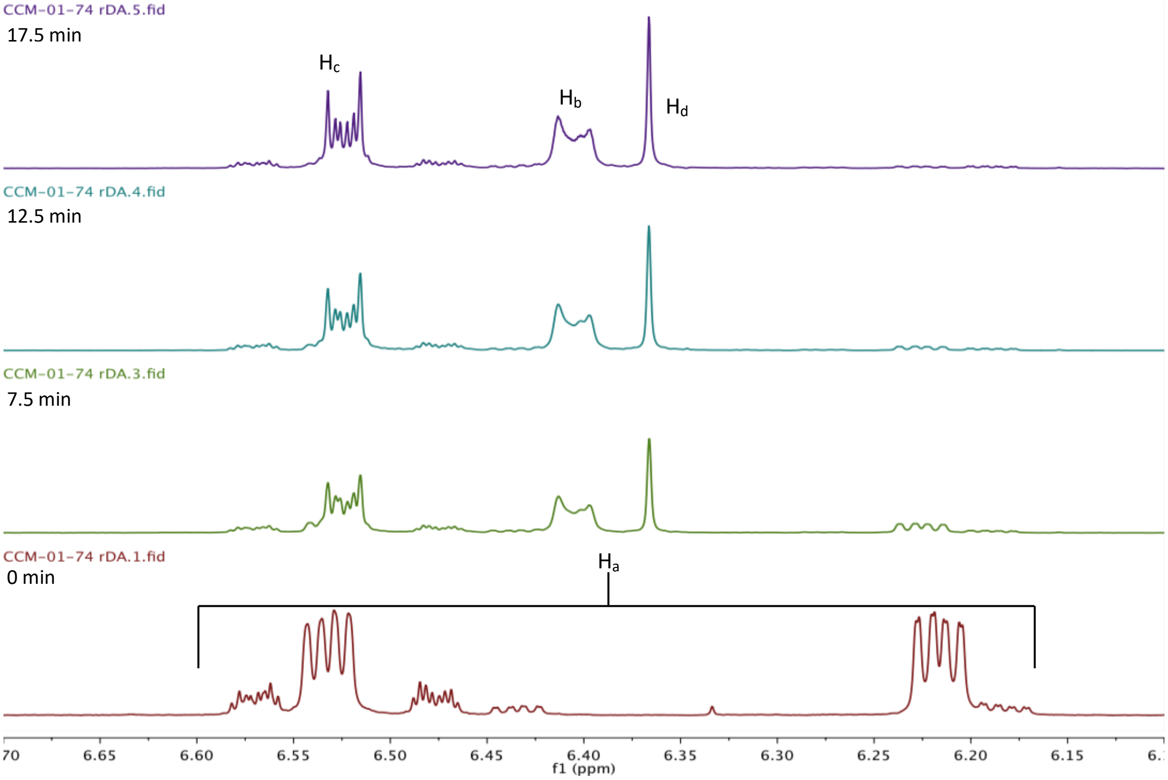

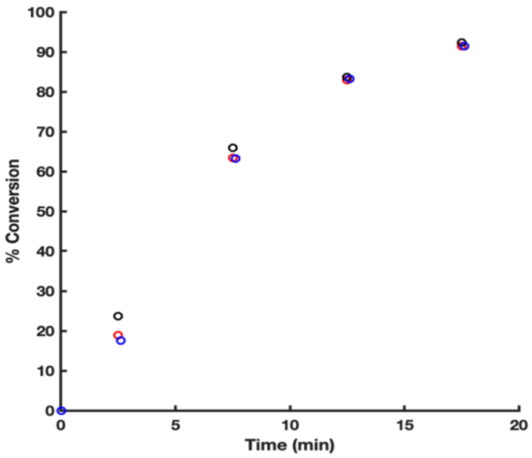

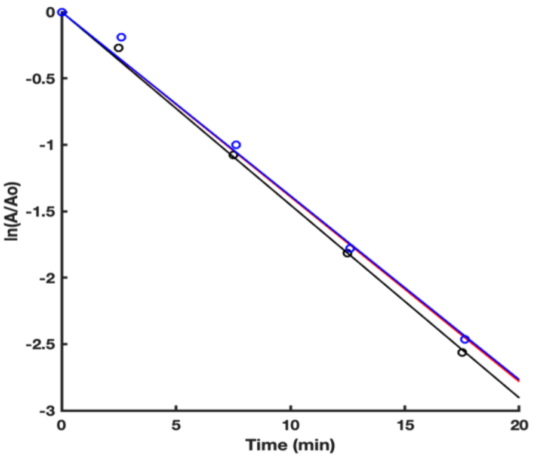

Trial	*k (s^{-1})*	*avg k (s^{-1})*	*t$_{1/2}$ (min)*	*avg t$_{1/2}$ (min)*
1	2.33E-03	2.33 ± 0.15 E-03	4.95	5.0 ± .31
2	2.38E-03		4.85	
3	2.27E-03		5.10	

Figure S43. Fragmentation kinetics of YND-PT **8a:d2** at 80 °C in DMSO-d6.

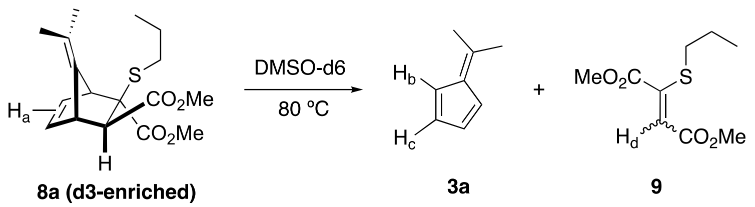

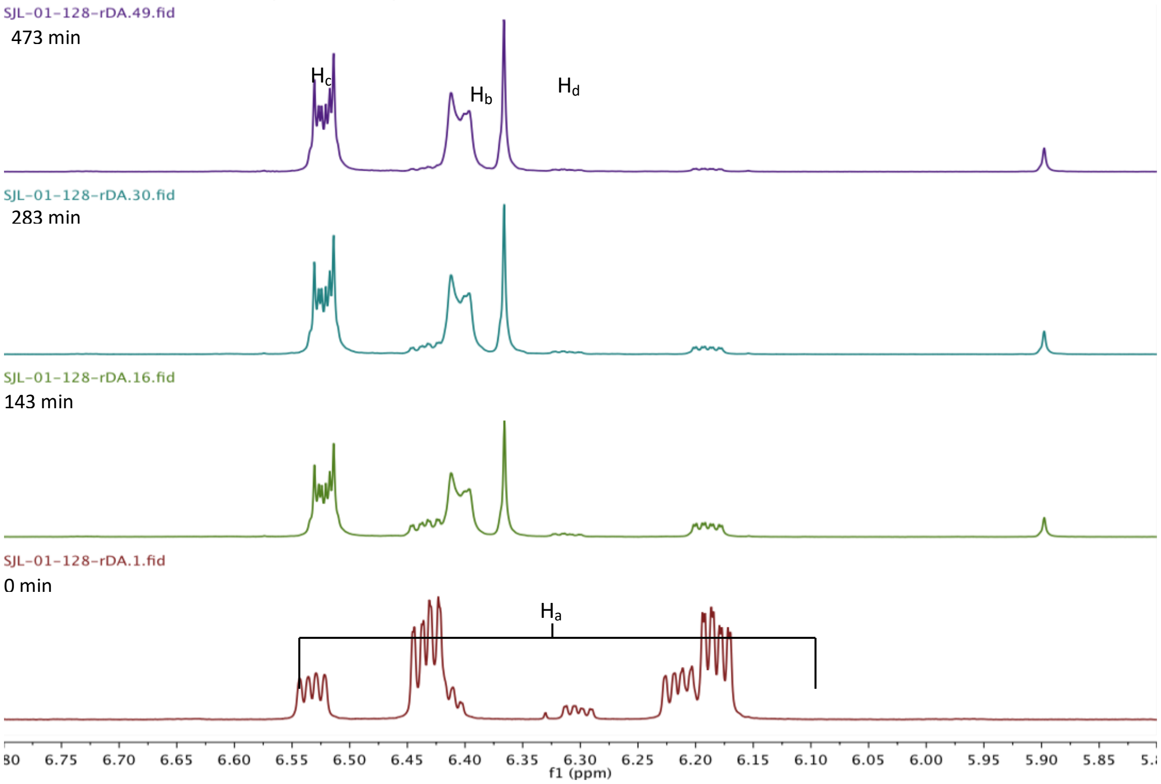

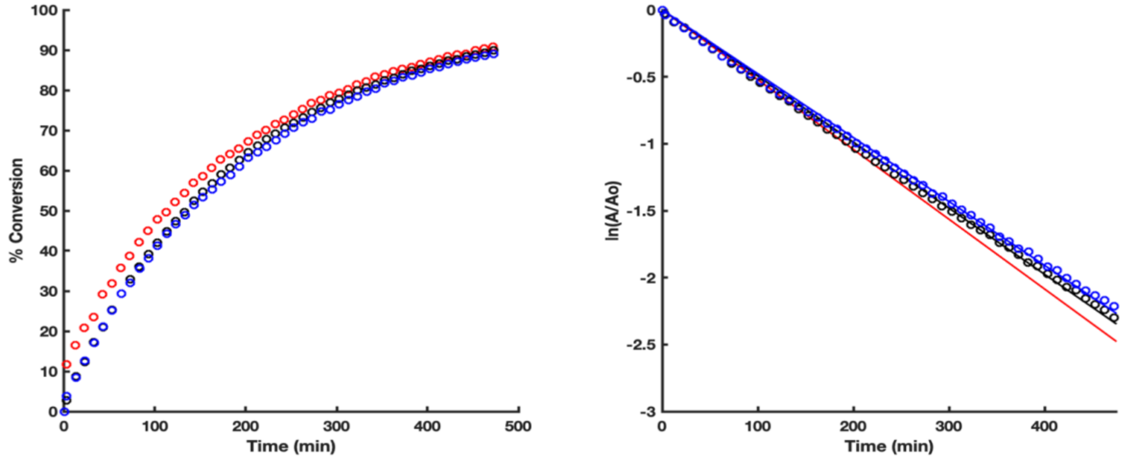

Trial	*k (s⁻¹)*	*avg k (s⁻¹)*	*$t_{1/2}$ (min)*	*avg $t_{1/2}$ (min)*
1	8.78E-05	8.33 ± 1.02 E-05	131.5	139 ± 17
2	8.23E-05		140.3	
3	7.98E-05		144.7	

Figure S44. Fragmentation kinetics of YND-PT **8a:d3** at 80 °C in DMSO-d6.

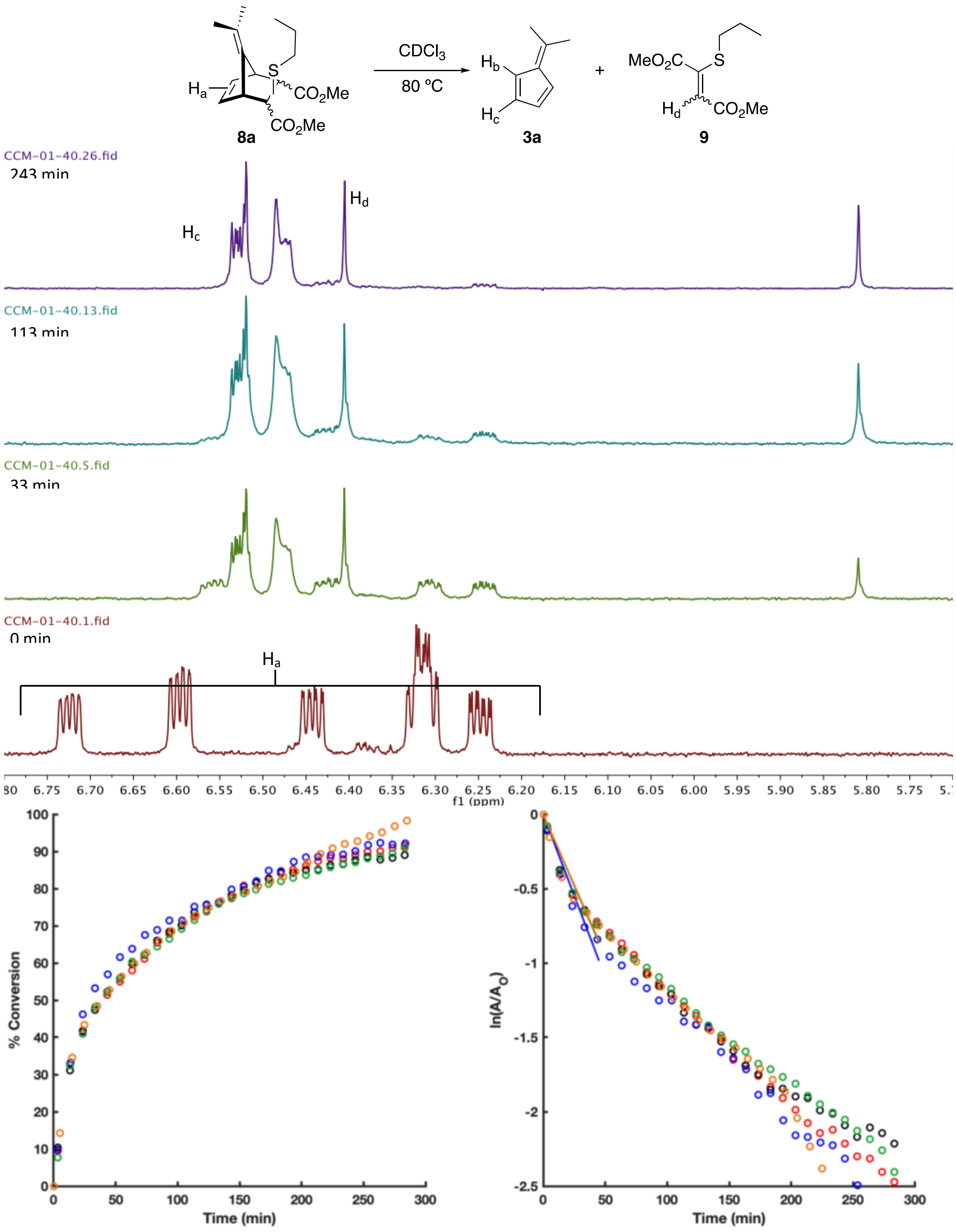

Trial	*k (s^{-1})*	*avg k (s^{-1})*	*$t_{1/2}$ (min)*	*avg $t_{1/2}$ (min)*
1	3.18E-04	3.28 ± 0.26 E-04	36.3	35.3 ± 2.6
2	3.65E-04		31.7	
3	3.20E-04		36.1	
4	3.17E-04		36.5	
5	3.20E-04		36.1	

Figure S45. Fragmentation kinetics of YND-PT **8a:d1-3** at 80 °C in $CDCl_3$.

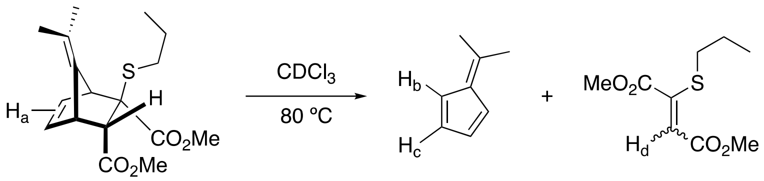

8a (d1-enriched) **3a** **9**

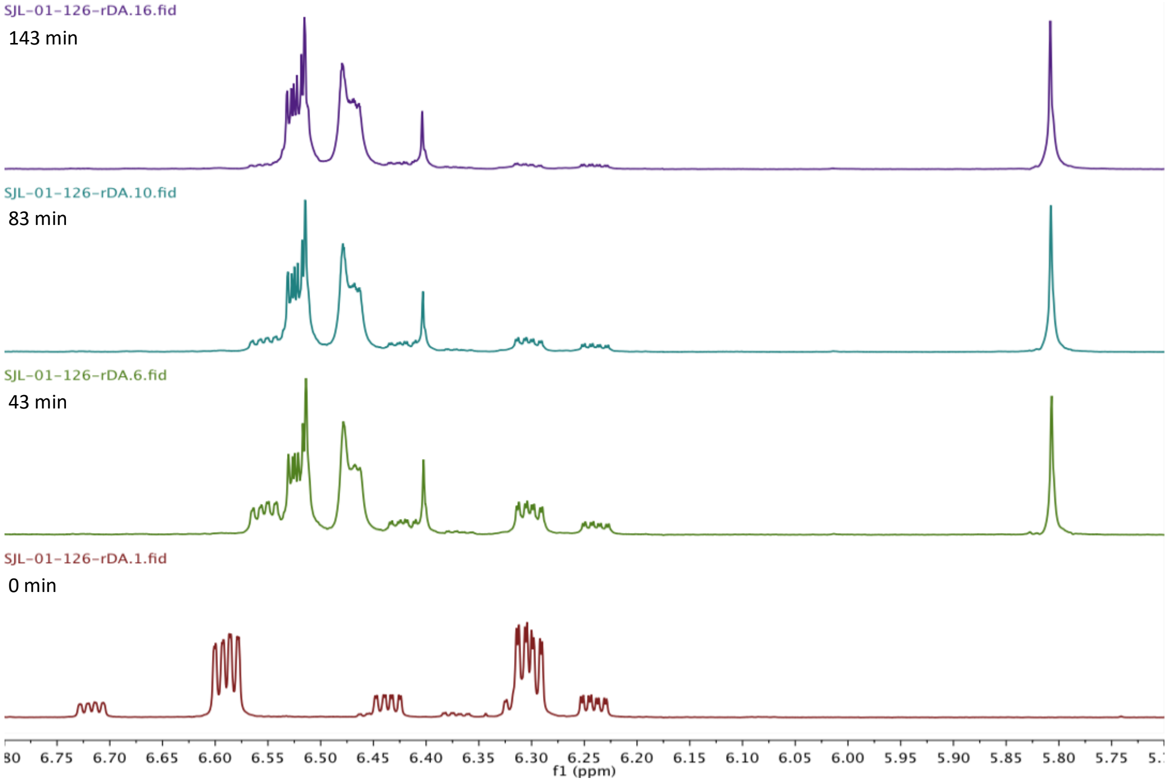

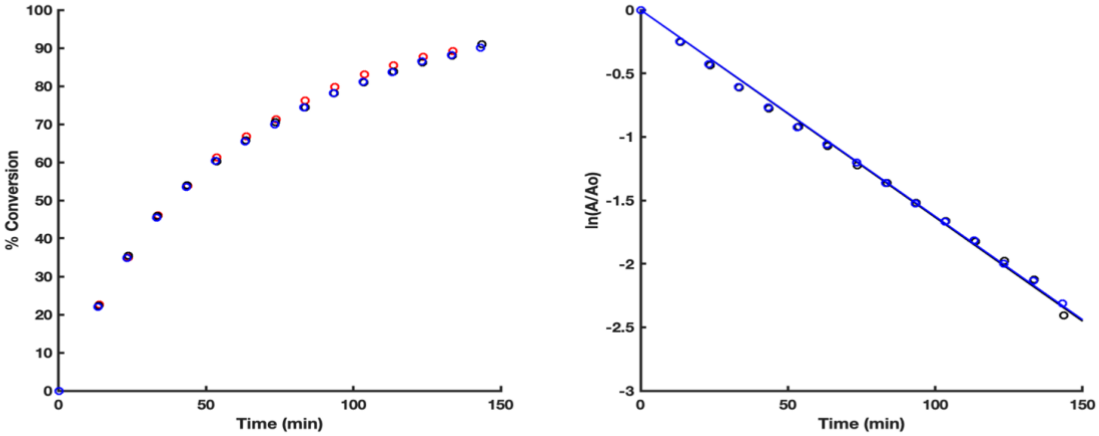

Trial	*k (s⁻¹)*	*avg k (s⁻¹)*	*t1/2 (min)*	*avg t1/2 (min)*
1	2.82E-04	2.75 ± 0.14 E-04	41.0	42.0 ± 2.2
2	2.72E-04		42.5	
3	2.72E-04		42.5	

Figure S46. Fragmentation kinetics of YND-PT **8a:d1** at 80 °C in $CDCl_3$.

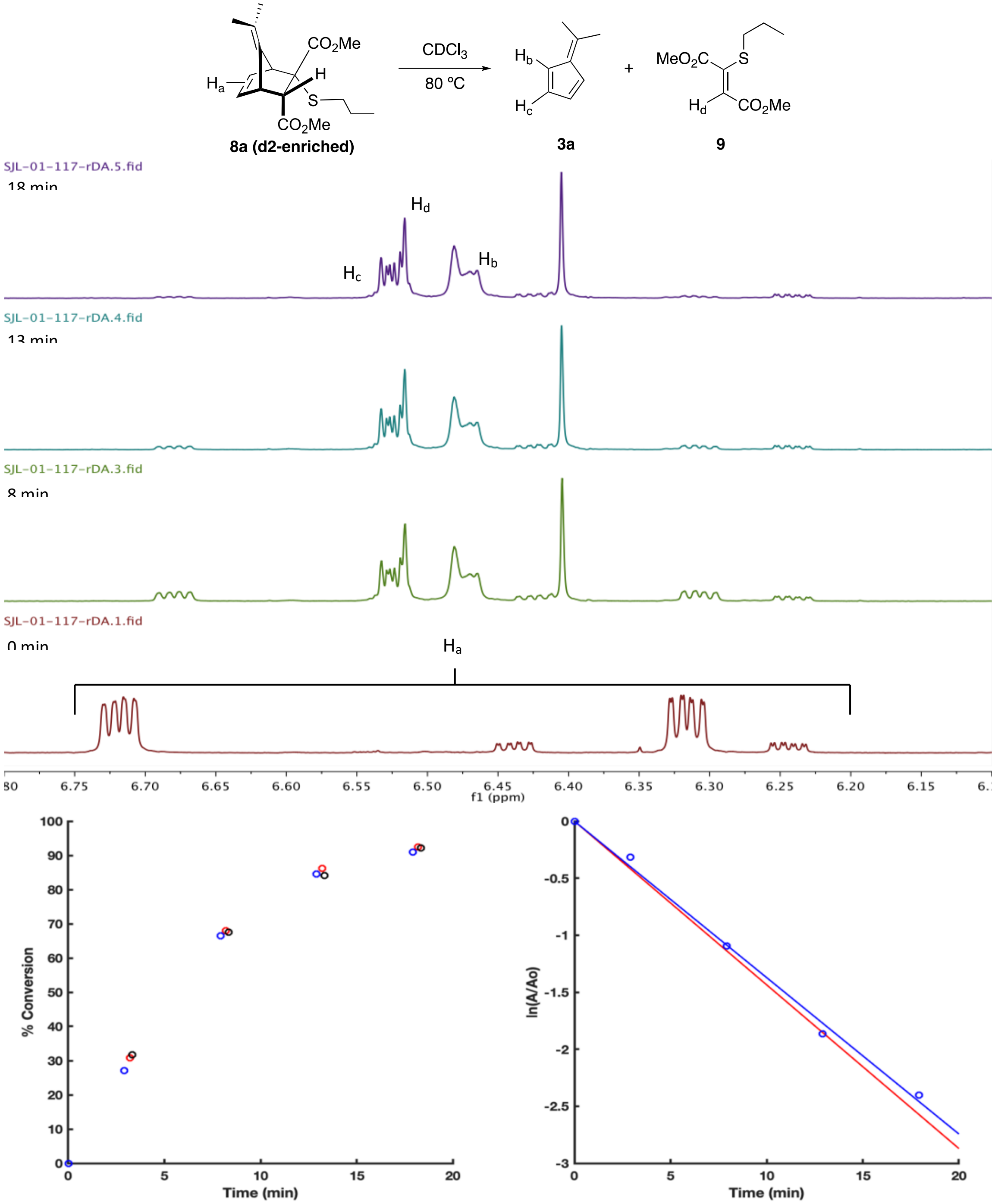

Trial	*k (s⁻¹)*	*avg k (s⁻¹)*	*t1/2 (min)*	*avg t1/2 (min)*
1	2.33E-03	2.30 ± 0.07 E-03	4.95	5.0 ± 0.2
2	2.28E-03		5.06	
3	2.28E-03		5.06	

Figure S47. Fragmentation kinetics of YND-PT **8a:d2** at 80 °C in $CDCl_3$.

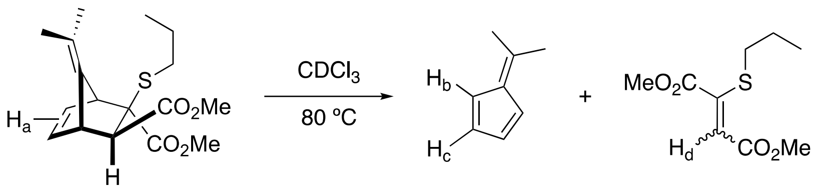

8a (d3-enriched) 3a 9

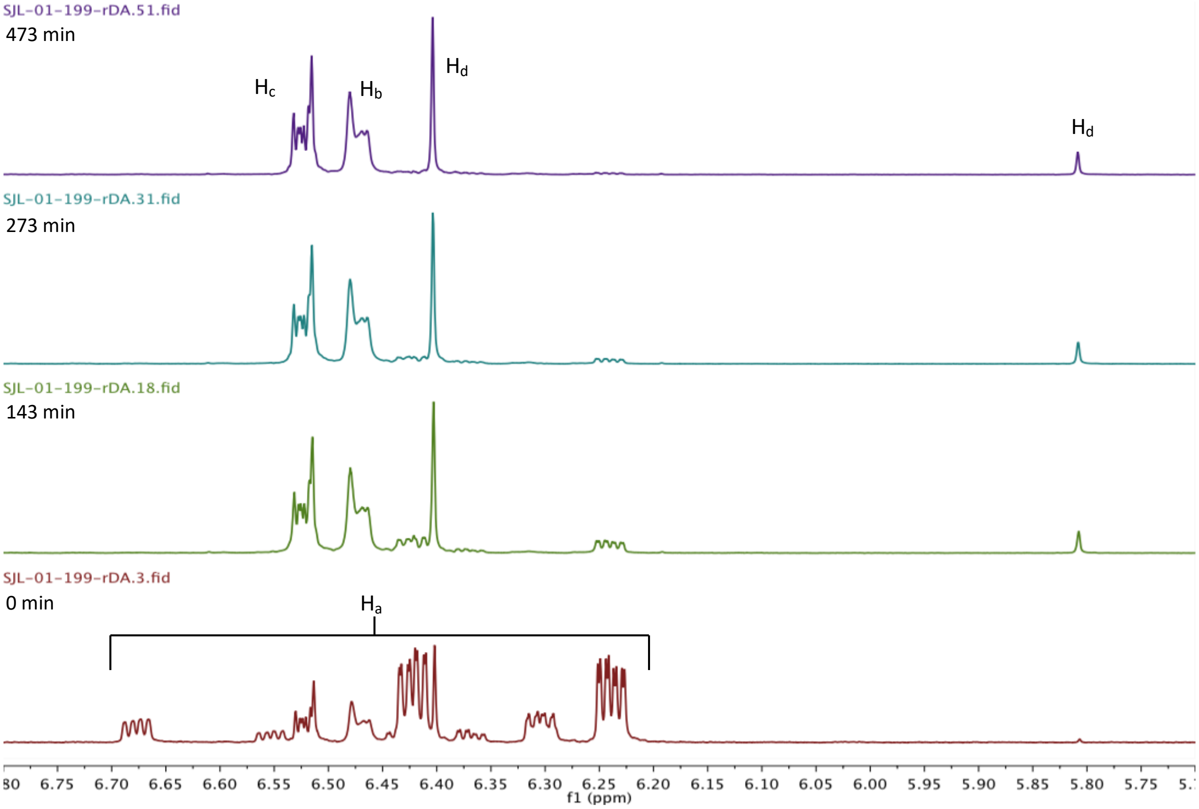

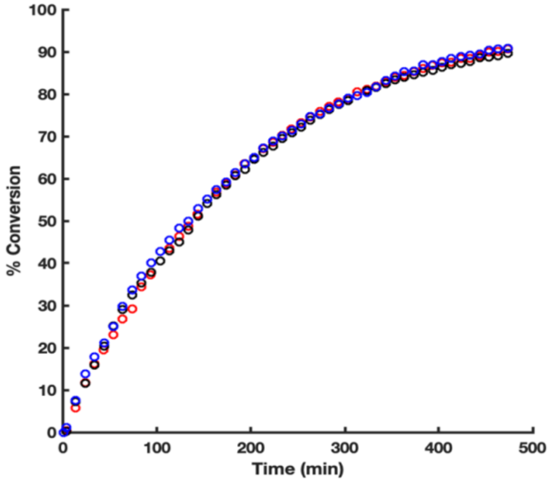

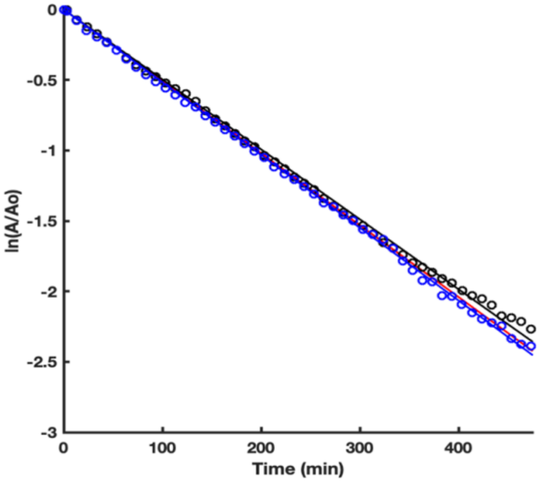

Trial	*k (s^{-1})*	*avg k (s^{-1})*	*$t_{1/2}$ (min)*	*avg $t_{1/2}$ (min)*
1	8.50E-05	8.47 ± 0.46 E-05	135.9	137 ± 8
2	8.27E-05		139.7	
3	8.63E-05		133.8	

Figure S48. Fragmentation kinetics of YND-PT **8a:d3** at 80 °C in $CDCl_3$.

7. Kinetic Simulations

Substrates for which diastereomeric percentages could be obtained (**6a**, **6b**, **6d**, **6e** and **6g**-**6m**) utilized kinetic simulations to extrapolate individual diastereomer rate constants as described previously.[3] The kinetic simulations code using a genetic algorithm and the Simplex method was implemented in MATLAB R2020a.[4,5] The kinetic simulations code for the case when three diastereomers are represented by overlapping ^{1}H NMR signals is reported below in the form of a MATLAB function:

```
function [rateConstants, error] = geneticSimplex(lnConcentrationData, ...
                         timeData, diastereomerRatios)
observedFit = @(m, x) m(1) * x;
fit_model = fitnlm(timeData, lnConcentrationData, observedFit, [1]);
kObs = -fit_model.Coefficients.Estimate(1);
errorFunc = @(k) sum((lnConcentrationData - log(diastereomerRatios(1) ...
         .* exp(-k(1) .* timeData) + diastereomerRatios(2) .* ...
         exp(-k(2) .* timeData) + diastereomerRatios(3) .* exp(-k(3) ...
         .* timeData))).^2);
gaOptions = optimoptions('ga', 'PopulationSize', 100, 'MaxGenerations', ...
         10000, 'CrossoverFraction', 0.5, 'MutationFcn', ...
         {'mutationadaptfeasible', 0.1});
[k] = ga(errorFunc, 3, [1 -1 0; -1 0 1; -1 0 0; 0 -1 0; 0 0 -1], ...
     [0; 0; 0; 0; 0], [], [], [kObs kObs 0], [1 1 kObs], [], gaOptions);
simplexOptions = optimset('MaxIter', 10000, 'TolFun', 1 * 10 ^ -4);
[rateConstants, error] = fminsearch(errorFunc, [k(1) k(2) k(3)], ...
                   simplexOptions);
end
```

Table S1. Kinetic Simulations Results for **6a:d1-3**

Temperature (°C)	k_{d1} (s^{-1})	k_{d2} (s^{-1})	k_{d3} (s^{-1})	SSE
30	3.02E-07	2.72E-06	6.13E-08	0.01288
30	3.26E-07	3.48E-06	4.72E-08	0.00624
30	3.15E-07	3.92E-06	5.34E-08	0.00393
70	1.06E-04	5.93E-04	2.64E-05	0.00369
70	1.03E-04	6.65E-04	2.08E-05	0.05192
70	1.01E-04	5.79E-04	2.03E-05	0.01518
80	3.37E-04	1.86E-03	5.82E-05	0.01286
80	3.01E-04	1.54E-03	7.73E-05	0.00575
80	3.14E-04	1.44E-03	6.18E-05	0.00695
90	8.66E-04	2.04E-03	2.04E-04	0.00189
90	7.38E-04	2.07E-03	2.63E-04	0.00709
90	9.13E-04	1.75E-03	2.19E-04	0.02345

8. References – Part 1

(1) Coskun, N.; Erden, I. An Efficient Catalytic Method for Fulvene Synthesis. *Tetrahedron Lett.* **2011**, *67*, 8607–8614.

(2) Erden, I.; Xu, F.; Sadoun, A.; Smith, W.; Sheff, G.; Ossun, M. Scope and Limitations of Fulvene Syntheses. Preparation of 6-Vinyl-Substituted and -Functionalized Fulvenes. First Examples. *J.Org.Chem.* **1995**, *60*, 813–820.

(3) Malouf, D. M.; Richardson, A. D.; L'Heureux, S. H.; McDonough, E. A.; Henry, A. M.; Sheng, J. Y.; Medhurst, E. A.; Canales, A. E.; Fleischer, C. J.; Cecil, T. B.; Thurman, S. E.; McMullen, C. C.; Costanzo, P. J.; Bercovici, D. A. Ylidenenorbornadiene Carboxylates: Experimental Kinetic Analysis of a Nucleophile-Induced Fragmentation Reaction. *Org. Lett.* **2022**, *24* (15), 2793–2797.

(4) Sieverding, P.; Osterbrink, J.; Besson, C.; Kögerler, P. Kinetics and Mechanism of Pyrrolidine Buffer-Catalyzed Fulvene Formation. *J. Org. Chem.* **2019**, *84* (2), 486–494. https://doi.org/10.1021/acs.joc.8b01660.

(5) Jeong, H.; John, J. M.; Schrock, R. R. Formation of Alternating Trans- A -Alt- B Copolymers through Ring-Opening Metathesis Polymerization Initiated by Molybdenum Imido Alkylidene Complexes. *Organometallics* **2015**, *34* (20), 5136–5145. https://doi.org/10.1021/ACS.ORGANOMET.5B00709/ASSET/IMAGES/LARGE/OM-2015-00709D_0015.JPEG.

(6) Stone, K. J.; Daniel Little, R. An Exceptionally Simple and Efficient Method for the Preparation of a Wide Variety of Fulvenes. **1984**.

(7) Kuthanapillil, J. M.; Nijamudheen, A.; Joseph, N.; Prakash, P.; Suresh, E.; Datta, A.; Radhakrishnan, K. V. Cycloaddition Profile of Pentafulvenes with 3-Oxidopyrylium Betaine: Experimental and Theoretical Investigations. *Tetrahedron* **2013**, *69* (46), 9751–9760. https://doi.org/10.1016/J.TET.2013.09.016.

(8) Xue, F.; Deng, H.; Xue, C.; Mohamed, D. K. B.; Tang, K. Y.; Wu, J. Reaction Discovery Using Acetylene Gas as the Chemical Feedstock Accelerated by the "Stop-Flow" Micro-Tubing Reactor System. *Chem. Sci.* **2017**, *8* (5), 3623–3627. https://doi.org/10.1039/C7SC00408G.

(9) Pracht, P.; Bohle, F.; Grimme, S. Automated Exploration of the Low-Energy Chemical Space with Fast Quantum Chemical Methods. *Phys. Chem. Chem. Phys.* **2020**, *22* (14), 7169–7192. https://doi.org/10.1039/C9CP06869D.

(10) MacroModel. Schrodinger, LLC: New York, NY 2021.

(11) Mohamadi, F.; Richards, N. G. J.; Guida, W. C.; Liskamp, R.; Lipton, M.; Caufield, C.; Chang, G.; Hendrickson, T.; Still, W. C. Macromodel—an Integrated Software System for Modeling Organic and Bioorganic Molecules Using Molecular Mechanics. *J. Comput. Chem.* **1990**, *11* (4), 440–467. https://doi.org/10.1002/jcc.540110405.

(12) Kohler, E. P.; Kable, J. The Diels-Alder Reaction in the Fulvene Series. *J. Am. Chem. Soc.* **1935**, *57* (5), 917–918. https://doi.org/10.1021/ja01308a042.

(13) Swan, E.; Platts, K.; Blencowe, A. An Overview of the Cycloaddition Chemistry of Fulvenes and Emerging Applications. *Beilstein J. Org. Chem.* **2019**, *15*, 2113–2132. https://doi.org/10.3762/bjoc.15.209.

(14) Preethalayam, P.; Krishnan, K. S.; Thulasi, S.; Chand, S. S.; Joseph, J.; Nair, V.; Jaroschik, F.; Radhakrishnan, K. V. Recent Advances in the Chemistry of Pentafulvenes. *Chem. Rev.* **2017**, *117* (5), 3930–3989. https://doi.org/10.1021/acs.chemrev.6b00210.

(15) Howard, M. H.; Alexander, V.; Marshall, W. J.; Roe, D. C. 6 , 6-Dimethylfulvene : Cycloadditions and a Rearrangement. *Synthesis (Stuttg).* **2003**, *30* (5), 120–129.

(16) Houk, K. N. Theoretical and Experimental Insights into Cycloaddition Reactions BT - Organic Chemistry; Springer Berlin Heidelberg: Berlin, Heidelberg, 1979; pp 1–40.

(17) Houk, K. N.; Luskus, L. J.; Bhacca, N. S. The Novel Double [6 + 4] Cycloaddition of Tropone to Dimethylfulvene. *J. Am. Chem. Soc.* **1970**, *92* (21), 6392–6394. https://doi.org/10.1021/ja00724a077.

(18) Wu, T. C.; Houk, K. N. Construction of Linear-Fused Tricyclopentanoids by Intramolecular [6 + 2] Cycloadditions of Fulvenes with Enamines. *J. Am. Chem. Soc.* **1985**, *107* (18), 5308–5309. https://doi.org/10.1021/ja00304a065.

(19) Liu, C.-Y.; Smith, D. A.; Houk, K. N. An Intramolecular [8+6] Cycloaddition. *Tetrahedron Lett.* **1986**, *27* (40), 4881–4884. https://doi.org/https://doi.org/10.1016/S0040-4039(00)85087-X.

(20) Bissette, A. J.; Fletcher, S. P. Mechanisms of Autocatalysis. *Angew. Chemie - Int. Ed.* **2013**, *52* (49), 12800–12826. https://doi.org/10.1002/anie.201303822.

(21) Hanopolskyi, A. I.; Smaliak, V. A.; Novichkov, A. I.; Semenov, S. N. Autocatalysis: Kinetics, Mechanisms and Design. *ChemSystemsChem* **2021**, *3* (1), e2000026. https://doi.org/https://doi.org/10.1002/syst.202000026.

(22) Rundlöf, T.; Mathiasson, M.; Bekiroglu, S.; Hakkarainen, B.; Bowden, T.; Arvidsson, T. Survey and Qualification of Internal Standards for Quantification by 1H NMR Spectroscopy. *J. Pharm. Biomed. Anal.* **2010**, *52* (5), 645–651. https://doi.org/10.1016/j.jpba.2010.02.007.
