# Supplementary material for: Experimental and Theoretical Exploration of the Kinetics and Thermodynamics of the Nucleophile-Induced Fragmentation of Ylidenenorbornadiene Carboxylates
Source: J Org Chem. 2023 Aug 3;88(16):11683–93. doi: 10.1021/acs.joc.3c00980 (PMC10442913; doi:10.1021/acs.joc.3c00980)
Supplement: Supplementary file 2 — jo3c00980_si_002.pdf [file jo3c00980_si_002.pdf]

Supporting Information

Experimental and Theoretical Exploration of the Kinetics and Thermodynamics of the Nucleophile-Induced Fragmentation of Ylidenenorbornadiene Carboxylates

Abigail D. Richardson,[1] Scott J. L'Heureux,[1] Ava M. Henry,[1] Elizabeth A. McDonough,[1] Cameron J. Fleischer,[1] Cameron C. McMullen,[1] Trevor R. Reynafarje,[1] Gisele P. Guerrero,[1] Quinn E. Williams,[1] Qingyang Zhou,[2] David M. Malouf,[1] Spencer E. Thurman,[1] Julia E. Soeller,[1] Jerry Y. Sheng,[1] Erica A. Medhurst,[1] Angel E. Canales,[1] Ty B. Cecil,[1] K.N. Houk,[2] Philip J. Costanzo,[1] Daniel A. Bercovici[1]*

[1]Department of Chemistry and Biochemistry, California Polytechnic State University, 1 Grand Avenue, San Luis Obispo, CA 93047, United States

[2]Department of Chemistry and Biochemistry, University of California, Los Angeles, California 90095, United States;

*email: dbercovi@calpoly.edu

PART 2 of 2

Table of Contents – Part 2 (this document)

Table of Contents – Part 2 (this document) 62
9. Computational Methods 63
9.1 ωB97X -D Cartesian coordinates and energies 63
9.2 M06-2X Cartesian coordinates and energies 88
9.3 B3LYP Cartesian coordinates and energies 98
10. References – Part 2 110
11. ^{1}H and ^{13}C NMR Spectra for New Compounds 111

Table of Contents – Part 1 (other document)

1. General S2
2. Synthetic Procedures S2
2.1 Fulvene Synthesis S2
2.2 Ylidenenorbornadiene (Di)carboxylate (YND) Synthesis S2
2.3 Synthesis of Ylidenenorbornadiene Dicarboxylates β-mercaptoethanol (BME) or Propanethiol (PT) Adducts (YND-BME or YND-PT) via Conjugate Addition S6
3. Diastereomer Structural Assignment S11
3.1 Confirmation of cis-7 stereochemistry S11
3.2 Identification of YND-PT 8a diastereomers d1-d3 ...... S17
4. Comparison of Hammett Substrate Diastereomers S28
5. Computational search of PES for lower TS energy of anti-exo diastereomer..... S29
6. 1H NMR Kinetic Studies S32
7. Kinetic Simulations S63
8. References – Part 1 ...... S64

9. Computational Methods

Calculations were performed with GAMESS version R1 released in 2020[1] and ORCA version 5.0.4.[2,3] Gas-phase ground state and transition state geometries were first optimized using the density functional ωB97X-D[4] and the 6-31+G(d) basis set in vacuo. Geometry optimizations and transition state optimizations were performed with tight convergence criteria of 1e-5 hartree/bohr. Prior to optimizations, conformational searches were performed with CREST version 2.10.2.[5] For **6a:d1-4** and **TS1-6a:d1-4**, the generated conformers were re-ranked using single point calculations with the density functional ωB97X-D, the 6-311+G(d,p) basis set, and DMSO implicit solvent using the IEF-PCM solvation model.[6] Vibrational frequencies were calculated in vacuo using the density functional ωB97X-D and the 6-31+G(d) basis set. A MATLAB[7] script was developed to calculate free energies for 1 atm at 298.15 K from the output of a GAMESS vibrational frequency calculation, and applies Truhlar's quasiharmonic correction,[8] which reduces the error in the estimation of entropies arising from the treatment of low frequency vibrational modes as harmonic oscillations by setting all frequencies less than 100 cm^{-1} to 100 cm^{-1}. Single point calculations with DMSO implicit solvent were performed on the optimized geometries using the density functional ωB97X-D and the 6-311+G(d,p) basis set, and the Mulliken charges[9] for **6a:d1** and **TS-6a:d1** were obtained from this correction. These calculations used the isotropic dielectric IEF-PCM solvation model with an iterative solver and used the solute's quantum mechanical density[10] for the IEF-PCM electrostatics. For **6a:d1-d4** and **TS1-6a:d1-4**, multiple structures with the lowest energies using the density functional ωB97X-D were optimized using the density functionals M06-2X[11] and B3LYP[12,13] and the 6-31+G(d) basis set. These ground state and transition state geometries were corrected using Truhlar's quasiharmonic correction and single point corrections with the respective density functional, the 6-311+G(d,p) basis set, and DMSO implicit solvent. With ORCA, further single point corrections were applied to the lowest energy structures each respective density functional using DLPNO-CCSD(T)[14] and the def2-TZVPP basis set and DMSO implicit solvent. Geometries are displayed using CYLview.[15]

9.1 ωB97X-D Cartesian coordinates and energies

3a

18

C 0.00000000 0.00000000 0.00000000

C 0.82072000 1.25920300 -0.03139600

C 2.17442900 1.25923400 -0.03101300

C 3.06607900 2.43100400 -0.06024800

C 4.34616800 1.99066000 -0.04940300

C 4.34619000 0.52787900 -0.01250000

C 3.06612000 0.08749000 -0.00164000

H 2.74881000 -0.94684600 0.02449800

H 5.23595400 -0.09141400 0.00307200
H 5.23594000 2.60992900 -0.06499100
H 2.74874300 3.46533400 -0.08630900
C -0.00004000 2.51835600 -0.06365200
H -0.66408900 2.55728800 0.80938900
H -0.64599100 2.52319700 -0.95112000
H 0.60255200 3.42728900 -0.07524700
H -0.64839100 -0.00402000 0.88568800
H 0.60264900 -0.90886100 0.01413100
H -0.66169100 -0.03985900 -0.87480100

trans-7

26

C 0.00000000 0.00000000 0.00000000
S -1.75886258 0.28432901 -0.41314770
C -2.41104369 -1.36036560 -0.54088333
C -2.94146236 -1.83879484 -1.67362572
C -2.79694018 -1.20875593 -3.01313607
O -3.97103726 -1.09723008 -3.62994264
C -3.93978432 -0.55548870 -4.95678795
H -4.97653791 -0.55415438 -5.28974792
H -3.53685836 0.45946806 -4.93937478
H -3.32377067 -1.18257333 -5.60516701
O -1.73855933 -0.89640482 -3.52219138
H -3.49243280 -2.77599535 -1.64064999
C -2.55193388 -2.23951678 0.67690419
O -2.02200164 -1.69444384 1.77215838
C -2.10292159 -2.48558999 2.96057474
H -3.14730768 -2.68554189 3.21103590
H -1.57535852 -3.43227025 2.82177279
H -1.62806316 -1.88783693 3.73756917
O -3.08074216 -3.32878031 0.65676312
C 0.88939219 -0.22070807 -1.22233685
O 0.60327485 -1.41341801 -1.90693287
H -0.15259312 -1.26104435 -2.50060529
H 1.92656453 -0.29657351 -0.87390758
H 0.82329189 0.64926130 -1.89106457

H 0.06668964 -0.84513332 0.68914857
H 0.31604357 0.90065466 0.53825487

cis-7
26

C 0.00000000 0.00000000 0.00000000
C -0.16919633 -0.79568107 -1.29652609
H 0.73599049 -0.69973988 -1.90601715
H -0.30755673 -1.86156103 -1.09277809
S -1.50972792 -0.20113940 -2.38369106
C -2.97012087 -0.74007951 -1.55343224
C -4.07269993 0.02114869 -1.55115326
C -5.34243076 -0.44998237 -0.96466300
O -5.50925903 -1.51072915 -0.39632173
O -6.31059509 0.46360687 -1.12462265
C -7.58325967 0.12678195 -0.56933019
H -7.49975388 -0.01750619 0.51065861
H -7.96915308 -0.78731398 -1.02740111
H -8.23061612 0.97328659 -0.79548353
H -4.05403806 1.02533757 -1.96105489
C -2.94975971 -2.12043283 -0.94787553
O -2.46862029 -2.36960391 0.14020227
O -3.43095938 -3.02932806 -1.78017665
C -3.54446640 -4.35850938 -1.25766354
H -4.21815475 -4.35394424 -0.39799803
H -2.56374841 -4.73773394 -0.96156043
H -3.95880658 -4.95096467 -2.07181797
O -1.15267918 0.07163317 0.79671881
H -1.54766269 -0.81249296 0.88526390
H 0.84110076 -0.45274146 0.55117952
H 0.27132194 1.03374818 -0.23439195

6a:d1
44

C 0.00000000 0.00000000 0.00000000

C	-0.88500832	0.33395705	1.18885321
C	-2.13397445	0.79195694	1.27666860
C	-2.98405992	1.07227952	0.06368320
H	-3.86172035	0.41310665	0.04928842
H	-2.44082186	0.93927195	-0.87429064
H	-3.36088559	2.10306184	0.09017078
C	-2.81454075	1.05433325	2.59683302
H	-3.14678294	2.09860627	2.66036903
H	-2.16833913	0.85788966	3.45521971
H	-3.71081071	0.42827696	2.69565604
C	0.15914528	0.01469636	2.24498105
C	0.61259970	-1.35229350	1.76316119
C	0.50978013	-1.36398267	0.43089337
H	0.83115304	-2.13656024	-0.25546127
H	1.03014972	-2.12130284	2.40025673
H	-0.11987256	0.09365263	3.29568273
C	1.22757734	1.08323074	1.82708062
C	2.59068473	0.85634217	2.44791758
O	3.14964781	-0.20887318	2.57546515
O	3.10891014	2.01355081	2.89201780
C	4.41942620	1.93009107	3.45051038
H	4.42144831	1.28158982	4.33005465
H	5.12040327	1.53427728	2.71074316
H	4.68545459	2.95029413	3.72594918
H	0.87503985	2.05425423	2.17488060
C	1.16570616	1.02892754	0.25243116
C	2.44727207	0.62153052	-0.46163215
O	2.51536218	-0.20317777	-1.34166007
O	3.49709083	1.34100764	-0.03810898
C	4.74862096	1.04024624	-0.65710711
H	5.46325186	1.73908434	-0.22282529
H	5.03641904	0.00898105	-0.43831567
H	4.68127102	1.18080312	-1.73845430
S	0.65354590	2.60791774	-0.57616436
C	1.33956312	3.95017390	0.45465802
C	0.22460508	4.69256937	1.19528415
O	-0.54336770	3.85708658	2.03595407
H	-0.95510227	3.17319008	1.48253448

H 0.66870419 5.45064363 1.84785493
H -0.42124739 5.20862684 0.46988860
H 1.86865156 4.63916547 -0.21171919
H 2.07212968 3.53419983 1.15013170
H -0.41266236 0.06199218 -1.00609995

6a:d2

44

C 0.00000000 0.00000000 0.00000000
C 0.06263107 1.18352767 0.94889286
C -0.24342404 2.28053112 0.24994814
C -0.49945468 1.85052548 -1.18524557
C -1.09237942 0.47146905 -0.94515349
C -2.15199508 -0.15535919 -1.45458504
C -3.09249063 0.50045193 -2.43246382
H -4.11272897 0.52421293 -2.02806802
H -3.13311860 -0.07327549 -3.36727775
H -2.80348570 1.52530513 -2.67693718
C -2.50695920 -1.57613025 -1.09668201
H -2.54596951 -2.19786673 -2.00081325
H -1.79320189 -2.03061274 -0.40617906
H -3.50308249 -1.62031504 -0.63741254
C 0.86671769 1.41309896 -1.80431712
C 1.90521288 2.51164915 -1.80582654
O 1.78449282 3.59926507 -1.29050076
O 2.98283129 2.15690957 -2.52232810
C 4.04532725 3.10915447 -2.56004825
H 3.70594777 4.04599136 -3.00841037
H 4.41169555 3.30636135 -1.54864017
H 4.82421637 2.65249718 -3.17074763
H 0.72305192 1.12431875 -2.84975877
C 1.21825798 0.10173619 -1.00207434
C 1.21645205 -1.10425270 -1.94278161
O 1.38870237 -1.04375917 -3.13900987
O 1.00954031 -2.25279312 -1.28970133
C 1.02771699 -3.43765392 -2.09511729
H 2.01936829 -3.57143496 -2.53229855

H 0.79866494 -4.25289261 -1.40952770
H 0.27262069 -3.36832057 -2.88119911
S 2.77042653 0.11241729 -0.01228551
C 4.10286723 -0.32903832 -1.19286642
C 4.78820371 -1.62908368 -0.78078437
O 3.92642195 -2.74510600 -0.82495986
H 3.15971464 -2.54552689 -0.26595202
H 5.23370533 -1.52060748 0.21964309
H 5.59842601 -1.84220514 -1.48515882
H 4.82635069 0.49191853 -1.20145481
H 3.68505159 -0.41300227 -2.19855380
H -1.04567167 2.55703773 -1.80934656
H -0.22089811 3.30877195 0.58612483
H 0.38439694 1.12480869 1.98208385
H -0.08103040 -0.98320954 0.45706225

6a:d3

44

C 0.00000000 0.00000000 0.00000000
C -1.24528699 -0.50042643 0.70978434
C -2.53255787 -0.19018538 0.57281054
C -3.60746694 -0.78641670 1.44210187
H -4.37898479 -1.26589459 0.82534573
H -3.22135714 -1.52065047 2.15059184
H -4.09892404 0.00360839 2.02348967
C -3.02702702 0.80631453 -0.44361480
H -3.55471673 1.62638607 0.05995012
H -2.22349630 1.24350372 -1.04127929
H -3.74455429 0.33618599 -1.12861984
C -0.50944413 -1.41156364 1.67829754
C 0.39172929 -2.15836520 0.71041716
C 0.69460557 -1.32197734 -0.28806458
H 1.37599908 -1.48752111 -1.11449755
H 0.77509243 -3.16019321 0.87073794
H -1.09329801 -2.01601672 2.36975667
C 0.40523407 -0.36504846 2.39357455
C -0.29549472 0.25173522 3.58613822

O -1.45483313 0.08498324 3.88664076
O 0.55643604 0.97702343 4.32724497
C -0.01244943 1.63705851 5.45750548
H -0.81990903 2.30275257 5.14000527
H 0.80393374 2.20409889 5.90505693
H -0.40739253 0.90812640 6.16943627
H 1.33091964 -0.81235881 2.76614059
C 0.76263841 0.63824234 1.22952297
C 2.26524107 0.71071752 0.99607713
O 3.10556396 0.44519528 1.82475765
O 2.55614978 1.11723756 -0.24558688
C 3.95165450 1.25959727 -0.54120980
H 4.38376673 2.03780452 0.09112318
H 4.46886866 0.31107941 -0.38002501
H 3.99378745 1.55056769 -1.59028114
S 0.08606292 2.34854375 1.41264671
C 1.29846661 3.24632204 2.45552364
C 1.92189393 4.41083891 1.69009300
O 2.67405815 3.99781553 0.57070335
H 2.09358577 3.46084614 0.00943824
H 1.14166973 5.12677755 1.39071128
H 2.62080112 4.93483475 2.34952999
H 2.06616804 2.54947172 2.79912539
H 0.76868220 3.61990548 3.33725420
H -0.09836207 0.68635468 -0.83846307

6a:d4

44

C 0.00000000 0.00000000 0.00000000
C 0.42129234 -0.11436818 1.45391285
C 0.35118849 1.10597606 1.99363700
C -0.07277356 2.06564266 0.89446204
C -0.98406470 1.15558800 0.09004368
C -2.19575989 1.33268633 -0.43372623
C -2.96220014 2.62018435 -0.28202687
H -3.92274559 2.44496939 0.21971558
H -3.18948918 3.04044827 -1.27061890

H -2.41140518 3.37556188 0.28176146
C -2.90224149 0.26943060 -1.23455286
H -3.09293366 0.62483640 -2.25624361
H -3.87997352 0.04203366 -0.79029759
H -2.33239387 -0.65918433 -1.30238883
C 1.18225276 2.14472529 -0.05120009
C 1.23163707 3.37730646 -0.92675301
O 2.25967446 3.97399508 -1.19319766
O 0.03013811 3.79744796 -1.31743580
C 0.01615055 4.89673044 -2.22939661
H 0.49470948 5.77193967 -1.78383479
H 0.53970596 4.61718979 -3.14687764
H -1.03689117 5.09327506 -2.42939447
H 2.09928500 2.17110702 0.53867872
C 1.12710419 0.75888990 -0.80118606
C 0.75368395 0.86790188 -2.27637699
O 1.12347811 1.75903784 -3.01312985
O 0.02114147 -0.16358110 -2.70176439
C -0.30744056 -0.16616059 -4.09076584
H 0.60034751 -0.23321257 -4.69554973
H -0.93215194 -1.04668796 -4.23776916
H -0.85338711 0.74278568 -4.35480926
S 2.69272410 -0.20565779 -0.75039788
C 3.90126771 0.87054899 -1.60597476
C 4.89724682 1.58803648 -0.69392847
O 4.33221571 2.55799923 0.15199689
H 3.82541620 3.19497464 -0.38360015
H 5.39762421 0.86703418 -0.04035555
H 5.66597114 2.03731973 -1.34416484
H 3.35637761 1.58762082 -2.22833913
H 4.45724110 0.21548565 -2.28591947
H -0.45023248 3.03579428 1.21451938
H 0.65421264 1.40764408 2.99011496
H 0.78440720 -1.02362035 1.91796371
H -0.31189493 -0.92100093 -0.48586875

TS1-6a:d1

44

C	0.00000000	0.00000000	0.00000000
C	-0.55485200	-0.60372600	-1.25744700
C	-0.47613500	0.01620800	-2.45509600
C	-1.05913400	-0.40259700	-3.71727200
C	-0.56062800	0.39059900	-4.72903700
C	0.22853800	1.41227800	-4.16437100
C	0.15672900	1.32756300	-2.76108900
H	0.90127700	1.80296500	-2.13270400
H	0.72231500	2.20584500	-4.70828900
H	-0.79604100	0.29525800	-5.78181600
H	-1.73839600	-1.23517100	-3.85112700
C	-1.22449800	-1.93317000	-1.06420300
H	-1.68030300	-2.32495800	-1.97436700
H	-0.49835300	-2.67066600	-0.69917500
H	-2.00624400	-1.84428400	-0.30044100
H	0.57015800	0.91444900	-0.17268600
H	-0.82961500	0.23373100	0.68071100
H	0.64736300	-0.71529300	0.52165900
C	-4.46236000	2.17922900	-5.98427000
O	-4.11730900	1.81956700	-4.64869500
C	-2.91902200	2.27821800	-4.21264300
C	-2.58608600	1.93125300	-2.84584800
C	-1.40355200	2.47958200	-2.32076300
C	-0.92817200	3.86016400	-2.73623200
O	-1.92927700	4.74006100	-2.60844800
C	-1.62224800	6.08202000	-2.99090500
H	-2.54334300	6.64384100	-2.83834700
H	-1.32411200	6.10846100	-4.04178700
H	-0.81696700	6.48450100	-2.37125600
O	0.20325800	4.17562600	-3.02339800
H	-1.29884200	2.35787800	-1.24194000
S	-3.63787200	0.93613100	-1.83119400
C	-4.61238800	2.21902000	-0.95802500
C	-5.42413200	3.11282300	-1.89364000
O	-6.30753800	2.39318400	-2.72132800
H	-5.75886900	1.86580600	-3.32363900
H	-4.74006000	3.73567200	-2.48912700

H -6.04261100 3.78377200 -1.28804900
H -3.94347900 2.83226700 -0.34324900
H -5.28200400 1.65927000 -0.29559400
O -2.18273100 2.94288100 -4.93348500
H -5.45370700 1.75765800 -6.15205500
H -3.74323900 1.75988200 -6.69294900
H -4.48593600 3.26608800 -6.09684500

TS1-6a:d2

44

C 0.00000000 0.00000000 0.00000000
C -0.58889900 -0.80036200 -1.12307200
C -1.57743700 -0.32875800 -1.91335400
C -2.28578200 -1.02207300 -2.96895200
C -2.99742700 -0.09632700 -3.71446700
C -2.91078800 1.15521300 -3.08652800
C -2.22774500 1.00830100 -1.85851800
H -1.79098300 1.86640800 -1.35911200
H -3.39859300 2.06440900 -3.41077500
H -3.58408500 -0.31880600 -4.59697600
H -2.21744000 -2.08015800 -3.18887800
C 0.01230400 -2.16422700 -1.30050100
H -0.57664700 -2.81388300 -1.95019800
H 1.01855700 -2.07510900 -1.73018800
H 0.12616300 -2.65677200 -0.32786700
H 1.09430200 0.02274200 -0.07133900
H -0.36357000 1.02811000 0.03774400
H -0.24871000 -0.48577400 0.95287300
C -2.41900500 -3.02801500 1.16216600
O -2.81946900 -1.82167300 0.52115200
C -3.83369500 -1.94812700 -0.37607000
C -4.26383000 -0.67881600 -0.94514000
C -3.64060300 0.53330500 -0.58228800
C -4.45948900 1.80500100 -0.49033400
O -5.35347900 1.68532100 0.50144100
C -6.18077900 2.82836900 0.73466500
H -6.84828100 2.54008200 1.54620200

H -6.74981200 3.07447900 -0.16536300
H -5.57203100 3.68875000 1.02344600
O -4.31885800 2.81965100 -1.13179800
H -2.98333600 0.45073500 0.28381600
S -5.63605500 -0.69633400 -2.06535700
C -7.11355300 -0.65307200 -0.97904400
C -7.18197300 -1.76784600 0.06295300
O -7.15641300 -3.05894000 -0.48446400
H -6.23977900 -3.23703800 -0.76167800
H -8.13388200 -1.66974700 0.59830300
H -6.37741800 -1.62796300 0.80168100
H -7.94837000 -0.75513700 -1.68084000
H -7.18588500 0.31982700 -0.48436900
O -4.31823100 -3.04865600 -0.60898500
H -3.24915000 -3.45812100 1.72856000
H -1.60787300 -2.74484600 1.83528500
H -2.07210600 -3.76295700 0.43067300

TS1-6a:d3

44

C 0.00000000 0.00000000 0.00000000
C 1.14289200 0.89126000 0.38231700
C 1.94596200 1.50379000 -0.51723700
C 3.03863900 2.41667200 -0.24220400
C 3.28716800 3.16948100 -1.38629900
C 2.56260000 2.62248800 -2.44471300
C 1.91362700 1.43921500 -2.00236200
H 1.09484900 0.98957500 -2.55146000
H 2.61384200 2.94914500 -3.47717300
H 3.99460500 3.98678700 -1.45789400
H 3.49925300 2.58382800 0.72303200
C 1.31241700 1.06975100 1.86445600
H 2.21846100 1.60998100 2.14055700
H 0.44845600 1.60461900 2.27919800
H 1.34310000 0.08601000 2.34749300
H 0.24675200 -1.03541700 0.26743900
H -0.89605800 0.27753400 0.56878500

H -0.22855500 0.01487000 -1.06409300
C 6.66616800 2.52779400 -2.90148100
O 5.55161700 1.67318400 -2.66594000
C 5.49806500 1.10699600 -1.43410900
C 4.31213300 0.28000800 -1.24865800
C 3.35760000 0.14646000 -2.28848900
H 3.71234600 0.46911700 -3.26500100
C 2.62186900 -1.16942000 -2.42974100
O 1.43254400 -1.36592800 -2.33571200
O 3.49100600 -2.13227200 -2.77020600
C 2.93178000 -3.43386200 -2.96521100
H 2.44351200 -3.77631500 -2.04932000
H 3.77593400 -4.07572900 -3.21534000
H 2.20355000 -3.41959600 -3.77984600
S 4.15949300 -0.63293000 0.26577700
C 5.21459800 -2.11040900 0.01749900
C 6.68092000 -1.82648700 -0.30389400
O 7.33162000 -1.04441700 0.66099000
H 7.04323500 -0.12491200 0.51987100
H 6.75810300 -1.37378300 -1.30427900
H 7.20665200 -2.78810000 -0.34287900
H 4.78088100 -2.74380500 -0.76156700
H 5.14479900 -2.63286700 0.97803500
O 6.39150800 1.29535800 -0.61766400
H 6.66286500 3.36902900 -2.20229700
H 6.55011300 2.88258000 -3.92609000
H 7.60430500 1.97864000 -2.78924200

TS1-6a:d4

44

C 0.00000000 0.00000000 0.00000000
C -0.78212800 -1.17370000 0.50599400
C -1.62997300 -1.89419200 -0.26076300
C -2.38788900 -3.06780000 0.12595200
C -2.76356300 -3.75011100 -1.02762200
C -2.47082900 -2.95067500 -2.13507100
C -1.98466000 -1.69837900 -1.68786600

H	-1.47057700	-1.00185200	-2.33896100
H	-2.71536300	-3.18955700	-3.16395200
H	-3.27540000	-4.70392500	-1.05435500
H	-2.51251500	-3.44148800	1.13439900
C	-0.52636500	-1.50688700	1.94851100
H	-1.25456900	-2.20062300	2.37208100
H	0.47297400	-1.94630400	2.06323700
H	-0.53600900	-0.58830000	2.54731800
H	-0.38070100	0.91696100	0.46828200
H	1.05344100	-0.09887700	0.29061100
H	-0.06586900	0.13642500	-1.07792300
C	-3.19498300	0.82680900	2.42753400
O	-3.29845400	0.18353600	1.16181700
C	-4.25427600	-0.77213100	1.06357000
C	-4.38051200	-1.31282400	-0.28738100
C	-3.72662100	-0.74697400	-1.40371200
H	-4.19117700	-0.99878500	-2.35613400
C	-3.34052600	0.71875500	-1.42979600
O	-2.26609000	1.19048200	-1.72193200
O	-4.42554600	1.46848000	-1.18933800
C	-4.21941000	2.88165200	-1.20329700
H	-3.48167000	3.16142400	-0.44680100
H	-5.19071400	3.31757300	-0.97146100
H	-3.87335000	3.21173000	-2.18599000
S	-5.63893800	-2.53000400	-0.55579500
C	-7.07869100	-1.48090600	-0.98064000
C	-7.46706500	-0.47596700	0.10486900
O	-7.74265000	-1.06565700	1.34866900
H	-6.89147100	-1.33022700	1.73973700
H	-8.38440900	0.03249900	-0.21395700
H	-6.68115800	0.28952500	0.19009300
H	-7.89584700	-2.19243900	-1.14138000
H	-6.88606400	-0.95317700	-1.92261700
O	-4.92759300	-1.08779600	2.03433700
H	-2.38284000	1.54784500	2.32276000
H	-2.96708500	0.10142600	3.21292000
H	-4.12937800	1.33699600	2.67650700

TS2-6a:d3

44

C	0.00000000	0.00000000	0.00000000
C	0.55906600	-1.33819500	-0.37962400
C	1.25425600	-2.12783700	0.47270100
C	1.74072100	-3.45166800	0.17586100
C	2.14650900	-4.10072100	1.32086800
C	2.13749600	-3.18646300	2.38031600
C	1.66205100	-1.86377800	1.91580500
H	0.87251800	-1.45801800	2.55677200
C	2.90121800	-0.82791800	2.03295000
C	2.41615600	0.59515800	2.22625700
O	1.32168600	0.90120600	2.65207200
O	3.38423400	1.48780800	2.01693400
C	3.05300800	2.84890100	2.29692700
H	2.21633800	3.17288300	1.67284700
H	3.95142800	3.41847200	2.06089600
H	2.78626300	2.96789600	3.34987600
H	3.33396100	-1.06387000	3.01462300
C	3.93006700	-1.12507700	1.00755200
C	4.64599600	-2.28193500	1.32471100
O	5.59058900	-2.71881300	0.44169500
C	6.27401300	-3.91462300	0.79753700
H	7.00205400	-4.07995300	0.00239700
H	6.78050900	-3.80378900	1.76000200
H	5.57720700	-4.75612500	0.85300700
O	4.41344400	-2.93601500	2.37575900
S	3.90587200	-0.38990700	-0.59058000
C	5.19564200	0.91767800	-0.53985600
C	6.55694400	0.45680900	-1.05238400
O	7.20994700	-0.44909600	-0.19684300
H	6.65465300	-1.24082500	-0.09171100
H	6.44635500	0.03687200	-2.06460800
H	7.21570200	1.33054500	-1.12460600
H	5.26798500	1.27917000	0.48780300
H	4.82325500	1.73157700	-1.17428700
H	2.31439600	-3.42487100	3.42023000

H 2.49955100 -5.12184100 1.38420300
H 1.72450800 -3.91088100 -0.80536200
C 0.28840900 -1.71971900 -1.80899500
H 0.60150500 -0.89730500 -2.46349100
H 0.80314500 -2.62230100 -2.13956800
H -0.78906800 -1.85908200 -1.96475900
H -0.97751000 0.14726900 -0.47324700
H -0.09721200 0.14036600 1.07561200
H 0.66579200 0.78816000 -0.38055400

TS3-6a:d3
44

C 0.00000000 0.00000000 0.00000000
C 1.41023300 0.43305400 -0.29181300
C 2.48020700 -0.06922900 0.44985100
C 3.94576100 0.28875800 0.33592800
C 4.59568000 -0.67666100 1.26219000
C 3.66087100 -1.21085700 2.08381400
C 2.36579100 -0.77013800 1.64396700
H 1.43774200 -1.04967400 2.12846200
H 3.83930600 -1.85990700 2.93273000
H 5.66679800 -0.80739700 1.33499400
C 4.11399400 1.77856300 1.06047800
C 3.13020700 1.96997000 2.14653000
C 1.79097300 2.24310400 1.92518100
O 1.03633200 2.42662900 3.04717900
C -0.30664800 2.83420400 2.83203900
H -0.34983200 3.75886500 2.24785900
H -0.72273400 2.99850500 3.82707500
H -0.88008200 2.05935200 2.31195300
O 1.23183100 2.30145400 0.78874500
H 3.49115300 1.95501500 3.16699300
C 5.53832400 1.95133600 1.59253900
O 6.46582200 1.43082100 0.77441400
C 7.82325700 1.67199700 1.14159000
H 8.03590800 1.25032400 2.12755100
H 8.02720400 2.74562000 1.15827800

H 8.42244100 1.18186300 0.37467300
O 5.81969700 2.54569900 2.60890200
S 4.04836600 2.99179100 -0.35565400
C 4.12674600 4.63113600 0.43459700
C 2.81630400 5.17245800 1.01851800
O 1.71781100 5.05365700 0.14798900
H 1.47851600 4.10973200 0.11500800
H 2.61395400 4.69568200 1.98550700
H 2.95479600 6.24356600 1.20456700
H 4.91510500 4.64533000 1.19577700
H 4.44706100 5.27216500 -0.39422600
H 4.38710700 0.34399800 -0.66058000
C 1.59216900 1.02592100 -1.65450900
H 1.15552800 0.33983400 -2.39319600
H 1.05297500 1.97515200 -1.72276400
H 2.63365700 1.20475600 -1.91585200
H -0.69825200 0.76008700 -0.35928100
H -0.21252100 -0.93534600 -0.53457300
H -0.18823500 -0.15878400 1.06294300

TS4-6a:d3
44

C 0.00000000 0.00000000 0.00000000
C 0.51321600 0.41754300 -1.35716900
C 0.14377700 -0.10349700 -2.58847100
C -0.48263800 -1.45957100 -2.72514700
H -0.96303100 -1.80705900 -1.81071300
H -1.21831100 -1.46686500 -3.53592900
H 0.30353500 -2.17763000 -3.00199400
C 0.69601600 0.46374800 -3.86907600
H 0.00800300 0.27308700 -4.69714500
H 0.87881000 1.53849000 -3.82197800
H 1.64428800 -0.03759800 -4.10730000
C 1.23850600 1.59311400 -1.14474400
C 1.46376400 1.79064900 0.24789300
C 0.81352200 0.80885200 0.93278900
H 0.75898000 0.71157500 2.01114200

H 2.03417800 2.60432900 0.67811800
H 1.64005200 2.23614100 -1.91956900
H -0.02441500 -1.07302900 0.20048500
C -1.51126500 0.57719100 0.20990700
C -2.50328400 -0.57558300 0.23602000
O -2.28548200 -1.73146200 -0.06319600
O -3.69435500 -0.15877200 0.67861900
C -4.72956000 -1.14051200 0.72749600
H -5.60730000 -0.61495200 1.10206800
H -4.45326000 -1.95905700 1.39636100
H -4.92155000 -1.54117500 -0.27215800
H -1.56229600 1.00838700 1.21625700
C -1.85131300 1.69372000 -0.70798800
C -1.93895900 3.00254500 -0.13043100
O -1.66198700 3.28752100 1.03207400
O -2.41464000 3.95744000 -0.99226200
C -2.46936600 5.27876000 -0.46774400
H -3.11582900 5.32220000 0.41283400
H -2.87718300 5.89447900 -1.27077100
H -1.47012800 5.62843500 -0.19234400
S -2.06489800 1.45697500 -2.40543200
C -3.67384000 0.60416900 -2.60885800
C -4.80524500 1.42941900 -1.98987400
O -4.87175200 2.74270700 -2.49468700
H -4.09404600 3.22749200 -2.17063300
H -4.69028800 1.43268500 -0.89836500
H -5.76216800 0.95463300 -2.23380300
H -3.81030900 0.52209700 -3.69254800
H -3.64490100 -0.40669100 -2.18705500

TS5-6a:d3

62

C 0.00000000 0.00000000 0.00000000
C -1.03669200 1.07653200 0.17199000
C -1.85490800 1.12655100 1.22043700
C -2.99391500 2.06293600 1.55991400
C -2.71479800 2.35125300 3.02798400

C	-2.12125200	1.19445800	3.55170800
C	-2.04397700	0.19919100	2.40327400
H	-1.34576700	-0.62731100	2.52420400
H	-2.30847600	0.84682000	4.56296500
H	-3.37778500	2.99183600	3.59912300
H	-3.17772400	2.93137100	0.92938000
C	-1.06884600	2.09796800	-0.93432900
H	-1.29436300	1.61437100	-1.89349600
H	-1.81339500	2.88018500	-0.76998100
H	-0.08619800	2.57746000	-1.04262900
H	0.99974800	0.44382700	-0.10618500
H	0.01830300	-0.70737700	0.83060400
H	-0.19828900	-0.57346600	-0.91412100
C	-3.51421600	-0.26081700	2.15521500
C	-3.57275600	-1.50077500	1.28879100
O	-2.63305400	-2.01791400	0.73024700
O	-4.81416700	-2.01318700	1.25859200
C	-4.98773800	-3.16995900	0.44188100
H	-4.71207300	-2.94845100	-0.59316500
H	-6.04587900	-3.42202800	0.51449100
H	-4.37131000	-3.99490800	0.80766000
H	-4.03140700	-0.50298700	3.08881400
C	-4.18568900	1.02629100	1.53158700
C	-5.37133100	1.50531100	2.35691100
O	-5.98067500	0.83081500	3.15539500
O	-5.67250100	2.78336400	2.09236300
C	-6.80683300	3.31323000	2.78821600
H	-7.70729300	2.77084200	2.49296900
H	-6.65679300	3.23817900	3.86782500
H	-6.87180200	4.35575800	2.47825100
S	-4.69032100	0.87501600	-0.24122100
C	-6.36576600	0.12857900	-0.21466800
C	-7.38576100	1.06419300	-0.85883800
O	-7.55304300	2.27139900	-0.14950900
H	-6.67532900	2.66930700	-0.03851000
H	-7.10691900	1.26294700	-1.90483600
H	-8.36455000	0.57435400	-0.86164100
H	-6.64705600	-0.10224300	0.81543100

H -6.32386000 -0.81522300 -0.76677800
C -1.08461800 3.84854700 3.30292100
C -0.84565600 3.16325700 4.59239400
C -0.14588600 1.92922200 4.16529500
C 0.45810200 2.21716400 2.93680300
C -0.11426400 3.38134700 2.41266200
H 0.06039200 3.77226000 1.41685700
H 1.14535600 1.56461200 2.41060400
H 0.20264000 1.15620600 4.84011000
C -1.19577300 3.54878500 5.83077500
C -1.92655700 4.83421100 6.11131000
H -2.85476400 4.62974600 6.66152200
H -1.32035100 5.49036300 6.74948200
H -2.18249200 5.38955000 5.20665000
C -0.88647600 2.73289500 7.05722800
H -0.26181100 3.30752100 7.75350200
H -1.81218500 2.48652800 7.59442000
H -0.36562100 1.80053700 6.83118100
H -1.59968200 4.79565900 3.19508000

9a

44

C 0.00000000 0.00000000 0.00000000
C 0.05444095 -0.67243934 -1.34596664
C -0.78001649 -0.38080414 -2.35978787
C -0.67027772 -0.86857979 -3.74022000
C -1.39034068 -0.12253262 -4.58386812
C -2.14366880 0.95794968 -3.86152049
C -1.90345254 0.65499797 -2.35348478
H -1.58962110 1.56531251 -1.83323003
C -3.26832360 0.21233166 -1.72110704
C -3.08951646 -0.35890157 -0.33022218
O -3.35465252 0.57879083 0.59576698
C -3.27307229 0.15195689 1.95957135
H -4.03836843 -0.60337447 2.15419101
H -3.45635541 1.04694263 2.55334882
H -2.28208643 -0.25629193 2.17308745

O   -2.75540795 -1.48658600 -0.05192828
H   -3.82277526 1.14950789 -1.58632776
C   -4.08695810 -0.66422812 -2.66516834
C   -4.23546352 -0.12463724 -3.88896093
O   -5.11560671 -0.54835010 -4.80213533
C   -4.70196369 -0.53470023 -6.16614106
H   -3.84000601 -1.19624294 -6.30669220
H   -4.45692964 0.47752638 -6.49674761
H   -5.55213821 -0.91783128 -6.73038107
O   -3.53823312 0.97950336 -4.24579979
S   -4.87928256 -2.19381349 -2.27354805
C   -6.51101357 -1.63423521 -1.66413776
C   -6.43320165 -0.95305578 -0.30135173
O   -5.85686483 -1.77440464 0.69254346
H   -5.03099333 -2.13868360 0.33310314
H   -7.44416898 -0.71602988 0.04607530
H   -5.88467827 -0.00202453 -0.39059027
H   -7.11481484 -2.54541875 -1.59022307
H   -6.96759283 -0.97205746 -2.40663937
H   -1.80423621 1.95994286 -4.13569806
H   -1.44129865 -0.23567562 -5.66163375
H   -0.03400124 -1.69153033 -4.04530087
C   1.15275889 -1.69819088 -1.45119825
H   2.13513215 -1.22791844 -1.31097209
H   1.03906806 -2.44342958 -0.65391084
H   1.16720193 -2.23219285 -2.40269132
H   -0.25887604 -0.72830470 0.77805466
H   0.98808599 0.40614926 0.25349214
H   -0.72076008 0.81948889 0.05288234

10a

44

C   0.00000000 0.00000000 0.00000000
C   0.19171958 0.32886702 -1.48528331
C   -0.89857800 1.22586275 -2.01616105
C   -2.08806259 0.71874269 -2.80664810
C   -2.84486823 1.98497090 -3.12010450

C	-2.16303239	3.04137009	-2.65111591
C	-0.96076972	2.57016401	-1.96201645
H	-0.22568553	3.22438054	-1.50423705
H	-2.44496296	4.08306524	-2.75249826
H	-3.78298138	2.00361763	-3.65888776
H	-1.69967664	0.31490711	-3.75627232
C	-2.86963026	-0.44755399	-2.14813075
C	-2.04440695	-1.69714088	-2.02143097
C	-0.71870825	-1.83712697	-2.14785683
O	-0.20509037	-3.08781787	-2.23590058
C	1.02337745	-3.32694581	-1.56028195
H	1.83736588	-2.73814482	-1.99079319
H	1.22523106	-4.39072214	-1.69075395
H	0.92840538	-3.10164639	-0.49079248
O	0.23331290	-0.88954177	-2.27247626
H	-2.57447589	-2.62457068	-1.84346364
C	-3.49847400	0.00790929	-0.82203283
O	-3.42994202	-0.94112075	0.11686451
C	-4.10269582	-0.64681503	1.34708148
H	-5.17264274	-0.53183040	1.15915916
H	-3.69911546	0.26702887	1.78936019
H	-3.90939860	-1.50484833	1.99033711
O	-4.03528852	1.07774563	-0.64387987
S	-4.29183387	-0.78885664	-3.31822614
C	-5.26834554	-2.07888850	-2.46507845
C	-6.62989376	-1.56267699	-2.00242851
O	-6.54234206	-0.61237717	-0.96473986
H	-6.09739750	0.18297814	-1.29483226
H	-7.19196227	-1.16101079	-2.85778528
H	-7.20139578	-2.40141867	-1.59166159
H	-5.40112078	-2.90698467	-3.16897633
H	-4.70902233	-2.45410792	-1.60349815
C	1.57508120	0.93562773	-1.71122973
H	2.34641602	0.24163375	-1.36204079
H	1.73318617	1.13835127	-2.77366653
H	1.67731583	1.87271437	-1.15630571
H	0.81159825	-0.64874595	0.34869256
H	0.02420027	0.93047371	0.57540095

H -0.95263051 -0.49705284 0.19395517

11a

44

C 0.00000000 0.00000000 0.00000000
C -0.38057845 1.46357729 -0.03299694
C 0.26347588 2.47352489 0.85450992
C 0.07936914 2.13253083 2.33572600
H -0.99440068 2.17034740 2.55089951
H 0.44048589 1.13145082 2.58257007
H 0.57372842 2.86243059 2.98497208
C -0.17693260 3.90947546 0.57040137
H 0.38090998 4.61684826 1.19360676
H -0.02413349 4.18046250 -0.47818442
H -1.24100332 4.01931637 0.80853110
S 2.11501447 2.41437012 0.31438699
C 2.32738849 0.95567975 -0.55208784
C 1.52902126 -0.27322847 -0.16491316
C 2.12120089 -0.93484431 1.06771472
O 1.72121750 -0.78991829 2.20815776
O 3.17736658 -1.68401873 0.75455623
C 3.91117836 -2.24445260 1.84807537
H 3.25336917 -2.84434001 2.48128555
H 4.36981442 -1.44144651 2.42910736
H 4.67800245 -2.86666816 1.38804614
H 1.68439799 -0.96430238 -0.99859174
C 2.76166224 1.05945591 -1.92228673
O 2.72447020 0.14587195 -2.73564580
O 3.28406433 2.27755377 -2.24777673
C 3.74513345 2.40320594 -3.58749619
H 2.92796667 2.24865620 -4.29782331
H 4.53711116 1.67872566 -3.79557732
H 4.13002488 3.42088111 -3.66825871
C 3.05589927 2.30290802 1.88132207
C 4.54428176 2.11818647 1.56401408
O 4.87227768 0.79694277 1.21266140
H 4.37417657 0.57027132 0.40629849

H 5.11250417 2.34549111 2.47079539
H 4.85884440 2.82878971 0.78514430
H 2.89049617 3.25433288 2.39847567
H 2.69303509 1.47683771 2.49507587
C -1.30253896 1.65905349 -0.99769419
C -1.60091831 0.38345228 -1.65417008
C -0.84500954 -0.57984743 -1.09937083
H -0.82066535 -1.62830829 -1.37438533
H -2.31519351 0.25979087 -2.46022995
H -1.76453003 2.60443764 -1.26197260
H -0.32737168 -0.42628053 0.96065745

12a

62

C 0.00000000 0.00000000 0.00000000
C -1.03527830 1.06933231 0.21817743
C -1.80066538 1.11927157 1.30626676
C -2.93373404 2.05194969 1.68586736
C -2.63235332 2.40375471 3.16090321
C -1.93591409 1.11486417 3.70952954
C -1.94282965 0.17657695 2.48590901
H -1.25489406 -0.66619045 2.54058858
C -3.41877016 -0.26598588 2.29795699
C -3.51985645 -1.52255027 1.45877441
O -2.60151927 -2.07435402 0.89850654
O -4.77314295 -2.00430158 1.45873010
C -4.98684559 -3.17637088 0.67346564
H -4.71733986 -2.98857406 -0.36978893
H -6.05028312 -3.39811359 0.76466137
H -4.38796281 -4.00765115 1.05364812
H -3.90972303 -0.49028285 3.25075835
C -4.11056313 1.01477503 1.67806505
C -5.30076018 1.48323795 2.50436299
O -5.86424838 0.82438741 3.34846455
O -5.65420168 2.73546412 2.19051204
C -6.78439094 3.26191964 2.89635047
H -7.67749223 2.68624521 2.64592273

H	-6.60405177	3.23179736	3.97342314
H	-6.88591205	4.28939983	2.54894530
S	-4.62789134	0.84670487	-0.09198108
C	-6.30617421	0.10659777	-0.04896301
C	-7.31801887	1.01925455	-0.73733928
O	-7.50472870	2.24439804	-0.06539216
H	-6.62975647	2.64161701	0.06645551
H	-7.02029371	1.18951828	-1.78321977
H	-8.29390613	0.52364582	-0.74511539
H	-6.59883467	-0.08610928	0.98587421
H	-6.26386603	-0.85695416	-0.56551713
H	-2.49395605	0.67010216	4.54027786
C	-0.58855772	1.67018293	4.29160072
C	-1.12956938	2.98784464	4.83815989
C	-1.59283456	3.53257659	3.49065629
C	-0.32173000	3.31212626	2.69211162
C	0.27184041	2.21119182	3.16532259
H	1.14055577	1.70962867	2.75286708
H	-0.03265667	3.88622294	1.81833895
H	-2.01354863	4.53800925	3.45387078
C	-1.22570621	3.47175737	6.07574025
C	-1.84142626	4.81373030	6.38185862
H	-2.68196485	4.70190043	7.07963971
H	-1.11001783	5.47139897	6.86997484
H	-2.20940761	5.32592786	5.48953450
C	-0.71939944	2.72675092	7.28508672
H	0.05813268	3.30758133	7.79864910
H	-1.53005908	2.57219872	8.00932439
H	-0.29898914	1.74977592	7.03513927
H	-0.09606762	0.98584319	4.98336590
H	-3.54344202	2.64632407	3.71490933
H	-3.13910154	2.91400766	1.05186554
C	-1.10055259	2.10703040	-0.87125380
H	-1.35077149	1.64009267	-1.83247794
H	-1.84071489	2.88624668	-0.67546717
H	-0.12136004	2.59008065	-0.99621085
H	1.00462427	0.44447229	-0.04228323
H	-0.01057725	-0.76715476	0.77585734

H   -0.16976534 -0.50293073 -0.96019540

Thermodynamic parameters are given in hartrees.
qG = Free energy (with quasiharmonic correction)
E_{DMSO} = DMSO corrected electronic energy
E_{CORR} = DPLNO-CCSD(T)/def2-TZVPP and DMSO corrected electronic energy

	E	*H*	*G*	*qG*	E_{DMSO}	E_{CORR}
**3a**	-310.732134	-310.566929	-310.608007	-310.608007	-310.807242	-
**trans-7**	-1086.158853	-1085.936801	-1085.997627	-1085.995468	-1086.376031	-
**cis-7**	-1086.160288	-1085.938313	-1085.999046	-1085.996962	-1086.378208	-
**6a:d1**	-1396.926379	-1396.535095	-1396.612543	-1396.609367	-1397.213181	-1395.170605
**6a:d2**	-1396.927180	-1396.535958	-1396.612385	-1396.609775	-1397.213687	-1395.172319
**6a:d3**	-1396.927664	-1396.536591	-1396.613394	-1396.610616	-1397.213777	-1395.173324
**6a:d4**	-1396.929767	-1396.538573	-1396.613874	-1396.611468	-1397.215205	-1395.172076
**TS1-6a:d1**	-1396.876577	-1396.488779	-1396.567815	-1396.564266	-1397.164187	-1395.120261
**TS1-6a:d2**	-1396.873643	-1396.485919	-1396.563991	-1396.560477	-1397.165314	-1395.120108
**TS1-6a:d3**	-1396.872433	-1396.485610	-1396.562701	-1396.559391	-1397.161179	-1395.117349
**TS1-6a:d4**	-1396.872313	-1396.484470	-1396.561682	-1396.558747	-1397.162191	-1395.118051
**TS2-6a:d3**	-1396.869185	-1396.480725	-1396.557721	-1396.554277	-1397.157201	-
**TS3-6a:d3**	-1396.858460	-1396.470833	-1396.544551	-1396.542833	-1397.145164	-
**TS4-6a:d3**	-1396.869200	-1396.481033	-1396.556943	-1396.554558	-1397.159788	-
**TS5-6a:d3**	-1707.634785	-1707.078016	-1707.173218	-1707.168202	-1707.990968	-
**9a**	-1396.901784	-1396.509798	-1396.586852	-1396.582740	-1397.185328	-
**10a**	-1396.894558	-1396.504132	-1396.579259	-1396.576228	-1397.177720	-

**11a**	-1396.892797	-1396.502396	-1396.577436	-1396.574973	-1397.181635	-
**12a**	-1707.704988	-1707.143871	-1707.236718	-1707.232558	-1708.059496	-

9.2 M06-2X Cartesian coordinates and energies

6a:d1

44

C 0.00000000 0.00000000 0.00000000
C -0.88465830 0.33982178 1.18950140
C -2.12538579 0.82204665 1.27428473
C -2.96845329 1.11039962 0.05860803
H -3.88878601 0.51356448 0.07984075
H -2.44478733 0.90315777 -0.87639810
H -3.27332736 2.16500320 0.04815022
C -2.79642808 1.11956496 2.59153570
H -3.11398181 2.16908277 2.63298275
H -2.14635886 0.93206811 3.44828725
H -3.69990467 0.50770102 2.70533605
C 0.16199631 0.03255345 2.24919026
C 0.63674586 -1.33062949 1.77264906
C 0.53256810 -1.35301476 0.43997564
H 0.87110943 -2.12295350 -0.24161547
H 1.07349450 -2.08755553 2.41226533
H -0.11972472 0.11647937 3.29885214
C 1.22093898 1.11005035 1.82038866
C 2.59812629 0.87203639 2.40313608
O 3.18274189 -0.18470812 2.43404286
O 3.10715194 2.00571323 2.91547529
C 4.43146889 1.89298176 3.43599591
H 4.45914126 1.16746909 4.25185057
H 5.11633975 1.57052326 2.64736897
H 4.69422909 2.88706609 3.79431278
H 0.87518667 2.08224646 2.17368691
C 1.14930354 1.04600322 0.24924706
C 2.44481539 0.65158221 -0.44189788

O 2.55184675 -0.21689562 -1.27073211
O 3.46393933 1.43719287 -0.05112449
C 4.73389290 1.11304024 -0.62049260
H 5.42803849 1.85916378 -0.23600691
H 5.03193969 0.10876237 -0.30931400
H 4.68222563 1.15876791 -1.71020013
S 0.61254406 2.61003322 -0.58188156
C 1.31533241 3.95247886 0.43806377
C 0.19903205 4.68805087 1.18310852
O -0.57239505 3.83433202 2.00239749
H -0.96132240 3.14456393 1.43328329
H 0.63784344 5.43663224 1.84977654
H -0.44149180 5.21215229 0.45961338
H 1.84015519 4.63916086 -0.23294451
H 2.05192974 3.53023267 1.12706183
H -0.41424001 0.05550871 -1.00656760

6a:d2

44

C 0.00000000 0.00000000 0.00000000
C 0.06856538 1.19994915 0.92915186
C -0.24873788 2.28550812 0.21607357
C -0.52023441 1.83309955 -1.21139899
C -1.09921523 0.45044387 -0.94878973
C -2.14753664 -0.19854677 -1.45587096
C -3.09495338 0.43460368 -2.44162526
H -4.11737383 0.43369609 -2.04395021
H -3.11529442 -0.14126055 -3.37510377
H -2.82544513 1.46488420 -2.68245709
C -2.48060274 -1.62106452 -1.08533039
H -2.51580392 -2.24761679 -1.98584446
H -1.75439677 -2.05799222 -0.39694778
H -3.47375075 -1.67497155 -0.62214812
C 0.84761834 1.39589441 -1.82802749
C 1.88493193 2.49490764 -1.81789873
O 1.75213132 3.58650152 -1.31948974
O 2.98530138 2.12735657 -2.49496557

C 4.05025473 3.07794116 -2.48879918
H 3.72589029 4.01719127 -2.94183007
H 4.37487921 3.26779030 -1.46190852
H 4.85186518 2.62630621 -3.07230141
H 0.71581484 1.09967910 -2.87366640
C 1.20795523 0.09899795 -1.01098295
C 1.21552404 -1.11743308 -1.93366798
O 1.44759679 -1.07822835 -3.11855364
O 0.94755115 -2.25363894 -1.27579682
C 0.98910117 -3.44355007 -2.07500814
H 2.00206647 -3.59153403 -2.45400881
H 0.70436716 -4.25242429 -1.40353287
H 0.28279374 -3.35908701 -2.90347355
S 2.76888327 0.13461509 -0.03732247
C 4.08220645 -0.32069738 -1.23500793
C 4.71114551 -1.65783610 -0.85721566
O 3.78062049 -2.71794737 -0.89974060
H 3.05244332 -2.49600875 -0.29389952
H 5.18014462 -1.59001599 0.13508435
H 5.49175898 -1.90621612 -1.58275501
H 4.83246208 0.47608208 -1.22084856
H 3.65179492 -0.36191565 -2.23833115
H -1.07594269 2.52616200 -1.84235226
H -0.22139822 3.31966602 0.53486266
H 0.40530019 1.15926829 1.95880391
H -0.06901261 -0.97907901 0.46815353

6a:d3

44

C 0.00000000 0.00000000 0.00000000
C -1.23334600 -0.51848400 0.71952700
C -2.51943000 -0.18499200 0.62945400
C -3.57484900 -0.77837000 1.52347300
H -4.38891300 -1.20485200 0.92427900
H -3.18007700 -1.55163500 2.18351900
H -4.00767000 0.00503100 2.15750500
C -3.02531300 0.84384300 -0.34808200

H	-3.50508500	1.66868700	0.19349200
H	-2.23269000	1.26543500	-0.97003800
H	-3.78546500	0.40748900	-1.00773600
C	-0.48130400	-1.43879700	1.66904000
C	0.43185200	-2.15881800	0.69069500
C	0.71733800	-1.30736300	-0.30123300
H	1.40075800	-1.45272600	-1.13028800
H	0.83521000	-3.15454400	0.84112900
H	-1.05658300	-2.05488700	2.35791500
C	0.41582100	-0.38083500	2.39024800
C	-0.32433000	0.23589800	3.55786800
O	-1.48497200	0.04754700	3.83113800
O	0.48959100	1.00439800	4.30036200
C	-0.13919800	1.68093300	5.38871900
H	-0.93975000	2.32607300	5.01573000
H	0.64561600	2.26910400	5.86323900
H	-0.56017600	0.96045500	6.09342700
H	1.34592600	-0.80668900	2.77858900
C	0.75933400	0.63183000	1.23302800
C	2.25760100	0.72679900	0.99585400
O	3.10136000	0.50162200	1.82993500
O	2.53907600	1.10907300	-0.25798900
C	3.93485900	1.27580400	-0.54338900
H	4.34261800	2.06803200	0.08712400
H	4.46539500	0.33822300	-0.36457700
H	3.98355700	1.55441600	-1.59505600
S	0.07119700	2.33340500	1.43378900
C	1.28528800	3.21292400	2.49167800
C	1.94746600	4.34491000	1.71236800
O	2.67991400	3.87326000	0.60272200
H	2.06714600	3.37387900	0.03534400
H	1.19309000	5.08079700	1.39820600
H	2.66663800	4.85524600	2.36031300
H	2.02965900	2.49784600	2.85023000
H	0.74702700	3.60623500	3.35966700
H	-0.11263600	0.69651400	-0.82893400

6a:d4

44

C	0.00000000	0.00000000	0.00000000
C	0.42070900	-0.11465900	1.45455200
C	0.34372600	1.10448400	1.99811800
C	-0.08010200	2.06752500	0.90032300
C	-0.98323500	1.15841800	0.08254600
C	-2.18001700	1.34517600	-0.47257400
C	-2.93955200	2.63868000	-0.33843300
H	-3.91240700	2.46863000	0.13937900
H	-3.13948100	3.05957500	-1.33227000
H	-2.39441600	3.38753200	0.23867700
C	-2.87536500	0.28650300	-1.28896100
H	-3.05181400	0.64931100	-2.31030900
H	-3.85863900	0.05860600	-0.85884400
H	-2.30225500	-0.64008800	-1.35300500
C	1.18151300	2.14784000	-0.03657200
C	1.21801900	3.37207200	-0.92214600
O	2.23594500	3.97249000	-1.20726700
O	0.00797100	3.76880700	-1.31919600
C	-0.00261300	4.83117500	-2.27528500
H	0.47240700	5.72235200	-1.86013100
H	0.53219100	4.51262400	-3.17326300
H	-1.05314800	5.01854300	-2.49326500
H	2.09939100	2.17652300	0.55296100
C	1.12468300	0.76789100	-0.79386000
C	0.73626500	0.89253000	-2.26374200
O	1.10601700	1.78322000	-2.99810500
O	-0.01164200	-0.13018600	-2.69005300
C	-0.34673900	-0.10765800	-4.07795000
H	0.55966200	-0.15938300	-4.68529600
H	-0.97169900	-0.98438800	-4.24147100
H	-0.89178700	0.80787800	-4.31914100
S	2.69079800	-0.18937100	-0.76136300
C	3.87319900	0.89815200	-1.64102300
C	4.88349900	1.59806300	-0.73449400
O	4.31327400	2.54606500	0.13349900
H	3.81877000	3.20358000	-0.39094800

H 5.39303500 0.86679100 -0.09999300
H 5.64208700 2.06515600 -1.38259400
H 3.30817100 1.62952600 -2.22884400
H 4.40706700 0.25619200 -2.34978900
H -0.46189800 3.03769100 1.21608200
H 0.64562800 1.40412200 2.99579500
H 0.78869700 -1.02295000 1.91749400
H -0.30610600 -0.91954200 -0.49282700

TS1-6a:d1

44

C 0.00000000 0.00000000 0.00000000
C -0.61303700 -0.56093500 -1.24965800
C -0.54773500 0.07865800 -2.43715900
C -1.19335300 -0.28458100 -3.68778700
C -0.66674900 0.48986200 -4.70761600
C 0.16692400 1.47481100 -4.15034200
C 0.10717700 1.38311300 -2.73918500
H 0.87392800 1.83421300 -2.11822700
H 0.67150700 2.26301300 -4.69376700
H -0.92639900 0.41352300 -5.75682600
H -1.91015800 -1.08733000 -3.81416900
C -1.32941000 -1.86650400 -1.06252600
H -1.82310500 -2.21839200 -1.96877100
H -0.62407000 -2.63836900 -0.73052200
H -2.08632200 -1.76027200 -0.27648500
H 0.58975300 0.90108800 -0.17557400
H -0.80037500 0.24186800 0.71252900
H 0.64213700 -0.74447200 0.48537500
C -4.49002300 2.23004600 -5.93142100
O -4.13023800 1.83007000 -4.61053100
C -2.94319900 2.31396000 -4.16718900
C -2.59812800 1.94662700 -2.80700800
C -1.41064600 2.50491100 -2.28692700
C -0.95911300 3.88813000 -2.72150800
O -1.96329500 4.76328800 -2.57743300
C -1.66892300 6.09258200 -3.01135200

H -2.58035100 6.66425200 -2.84302400
H -1.40872100 6.08087500 -4.07236200
H -0.83850100 6.50771200 -2.43596100
O 0.15811400 4.20448900 -3.05108500
H -1.31289000 2.39915000 -1.20415200
S -3.65212500 0.95892600 -1.79145300
C -4.65688600 2.25892900 -0.97602400
C -5.47252200 3.08135600 -1.96880600
O -6.33775900 2.28972500 -2.74815200
H -5.77354900 1.73174100 -3.31100400
H -4.79192900 3.67095800 -2.60171700
H -6.10438000 3.78355500 -1.41483900
H -3.99855600 2.90922000 -0.38922900
H -5.32383600 1.71868100 -0.29582200
O -2.22693400 3.00899000 -4.87647900
H -5.47084700 1.79212200 -6.11425800
H -3.76062600 1.85696200 -6.65455300
H -4.53775600 3.31929900 -5.99992300

TS1-6a:d2

44

C 0.00000000 0.00000000 0.00000000
C -0.61896700 -0.80192100 -1.10531900
C -1.60923600 -0.31895200 -1.88591900
C -2.37814700 -1.01484300 -2.89802300
C -3.06662400 -0.07524700 -3.66059400
C -2.93619700 1.18049000 -3.05759400
C -2.23855100 1.03030000 -1.82913000
H -1.77659800 1.88575000 -1.34672500
H -3.40883700 2.09743300 -3.38477100
H -3.67452000 -0.29722000 -4.52911400
H -2.34519900 -2.07940400 -3.09862900
C -0.06567400 -2.18780600 -1.26222300
H -0.68478900 -2.82660700 -1.89458100
H 0.93953600 -2.14285700 -1.70038200
H 0.03915300 -2.66286900 -0.27986600
H 1.09425600 -0.03966200 -0.05432400

H -0.30948800 1.04598200 0.00070700
H -0.29003000 -0.44559600 0.96182500
C -2.41809000 -3.02913400 1.15891000
O -2.79095500 -1.81034900 0.52086600
C -3.81057200 -1.92424300 -0.37433000
C -4.23436200 -0.64687000 -0.93367400
C -3.60415300 0.56739100 -0.56443300
C -4.43749600 1.83062200 -0.48041200
O -5.36691700 1.70143300 0.47786200
C -6.21505600 2.83879200 0.66006200
H -6.90674900 2.56299900 1.45477100
H -6.75300200 3.05600500 -0.26590900
H -5.62385000 3.71132700 0.94615300
O -4.28221900 2.84780300 -1.11143600
H -2.95831600 0.48214000 0.31214500
S -5.62766100 -0.64020700 -2.02603200
C -7.07620700 -0.64851900 -0.89909400
C -7.12001400 -1.83639600 0.05703900
O -7.12704600 -3.07612800 -0.59911900
H -6.21582800 -3.24424200 -0.90278900
H -8.05062400 -1.77465900 0.63335700
H -6.28594600 -1.76436400 0.77216900
H -7.92960600 -0.69853800 -1.58385500
H -7.11863300 0.29012500 -0.34067000
O -4.29718300 -3.01953800 -0.61798700
H -3.25931900 -3.43460300 1.72631300
H -1.59588200 -2.77207600 1.82829100
H -2.10091900 -3.77132200 0.42210600

TS1-6a:d3

44

C 0.00000000 0.00000000 0.00000000
C -1.29939000 0.62935300 -0.41192400
C -2.15680700 1.18084700 0.46894800
C -3.45963700 1.80818800 0.22314600
C -3.71034700 2.68353700 1.30262900
C -2.85447500 2.35962100 2.34215200

C -2.05441000 1.25186800 1.94326200
H -1.18423500 0.93354900 2.50831500
H -2.88875400 2.76992300 3.34489800
H -4.52941800 3.39244800 1.35105600
H -3.95908900 1.86400200 -0.73758200
C -1.56079800 0.59951600 -1.88997700
H -2.50895600 1.06030700 -2.16894100
H -0.75142400 1.10832500 -2.42787800
H -1.57581800 -0.44255900 -2.23021800
H 0.17605500 0.04129100 1.07607700
H 0.00143000 -1.05321900 -0.30305300
H 0.83681700 0.49413600 -0.50883000
C -6.89395900 1.68013000 3.16482000
O -5.67639800 1.04486600 2.77572300
C -5.66782300 0.53916800 1.52489800
C -4.36063800 -0.06407400 1.15268100
C -3.40457100 -0.24861400 2.19495900
H -3.74468100 -0.00712700 3.19479800
C -2.41225400 -1.35959300 2.21208800
O -1.97304800 -1.98364800 1.27445600
O -1.99472000 -1.57348200 3.48055900
C -1.00231100 -2.58769300 3.62944800
H -1.38331600 -3.54857100 3.27607900
H -0.78252000 -2.62969700 4.69517600
H -0.10530700 -2.33025300 3.06004000
S -4.36744700 -0.96385300 -0.38877800
C -4.96306000 -2.60719100 0.13396400
C -6.35314400 -2.59195300 0.76363300
O -7.33603700 -2.05345100 -0.08081900
H -7.17285400 -1.09477100 -0.14757900
H -6.31984200 -2.06111400 1.72938000
H -6.65022700 -3.62535000 0.97443800
H -4.22561000 -3.06237500 0.80330700
H -4.99037300 -3.18618100 -0.79530700
O -6.64896200 0.60513300 0.80860800
H -7.72661600 0.97653500 3.10091100
H -7.10012000 2.53477000 2.51534800
H -6.73896400 2.00547200 4.19268600

TS1-6a:d4

44

C	0.00000000	0.00000000	0.00000000
C	-0.84716900	-1.10706200	0.55092800
C	-1.71001600	-1.82956400	-0.19441300
C	-2.53001400	-2.95501400	0.23120400
C	-2.86124000	-3.70028700	-0.90429200
C	-2.53121400	-2.95747700	-2.03862500
C	-2.03779200	-1.69186600	-1.63334100
H	-1.52739200	-1.00622800	-2.29954000
H	-2.75509100	-3.23918700	-3.06166500
H	-3.38352700	-4.64923900	-0.90093000
H	-2.67160500	-3.28803700	1.25273000
C	-0.64759200	-1.36884000	2.01716800
H	-1.42865800	-1.99360100	2.45448600
H	0.32045000	-1.85681900	2.18743800
H	-0.61923000	-0.41650900	2.56012400
H	-0.35455500	0.95929100	0.39980200
H	1.03868300	-0.12766800	0.32880900
H	-0.03162300	0.06835700	-1.08596200
C	-3.29424100	0.84288300	2.51227300
O	-3.36874500	0.19636200	1.24364500
C	-4.30996400	-0.77200500	1.14159900
C	-4.41509100	-1.32857200	-0.21355500
C	-3.78765700	-0.73434500	-1.33051600
H	-4.26069700	-0.96446000	-2.28452500
C	-3.37743600	0.72291800	-1.30453500
O	-2.28157900	1.17908800	-1.52368500
O	-4.45790100	1.48954300	-1.08547500
C	-4.20343600	2.89371600	-1.02577300
H	-3.50881700	3.11029700	-0.20991200
H	-5.17041600	3.35953300	-0.84246400
H	-3.77482400	3.24578600	-1.96662900
S	-5.68478300	-2.53572800	-0.48373800
C	-7.10247500	-1.45446100	-0.89718200
C	-7.49401700	-0.49894300	0.22834800

O   -7.78954100 -1.15718900 1.43229600
H   -6.94394900 -1.45150700 1.81777700
H   -8.40237000 0.03500600 -0.07321000
H   -6.70112100 0.25249800 0.36465300
H   -7.92509800 -2.14799500 -1.10152000
H   -6.88080100 -0.89341400 -1.81277400
O   -4.98536800 -1.09563700 2.10401900
H   -2.49404800 1.57801500 2.41970000
H   -3.06623400 0.11973900 3.29909100
H   -4.24359000 1.33222700 2.74429700

Thermodynamic parameters are given in hartrees.
qG = Free energy (with quasiharmonic correction)
E_{DMSO} = DMSO corrected electronic energy
E_{CORR} = DPLNO-CCSD(T)/def2-TZVPP and DMSO corrected electronic energy

	E	*H*	*G*	*qG*	*E_{DMSO}*	*E_{CORR}*
**6a:d1**	-1396.790452	-1396.399097	-1396.475880	-1396.472813	-1397.107597	-1395.170700
**6a:d2**	-1396.792503	-1396.401290	-1396.477456	-1396.474867	-1397.109096	-1395.171483
**6a:d3**	-1396.793830	-1396.402722	-1396.478439	-1396.476115	-1397.109906	-1395.172432
**6a:d4**	-1396.793851	-1396.402221	-1396.477269	-1396.475029	-1397.109248	-1395.171816
**TS1-6a:d1**	-1396.744491	-1396.356863	-1396.435354	-1396.432137	-1397.062576	-1395.120629
**TS1-6a:d2**	-1396.740942	-1396.353271	-1396.431047	-1396.427526	-1397.062670	-1395.119959
**TS1-6a:d3**	-1396.742503	-1396.354527	-1396.432072	-1396.428703	-1397.061411	-1395.120750
**TS1-6a:d4**	-1396.740888	-1396.353386	-1396.430366	-1396.427444	-1397.060530	-1395.118786

9.3 B3LYP Cartesian coordinates and energies

6a:d1
44

C   0.00000000 0.00000000 0.00000000
C   0.94116800 -0.45977000 1.10807200
C   2.20733800 -0.88910900 1.09451300
C   3.02057100 -1.00522200 -0.17410700
H   2.47324700 -0.68647000 -1.06508600
H   3.34167400 -2.04475900 -0.33148500

H	3.93544100	-0.39907400	-0.10267500
C	2.94251600	-1.30375500	2.34829700
H	3.85024900	-0.69837300	2.48635400
H	3.27099000	-2.35021200	2.27242700
H	2.33483000	-1.20890800	3.25228100
C	-0.06397400	-0.27276600	2.24354000
C	-0.53453300	1.14459900	1.94083900
C	-0.48348900	1.31376900	0.61055900
H	-0.80857000	2.17357100	0.03547500
H	-0.90364600	1.84403600	2.68251900
H	0.26485000	-0.46754000	3.26541900
C	-1.14308200	-1.31130600	1.74547200
C	-2.45928500	-1.36520600	2.50194500
O	-3.01322600	-2.41486900	2.80098900
O	-2.93032400	-0.16907000	2.87982400
C	-4.21218700	-0.17231400	3.54002200
H	-4.96689500	-0.60080200	2.87572100
H	-4.16373300	-0.75175000	4.46589700
H	-4.42919100	0.87576000	3.74998100
H	-0.71410900	-2.30665100	1.86919000
C	-1.19801000	-1.02929700	0.18749800
C	-2.53124600	-0.52411900	-0.34652600
O	-3.61900600	-0.87541500	0.07608300
O	-2.38498300	0.31058200	-1.39337500
C	-3.60119500	0.73954400	-2.03445500
H	-4.25544100	1.23993300	-1.31543700
H	-3.28480600	1.42982500	-2.81727800
H	-4.12402300	-0.11902400	-2.46548500
S	-0.81056700	-2.53583500	-0.85410800
C	-2.14249100	-3.75289800	-0.45094900
C	-1.73313100	-4.90445700	0.47451200
O	-1.37304000	-4.52453700	1.78920000
H	-2.09453000	-3.99829000	2.19130400
H	-2.57445300	-5.62009900	0.49206400
H	-0.86366200	-5.43021100	0.06504300
H	-2.45204500	-4.17279600	-1.41639200
H	-3.00209600	-3.20576800	-0.05056100
H	0.36579500	0.04941000	-1.02457800

6a:d2

44

C	0.00000000	0.00000000	0.00000000
C	0.06630200	1.16059700	0.98721500
C	-0.21745000	2.28477200	0.31204600
C	-0.46936700	1.89470900	-1.14176300
C	-1.09241400	0.51752600	-0.93203300
C	-2.21365000	-0.04576200	-1.39804800
C	-3.16763400	0.67995800	-2.31964800
H	-4.16483800	0.75683900	-1.86244800
H	-3.29555400	0.12596500	-3.26068300
H	-2.83416500	1.69145200	-2.56708800
C	-2.64345900	-1.44787200	-1.03011600
H	-2.78634500	-2.05815100	-1.93348800
H	-1.92336200	-1.96045300	-0.38688800
H	-3.61143900	-1.43044700	-0.50841800
C	0.90144700	1.45699700	-1.77221100
C	1.93467600	2.57020000	-1.77976200
O	1.79429600	3.66426100	-1.27144800
O	3.02044500	2.23291700	-2.51048900
C	4.03691000	3.24626900	-2.62994500
H	3.63169500	4.13257500	-3.12593800
H	4.41436300	3.52663400	-1.64243300
H	4.82524600	2.79514200	-3.23426600
H	0.75182000	1.17825800	-2.82049000
C	1.24496600	0.12231900	-0.98774900
C	1.28838700	-1.07026700	-1.95154400
O	1.48233600	-0.98591200	-3.14814100
O	1.09875000	-2.24492700	-1.31797300
C	1.16282900	-3.42720500	-2.14690900
H	2.16140900	-3.52426100	-2.57845900
H	0.95429600	-4.25799000	-1.47180800
H	0.41207800	-3.37063000	-2.93883600
S	2.79117200	0.12738700	0.05478400
C	4.18839800	-0.31633700	-1.07891600
C	4.90675000	-1.57182600	-0.57943500

O 4.08820500 -2.73292600 -0.61151700
H 3.27101700 -2.52516200 -0.12283800
H 5.30384300 -1.40425400 0.43439700
H 5.75677300 -1.78327400 -1.23873400
H 4.88138000 0.53136200 -1.10132700
H 3.80515900 -0.46251300 -2.09154400
H -1.00349900 2.62642800 -1.74858300
H -0.18858800 3.30464700 0.67632500
H 0.37178100 1.06782300 2.02416700
H -0.09372600 -0.99648400 0.42563000

6a:d3

44

C 0.00000000 0.00000000 0.00000000
C -1.26091500 -0.48106200 0.70962300
C -2.55566000 -0.20720900 0.51712100
C -3.65334500 -0.83518200 1.34330900
H -4.35524000 -1.38280200 0.69689500
H -3.27386700 -1.52457300 2.10028200
H -4.23478600 -0.06042900 1.86198100
C -3.04170100 0.75381300 -0.54374500
H -3.64774000 1.54934200 -0.08774100
H -2.22838900 1.22968800 -1.09846100
H -3.69028200 0.23937800 -1.26789700
C -0.53642100 -1.39579700 1.69364700
C 0.34928000 -2.17116900 0.72432800
C 0.66701900 -1.34481800 -0.28467600
H 1.33809900 -1.53143500 -1.11660000
H 0.70732100 -3.18327400 0.88868100
H -1.13044400 -1.98445900 2.39111400
C 0.40981000 -0.36077100 2.40748500
C -0.26654000 0.25883300 3.62151900
O -1.42591500 0.09797100 3.94323300
O 0.61389300 0.96261900 4.36853500
C 0.08166800 1.56797500 5.56171100
H -0.76195600 2.21874300 5.31546500
H 0.90643400 2.14143100 5.98751500

H -0.25077100 0.79714600 6.26301800
H 1.32872900 -0.82991100 2.77192400
C 0.79242800 0.63020400 1.22939700
C 2.30151600 0.67284000 0.99390400
O 3.14087800 0.38743700 1.82453600
O 2.60487300 1.08612900 -0.25335700
C 4.01310000 1.20995000 -0.55368600
H 4.45039200 2.00696400 0.05226500
H 4.52552000 0.26439700 -0.35898100
H 4.05646600 1.46556200 -1.61301600
S 0.15544800 2.38101100 1.40094500
C 1.40509600 3.29374700 2.41978500
C 1.92840900 4.51174300 1.65506600
O 2.64815200 4.16540600 0.47908300
H 2.07112100 3.58935300 -0.05473800
H 1.09909100 5.19576100 1.41545800
H 2.63594600 5.05547900 2.29164300
H 2.22261000 2.61845900 2.68153500
H 0.91764100 3.61100300 3.34742100
H -0.08551500 0.68447500 -0.84160600

6a:d4
44

C 0.00000000 0.00000000 0.00000000
C 0.41747300 -0.12043400 1.46269500
C 0.35530000 1.10487300 2.00536500
C -0.06464700 2.07196700 0.90199000
C -0.99150200 1.15639700 0.10923900
C -2.25514300 1.29398400 -0.30852800
C -3.06396700 2.54464800 -0.05291400
H -3.93632600 2.31960300 0.57790600
H -3.45459000 2.95253500 -0.99621600
H -2.48363300 3.33081300 0.43627600
C -2.99837600 0.20395900 -1.04612600
H -3.38795000 0.57935900 -2.00355300
H -3.87000200 -0.13090500 -0.46449000
H -2.37543000 -0.66883200 -1.25616700

C 1.20201200 2.15007000 -0.04689800
C 1.28316400 3.39385800 -0.91467900
O 2.32630700 3.99508500 -1.13147600
O 0.09249900 3.82701100 -1.35356800
C 0.11638000 4.97774000 -2.22099300
H 0.52491700 5.84375900 -1.69274700
H 0.72451700 4.76432000 -3.10363300
H -0.92447100 5.15022700 -2.49746500
H 2.11256800 2.17077200 0.55275900
C 1.14870900 0.75200200 -0.79508800
C 0.84246400 0.84569100 -2.28947600
O 1.27267400 1.71905200 -3.02278500
O 0.10346400 -0.18510500 -2.73978500
C -0.11750200 -0.22871100 -4.16221200
H 0.83447600 -0.33575200 -4.68980300
H -0.74884100 -1.10266000 -4.32699600
H -0.61964700 0.68226900 -4.49862900
S 2.72068700 -0.24662900 -0.68806800
C 4.00382900 0.77684600 -1.53617700
C 4.97576900 1.52190700 -0.61290900
O 4.39402300 2.52637700 0.19635800
H 3.89195200 3.15565000 -0.36143800
H 5.45496800 0.81920500 0.07739200
H 5.76757900 1.94744800 -1.25479000
H 3.50379300 1.46408700 -2.22627800
H 4.57873600 0.06991700 -2.14761000
H -0.43779200 3.04427000 1.22339600
H 0.65323700 1.40371700 3.00590000
H 0.76726800 -1.03519500 1.92911000
H -0.31606100 -0.91901700 -0.48788000

TS1-6a:d1

44

C 0.00000000 0.00000000 0.00000000
C 0.94305500 -0.44699200 1.10307500
C 2.18931600 -0.91492000 1.07278600
C 2.96602700 -1.07504600 -0.20785900

H	2.40165300	-0.75769900	-1.08693700
H	3.24518700	-2.12653900	-0.34819500
H	3.89731700	-0.49642500	-0.16893200
C	2.92674200	-1.34719600	2.31315000
H	3.86211200	-0.78390200	2.41926800
H	3.19747300	-2.40776600	2.24175500
H	2.34092100	-1.20794700	3.22393600
C	-0.05576500	-0.27386400	2.23959100
C	-0.56889600	1.12390200	1.93417900
C	-0.51921800	1.29505700	0.60833600
H	-0.87531200	2.14107000	0.03236900
H	-0.97031200	1.80724900	2.67276100
H	0.27991200	-0.45916300	3.25996000
C	-1.09960700	-1.33200600	1.73939700
C	-2.42602400	-1.39177600	2.46291400
O	-2.99978500	-2.43385300	2.71795300
O	-2.89410600	-0.20278200	2.83812500
C	-4.21473800	-0.21287200	3.38640400
H	-4.90963500	-0.61134900	2.64361900
H	-4.24751300	-0.82681900	4.28865400
H	-4.44437700	0.82645400	3.61598300
H	-0.65549100	-2.32152000	1.86584300
C	-1.16498800	-1.04675300	0.19493800
C	-2.49832800	-0.52970800	-0.31356200
O	-3.57809700	-0.84454700	0.13802100
O	-2.35783400	0.26996000	-1.37699200
C	-3.57702400	0.70743200	-1.97898100
H	-4.17048100	1.27680300	-1.26006600
H	-3.28039400	1.33399100	-2.81846700
H	-4.15558400	-0.15235400	-2.32420700
S	-0.77784500	-2.52313700	-0.83561500
C	-2.11615900	-3.69877100	-0.40894300
C	-1.67568600	-4.87003700	0.46670800
O	-1.28138400	-4.49942500	1.76451100
H	-2.01092200	-4.01283600	2.19226300
H	-2.50852200	-5.59038600	0.50008000
H	-0.81644600	-5.37615500	0.01651000
H	-2.49994700	-4.08360800	-1.35978800

H -2.93229600 -3.14468800 0.06785700
H 0.36097500 0.05277800 -1.02624600

TS1-6a:d2

44

C 0.00000000 0.00000000 0.00000000
C -0.54540400 -0.83603100 -1.12501000
C -1.59858600 -0.44173200 -1.89090800
C -2.22209700 -1.15273500 -2.98619600
C -3.00804200 -0.26523400 -3.71776300
C -3.02514100 0.97703400 -3.06338200
C -2.35467200 0.85061100 -1.80783900
H -1.93030500 1.73342800 -1.33919400
H -3.56511700 1.85931300 -3.38125600
H -3.55198500 -0.50890300 -4.62320600
H -2.05653200 -2.19277800 -3.24056600
C 0.18479500 -2.13278900 -1.34978100
H -0.24817500 -2.74348000 -2.14446800
H 1.23583800 -1.93779400 -1.60717100
H 0.19633900 -2.72631600 -0.42531700
H 1.05587800 0.24816800 -0.17998700
H -0.54611200 0.93304100 0.15169300
H -0.03089600 -0.57141900 0.93826000
C -2.56085500 -3.16846000 1.33938400
O -2.91913500 -1.96530900 0.64439400
C -3.97329600 -2.08249400 -0.23039400
C -4.36330800 -0.82188300 -0.84239200
C -3.69349300 0.40158900 -0.53228700
C -4.49890900 1.68881500 -0.40372600
O -5.38576900 1.57227000 0.60733800
C -6.16085700 2.75189600 0.90499200
H -6.83492300 2.45510500 1.70941700
H -6.72343900 3.07105800 0.02363400
H -5.50549600 3.56485300 1.23050300
O -4.34696000 2.71485700 -1.03426000
H -3.04286500 0.30569600 0.33905400
S -5.70292200 -0.85635600 -2.01364000

C -7.25359800 -0.71771500 -1.00380800
C -7.42101100 -1.78584000 0.08174000
O -7.38699500 -3.11191400 -0.40345200
H -6.45187300 -3.31952000 -0.60708900
H -8.40844900 -1.64446500 0.54143700
H -6.66730600 -1.63138100 0.86927700
H -8.04249300 -0.82525600 -1.75772300
H -7.32411800 0.28044100 -0.55964100
O -4.51965100 -3.17398200 -0.39674400
H -3.41309600 -3.55767000 1.90388100
H -1.75463900 -2.88200600 2.01850100
H -2.21963800 -3.93755900 0.63933200

TS1-6a:d3
44

C 0.00000000 0.00000000 0.00000000
C -1.28953100 0.70744600 -0.30744000
C -2.08917900 1.26397500 0.64447200
C -3.30521000 2.01899900 0.43254300
C -3.53924700 2.82849200 1.55123700
C -2.63549600 2.47832600 2.55314000
C -1.89185000 1.31898200 2.13203000
H -0.92918300 1.09211300 2.57814000
H -2.61381600 2.89243700 3.55598300
H -4.32949500 3.56606500 1.63677200
H -3.88026300 2.04956600 -0.48470700
C -1.60932400 0.79296800 -1.77766800
H -2.58750700 1.22689900 -1.99108300
H -0.84748600 1.39336000 -2.29573400
H -1.57525500 -0.21013000 -2.22217800
H 0.25639200 -0.00348000 1.05835500
H -0.06856600 -1.04742500 -0.32686800
H 0.82200700 0.45360600 -0.57237500
C -6.68557400 1.62821800 3.61351300
O -5.42360500 1.02680900 3.29007900
C -5.39566800 0.28898700 2.13256200
C -4.09031400 -0.29945700 1.87163200

C -2.98033700 -0.07214800 2.76075300
H -3.30060600 0.31606000 3.72771400
C -2.02028200 -1.22535400 3.02437800
O -0.82152200 -1.24887100 2.83205500
O -2.68080000 -2.24047000 3.62255000
C -1.87477100 -3.36825600 4.02121100
H -1.42118500 -3.83948600 3.14488000
H -2.56584200 -4.05300500 4.51436900
H -1.08790700 -3.04866900 4.70964300
S -3.91839900 -1.35289100 0.44504300
C -4.63273100 -2.99033500 0.93834100
C -6.11575000 -2.98741700 1.32759600
O -6.96566300 -2.42619800 0.34963900
H -6.86634900 -1.45540200 0.42208700
H -6.24377600 -2.48389400 2.29749100
H -6.42739600 -4.03263300 1.45721500
H -4.03520800 -3.42205900 1.74795900
H -4.48716500 -3.59152500 0.03231000
O -6.41447000 0.15475500 1.45338400
H -7.46694800 0.86780700 3.69914900
H -6.97813400 2.35121700 2.84528500
H -6.53052300 2.12914300 4.57120200

TS1-6a:d4

44

C 0.00000000 0.00000000 0.00000000
C -0.76368000 -1.18743600 0.51553700
C -1.69495100 -1.85944500 -0.21525800
C -2.39843500 -3.06811000 0.15998300
C -2.83288200 -3.71600400 -1.00178300
C -2.60683700 -2.87655100 -2.09541000
C -2.14119200 -1.60486300 -1.62207500
H -1.60981200 -0.92149100 -2.27571800
H -2.89100800 -3.08869200 -3.12100400
H -3.31744300 -4.68495000 -1.03577100
H -2.46361500 -3.48026500 1.15940500
C -0.36820800 -1.61975200 1.90371900

H	-0.99404200	-2.41579300	2.31160400
H	0.67397600	-1.97080200	1.91006300
H	-0.41012000	-0.76295900	2.58977400
H	-0.15303300	0.86013400	0.66715600
H	1.07878600	-0.21476200	0.01365300
H	-0.29344600	0.30438400	-1.00341100
C	-3.37718800	0.92231200	2.53466800
O	-3.42883900	0.26682500	1.25817000
C	-4.42780500	-0.65880600	1.11054000
C	-4.51607500	-1.19459600	-0.24101600
C	-3.79536100	-0.66297000	-1.36033300
H	-4.27554200	-0.91209100	-2.30804300
C	-3.42981500	0.81625800	-1.43131500
O	-2.35972900	1.28859400	-1.75821300
O	-4.52537600	1.56980300	-1.20551400
C	-4.34665600	2.99472700	-1.32776800
H	-3.57833300	3.34077900	-0.63119100
H	-5.31688000	3.42626600	-1.07882700
H	-4.05677700	3.25806800	-2.34905100
S	-5.74871800	-2.44487800	-0.53530000
C	-7.24136500	-1.46802600	-1.03404700
C	-7.72601100	-0.46298700	0.01900300
O	-7.98060800	-1.03591100	1.28541100
H	-7.11346200	-1.22980400	1.69618000
H	-8.67700200	-0.03837400	-0.32909900
H	-7.00436200	0.36460700	0.09369500
H	-8.00419600	-2.23782400	-1.20146300
H	-7.05242300	-0.95319900	-1.98510700
O	-5.16786200	-0.93848200	2.05317200
H	-2.56031700	1.64309400	2.45952900
H	-3.18291400	0.20059800	3.33350100
H	-4.32165900	1.43380100	2.74275400

Thermodynamic parameters are given in hartrees.
qG = Free energy (with quasiharmonic correction)
E_{DMSO} = DMSO corrected electronic energy
E_{CORR} = DPLNO-CCSD(T)/def2-TZVPP and DMSO corrected electronic energy

	E	*H*	*G*	*qG*	E_{DMSO}	E_{CORR}
**6a:d1**	-1396.592411	-1396.205401	-1396.280866	-1396.278786	-1396.889447	-1395.170537
**6a:d2**	-1396.589464	-1396.202928	-1396.279828	-1396.276894	-1396.887891	-1395.170492
**6a:d3**	-1396.589790	-1396.203156	-1396.279989	-1396.277177	-1396.888251	-1395.171798
**6a:d4**	-1396.591446	-1396.204670	-1396.280013	-1396.277728	-1396.888720	-1395.170067
**TS1-6a:d1**	-1396.557780	-1396.174622	-1396.255023	-1396.250995	-1396.858315	-1395.116908
**TS1-6a:d2**	-1396.554052	-1396.170711	-1396.250093	-1396.246203	-1396.858127	-1395.115474
**TS1-6a:d3**	-1396.552887	-1396.169463	-1396.248141	-1396.244610	-1396.854497	-1395.112796
**TS1-6a:d4**	-1396.552887	-1396.168345	-1396.248672	-1396.244335	-1396.854089	-1395.113453

10. References – Part 2

(1) Barca, G. M. J.; Bertoni, C.; Carrington, L.; Datta, D.; De Silva, N.; Deustua, J. E.; Fedorov, D. G.; Gour, J. R.; Gunina, A. O.; Guidez, E.; Harville, T.; Irle, S.; Ivanic, J.; Kowalski, K.; Leang, S. S.; Li, H.; Li, W.; Lutz, J. J.; Magoulas, I.; Mato, J.; Mironov, V.; Nakata, H.; Pham, B. Q.; Piecuch, P.; Poole, D.; Pruitt, S. R.; Rendell, A. P.; Roskop, L. B.; Ruedenberg, K.; Sattasathuchana, T.; Schmidt, M. W.; Shen, J.; Slipchenko, L.; Sosonkina, M.; Sundriyal, V.; Tiwari, A.; Galvez Vallejo, J. L.; Westheimer, B.; Włoch, M.; Xu, P.; Zahariev, F.; Gordon, M. S. Recent Developments in the General Atomic and Molecular Electronic Structure System. *J. Chem. Phys.* **2020**, *152* (15). https://doi.org/10.1063/5.0005188.

(2) Neese, F. The ORCA Program System. *Wiley Interdiscip. Rev. Comput. Mol. Sci.* **2012**, *2* (1), 73–78. https://doi.org/10.1002/wcms.81.

(3) Neese, F. Software Update: The ORCA Program System—Version 5.0. *Wiley Interdiscip. Rev. Comput. Mol. Sci.* **2022**, *12* (5), 1–15. https://doi.org/10.1002/wcms.1606.

(4) Chai, J.-D.; Head-Gordon, M. Long-Range Corrected Hybrid Density Functionals with Damped Atom–Atom Dispersion Corrections. *Phys. Chem. Chem. Phys.* **2008**, *10* (44), 6615–6620. https://doi.org/10.1039/B810189B.

(5) Pracht, P.; Bohle, F.; Grimme, S. Automated Exploration of the Low-Energy Chemical Space with Fast Quantum Chemical Methods. *Phys. Chem. Chem. Phys.* **2020**, *22* (14), 7169–7192. https://doi.org/10.1039/C9CP06869D.

(6) Cancès, E.; Mennucci, B.; Tomasi, J. A New Integral Equation Formalism for the Polarizable Continuum Model: Theoretical Background and Applications to Isotropic and Anisotropic Dielectrics. *J. Chem. Phys.* **1997**, *107* (8), 3032–3041. https://doi.org/10.1063/1.474659.

(7) MATLAB. 9.8.0.1323502 (R2020a). The MathWorks Inc.: Natick, Massachusetts 2020.

(8) Ribeiro, R. F.; Marenich, A. V.; Cramer, C. J.; Truhlar, D. G. Use of Solution-Phase Vibrational Frequencies in Continuum Models for the Free Energy of Solvation. *J. Phys. Chem. B* **2011**, *115* (49), 14556–14562. https://doi.org/10.1021/jp205508z.

(9) R. S. Mulliken. Electronic Population Analysis on LCAO. *J. Chem. Phys.* **1955**, *23* (January 1955), 1833.

(10) Marenich, A. V.; Cramer, C. J.; Truhlar, D. G. Universal Solvation Model Based on Solute Electron Density and on a Continuum Model of the Solvent Defined by the Bulk Dielectric Constant and Atomic Surface Tensions. *J. Phys. Chem. B* **2009**, *113* (18), 6378–6396. https://doi.org/10.1021/jp810292n.

(11) Zhao, Y.; Truhlar, D. G. The M06 Suite of Density Functionals for Main Group Thermochemistry, Thermochemical Kinetics, Noncovalent Interactions, Excited States, and Transition Elements: Two New Functionals and Systematic Testing of Four M06-Class Functionals and 12 Other Function. *Theor. Chem. Acc.* **2008**, *120* (1–3), 215–241. https://doi.org/10.1007/s00214-007-0310-x.

(12) Lee, C.; Yang, W.; Parr, R. G. Development of the Colle-Salvetti Correlation-Energy Formula into a Functional of the Electron Density. *Phys. Rev. B* **1988**, *37* (2), 785–789. https://doi.org/10.1103/PhysRevB.37.785.

(13) Beck, A. D. Density-Functional Thermochemistry. III. The Role of Exact Exchange. *J. Chem. Phys.* **1993**, *98* (7), 5648–5656.

(14) Liakos, D. G.; Guo, Y.; Neese, F. Comprehensive Benchmark Results for the Domain Based Local Pair Natural Orbital Coupled Cluster Method (DLPNO-CCSD(T)) for Closed-And Open-Shell Systems. *J. Phys. Chem. A* **2020**, *124* (1), 90–100. https://doi.org/10.1021/acs.jpca.9b05734.

(15) Legault, C. Y. CYLview. Universite de Sherbrook 2020, http://www.cylview.org.

^{1}H and ^{13}C NMR Spectra for New Compounds

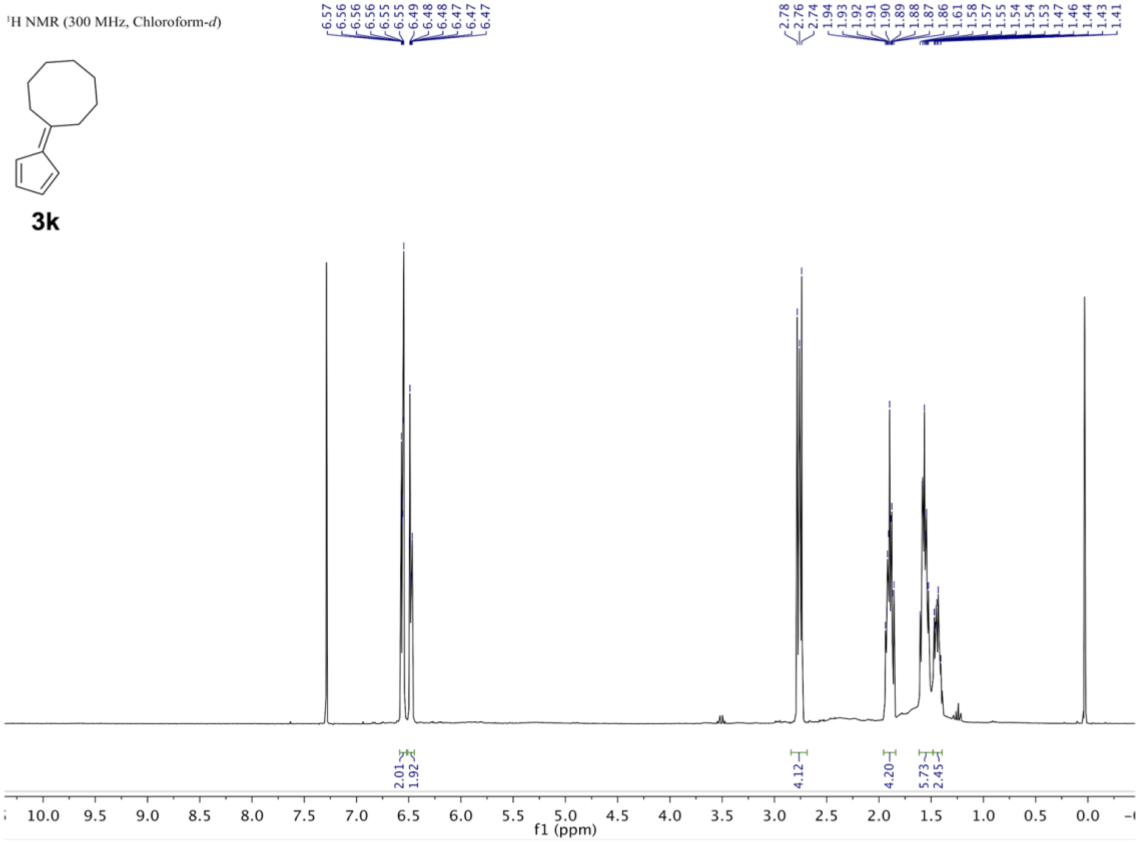

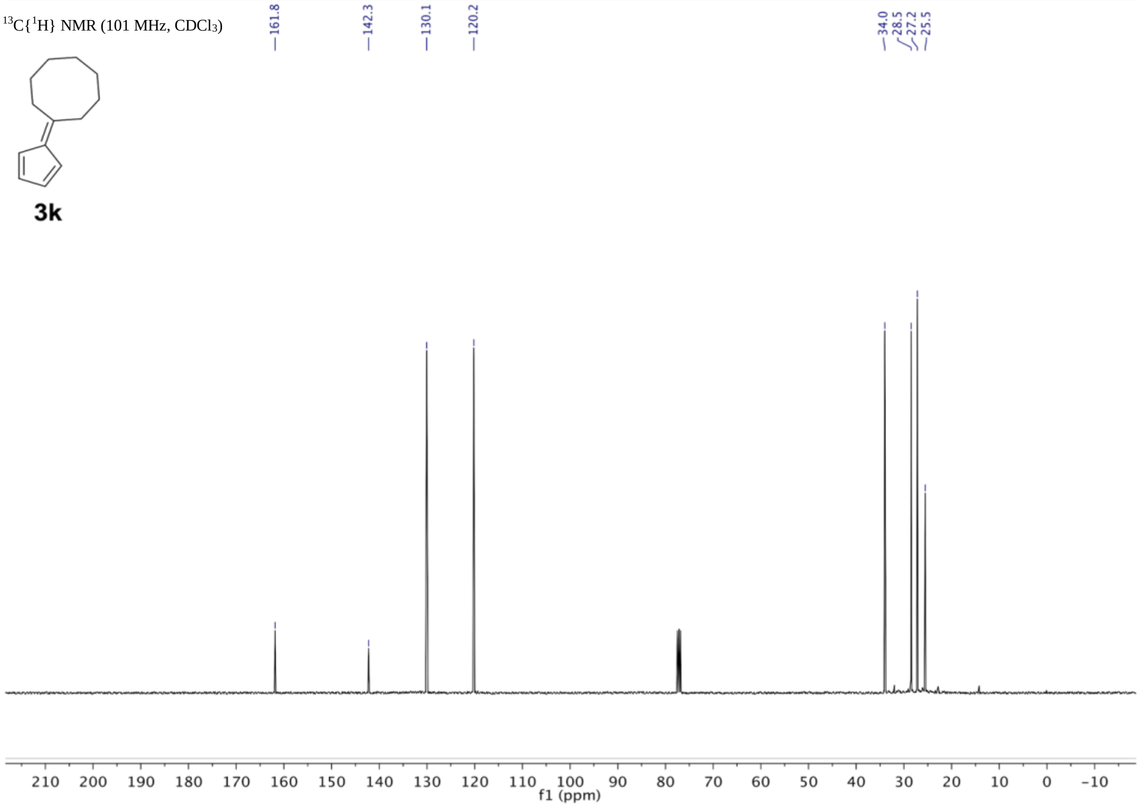

^{1}H NMR (400 MHz, Chloroform-*d*)

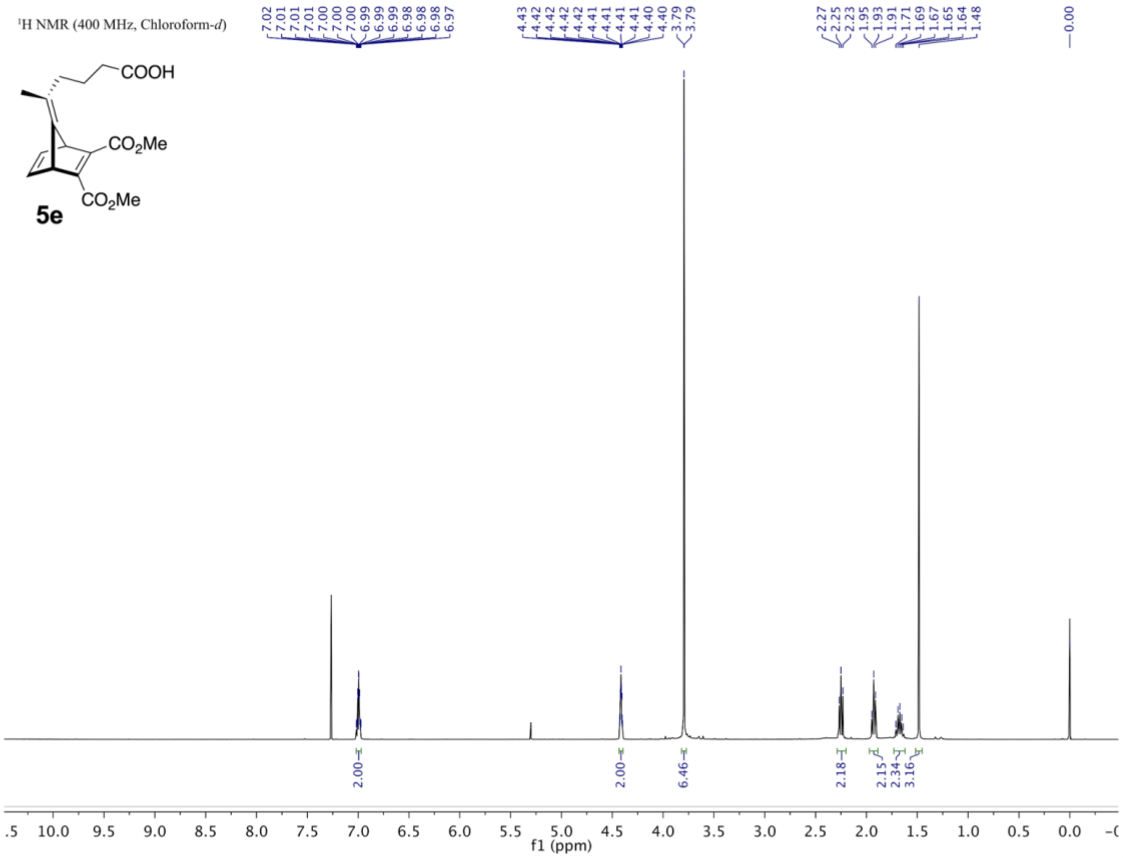

$^{13}C\{^{1}H\}$ NMR (101 MHz, $CDCl_3$)

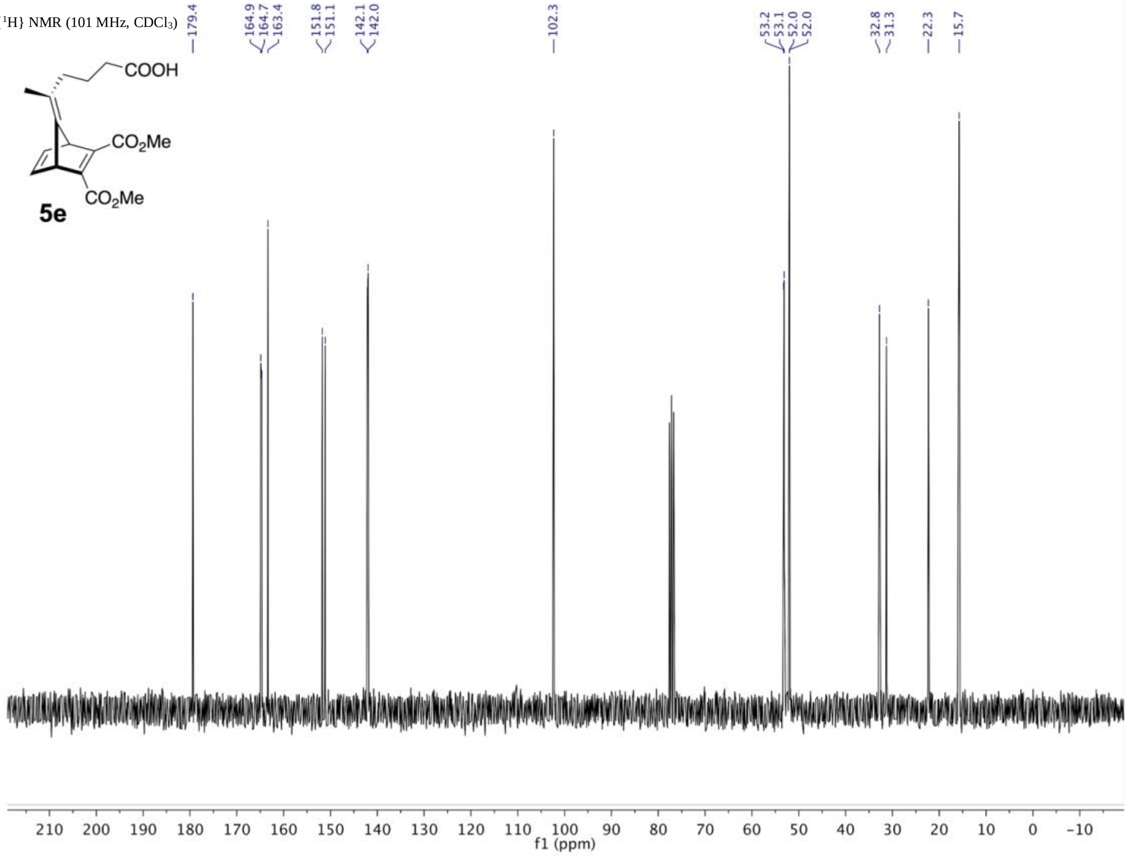

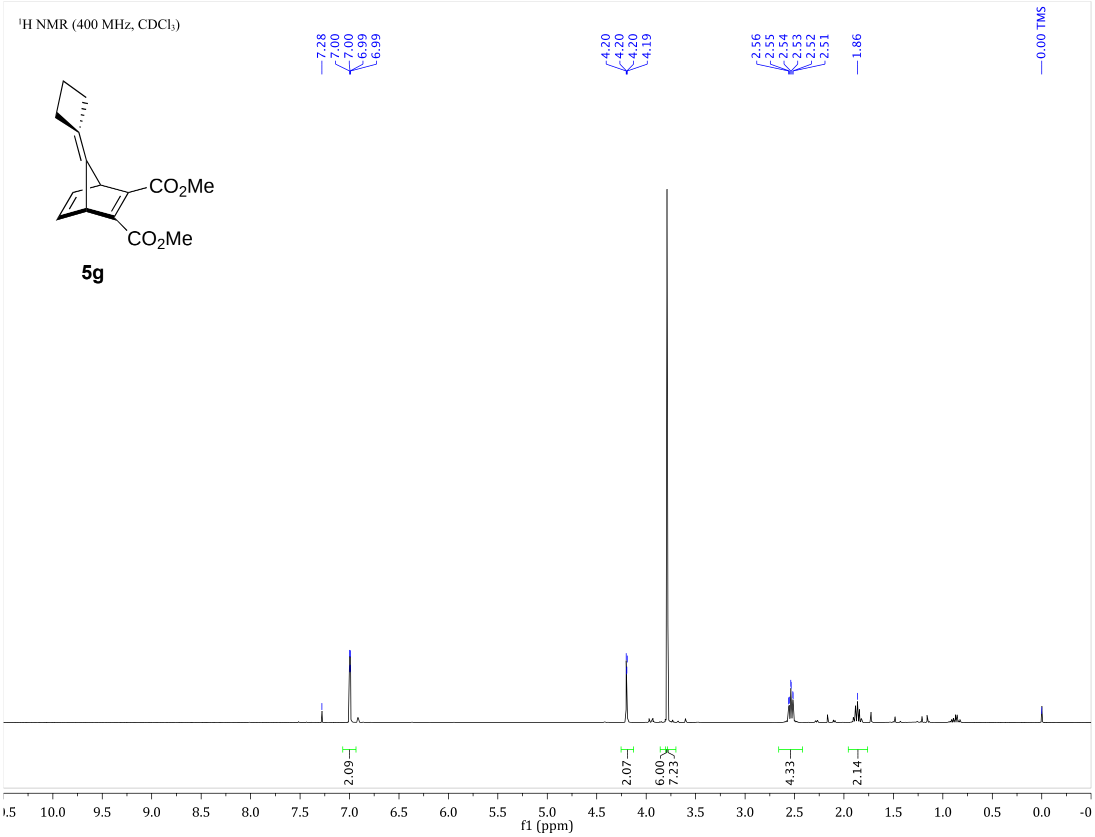

1H NMR (400 MHz, CDCl3)
7.28
7.00
7.00
6.99
6.99
4.20
4.20
4.20
4.19
2.56
2.55
2.54
2.53
2.52
2.51
1.86
0.00 TMS
CO2Me
CO2Me
5g
2.09
2.07
6.00
7.23
4.33
2.14
f1 (ppm)

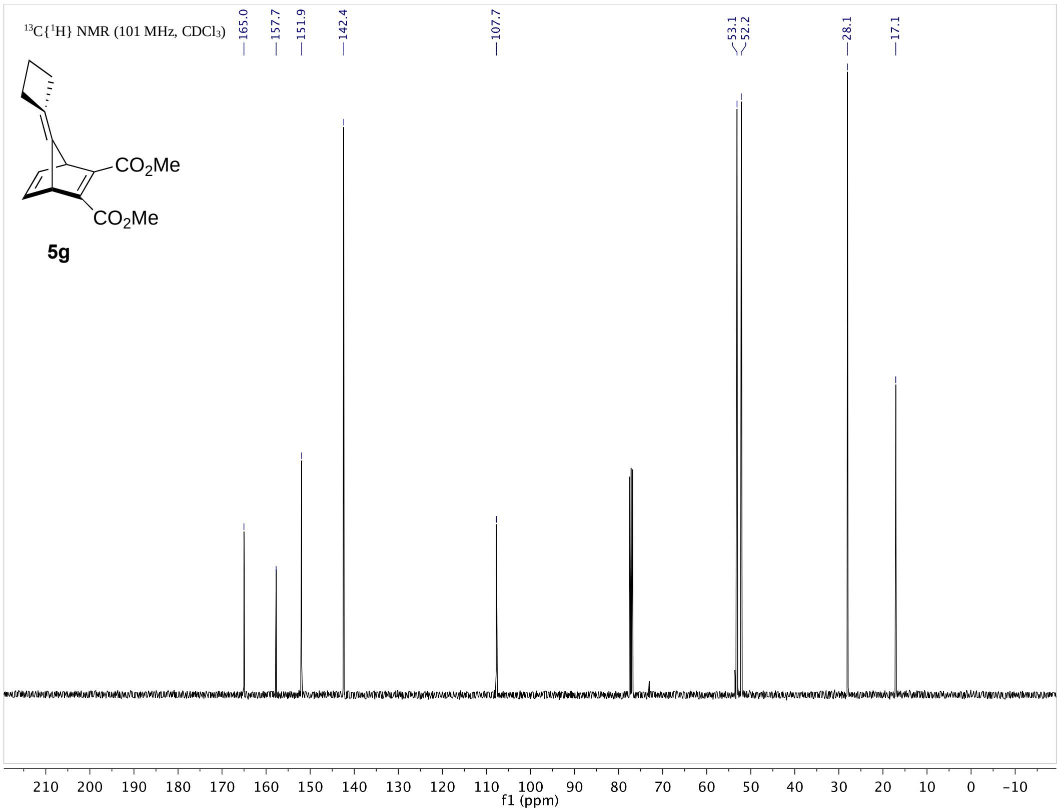

13C{1H} NMR (101 MHz, CDCl3)
165.0
157.7
151.9
142.4
107.7
53.1
52.2
28.1
17.1
CO2Me
CO2Me
5g
f1 (ppm)

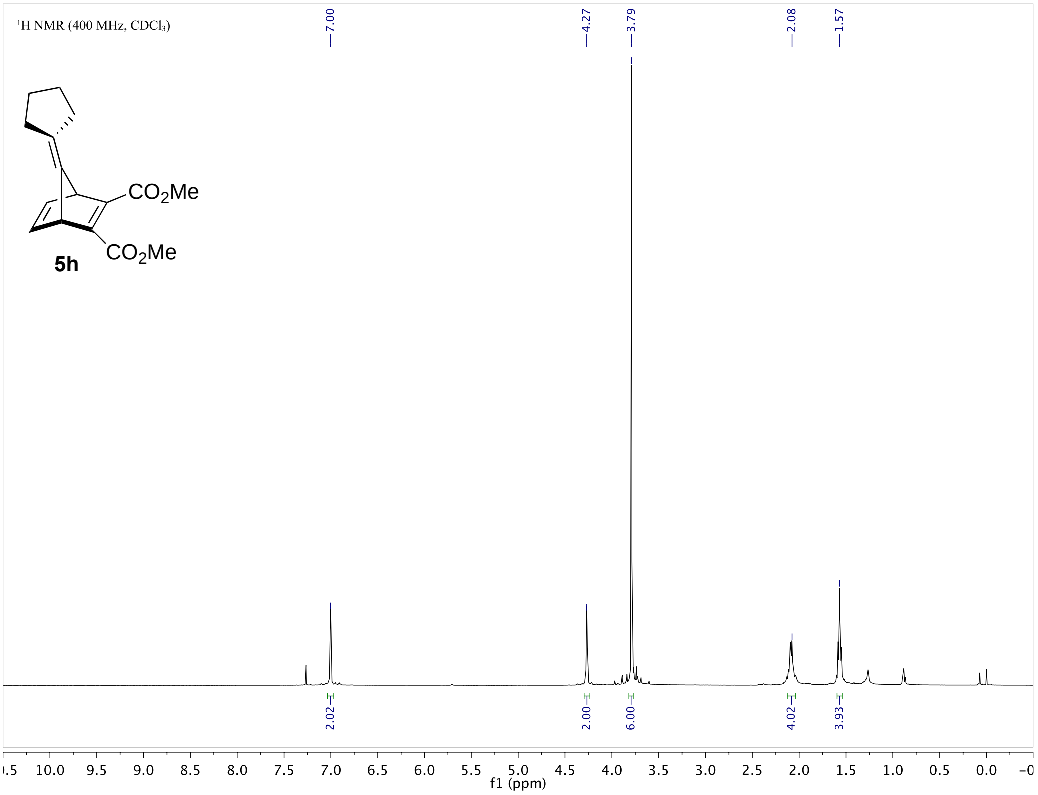
1H NMR (400 MHz, CDCl3)
7.00
4.27
3.79
2.08
1.57
CO2Me
CO2Me
5h
2.02
2.00
6.00
4.02
3.93
f1 (ppm)

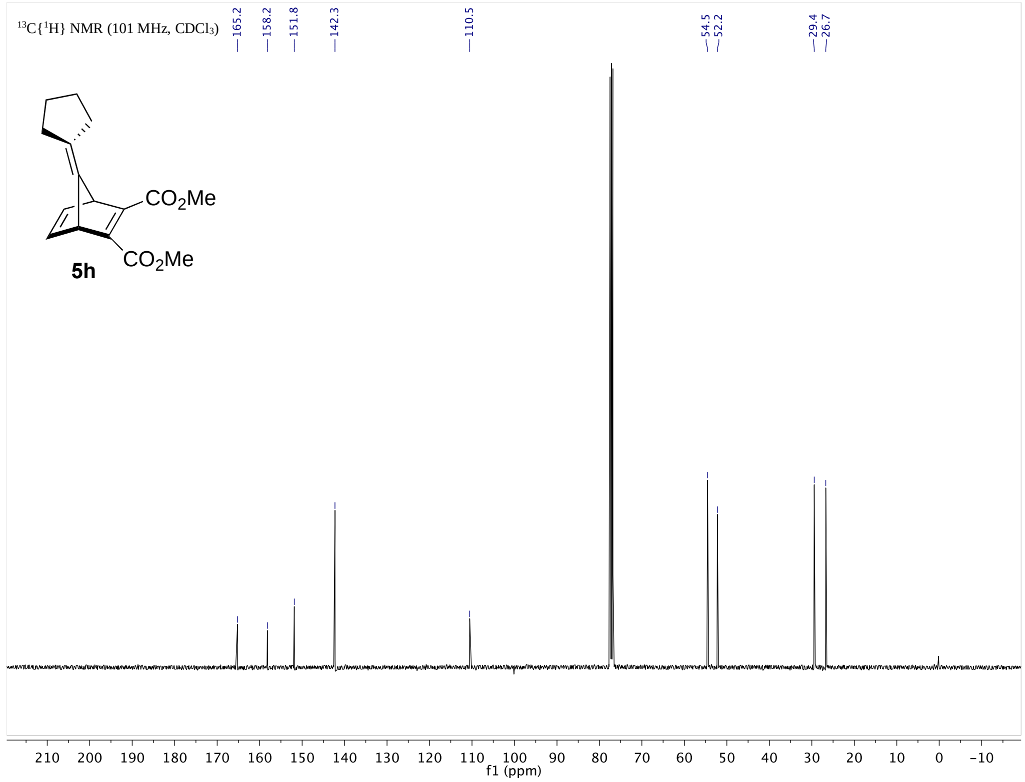
13C{1H} NMR (101 MHz, CDCl3)
165.2
158.2
151.8
142.3
110.5
54.5
52.2
29.4
26.7
CO2Me
CO2Me
5h
f1 (ppm)

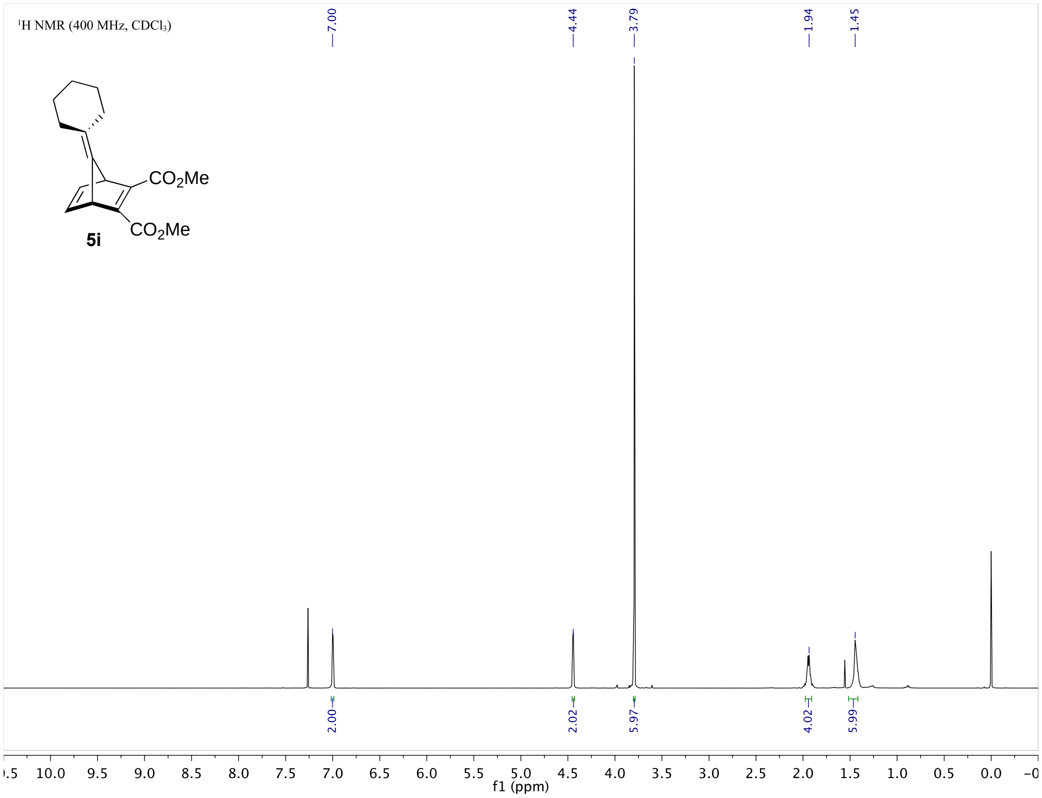

1H NMR (400 MHz, CDCl3)
7.00
4.44
3.79
1.94
1.45
CO2Me
CO2Me
5i
2.00
2.02
5.97
4.02
5.99
f1 (ppm)

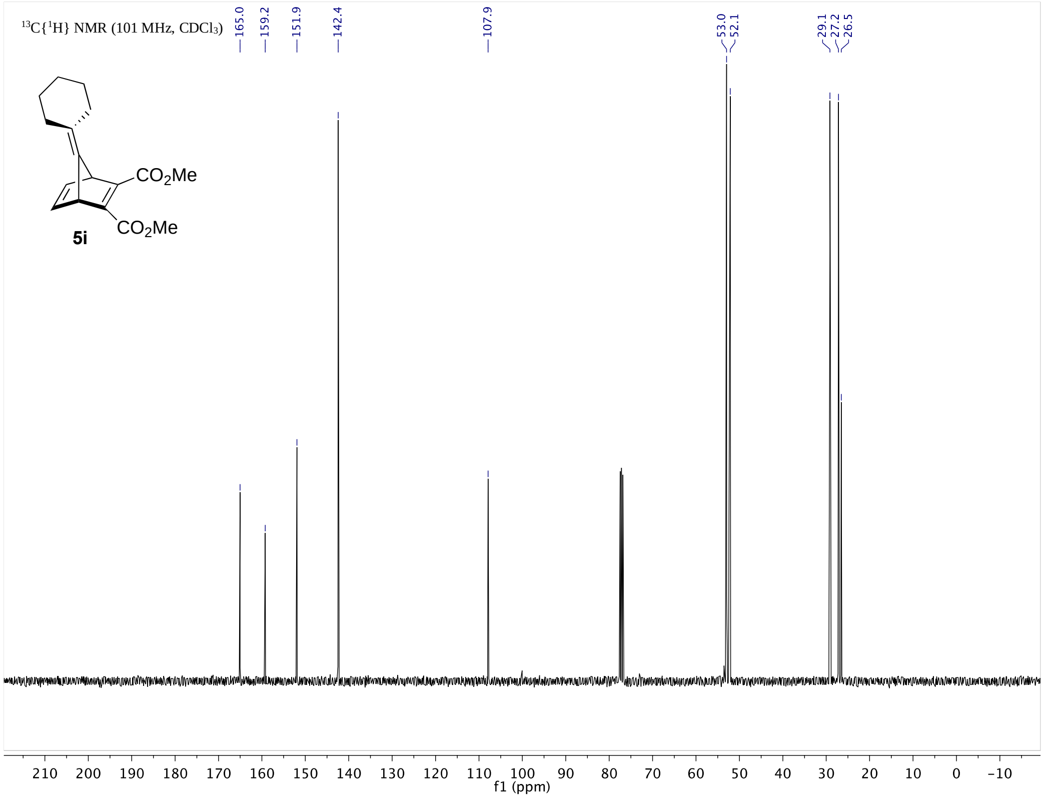

13C{1H} NMR (101 MHz, CDCl3)
165.0
159.2
151.9
142.4
107.9
53.0
52.1
29.1
27.2
26.5
CO2Me
CO2Me
5i
f1 (ppm)

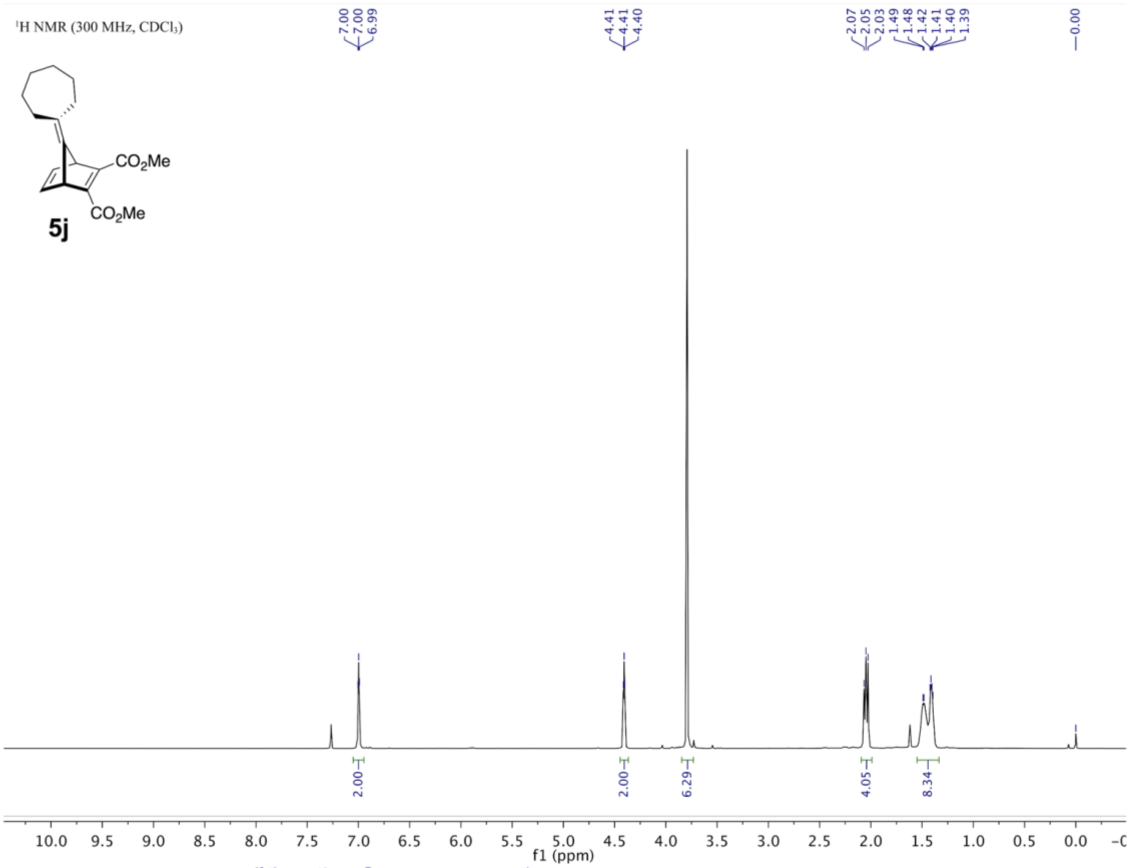

^1H NMR (300 MHz, $CDCl_3$)
7.00
7.00
6.99
4.41
4.41
4.40
2.07
2.05
2.03
1.49
1.48
1.42
1.41
1.40
1.39
0.00
CO_2Me
CO_2Me
5j
2.00
2.00
6.29
4.05
8.34
10.0 9.5 9.0 8.5 8.0 7.5 7.0 6.5 6.0 5.5 5.0 4.5 4.0 3.5 3.0 2.5 2.0 1.5 1.0 0.5 0.0
f1 (ppm)

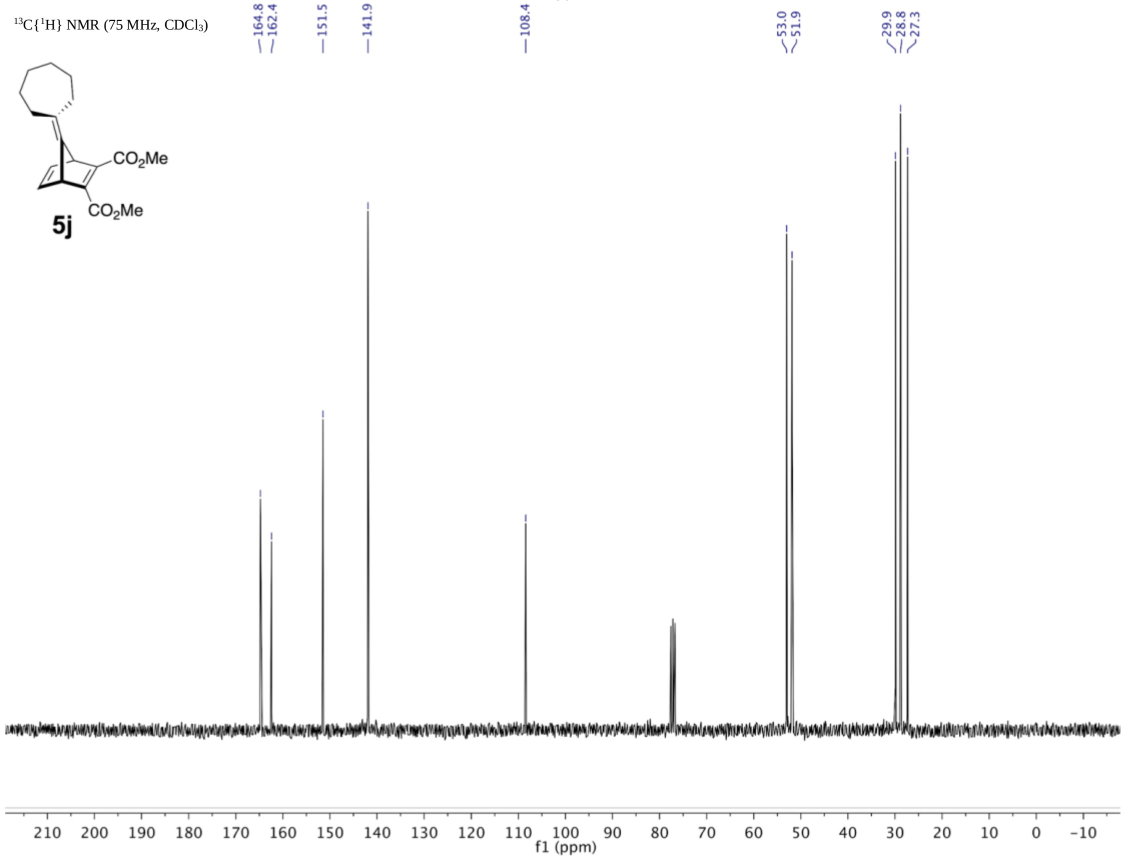

$^{13}C\{^1H\}$ NMR (75 MHz, $CDCl_3$)
164.8
162.4
151.5
141.9
108.4
53.0
51.9
29.9
28.8
27.3
CO_2Me
CO_2Me
5j
210 200 190 180 170 160 150 140 130 120 110 100 90 80 70 60 50 40 30 20 10 0 −10
f1 (ppm)

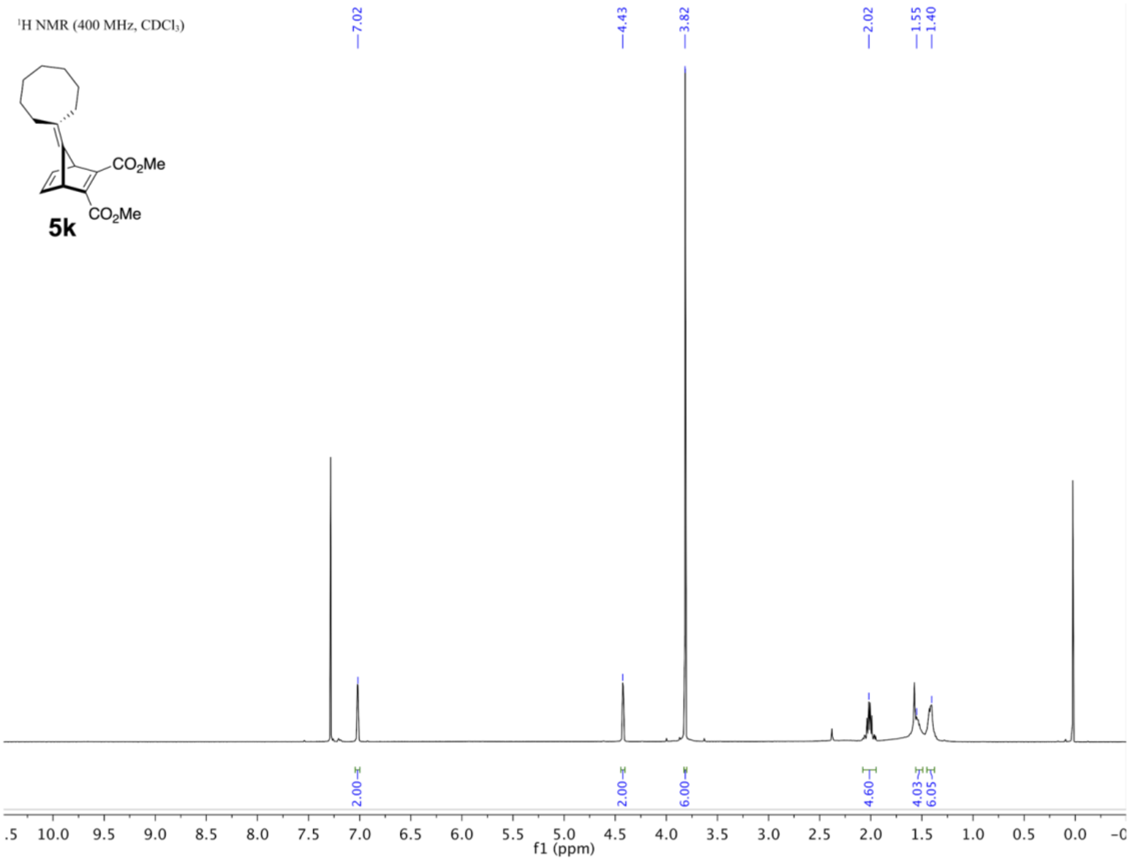

^{1}H NMR (400 MHz, CDCl$_3$)
7.02
4.43
3.82
2.02
1.55
1.40
CO_2Me
CO_2Me
5k
2.00
2.00
6.00
4.60
4.03
6.05
f1 (ppm)

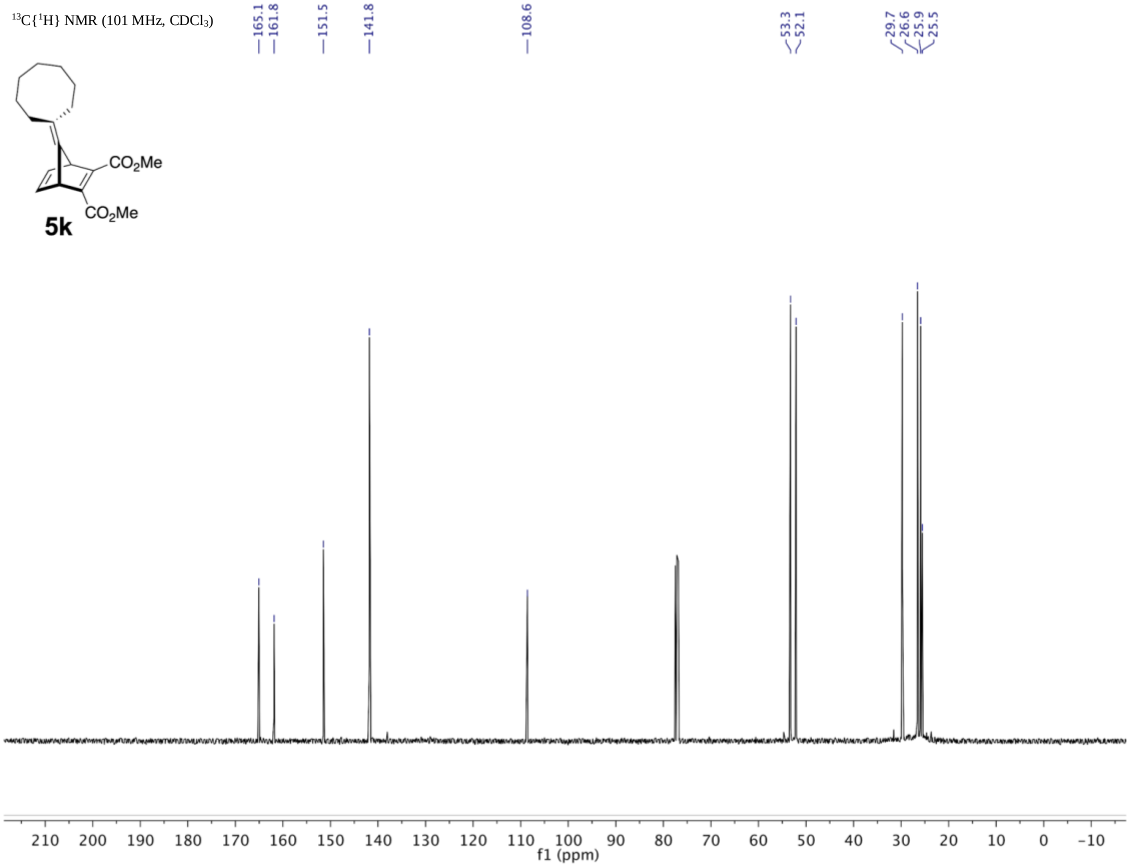

^{13}C{^{1}H} NMR (101 MHz, CDCl$_3$)
165.1
161.8
151.5
141.8
108.6
53.3
52.1
29.7
26.6
25.9
25.5
CO_2Me
CO_2Me
5k
f1 (ppm)

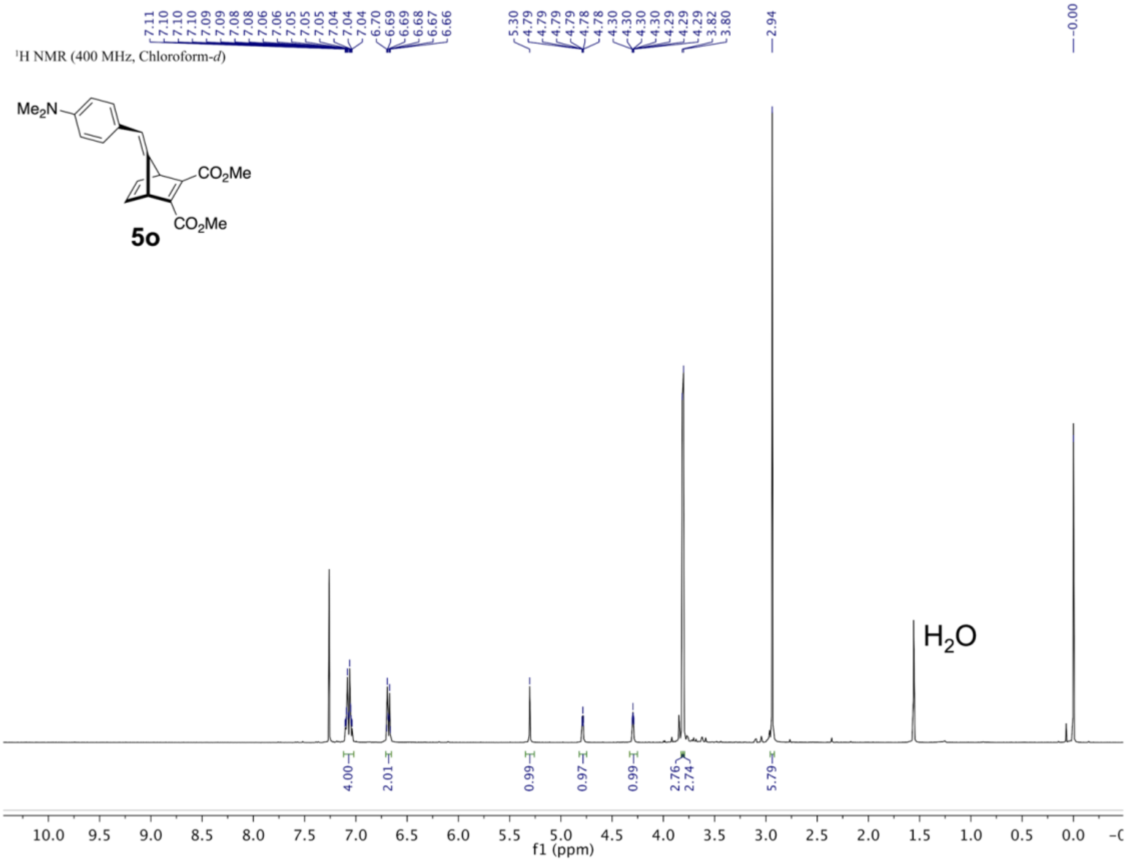

¹H NMR (400 MHz, Chloroform-*d*)
Me2N
CO2Me
CO2Me
5o
7.11 7.10 7.10 7.09 7.09 7.08 7.08 7.06 7.06 7.05 7.05 7.05 7.04 7.04 6.70 6.69 6.69 6.68 6.67 6.66
5.30 4.79 4.79 4.79 4.78 4.78 4.30 4.30 4.30 4.29 4.29 4.29 3.82 3.80
2.94
-0.00
H2O
4.00 2.01 0.99 0.97 0.99 2.76 2.74 5.79
10.0 9.5 9.0 8.5 8.0 7.5 7.0 6.5 6.0 5.5 5.0 4.5 4.0 3.5 3.0 2.5 2.0 1.5 1.0 0.5 0.0
f1 (ppm)

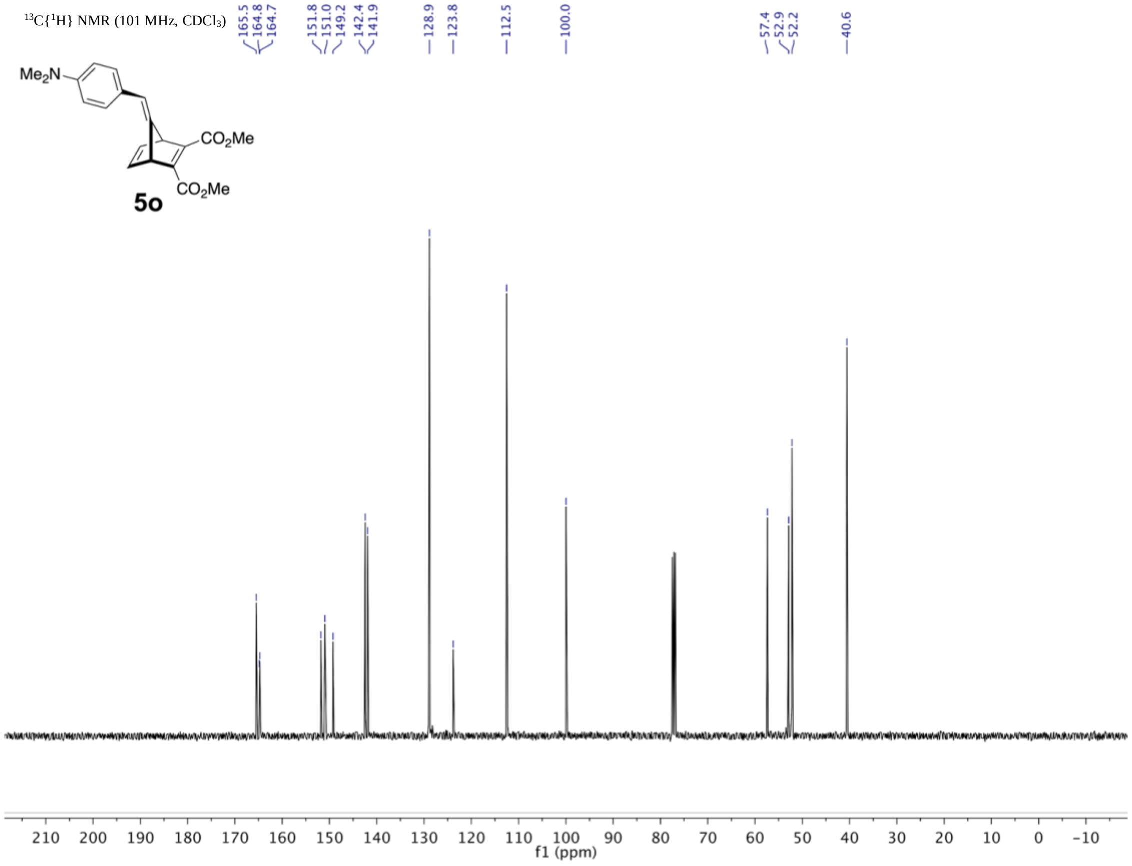

¹³C{¹H} NMR (101 MHz, CDCl₃)
Me2N
CO2Me
CO2Me
5o
165.5 164.8 164.7 151.8 151.0 149.2 142.4 141.9 128.9 123.8 112.5 100.0 57.4 52.9 52.2 40.6
210 200 190 180 170 160 150 140 130 120 110 100 90 80 70 60 50 40 30 20 10 0 -10
f1 (ppm)

^{1}H NMR (400 MHz, Chloroform-*d*)

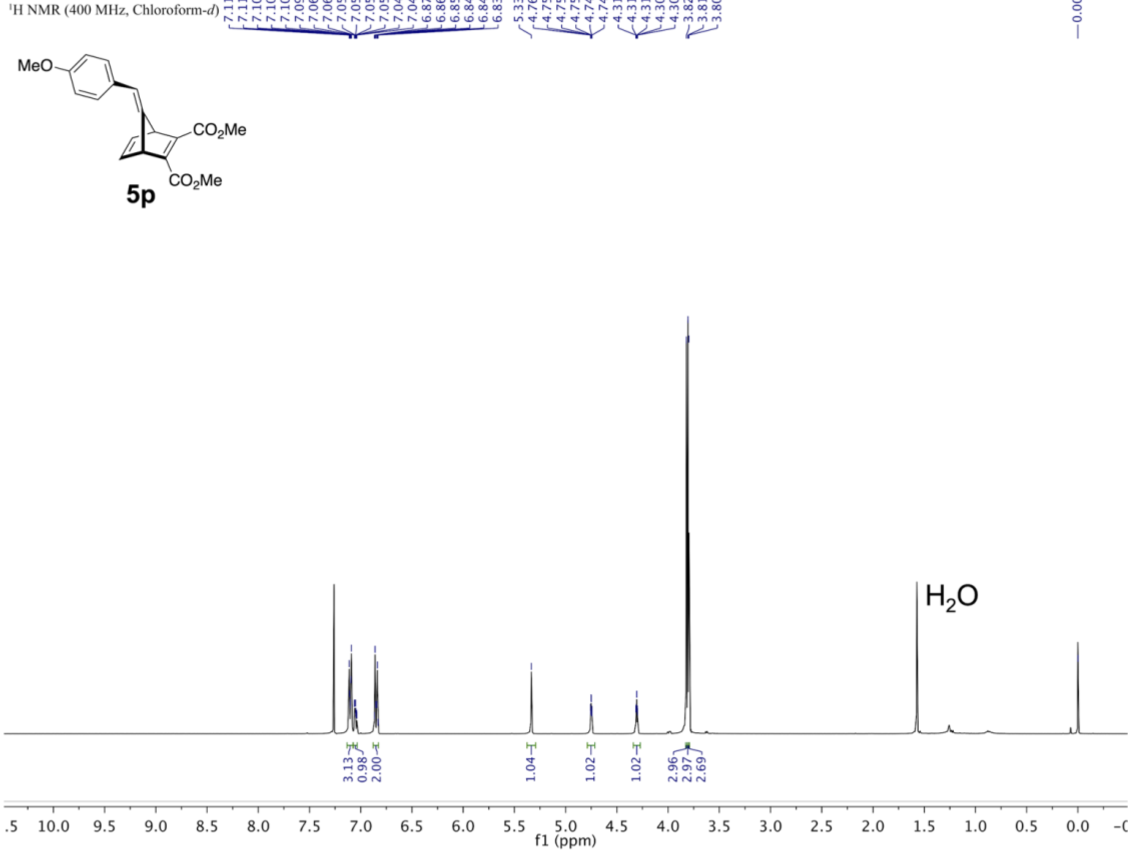

$^{13}C\{^{1}H\}$ NMR (101 MHz, $CDCl_3$)

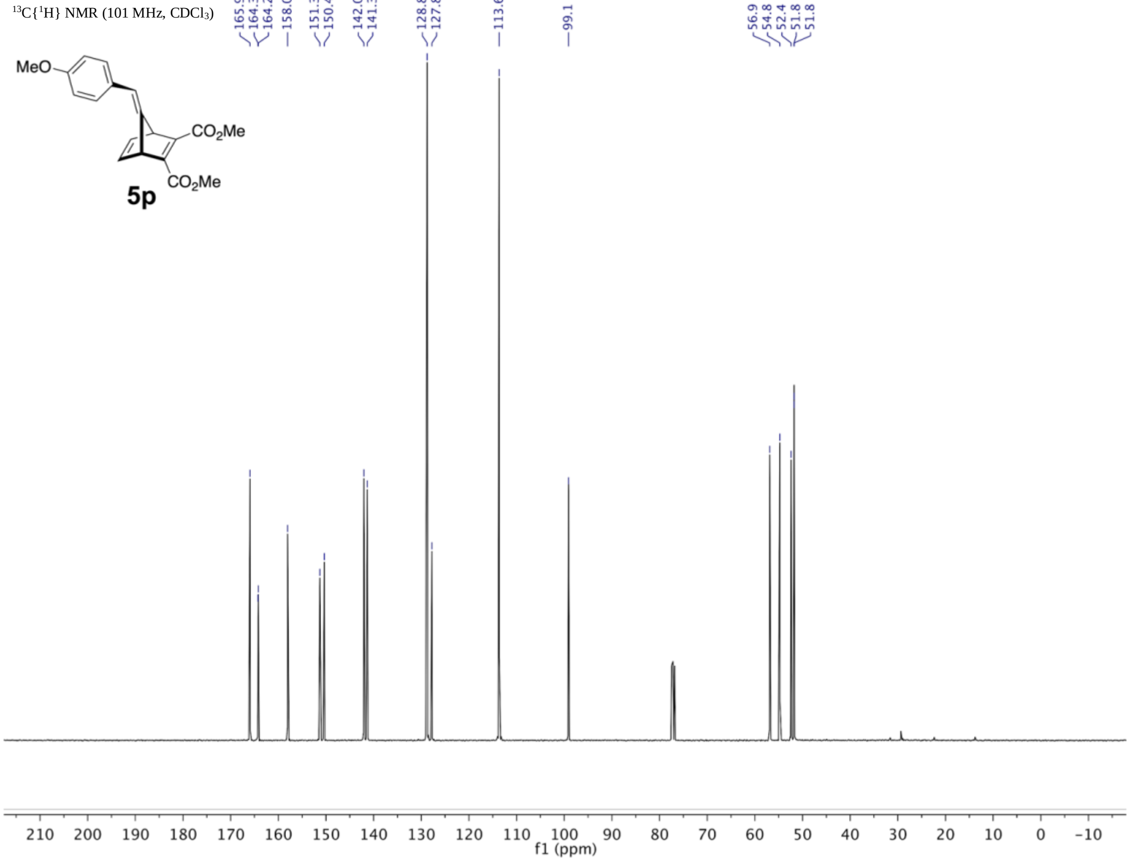

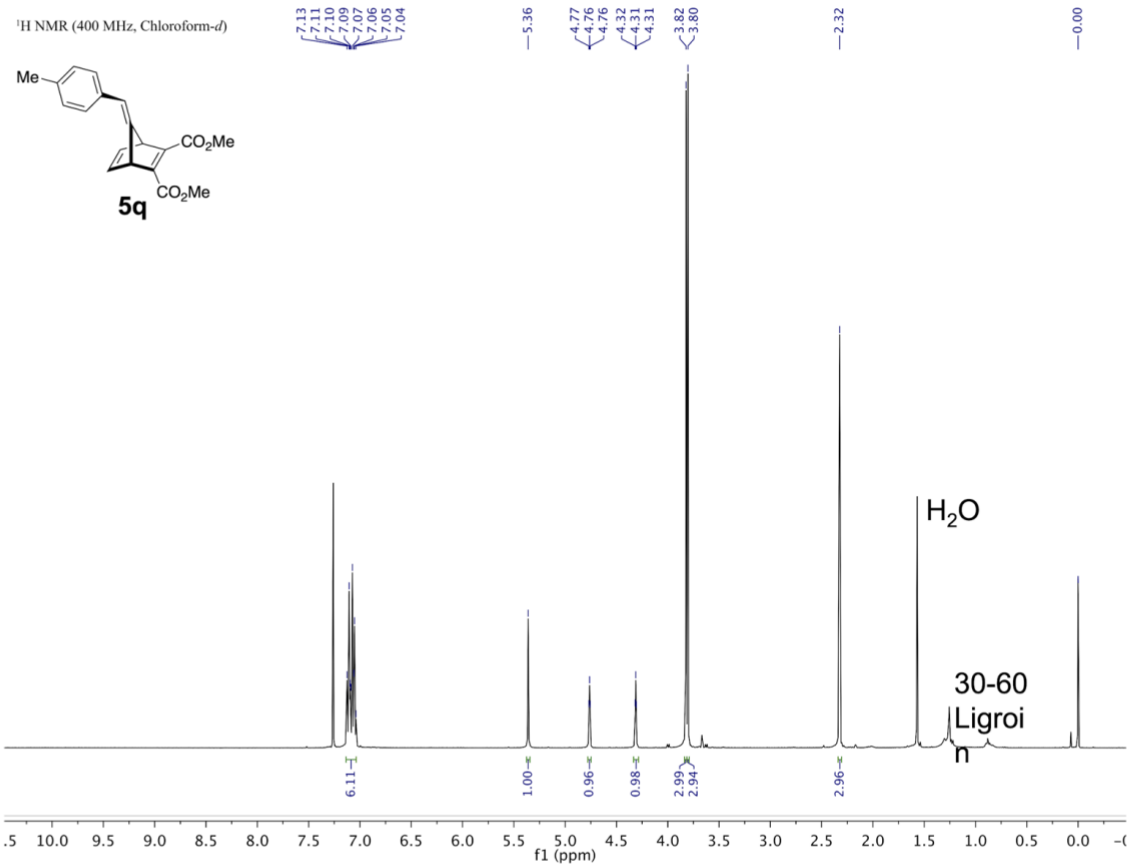

1H NMR (400 MHz, Chloroform-d)
Me
CO2Me
CO2Me
5q
7.13
7.11
7.10
7.09
7.07
7.06
7.05
7.04
5.36
4.77
4.76
4.76
4.32
4.31
4.31
3.82
3.80
2.32
0.00
H2O
30-60
Ligroi
n
6.11
1.00
0.96
0.98
2.99
2.94
2.96
f1 (ppm)

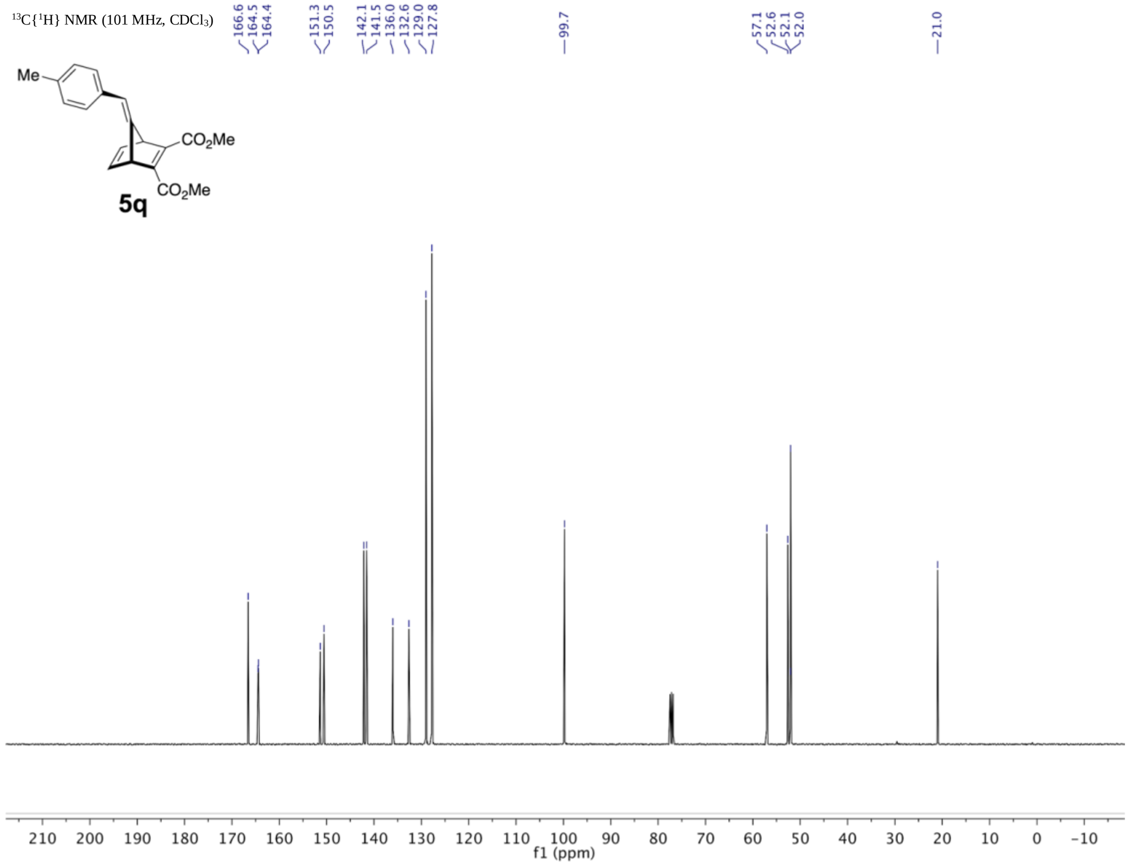

13C{1H} NMR (101 MHz, CDCl3)
Me
CO2Me
CO2Me
5q
166.6
164.5
164.4
151.3
150.5
142.1
141.5
136.0
132.6
129.0
127.8
99.7
57.1
52.6
52.1
52.0
21.0
f1 (ppm)

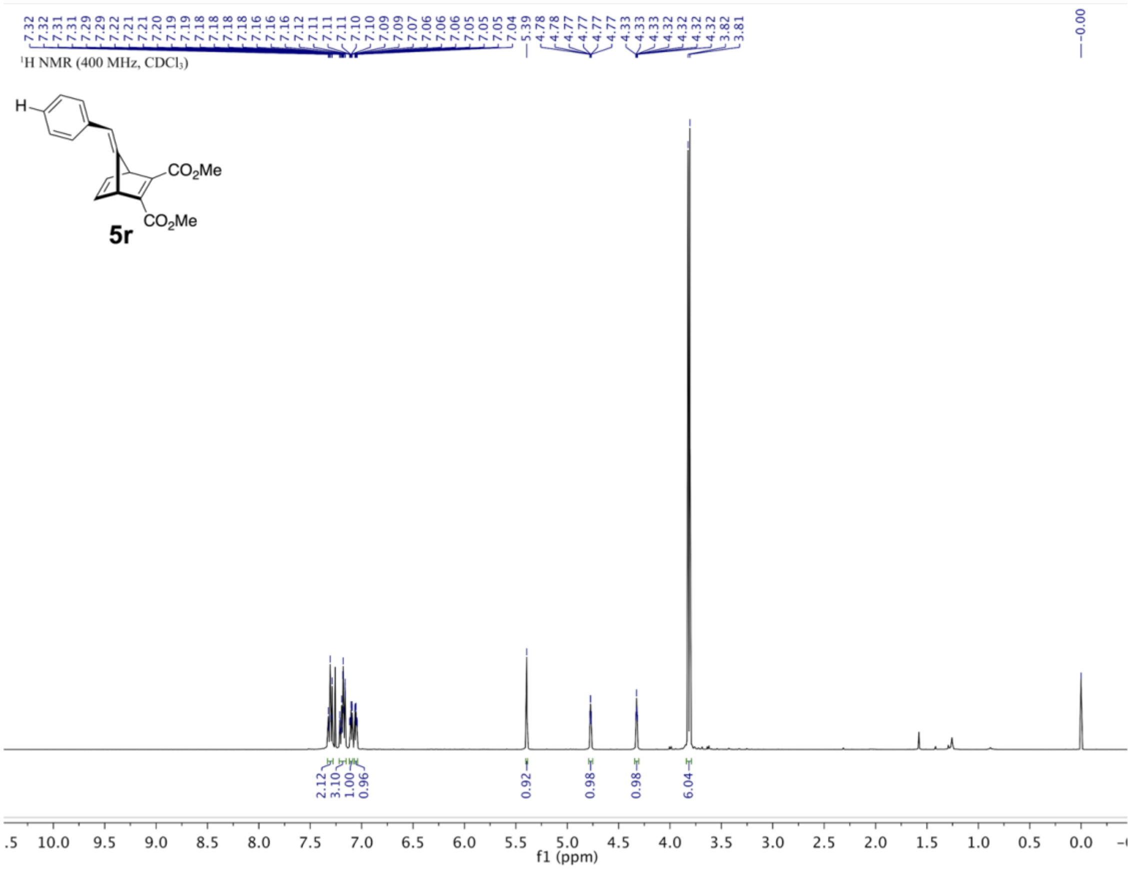
7.32 7.32 7.31 7.31 7.29 7.22 7.21 7.21 7.20 7.19 7.19 7.18 7.18 7.18 7.18 7.16 7.16 7.16 7.12 7.11 7.11 7.10 7.10 7.09 7.09 7.07 7.06 7.06 7.06 7.05 7.05 7.05 7.04 5.39 4.78 4.78 4.77 4.77 4.77 4.77 4.33 4.33 4.33 4.32 4.32 4.32 4.32 3.82 3.81
0.00
^{1}H NMR (400 MHz, $CDCl_3$)
H
CO_2Me
CO_2Me
5r
2.12 3.10 1.00 0.96 0.92 0.98 0.98 6.04
f1 (ppm)

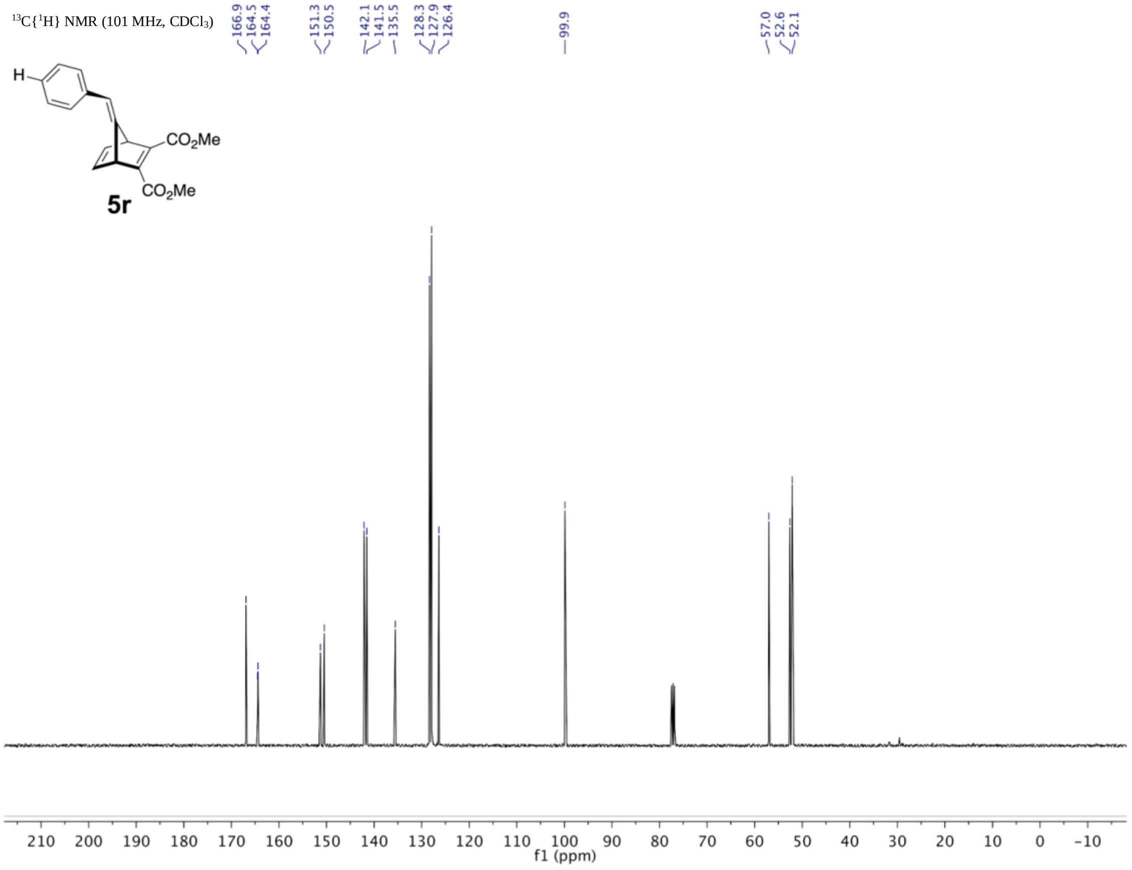
$^{13}C\{^1H\}$ NMR (101 MHz, $CDCl_3$)
166.9 164.5 164.4 151.3 150.5 142.1 141.5 135.5 128.3 127.9 126.4 99.9 57.0 52.6 52.1
H
CO_2Me
CO_2Me
5r
f1 (ppm)

^{1}H NMR (300 MHz, Chloroform-*d*)

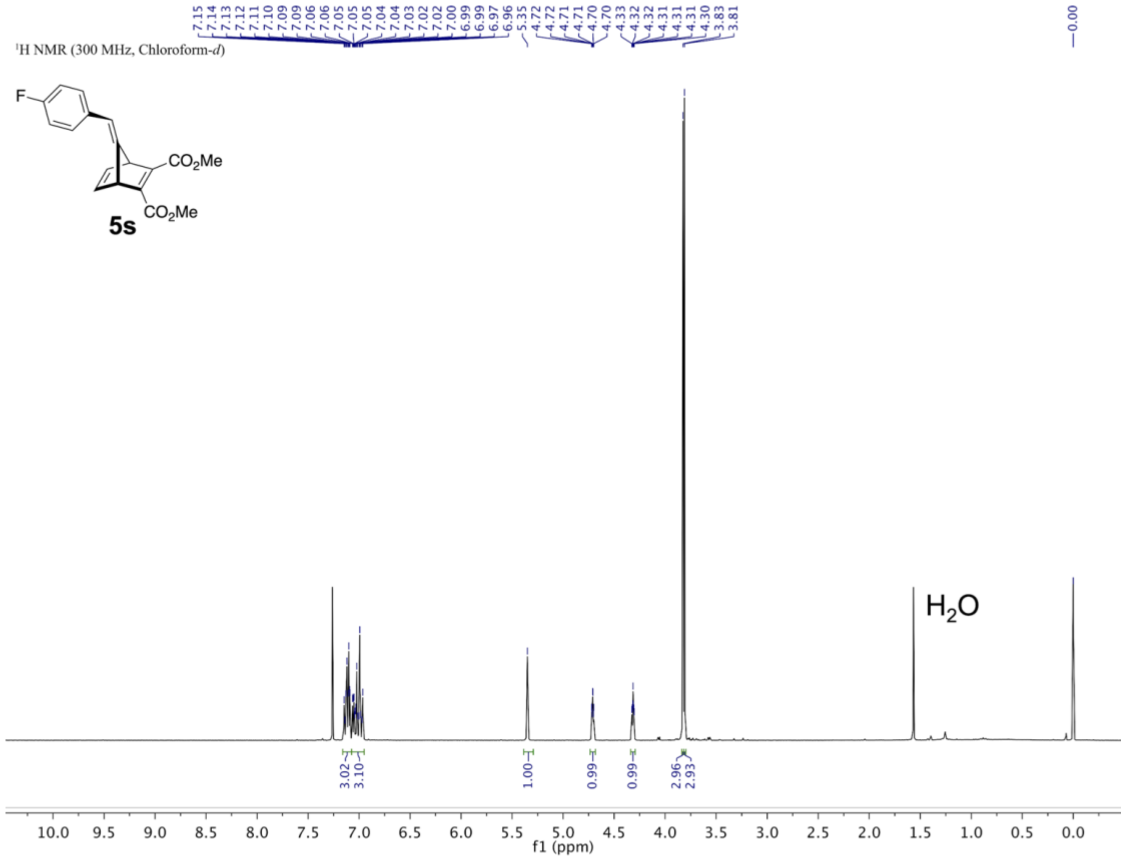

$^{13}C\{^{1}H\}$ NMR (75 MHz, $CDCl_3$)

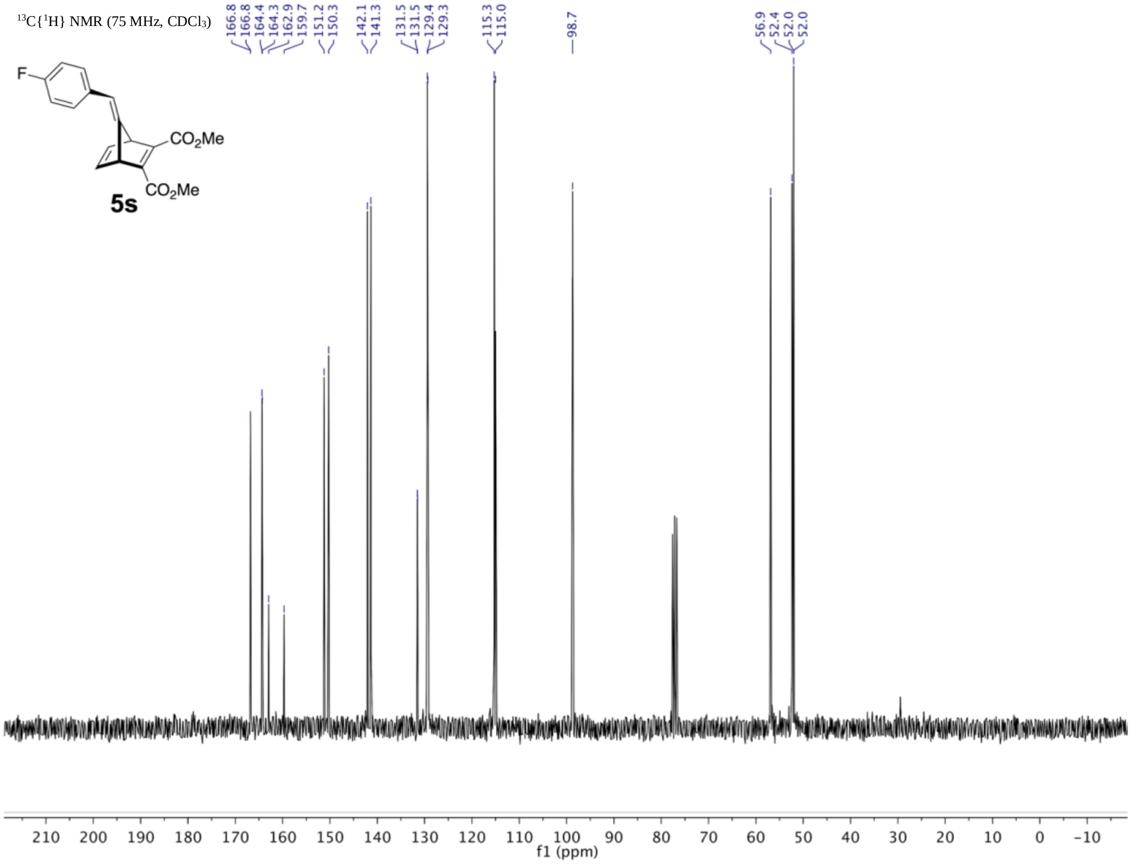

^{1}H NMR (400 MHz, $CDCl_3$)

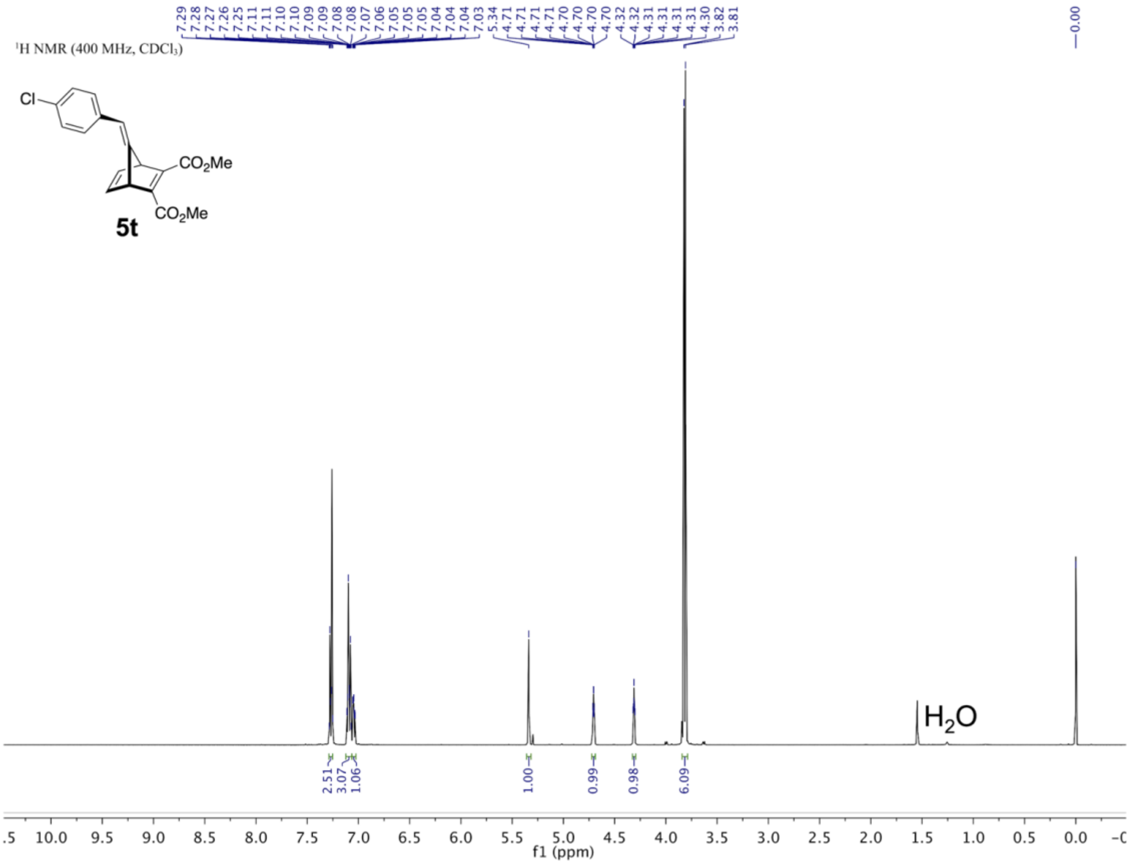

$^{13}C\{^{1}H\}$ NMR (101 MHz, $CDCl_3$)

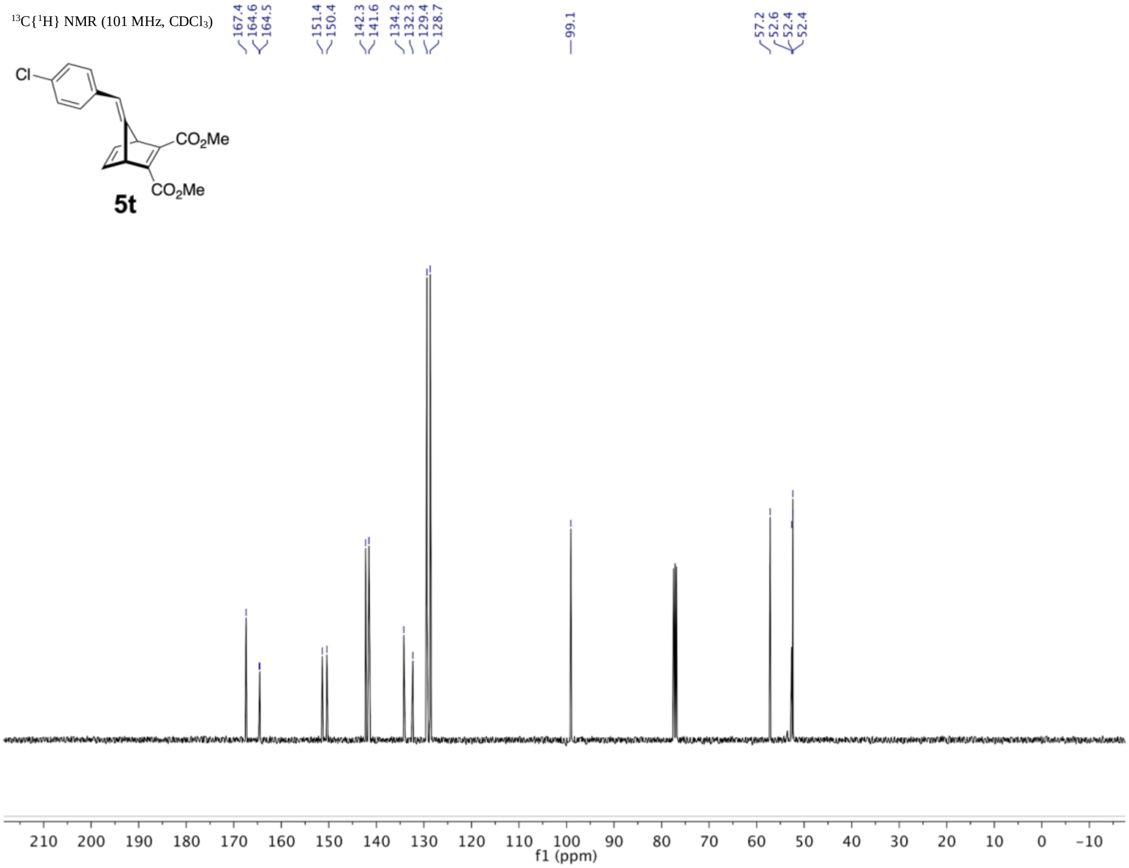

^{1}H NMR (300 MHz, Chloroform-*d*)

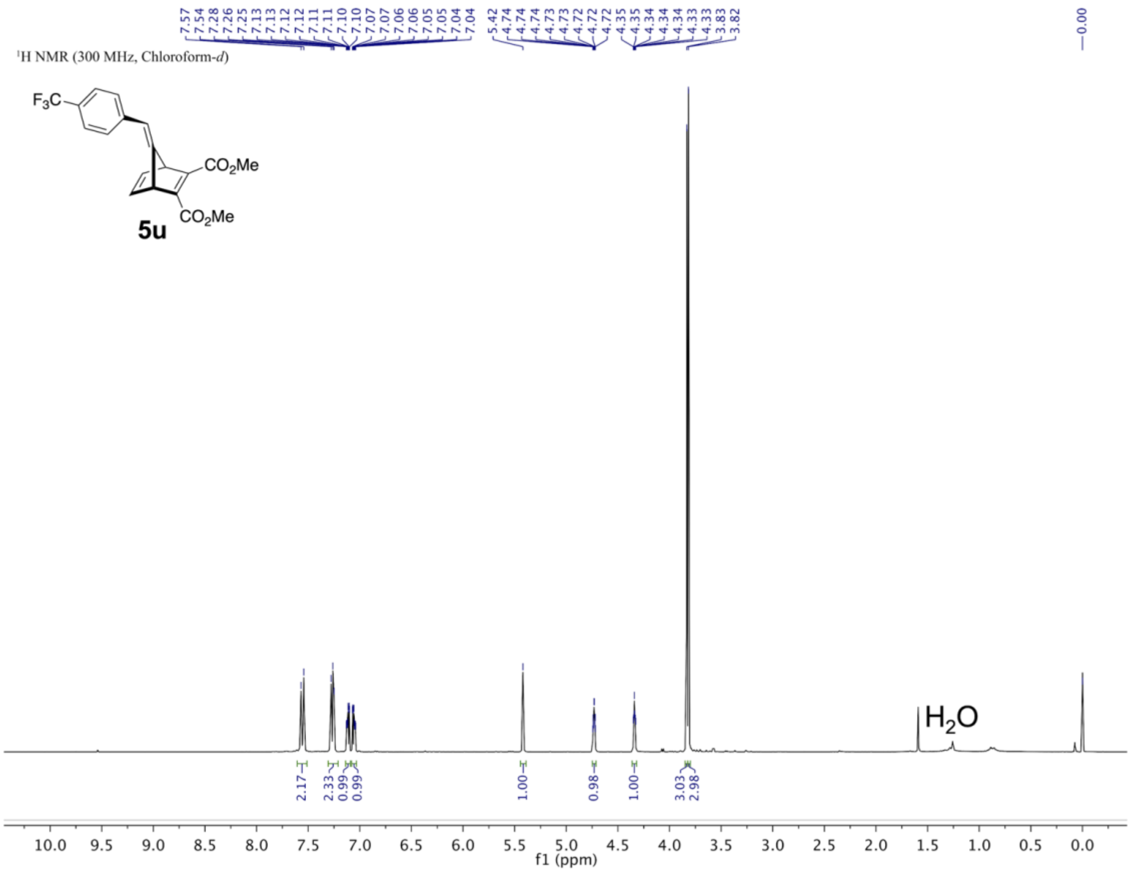

$^{13}C\{^{1}H\}$ NMR (75 MHz, $CDCl_3$)

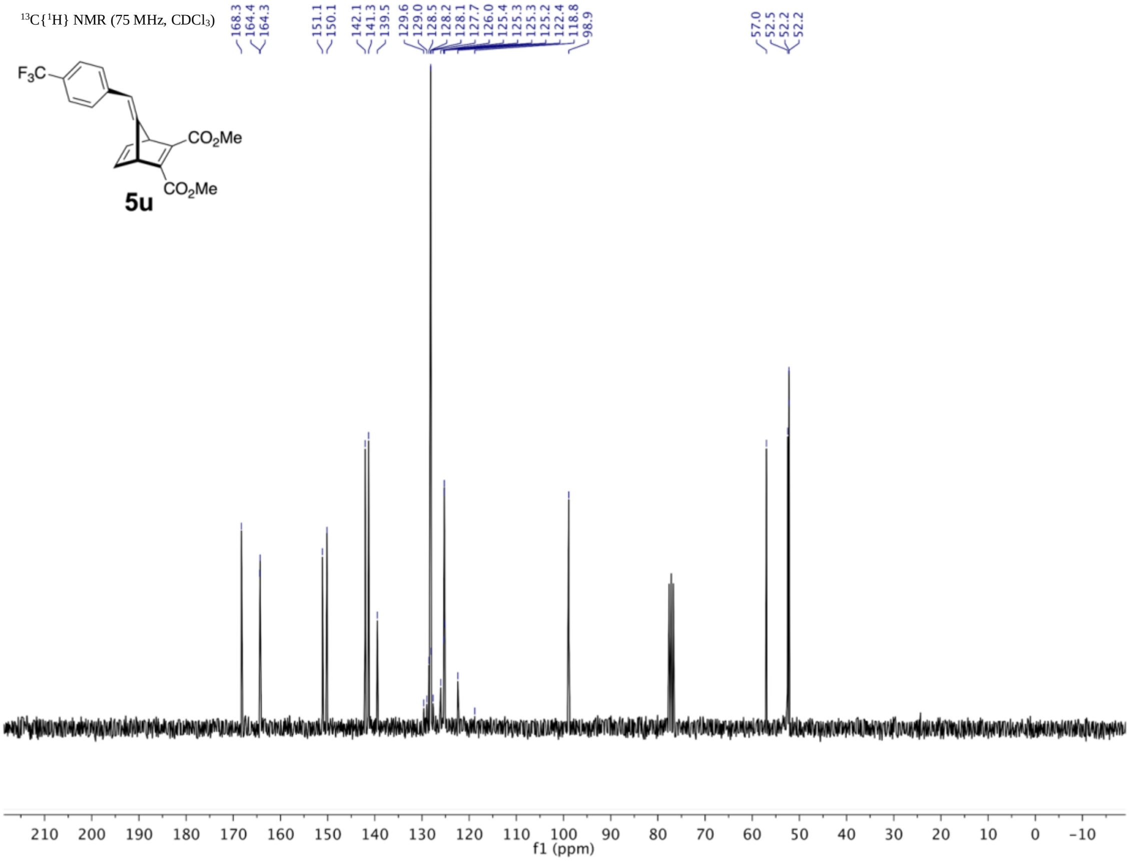

^1H NMR (400 MHz, $CDCl_3$)

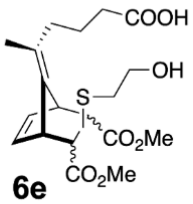

6e

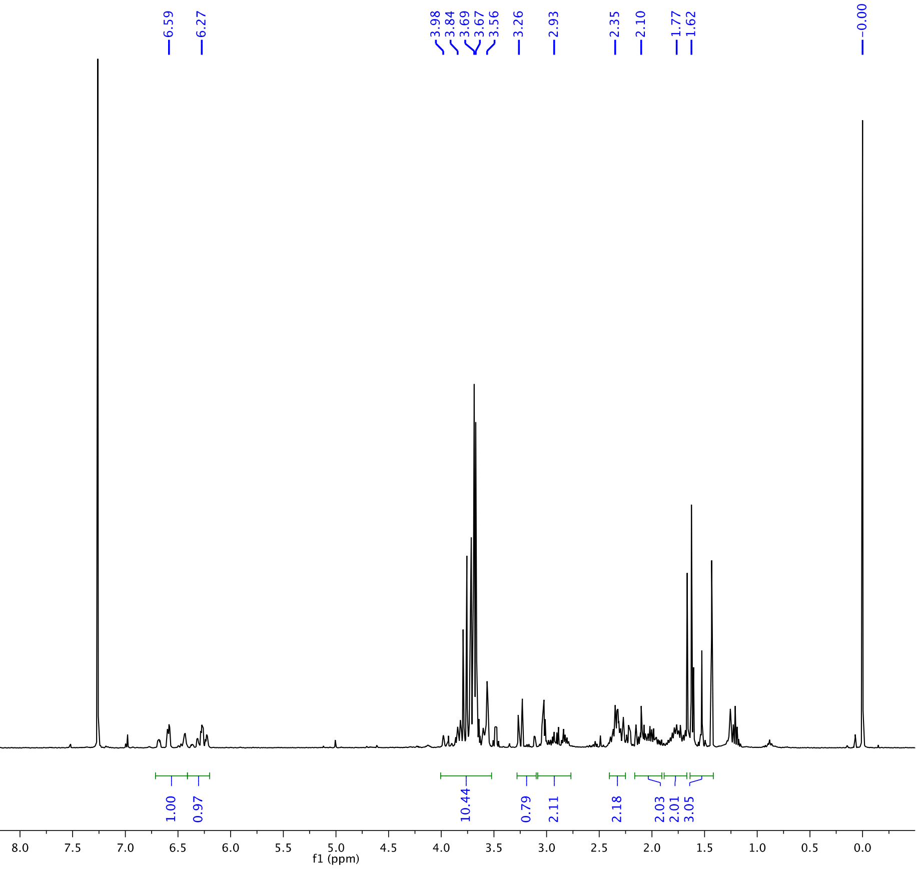

^{1}H NMR (400 MHz, $CDCl_3$)

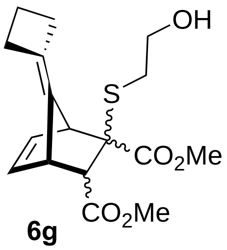

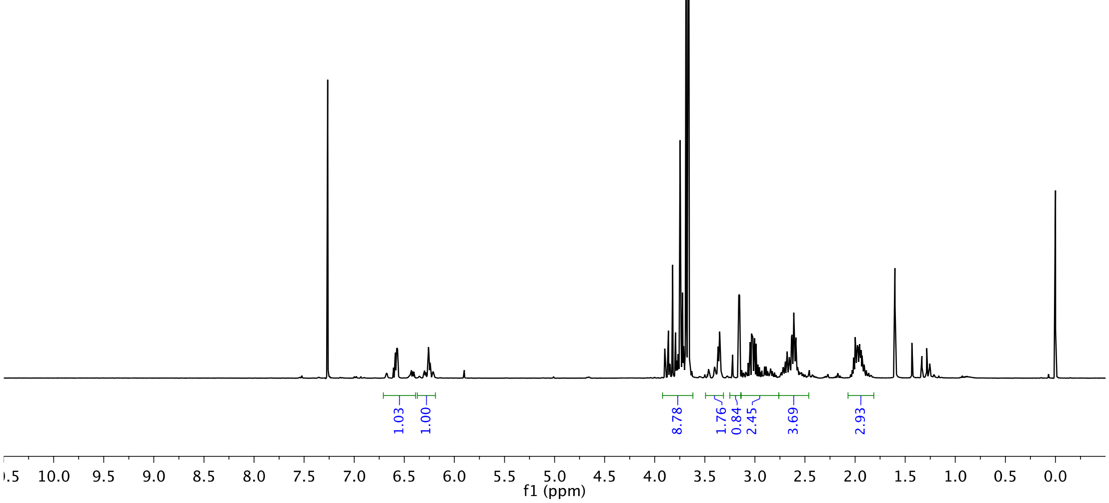

^{1}H NMR (400 MHz, $CDCl_3$)

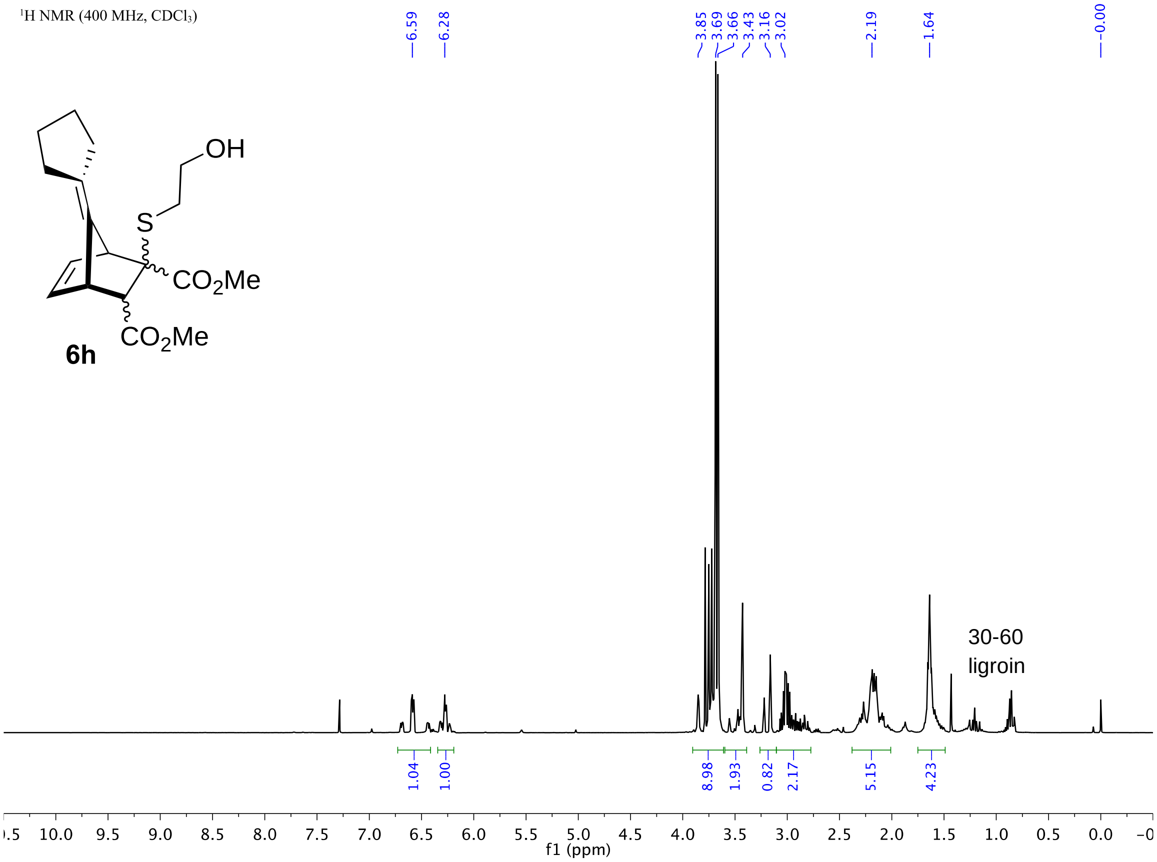

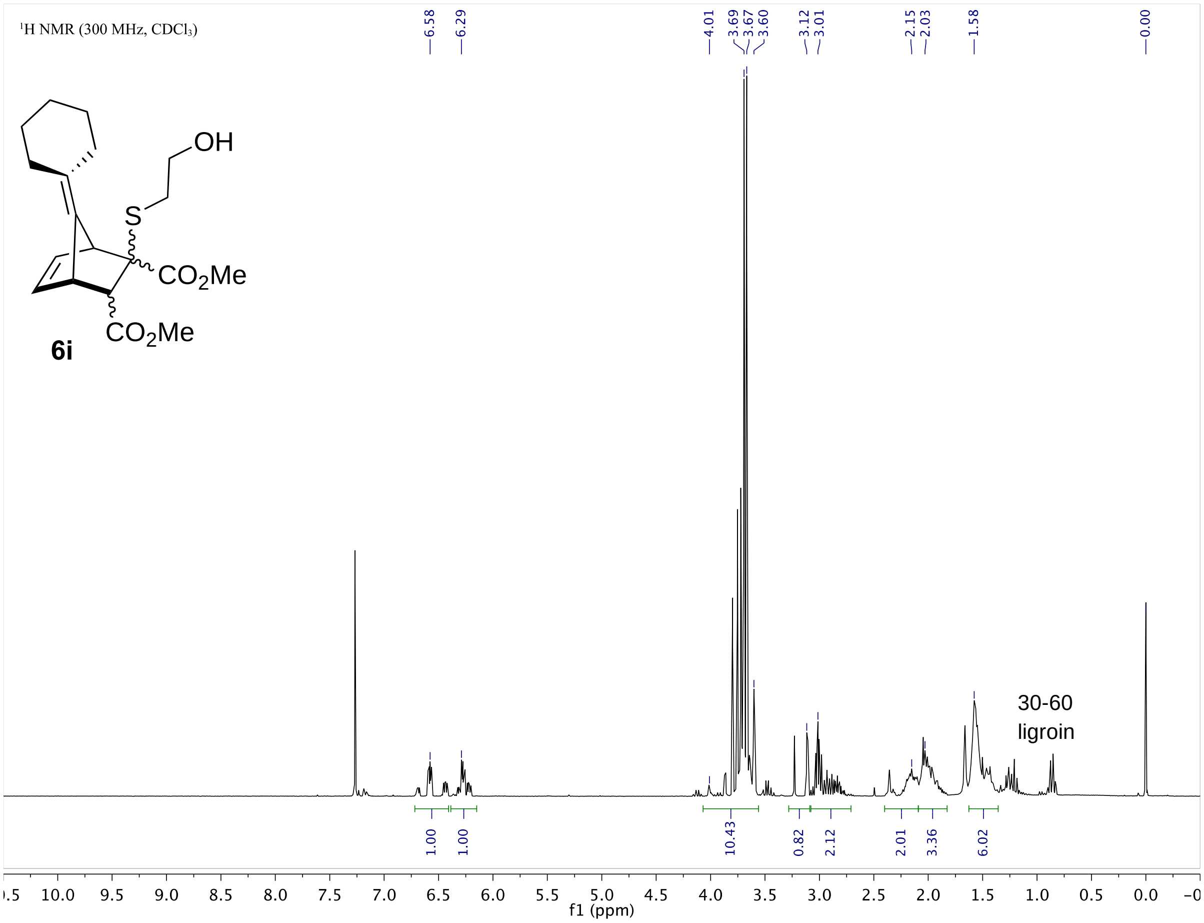

1H NMR (300 MHz, CDCl3)
6.58
6.29
4.01
3.69
3.67
3.60
3.12
3.01
2.15
2.03
1.58
0.00
OH
S
CO2Me
CO2Me
6i
30-60
ligroin
1.00
1.00
10.43
0.82
2.12
2.01
3.36
6.02
f1 (ppm)

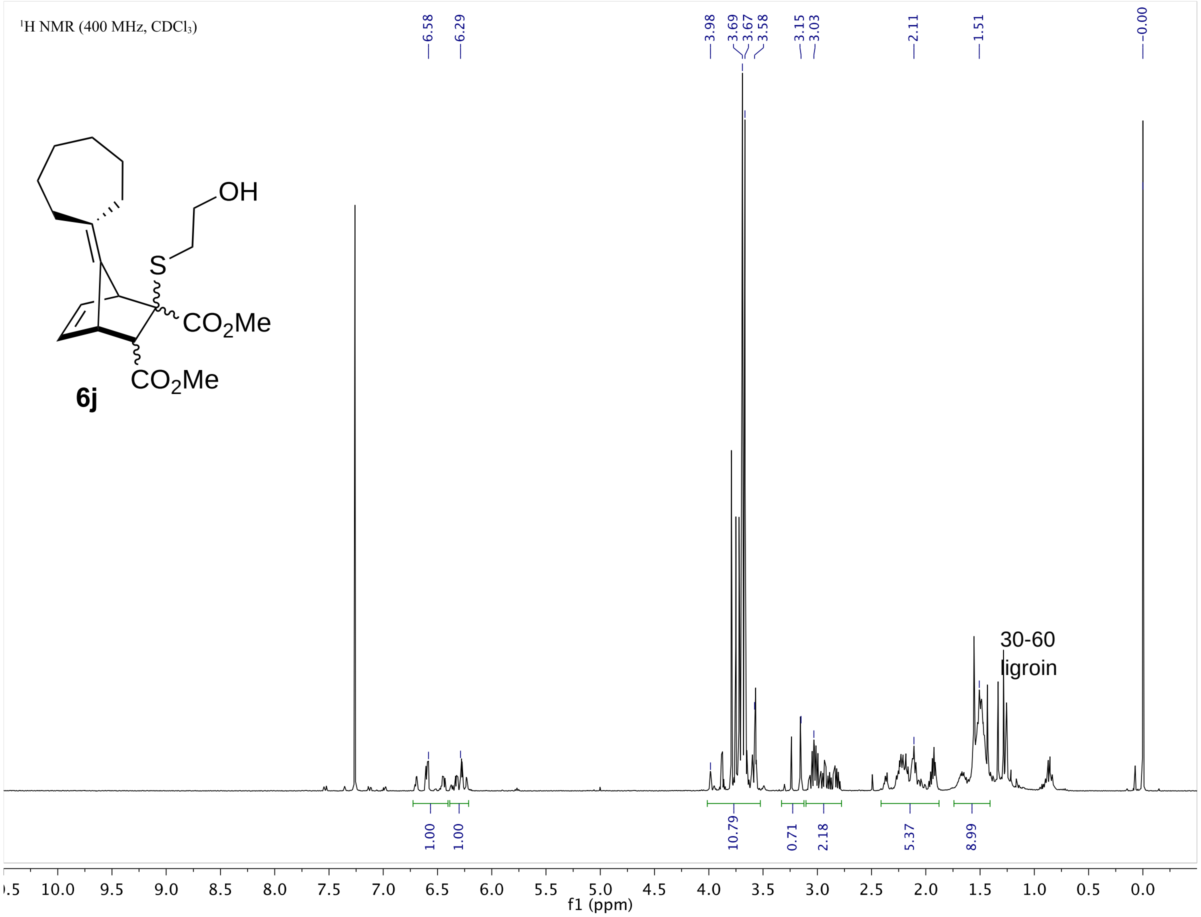

¹H NMR (400 MHz, CDCl₃)
6.58
6.29
3.98
3.69
3.67
3.58
3.15
3.03
2.11
1.51
0.00
OH
S
CO2Me
CO2Me
6j
30-60
ligroin
1.00
1.00
10.79
0.71
2.18
5.37
8.99
10.0 9.5 9.0 8.5 8.0 7.5 7.0 6.5 6.0 5.5 5.0 4.5 4.0 3.5 3.0 2.5 2.0 1.5 1.0 0.5 0.0
f1 (ppm)

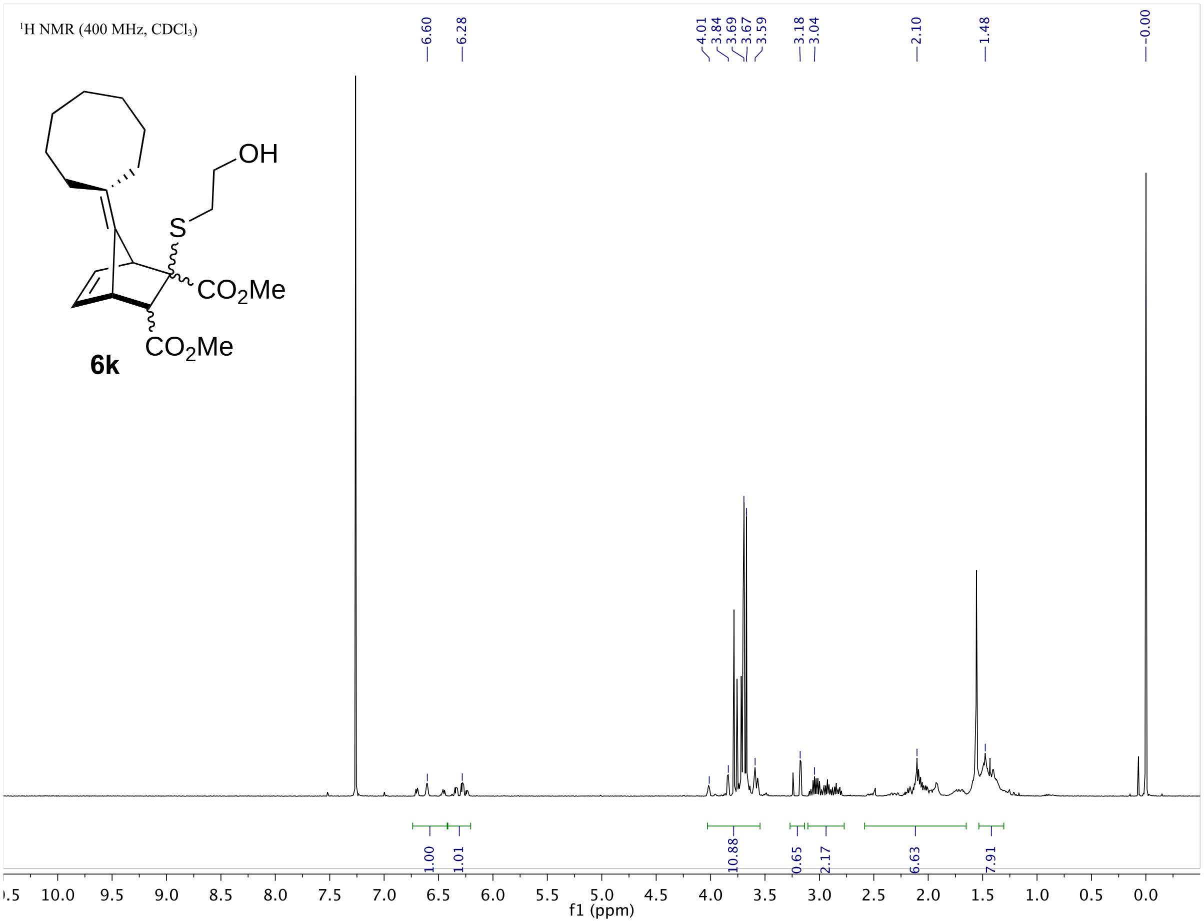

1H NMR (400 MHz, CDCl3)
6.60
6.28
4.01
3.84
3.69
3.67
3.59
3.18
3.04
2.10
1.48
-0.00
OH
S
CO2Me
CO2Me
6k
1.00
1.01
10.88
0.65
2.17
6.63
7.91
f1 (ppm)

^1H NMR (400 MHz, $CDCl_3$)

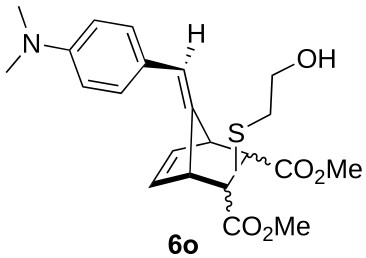

6o

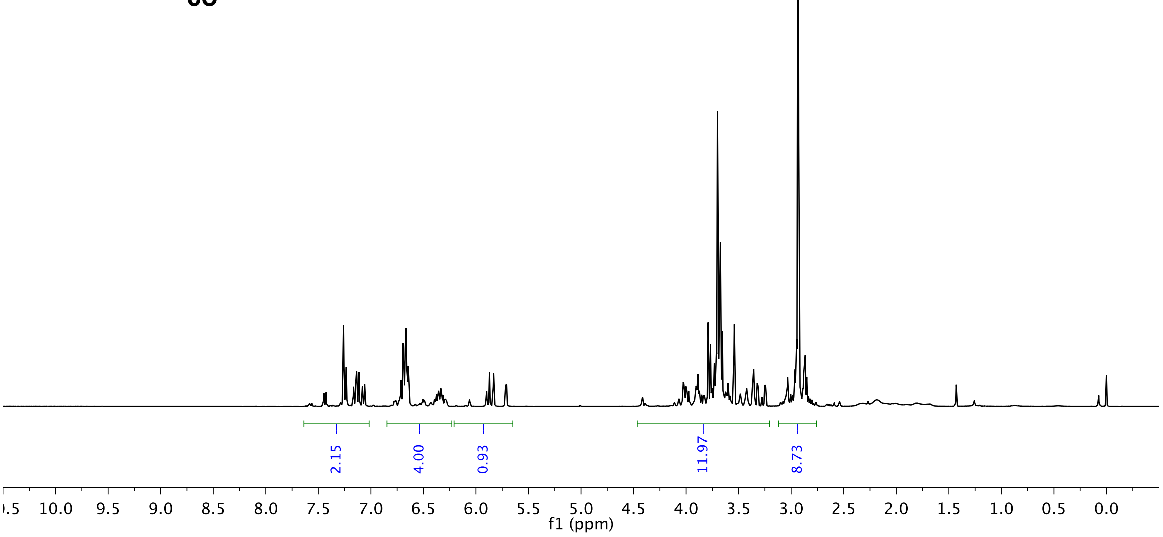

^1H NMR (400 MHz, $CDCl_3$)

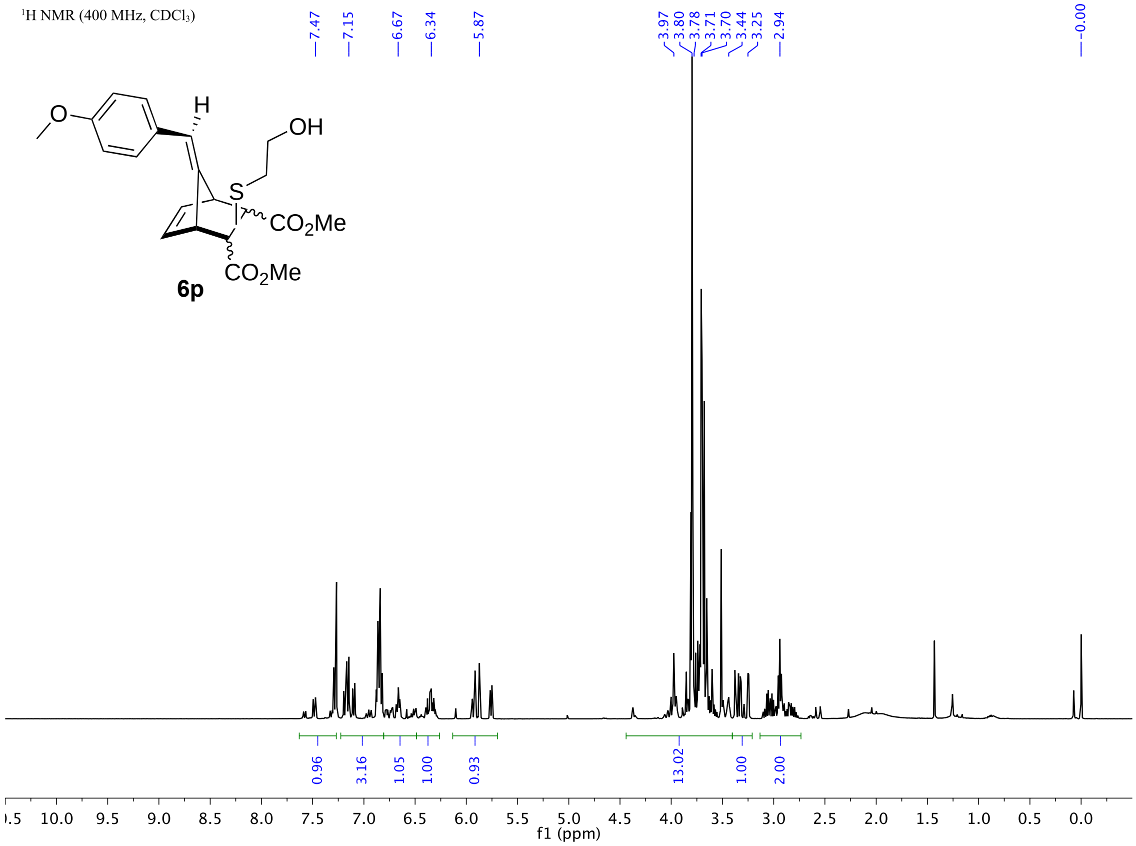

^1H NMR (400 MHz, $CDCl_3$)

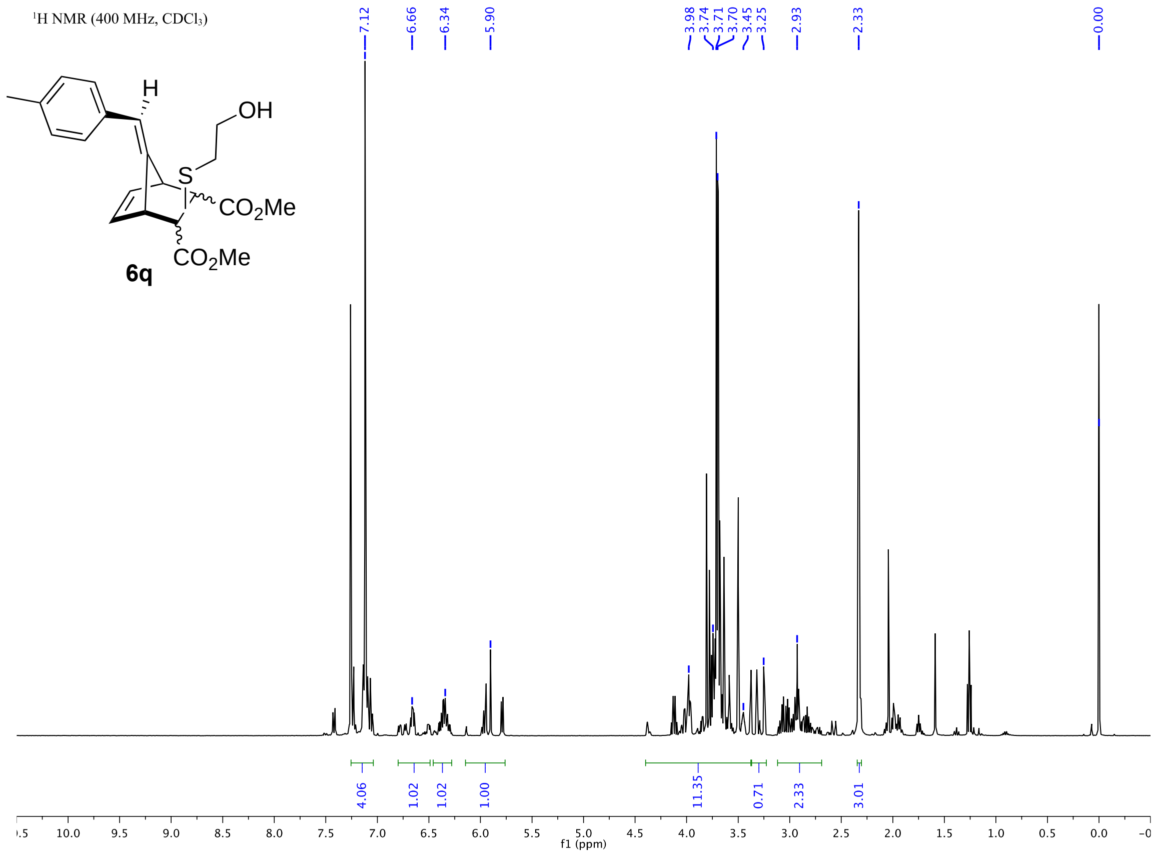

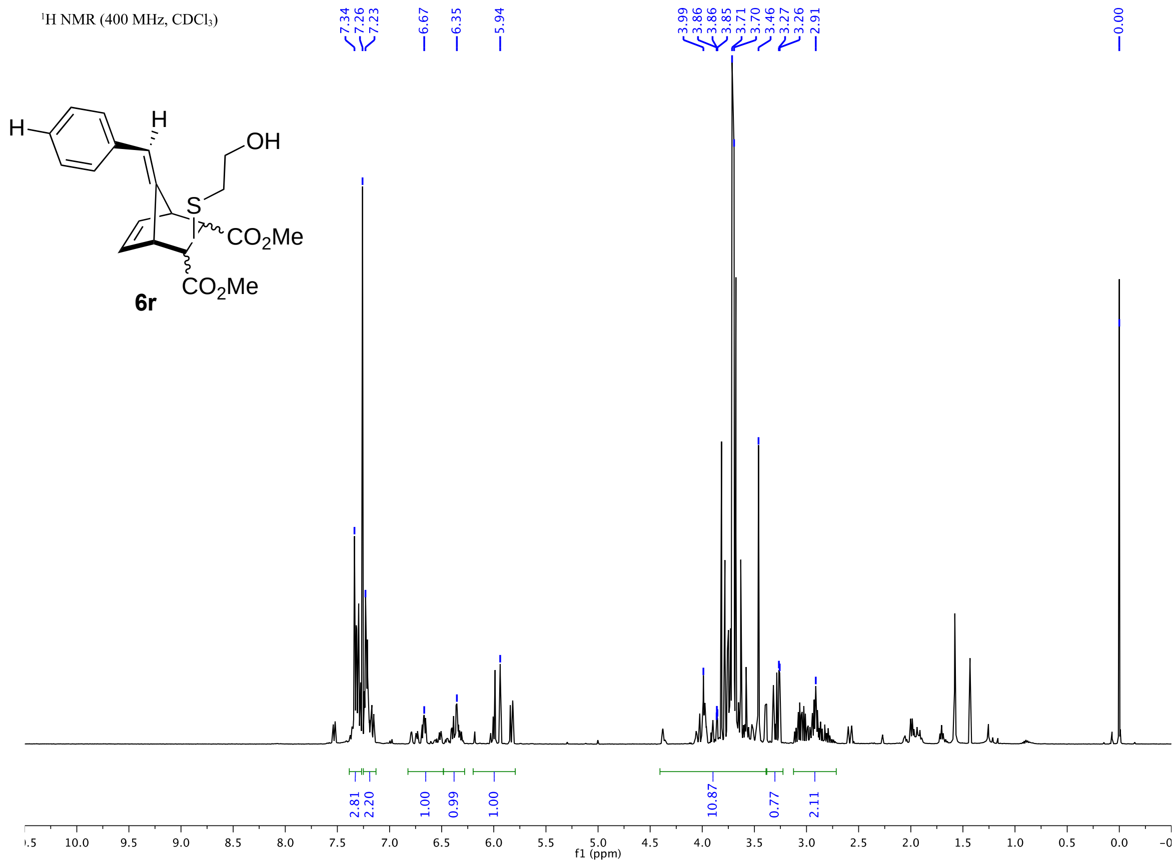

^{1}H NMR (400 MHz, CDCl$_3$)
7.34
7.26
7.23
6.67
6.35
5.94
3.99
3.86
3.85
3.71
3.70
3.46
3.27
3.26
2.91
0.00
H
H
OH
S
CO_2Me
CO_2Me
6r
2.81
2.20
1.00
0.99
1.00
10.87
0.77
2.11
f1 (ppm)

¹H NMR (400 MHz, $CDCl_3$)

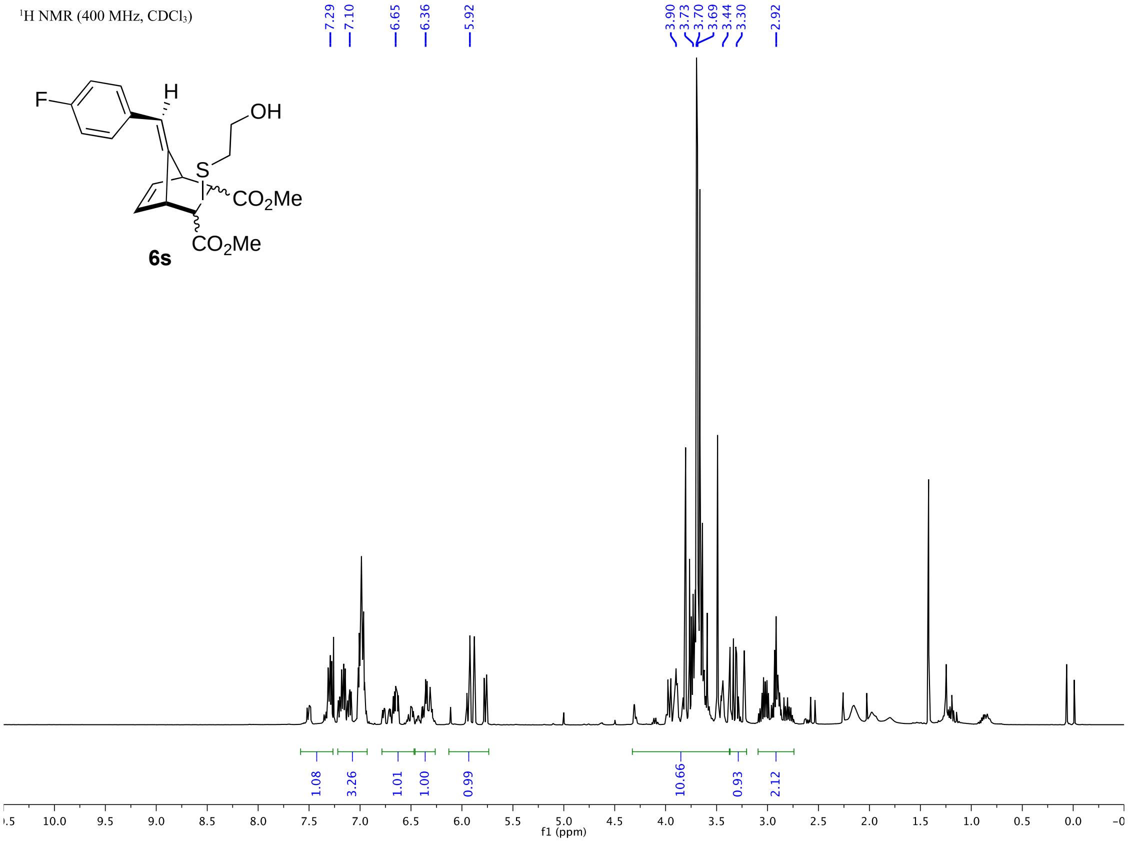

^{1}H NMR (400 MHz, Chloroform-*d*)

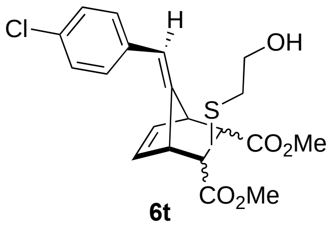

6t

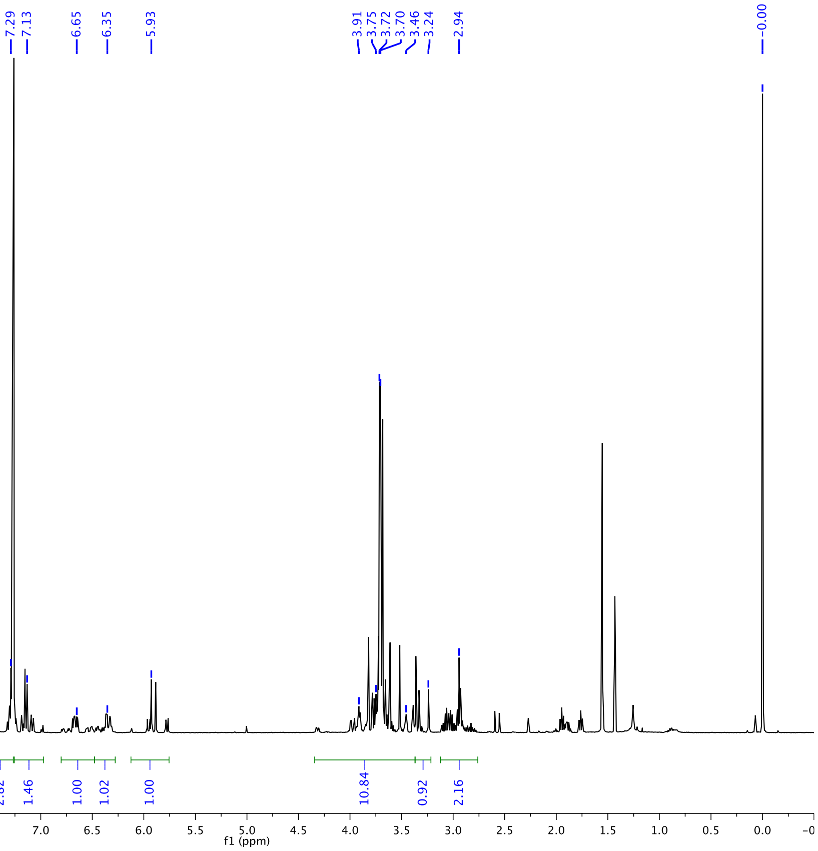

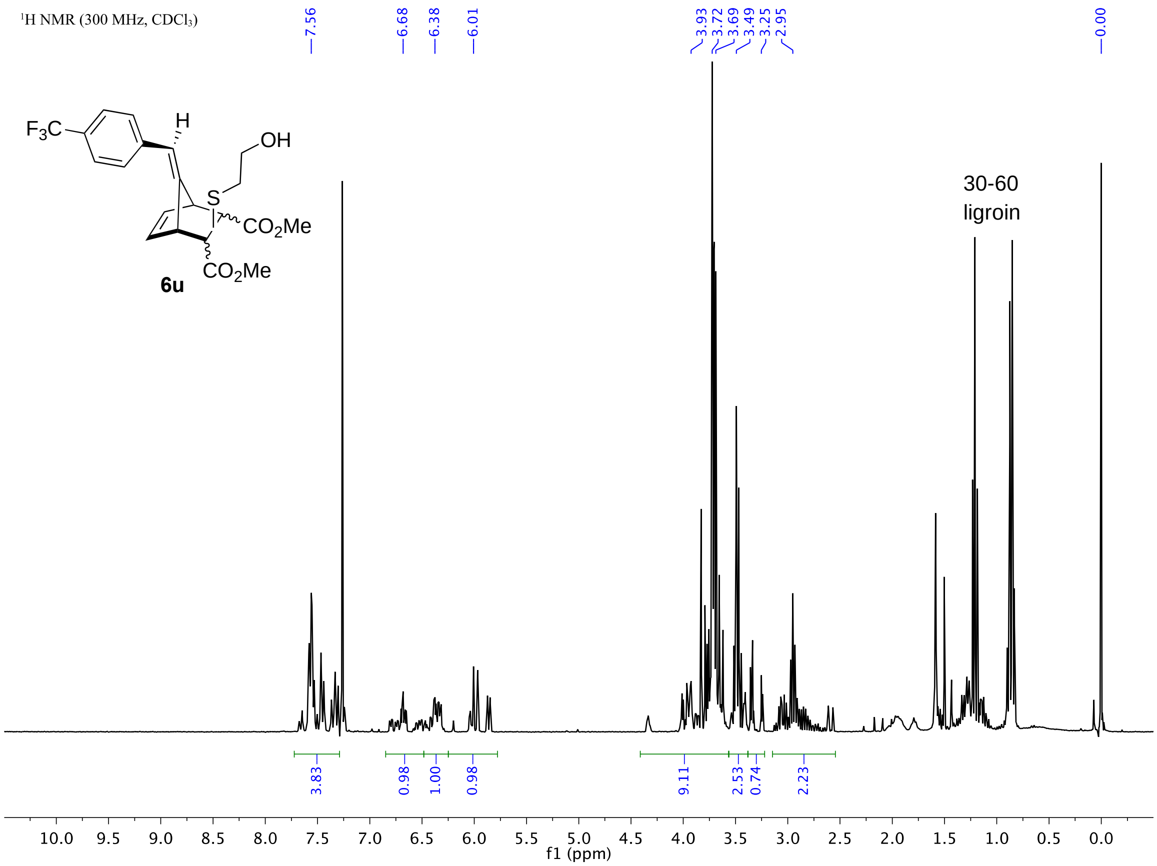

¹H NMR (300 MHz, CDCl₃)
7.56
6.68
6.38
6.01
3.93
3.72
3.69
3.49
3.25
2.95
0.00
F3C
H
OH
S
CO2Me
CO2Me
6u
30-60
ligroin
3.83
0.98
1.00
0.98
9.11
2.53
0.74
2.23
10.0
9.5
9.0
8.5
8.0
7.5
7.0
6.5
6.0
5.5
5.0
4.5
4.0
3.5
3.0
2.5
2.0
1.5
1.0
0.5
0.0
f1 (ppm)

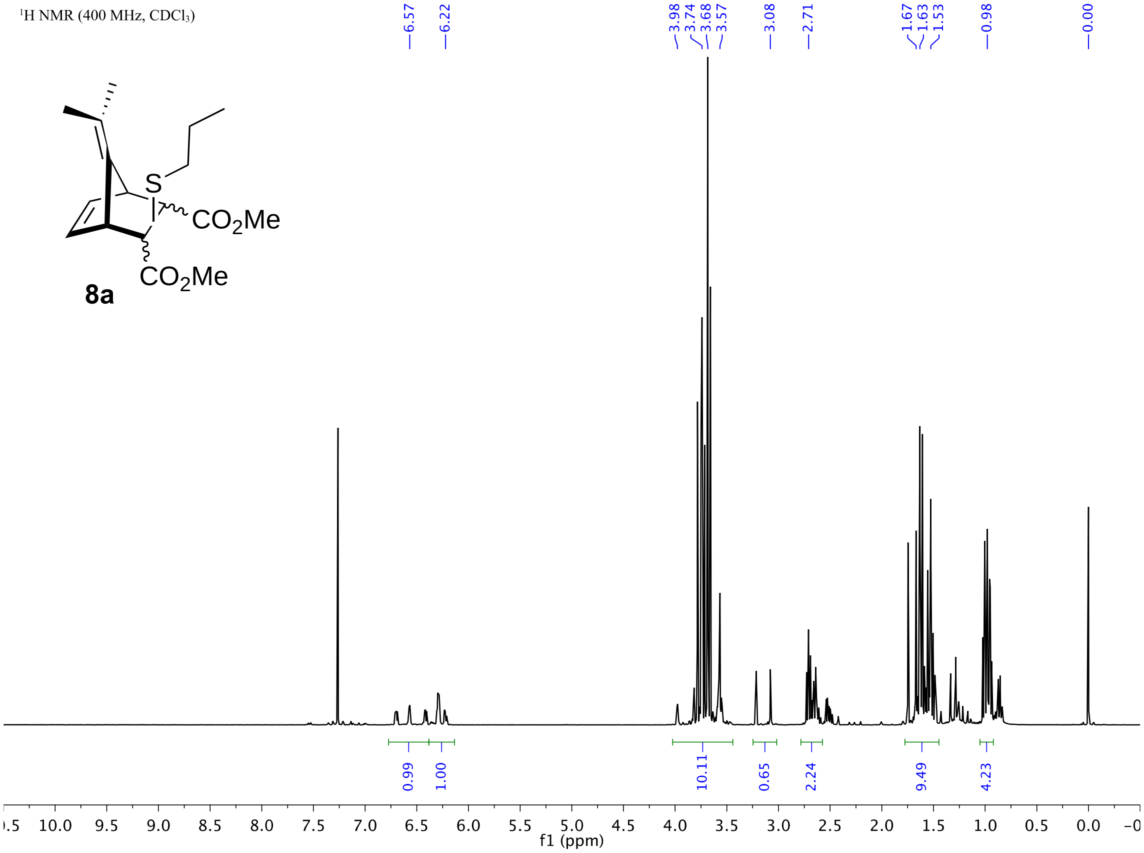

^{1}H NMR (400 MHz, CDCl$_3$)
S
CO$_2$Me
CO$_2$Me
8a
6.57
6.22
3.98
3.74
3.68
3.57
3.08
2.71
1.67
1.63
1.53
0.98
0.00
0.99
1.00
10.11
0.65
2.24
9.49
4.23
f1 (ppm)
